# Reference Case Methods for Expert Elicitation in Health Care Decision Making

**DOI:** 10.1177/0272989X211028236

**Published:** 2021-07-16

**Authors:** Laura Bojke, Marta O. Soares, Karl Claxton, Abigail Colson, Aimée Fox, Chris Jackson, Dina Jankovic, Alec Morton, Linda D. Sharples, Andrea Taylor

**Affiliations:** Centre for Health Economics, University of York, York, UK; Centre for Health Economics, University of York, York, UK; Centre for Health Economics, University of York, York, UK; The Department of Management Science, University of Strathclyde, Glasgow, UK; Centre for Health Economics, University of York, York, UK; MRC Biostatistics Unit, University of Cambridge, Cambridge, UK; Centre for Health Economics, University of York, York, UK; The Department of Management Science, University of Strathclyde, Glasgow, UK; London School of Hygiene and Tropical Medicine, London, UK; Leeds University Business School, Leeds, UK

**Keywords:** Decision-making, economic-evaluation, Elicitation, uncertainty

## Abstract

**Background:**

The evidence used to inform health care decision making (HCDM) is typically uncertain. In these situations, the experience of experts is essential to help decision makers reach a decision. Structured expert elicitation (referred to as elicitation) is a quantitative process to capture experts’ beliefs. There is heterogeneity in the existing elicitation methodology used in HCDM, and it is not clear if existing guidelines are appropriate for use in this context. In this article, we seek to establish reference case methods for elicitation to inform HCDM.

**Methods:**

We collated the methods available for elicitation using reviews and critique. In addition, we conducted controlled experiments to test the accuracy of alternative methods. We determined the suitability of the methods choices for use in HCDM according to a predefined set of principles for elicitation in HCDM, which we have also generated. We determined reference case methods for elicitation in HCDM for health technology assessment (HTA).

**Results:**

In almost all methods choices available for elicitation, we found a lack of empirical evidence supporting recommendations. Despite this, it is possible to define reference case methods for HTA. The reference methods include a focus on gathering experts with substantive knowledge of the quantities being elicited as opposed to those trained in probability and statistics, eliciting quantities that the expert might observe directly, and individual elicitation of beliefs, rather than solely consensus methods. It is likely that there are additional considerations for decision makers in health care outside of HTA.

**Conclusions:**

The reference case developed here allows the use of different methods, depending on the decision-making setting. Further applied examples of elicitation methods would be useful. Experimental evidence comparing methods should be generated.

## Background

Evidence on health benefits and resource use associated with health interventions may be required to inform health care decision making (HCDM), including assessments of cost-effectiveness.^1^ In a model-based analysis, key parameters, such as treatment effects, are not known precisely because of sampling uncertainty. There are often other limitations in the evidence; for example, the licensing of cancer products may be based on evidence of progression-free survival rather than overall survival or the evidence base may not be well developed (e.g., diagnostics, medical devices, early access to medicines, or public health).

It is important that the uncertainty in this evidence is quantified. If not, any analysis using this evidence may give decision makers a misleading view of the consequences associated with their decision.^2^ By quantifying uncertainty, it is also possible to assess the potential value of additional evidence.^3^ In these situations, the experience of experts is useful and, in some cases, critical to reach a decision. To ensure accountability in the decision, expert judgements should be explicit and their inclusion in HCDM transparent. The process by which beliefs of experts can be quantified according to scientific principles has been called structured expert elicitation (hereafter “elicitation”).^4^ When empirical evidence is unsuitable or does not exist, elicitation can provide point and interval estimates describing the state of knowledge for parameters required to make the decision. Where experimental evidence does not exist at all, the expert can use their knowledge of the parameter based on their observations (e.g., knowledge gained from clinical practice). Where the experimental evidence is unsuitable, for example in a different population, experts may be required to extrapolate from one population to another.

There is increasing interest in elicitation to inform HCDM, as new technologies are assessed progressively closer to their launch into the market. Elicitation may also be particularly valuable for early-stage cost-effectiveness models or for rare or emerging diseases, for which little or no evidence is available. A review of company submissions appraised by the National Institute for Health and Care Excellence (NICE) found that expert judgment is ubiquitous in company submissions (23/25).^5^ In the context of cost-effectiveness analysis, a review of applied studies in decision modeling for cost-effectiveness analysis found heterogeneity in the methodology used for elicitation, with little consideration of existing elicitation guidance reported.^6^

Elicitation has also been used widely in disciplines, including weather forecasting and engineering.^7^ Guidance that exists for elicitation in these contexts suggests several key issues to consider when designing, conducting, and analyzing an elicitation exercise, with multiple methodological choices at each stage. The preferred methods are inconsistent across different guidance; examples include the use of group- or individual-level elicitation methods.^8^

In addition to discipline-specific guidance, there are also published generic guidance documents. A number of these have been used in HCDM, the most notable being the Sheffield Elicitation Framework (SHELF)^9^ and Cooke’s classical method.^10^ Despite their use in HCDM, little is known about the suitability of methods proposed in these generic guidelines. Some of the methods recommended in generic guidance may not be suitable in HCDM, for example, the elicitation of complex quantities or the use of more complex methods. The reasons for this include resource and time constraints in HCDM; the types of experts typically consulted, usually recruited for their subject knowledge rather than quantitative background; and the wide range of parameters required for elicitation.^11^

In this article, we describe the development of reference methods for expert elicitation to inform HCDM. Details are reported in full elsewhere.^12^ The intention is for these reference methods to be used by a range of decision makers to generate their own guidance for expert elicitation, for example, across the globe and/or across different areas of HCDM. Here we describe the reviews undertaken to compile methods available for expert elicitation, the approach used to critique the different methods for expert elicitation and determine their suitability for use in HCDM, and finally the set of reference methods that were produced. Given the infancy of expert elicitation in HCDM and the lack of evidence to support many of the methods choices, we define these reference methods for one aspect of HCDM, health technology assessment (HTA). Thereafter, we highlight the complexities and challenges for HCDM outside of this setting.

## Methods

**Figure 1:**
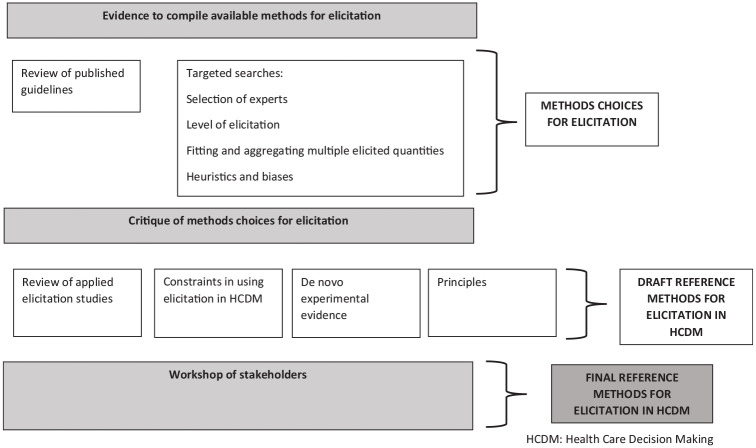
Evidence sources used to develop health care decision making (HCDM) reference methods for elicitation.

We conducted systematic and nonsystematic reviews of evidence to compile available methods (described in the section “Reviews of Evidence to Compile Methods for Elicitation”). To generate reference methods for HCDM, we then developed resources to critique the identified methods for elicitation (described in the “Critiques of Methods Choices for Elicitation” section). Details are reported in full elsewhere.^12^ We summarize the evidence sources and critique approaches in the sections below (“Determining Appropriate Methodological Choices for Elicitation in HCDM”). Figure 1 presents the broad structure of the evidence sources and critique methods.

### Reviews of Evidence to Compile Methods for Elicitation

We used a range of evidence sources. These sources are summarized below.

#### Review of published guidelines

We have undertaken a review of guidelines for elicitation published in either the peer-reviewed or gray literature. The elicitation guidelines were systematically reviewed according to the search strategy and inclusion criteria presented in the appendix (also detailed elsewhere^12^). This review (not restricted to HCDM) included guidelines concerning probabilistic judgments that offered guidance on more than 1 stage of the elicitation process.^12^ Information was extracted from these guidelines to create an overview of the sequential stages of the elicitation process (design, conduct, and analysis), the elements within each of these stages, and the choices involved in each of these elements (see the appendix for the extraction template), for example, training and preparation is an element of elicitation, which requires choices about whether and how to pilot the elicitation and what to cover when training experts.

We determined where current advice conflicts or agrees across guidelines. Where the guidelines agreed, we assumed this methodological choice represented best practice and accepted it as the reference case method.

#### Targeted searches

A priori, we were aware of many elements of elicitation that were not discussed in any depth in the existing guidelines. It was therefore not clear what methods choices were available for these elements. To augment the existing published guidelines, we conducted semistructured searches to identify the full set of choices for these elements.^12^ The searches also aimed to identify any agreed “best practice” for elicitation in these elements. Further details of the methods of the targeted searches are reported elsewhere.^12^ Specifically, we conducted targeted searches for 5 methods choices. The areas for targeted searches were chosen following consultation with our project advisory group and are as follows:

1. selection of experts,
2. level of elicitation (individual or group),
3. fitting of parametric models to elicited judgments and subsequent aggregation across multiple experts,
4. assessment of the accuracy of expert judgments, and
5. identification of cognitive heuristics and biases and methods to minimize the impact of these on the elicitation.

### Critique of Methods Choices for Elicitation

In the anticipated absence of definitive statements of agreement from the review of guidelines and targeted searches, it was necessary to critique the methods choices identified in “Reviews of Evidence to Compile Methods for Elicitation” section. We did this according to the principles for successful elicitation that we developed as part of this project (see the “Principles for Elicitation in HCDM” section). In addition, we augmented the critique using evidence on the choice of methods that might be suitable in HCDM from the applied studies review (“Review of Applied Studies” section),^11^ the constraints in using elicitation in HCDM that we identified (“Constraints in Using Elicitation in HCDM”), and conclusions drawn from experiments conducted as part of this project (“De Novo Experimental Evidence”). Full details are presented elsewhere.^12^

#### Review of applied studies

We have previously published a review of cost-effectiveness studies that include elicitation.^11^ The review considered the methods used and the specific challenges in conducting elicitation in this context. We identified 21 applied studies. Many authors expressed methodological uncertainty in justifying their choices. From the review, several aspects of the context area (HCDM) emerged as potentially important in determining methodological choices in elicitation. We used the findings from this review of applied studies to generate the core principles for elicitation in HCDM (“Principles for Elicitation in HCDM”) and also to critique the methods choices from the review of guidelines.^11,12^

#### Constraints in using elicitation in HCDM

In considering how reference methods for elicitation in HCDM might be used in practice, it is important to understand how different decision-making settings may influence the requirements for, and practicalities of, elicitation. We considered the potential practical constraints of using elicitation in HCDM at various levels of decision making when generating the principles and assessing the applicability of methods identified in the review of published guidelines. A formal review of the challenges and constraints faced by different HCDMs was not possible. Instead, this source of evidence drew on the observations and experiences of the authors and an advisory group convened as part of the project (see the Acknowledgements for details of this group). See Bojke et al.^12^ for further details.

#### De novo experimental evidence

As part of this work, we generated evidence from randomized simulation experiments to compare method choices for elicitation.^12^ Randomization concerned the level of precision specified in the scenarios presented to participants for experiment 1, the distributions of subgroups for extrapolation for experiment 2, and the degree of discordance between individual and group summaries in experiment 3. Randomization was undertaken to explore multiple scenarios within each of the experiments while not overburdening participants. It also helped to standardize other aspects of the participant’s knowledge, such as their level of training in probability and statistics.

Specifically, the experiments concerned 1) different methods to encode experts judgments, the variable interval method, and the fixed interval method; 2) requiring experts to extrapolate from their individual knowledge to populations with different prevalence of a successful outcome, and 3) the use of Delphi-type processes to understand how experts revise their estimates in the light of group summaries.^12^ The experiments were conducted using the Shiny package for R.

To conduct these experiments, we used a simulated (virtual) learning process to standardize participants’ knowledge. This allowed us to compare the elicited probabilities directly to the distribution implied by the observed data set, therefore providing a measurement of accuracy. Two main metrics were used: 1) bias in location (difference in the mean of the elicited distribution and the posterior distribution implied by the data) and 2) bias in uncertainty (the ratio of the standard deviation of the elicited distribution to the standard deviation of the posterior distribution implied by the data).

In the first experiment, each participant was shown observations from a stochastic simulation model. The context was an abstract generic medical problem. The participants were asked to choose between treatments with differing levels of effectiveness. Experiments 2 and 3 followed on from the context specified in experiment 1 and 3. We report further details of the methods used in these experiments elsewhere.^12^ We use the results from these experiments to provide additional information on the suitability of methods arising in these three areas, from the review of guidelines.

#### Principles for elicitation in HCDM

These principles are primarily informed by the findings of the published review of the use and challenges in applying elicitation in cost-effectiveness modeling (“Review of Applied Studies” section)^11^ and the review that identified constraints in using elicitation in HCDM more generally (“Constraints in Using Elicitation in HCDM” section).^12^ We also reflect the requirements for elicitation reported in the guidance by Cooke.^10^ These requirements represent “good practice” in elicitation generally and are widely referred to in the elicitation literature.

We first drafted the principles and then amended these following a meeting with our advisory group, convened to guide the project. The advisory group consisted of elicitation methodologists and users (see the “Acknowledgments” section for the list of advisory group members). We presented the redrafted principles at the workshop described in the next section.

### Determining Appropriate Methodological Choices for Elicitation in HCDM

We have made recommendations for each element within the stages for elicitation by assessing which choices are supported by the principles or the evidence and which could be left flexible according to the specific elicitation context. We convened a stakeholder workshop, in which we presented our draft reference methods for elicitation in HCDM, for the purposes of gaining feedback and establishing validity. We identified the relevant stakeholders as HTA decision makers, methodologists, industry representatives, and commissioners. To gather stakeholders, we reached out to UK decision makers, including those from NICE, NHS England, and Public Health England; authors of the elicitation articles identified in our applied studies review^11^; and key contacts in industry and consultancy. Approximately 30 stakeholders attended the event.

We gathered opinion through presentation and discussion, followed by communication with specific individuals who wished to speak about the topic outside of the meeting. Following feedback from this workshop, we generated a set of final reference case methods. The workshop also sought to identify challenges in using elicitation in different settings, for example, where evidence is immature or where decisions concern orphan drugs. We documented these challenges along with the examples of areas in which they arose.

## Results

### Evidence to Inform the Set of Choices

#### Review of published guidelines

We identified 16 unique guidelines (see the appendix for the search results and the full list of included guidelines; see Bojke et al.^12^ for further details). Five are generic ^9,13–16^ and 11 are domain specific.^17–27^ The guidelines include the widely cited European Food Safety Agency guideline,^25^ Cooke’s Classical Model,^13^ and SHELF.^9^ Although some of these guidelines have been used in HCDM, for example, SHELF^9^ and the IDEA protocol,^15^ none were developed specifically for this context, and none discuss their applicability to HCDM.

Details of the elements and methodological choices contained in existing guidelines are presented in the appendix. In addition, we present, for each methodological choice, the level of agreement between existing guidelines (see the appendix). There are relatively few methods choices for which the existing guidelines entirely agree. Areas of agreement are 1) the need to decompose (breakdown) variables into several smaller, more observables quantities; 2) the number of experts should be between 5 and 10; 3) the roles of experts within the elicitation task should be made explicit; 4) there should be piloting of the task; 5) experts should provide rationales for their judgments; and 6) aggregation should be undertaken after elicitation. There are many methodological choices for which guidelines have only partial agreement on the appropriate choice or else no agreement at all.

#### Targeted searches

In the 5 areas subjected to targeted searches, there is very little empirical evidence to support or discount any specific choices, and none of the evidence that does exist focuses on HCDM.^12^ Any conclusions offered on these elements are generally anecdotal rather than empirically based. For example, regarding the minimization of bias, there is a suggestion that experts should not be asked to express confidence intervals in a single-stage process, as doing so results in participants focusing on a narrow set of salient possibilities. Instead, lower bounds, upper bounds, and median values should be elicited separately.^9,15,19^ Full details of the targeted searches results are reported elsewhere.^12^

### Resources to Critique Methods Choices

The review of applied studies (“Review of Applied Studies” section), the constraints that may have implications for elicitation in HCDM (“Constraints in Using Elicitation in HCDM”), and the evidence generated from the experiments (“De Novo Experimental Evidence”) are reported in detail elsewhere, and results are therefore not repeated here.^11,12^ Instead, we describe the principles that were generated and refer to evidence from the “Critique of Methods Choices for Elicitation” section in the critique of methodological choices section below (“Critique of Methodological Choices for Elicitation in HCDM”).

We developed 9 principles for judging the suitability of choices available for elicitation. These are summarized below. Workshop participants agreed unanimously that these represented a complete set of requirements for elicitation conducted in HCDM, with stakeholders suggesting only minor changes to the wording.

#### Principle 1: Ensure transparency in elicitation

Systematic and transparent reporting of elicitation helps to improve the validity of the resulting expert judgments, allows the elicitation to be peer assessed, and supports others who use the judgments in their own analysis. If there is insufficient space to describe the elicitation process in the primary study report, separate details of the elicitation, ideally comprising an elicitation protocol and results, should be fully documented.

#### Principle 2: The elicitation must provide useful information for the decision problem

The elicitation must be fit for purpose, in that it must provide information that is relevant to the decision problem. If a decision model is employed by the analyst to synthesize evidence to determine cost-effectiveness, then the quantities being elicited should be consistent with the parameters and structure of the model. For example, suppose we believe that 2 model parameters are likely to be correlated, such that a belief that 1 parameter is high implies belief that the other one is high. In these circumstances, an elicitation designed to inform these parameters should give information about their correlation (e.g., by eliciting the second quantity conditionally on the first). Multiple quantities must also be mathematically consistent; for example, probabilities of mutually exclusive events should sum to one.

#### Principle 3: Elicitation should aim for consistency but respect the constraints of the decision-making context

There are different potential users of elicitation, from local level to national or international decision makers, including reimbursement agencies and research funders. These different decision-making entities have quite different capacities to conduct elicitation and incorporate it into their decision-making processes. It is important that a degree of flexibility is retained in the reference case for elicitation in HCDM, but the sensitivity of results to the choices made should be explored.

#### Principle 4: Elicitation should reflect uncertainty at the individual expert level

Judgments elicited from experts need to reflect the imperfect knowledge they have. In elicitation, experts are often required to provide both a point estimate of the quantity(s) of interest and an assessment of their uncertainty in that estimate. An important concern is that, when reflecting on their own experiences, experts may mistakenly report the extent of variability (e.g., between disease outcomes for individuals) rather than uncertainty in knowledge (e.g., about the expected incidence rate of the outcome).

#### Principle 5: Elicitation should recognize and act on biases

There are many biases and heuristics (cognitive shortcuts that individuals use when asked for complex judgments) that apply to elicitation, including overconfidence/underconfidence, overextremity (tendency to use the extremes when responding), discrimination (including prejudice or stereotyping), or susceptibility to base rate neglect (*a disregard of fundamental statistical reality)*. An elicitation task should be designed and conducted using techniques that mitigate against heuristics and other sources of bias, and appropriate training should be given to experts.^14^

#### Principle 6: An elicitation task should be suitable for experts who possess substantive skills and who are less likely be trained in probability and statistics

Substantive experts in HCDM are often health professionals who are unlikely to have had extensive experience of elicitation and unlikely to have developed the necessary normative skills (e.g., in probability and statistics). Methods of elicitation employed in other areas may not be directly suitable in HCDM unless there is additional training before use.

#### Principle 7: The elicitation task should recognize where adaptive skills are required

In some instances, adaptive skills may be relevant for elicitation in HCDM. For example, in early cost-effectiveness modeling or early-stage trial design, experts may not be familiar with the target quantity for elicitation but are substantive experts in 1 or more related quantities (for example, the quantity in a similar population to the target population). In this situation, knowledge of the related quantity may need to be adapted.

#### Principle 8: Elicitation should recognize, and act on, between-expert variation

In the context of HCDM, between-expert variation is common. There may be genuine heterogeneity in the populations that experts draw upon to formulate their judgments. In this case, it is desirable to reflect this heterogeneity in the pooled distribution, whether through group consensus or mathematical aggregation methods. It is also important to understand why between-expert heterogeneity is present.

#### Principle 9: Elicitation should promote high performance

In HCDM, experts may be motivated to undertake the task to the best of their abilities because of their interest in the topic area and for altruistic reasons. However, not all experts within an elicitation may possess the same subject knowledge, and there may be differences in normative (e.g., probability and statistics) expertise. Where possible, an elicitation task should account for differing levels of normative expertise and encourage experts with substantive knowledge to perform equally, for example, in providing unbiased estimates. As well as promoting high performance, an elicitation may want to explore differences in expert performance.

### Critique of Methodological Choices for Elicitation in HCDM

The critique determined the suitability of elicitation methods according to their adherence to the principles of elicitation for HCDM reported here (see the appendix for full details of which principles are applied to which methods choices), the results from the experiments, and the constraints.^12^

#### Selecting quantities (preparation and design)

A key requirement is that the elicited information should be fit for purpose and describe an expert’s uncertainty regarding the quantity of interest. Experts in HCDM are often recruited because of their subject expertise and may be less likely to have statistical expertise. To aid completion by experts in HCDM, existing guidelines are consistent about the need to break down variables into simpler quantities to elicit.

Despite the lack of empirical evidence to support this assertion, we believe that questions should be posed in a manner consistent with how experts express their knowledge. As a result, elicitation tasks should specify observable quantities, such as probabilities (expressed as proportions or frequencies), and more complex quantities such as odds ratios or variances should be avoided. The use of observable quantities may aid experts when they are required to extrapolate outside of their knowledge base. The experiments we conducted concluded that such extrapolation is unlikely to produce more biased judgments or more inaccurate expressions of an expert’s uncertainty.

In some circumstances, the quantities elicited may have a degree of dependency. In HCDM, the aim should be to ask about independent quantities where possible.^9,13,15,17,21–27^ If this is not possible, dependent quantities can be r-expressed in terms of independent quantities or conditional quantities, or dependence methods can be used.^15^

#### Methods to encode judgments (preparation and design)

In general, existing guidelines suggest that both the fixed interval method and the variable interval method can be used to encode judgments.^9,13,16,17,20,23,25–27^ Because experts may be recruited primarily because of their substantive skills, the suitability of alternative methods must recognize differences in their normative (e.g., probability and statistics) skills. Evidence from our experiments suggested that the fixed interval method and the variable interval method are equally appropriate for HCDM in terms of providing accurate representations of an expert’s uncertainty, although there is some preference for the fixed interval method, delivered using a “chips and bins” approach.^12^ Decision makers may therefore choose either but should apply them consistently in their setting.

#### Selecting experts

The existing guidelines and targeted searches suggest features to consider when selecting experts. These include normative expertise, substantive expertise, and willingness to participate. The constraints of conducting elicitation in HCDM may dictate that the selection process focuses on only 1 or 2 key characteristics. It is worth noting that there may be a limited number of health care professionals with the relevant substantive expertise, and therefore, more opportunistic methods for recruitment may be required, such as peer nomination. In some instances, adaptive skills may be required for an elicitation, particularly in the case of new and emerging technologies. It is not clear what metrics can be used to determine an expert’s level of adaptive skills.

Identifying an unbiased expert poses a challenge, and indeed, an entirely unbiased expert may not exist. The targeted searches showed that the elicitation should attempt to recruit experts who are free from motivational biases by collecting disclosure of personal and financial interests and conflicts of interest.^9,14,19,22,27^ In addition, efforts should be made to ensure that the sample of experts contains a range of viewpoints, with the intention of “balancing out.”^13,15–17,19,20,22^ This may dilute the effect of motivational biases.

Between-expert variation may exist, and methods used to select experts must attempt to capture the range of plausible beliefs. Identification of experts through recommendations by peers, either formally or informally, may generate a pool of experts that are all similar. Instead, it may be preferable to identify experts through research outputs, by known experience, or by using a profile matrix. The elicitation can also seek diversity in background and a balance of different viewpoints. Recruiting a larger number of experts may help to fulfill these criteria (5–10 experts are suggested by the existing literature identified in the targeted searches^12^).

#### Piloting and training

All existing guidelines agree that an elicitation should be piloted on a smaller set of experts prior to the actual task, with subsequent revision based on feedback and follow up of any issues that arise. For example, priors that are incoherent may indicate the need to respecify the quantities elicited or the questions asked.

Training of experts is essential and should focus on enabling nonnormative but substantive experts to express their uncertain beliefs at an individual level. Training also plays a key role in minimizing biases. Although evidence in the context of HCDM is weak, there are some suggestions from the literature that training can reduce the effect of anchoring and adjustment bias, confirmation bias, and overconfidence.

The training delivered to experts will be guided, in part, by the specific task, and include, for example, the description of quantities, the description of the performance measurement, and how to manage dependence. The core elements of training are a description of what is required from the experts, an outline of the elicitation process, an outline of the questions that will be asked, and example and practice questions.^12^

#### Level and conduct of elicitation

Existing guidelines are inconsistent regarding whether elicitation should be individual or group based.^12^ Group discussion can help experts with less clinical knowledge or probability and statistics training. However, interaction between experts can also introduce biases, and the act of striving for consensus can potentially eliminate important between expert variation. The constraints apparent in HCDM, such as limited access to experienced experts and short time scales for decision making, may also discourage the use of consensus methods.^12^ In addition, there is no evidence from our experiments that those who revise their judgments following group feedback have different accuracy than those that who did not revise their judgments, which casts some doubts on the benefits of the Delphi iteration process. For these reasons, we believe it is preferable to elicit from experts individually.

When using individual elicitation, there should still be possibilities for interaction between experts. This should follow on from the individual elicitation where practically feasible and useful. For consensus methods, again, the elicitation should first conduct individual elicitation followed by the group consensus stage. Feedback should follow the elicitation task, with graphical feedback considered for experts unfamiliar with probability and statistics.

Many of the existing guidelines agree that face-to-face administration is preferred.^9,13,17–22,24^ It is thought to promote good performance and maximize engagement with experts. Face-to-face elicitation is necessary for some consensus methods; however, it is not necessary for aggregating judgments mathematically. The constraints in HCDM are the biggest factors in driving the method choice. If the task requires many experts, face-to-face elicitation may be prohibitively time-consuming and resource intensive.

#### Aggregation, analysis, and postelicitation

The existing guidelines agree that, following elicitation of judgments, there should be an aggregation of the elicited information across experts. In the context of HCDM, however, aggregation should not simply focus on reducing variability between experts; instead, efforts should also be made by the elicitation facilitator to understand the reasons for any variability. To generate an aggregate summary (e.g., for use in a probabilistic decision model), it is necessary to fit a probability distribution. For the purposes of using the elicitation results in further analysis, a smooth fitted distribution is preferred to an empirical summary (without fitting). The choice of distribution will depend on the quantity elicited. Parametric distributions (e.g., normal, beta, or gamma) may be appropriate. The best-fitting distribution should be determined using standard statistical methods (e.g., ordinary least squares, generalized method of moments, or maximum likelihood). Simple mathematical rules for aggregation, such as linear opinion pool with equal weights, are the most commonly applied in HCDM and are straightforward to implement.

Documentation of the elicitation design, conduct, and analysis is key to understanding the choices made and the rationale for these. It is also important in assessing the validity of the distributions elicited. Details should be reported in the body of a report if possible and as a separate appendix if not. The documentation should include the justifications given by the experts, for their judgments.

#### Managing biases

There is very little in the existing guidelines on methods to minimize bias. The targeted search conducted to identify methods to minimize cognitive heuristics and biases^12^ suggests that efforts should be made to identify the likely biases given the type of experts who have been recruited. Relevant strategies to minimize these should then be employed.^12^ To mitigate biases, experts can be told as part of their training about the likely sources of bias and asked to be aware of these when responding to questions. In addition, questions can be framed in a way to minimize bias and ambiguity. This could include asking experts to first specify their plausible upper and lower bounds and giving experts the opportunity to revise the information they provide.

#### Validation

Commonly discussed elements of validation include verifying that the elicitation captures what experts truly believe and that the expressed probabilities reflect reality. Above all, validation should focus on the extent to which the elicited beliefs are fit for purpose for the intended task. This could be assessed by coherence and consistency with the intended HCDM it is to inform (i.e., an assessment of face validity). It is also important to understand how experts formulate their beliefs and why they present heterogeneous beliefs. An external review of the elicited priors, by experts not involved in the elicitation task, should be undertaken to assess validity.

### Generating Reference Case Methods for Elicitation

The sparse evidence supporting the methodological options in elicitation means that, for many elements, there remains uncertainty about the most appropriate choices, and further research is necessary. The previous section lays out considerations that are required to generate reference case methods for elicitation in HCDM settings. These are also reported in detail elsewhere.^12^ This critique helps to highlight the tradeoffs required when developing context-specific methods, where we need to take into account not only accuracy but also context-specific features, restrictions, and constraints.

Elicitation can inform HCDM in diverse settings, ranging from local-level prioritization to strategic planning for emerging threats. It has, perhaps, been most frequently applied in national level reimbursement, price negotiation, and clinical guideline development,^7^ collectively referred to as HTA. We have developed an exemplar set of reference methods for elicitation in the HTA context (see Table 1).

**Table 1:** Reference Case for Health Technology Assessment (HTA)

Element	Reference Methods Suggested	Additional Considerations outside of HTA
Selecting quantities	1. Simple observable quantities should be elicited where possible; ratios or complex parameters such as regression coefficients should not be elicited directly. 2. Dependence between variables should be captured in elicitation. Expressing dependent variables in terms of independent variables is preferable when experts do not have strong normative skills. 3. Wording should be clear and quantities should be decomposed where this means a better fit with experts intuition.	—
Methods to encode judgments	Both variable interval methods or fixed interval methods can be used. Decision makers should aim for consistency across applications.	Fixed interval methods may be more appropriate for experts less familiar with elicitation or where face-to-face training is impossible.
Selecting experts	1. Recruitment will be driven by the context; however, the elicitation should pursue diversity, representing the full range of valid expert beliefs. Experts should be willing to participate. 2. Focus on gathering substantive expertise or experience. Normative skills (for example, in probability and statistics) can be developed during the training session as part of the elicitation. 3. Minimize and record conflicts of interest among the experts. Include experts external to the elicitation task (i.e., not those involved in developing the task). 4. At least 5 experts should be included in the elicitation.	1. Researchers may have limited access to sufficient experts, for example, in rare diseases; therefore, expert recruitment may be more challenging and rely on peer nomination. 2. Adaptive skills may be required for new technologies since indirect evidence may outweigh directly relevant evidence (e.g., childhood diseases may be informed by adult versions with some extrapolation and appropriate weighting).
Piloting and training	1. Training is crucial and should focus on avoiding bias and expressing uncertainty. 2. Piloting should be undertaken.	—
Level and conduct of elicitation	1. Beliefs should be elicited from experts individually, even if a group interaction follows. 2. Interaction between experts should be structured through face-to-face sessions. 3. Between-expert variation should be explored explicitly. 4. Face-to-face where possible to allow a facilitator to deliver training to the expert. 5. Feedback to experts should be given during the elicitation. Following feedback, experts should be given an opportunity to revise their distributions, either during or after an elicitation session.	Group discussion may be needed to generate a distribution, for example, in early technologies or when eliciting more abstract/complex (nonobservable) quantities cannot be avoided, such as those relating to service delivery, public health programs, or patient pathways. Practical constraints may dictate remote delivery of elicitation, for example, though video conferencing.
Aggregation, analysis, and postelicitation validation	1. Probability distributions should be fitted to individually elicited judgments. 2. Following fitting, a summary of the individual distributions should be obtained using linear pooling with equal weighting of experts. 3. Any adjustments applied should be to improve coherence and consistency and not to reduce variability. Internal and external review can be used to assess validity. 4. Rationales for how the experts made their judgments should be collected and recorded after elicitation. 5. All methodological choices for the elicitation must be documented and justified.	1. Pooling methods, other than linear pooling, may better accommodate expert heterogeneity. Further research is needed to explore which methods are most appropriate in these circumstances. 2. Weighting may be preferable in some circumstances, for example, where experts represent different disciplines or contribute different perspectives on the elicited quantities and therefore considerable heterogeneity is anticipated, but a single agreed consensus distribution is required. Weighting may be achieved implicitly through consensus or explicitly through performance weighting, although it is difficult to see how performance scores would be generated in this context.

In summary, our reference case methods state that, in HTA, the elicitation should focus on gathering substantive expertise or experience. Elicitation skills can be developed during the training, which should focus primarily on avoiding bias and expressing uncertainty. In recruiting experts, conflicts of interest should be minimized and if necessary recorded. Experts external to the elicitation task should be included (i.e., not those involved in developing the task). Beliefs should be elicited face to face and from experts individually and then pooled. Between-expert heterogeneity should be explored explicitly. Simple observable quantities should be elicited where possible, with efforts made to capture dependence between quantities in a way that can be elicited reliably. Either the variable interval method or the fixed interval method can be used, with the choice depending on which best suits the type of expert and the elicitation task.

Although these reference methods are intended to reflect emerging best practice in HTA, given the infancy of elicitation applied to HCDM, it is important to allow a degree of flexibility in the reference methods recommended here. A decision maker adopting this protocol could choose to specify methods for the reference case to ensure greater consistency across appraisals. In cases in which nonreference case methods are employed, choices should be justified and sensitivity analyses undertaken.

Elicitation may also be useful for decision makers outside of HTA, for example, at a local level or in the context of the appraisal of early technologies that have yet to progress through the regulatory process. In addition, there may be additional challenges in some HTA contexts, for example, in the assessment of genomic treatments or treatments for rare diseases. In such settings, a potential reference case should consider the additional issues summarized in the third column of Table 1.

## Conclusions

Elicitation can be a valuable method for HCDM, particularly to inform reimbursement decisions that are supported by model-based economic evaluation. Elicitation provides the additional information needed to reach a decision when empirical evidence is lacking.

This article describes work to generate reference case methods for elicitation for HCDM. We believe that the results will be useful for analysts and decision makers in HCDM. Elicitation conducted in this context to date has not used a common set of methods and, above all, has not consistently considered the implications of the methods choices made when designing and conducting an elicitation. To improve the accountability of HCDM, the procedure used to derive expert judgments should be transparent and documented.

The reference case methods presented here serve as a benchmark for good practice and reporting. Although consistency in the methods applied is desirable to ensure consistent evaluations, the lack of evidence on the performance of different methodological choices means we could not be prescriptive. This reference case is therefore, by virtue of the evidence used to support it, flexible in many choices. This may be a useful characteristic, as it is possible to apply the reference case across different settings within HTA. Deviations from the suggested methods should be justified and limitations discussed in the elicitation documentation. It may be useful to report the methods used in the applied elicitation using the reference case methods as a benchmark.

Here we illustrate the development of a reference case specific to the HTA setting. Different HCDM contexts have different constraints and requirements. Outside of HTA, there are key methodological choices that may involve additional or different considerations, for example, as part of the commissioning process at a local level or for early technologies that have yet to progress through the regulatory process. Moreover, in some circumstances, it may not be possible to conduct face-to-face elicitation. Group discussion may be needed to generate a distribution, where there is no practical experience of the quantity of interest.

The major limitation of this work lies in the evidence available from the wider literature, on which to base methods choices and determine their appropriateness. The lack of an agreed-upon definition for accuracy of elicitation also limits the choice of “best” methods. In many circumstances, expert beliefs are unobservable to the analyst, so that determining how well methods perform in enabling experts to express their beliefs is a complex task.

There are important areas warranting further research. These include strategies to recruit experts, methods for training experts to minimize bias, and methods for eliciting dependent quantities from nonnormative experts. Application of the reference case in further studies, including in settings with a range of constraints, will generate valuable evidence regarding its applicability and value.

## Supplemental Material

sj-docx-1-mdm-10.1177_0272989X211028236 – Supplemental material for Reference Case Methods for Expert Elicitation in Health Care Decision Making Supplemental material, sj-docx-1-mdm-10.1177_0272989X211028236 for Reference Case Methods for Expert Elicitation in Health Care Decision Making by Laura Bojke, Marta O. Soares, Karl Claxton, Abigail Colson, Aimée Fox, Chris Jackson, Dina Jankovic, Alec Morton, Linda D. Sharples and Andrea Taylor in Medical Decision Making

sj-docx-2-mdm-10.1177_0272989X211028236 – Supplemental material for Reference Case Methods for Expert Elicitation in Health Care Decision Making Supplemental material, sj-docx-2-mdm-10.1177_0272989X211028236 for Reference Case Methods for Expert Elicitation in Health Care Decision Making by Laura Bojke, Marta O. Soares, Karl Claxton, Abigail Colson, Aimée Fox, Chris Jackson, Dina Jankovic, Alec Morton, Linda D. Sharples and Andrea Taylor in Medical Decision Making
