# Supplementary material for: Reference Case Methods for Expert Elicitation in Health Care Decision Making
Source: Med Decis Making. 2021 Jul 16;42(2):182–93. doi: 10.1177/0272989X211028236 (PMC8777312; doi:10.1177/0272989X211028236)
Supplement: sj-docx-1-mdm-10.1177_0272989X211028236 – Supplemental material for Reference Case Methods for Expert Elicitation in Health Care Decision Making [file sj-docx-1-mdm-10.1177_0272989X211028236.docx]

**Appendix**

**Search details for the review of existing published guidelines for elicitation**

We used the following inclusion criteria to identify elicitation guidelines:

1. Guidelines must be full-length (i.e., no conference abstracts), English-language documents published from 1990-2018.
2. Guidelines must focus on the structured elicitation of explicitly probabilistic judgements from experts (i.e., no papers primarily about eliciting rankings, paired comparisons, or other non-probabilistic information from experts).
3. Guidelines must offer recommendations for practice concerning more than one of the stages of an elicitation (i.e., design, preparation, conduct, and analysis).

Guidelines are published as scientific papers and as policy documents or other grey literature, so we adopted a broad search strategy designed to capture all relevant guidelines (Figure 1). We searched Scopus and Web of Science for the period from 1990 to 2018 using the following keywords: “(expert AND (judgment OR judgement OR opinion) AND (elicit*) AND (method* OR protocol OR procedure OR guid* OR technique)),” which yielded 827 results in Web of Science and 989 in Scopus. Results were screened based on the title and abstract. If a paper potentially met the inclusion criteria based on its title and abstract, the full-text source was reviewed. The references lists in each of the full-text articles were reviewed to identify additional possible guidelines.

**Extraction template**

For each of the included SEE guidelines, information was gathered on the elicitation process in an extraction template (Table 1). This describes the elicitation process as pertaining to 3 stages: 1) preparation and design, 2) conduct and 3) post-elicitation. The extraction template was based on previous reviews of the elicitation process and was piloted and refined before use in this review.

| Source |  |
| --- | --- |
| Type of article |  |
| Domain |  |
| Self-reported objective |  |
| **Preparation and design** | |
| What quantities to elicit? |  |
| Who/how many experts? |  |
| How to encode judgements? |  |
| How to manage biases? |  |
| How to approach validation? |  |
| Piloting the exercise |  |
| Training and preparation for experts |  |
| Training for other roles |  |
| **Elicitation** | |
| Level of elicitation |  |
| Mode of administration |  |
| Feedback to experts & revision |  |
| Opportunity for interaction |  |
| Feedback from experts on process |  |
| Rationales |  |
| **Aggregation, analysis, and post-elicitation** | |
| If/how to aggregate |  |
| Fit to distribution |  |
| Adjusting judgements |  |
| Documentation |  |

**Search results**


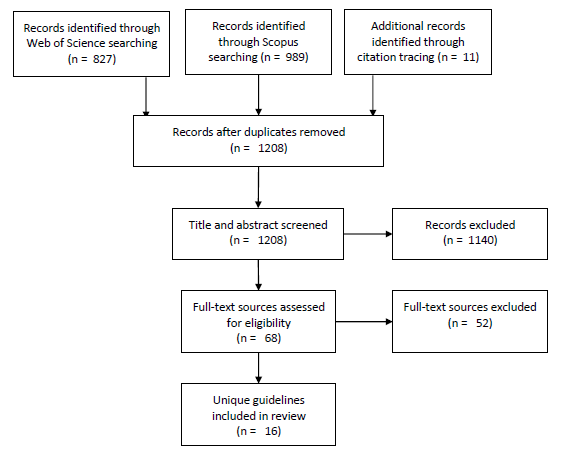


In some cases, multiple sources with the same or similar author lists provide very similar recommendations. For the purposes of this review, these sources are considered to be the same guidance, and only one version was included. The earliest complete version of the guidance that appeared was used, but references to the “duplicate” guidelines are included in the next section.

In the screening process, papers that focused on only one aspect of the elicitation process were excluded, such as how to encode judgements or fit judgements to distributions. Descriptions of software that did not discuss aspects of elicitation not managed within the software, were excluded. Reviews of past elicitation work or where experts can be used within a specific field were excluded, if they did not offer recommendations for practice. Finally, cases studies that focused on the presentation of a set of results rather than a methodology for future work were excluded.

**Guidelines included in the review and related sources identified in the search**

| **Key reference** | **Domain** | **Type of article** |
| --- | --- | --- |
| (Choy, O'Leary et al. 2009) | Ecology | Review of evidence and/or practice |
| Classical Model  (Cooke and Goossens 2000) | Generic | Review of evidence and/or practice; Reflection on personal practice |
| EFSA Delphi  (European Food Safety 2014) | Food safety | Agency guidance |
| (EPA 2009) | Environmental protection | Agency guidance |
| (Garthwaite, Kadane et al. 2005) | Generic | Review of evidence and/or practice |
| IDEA protocol (Hemming, Burgman et al. 2018) | Generic | Review of evidence and/or practice; Reflection on personal practice |
| (Ashcroft, Austin et al. 2016) | Insurance | Agency guidance |
| (Tredger, Lo et al. 2016) | Insurance | Agency guidance |
| (Kaplan 1992) | Risk and reliability | Reflection on personal practice |
| (Keeney and Vonwinterfeldt 1991) | Nuclear | Reflection on personal practice |
| (Knol, Slottje et al. 2010) | Environmental health | Review of evidence and/or practice |
| (Meyer and Booker 2001) | Generic | Review of evidence and/or practice |
| (Kotra, Lee et al. 1996) | Nuclear | Agency guidance |
| (Budnitz, Apostolakis et al. 1997) | Nuclear | Agency guidance |
| SHELF  (Gosling 2018) | Generic | Review of evidence and/or practice; Reflection on personal practice |
| (Walls and Quigley 2001) | Risk and reliability | Reflection on personal practice |

**Choices available from existing guidelines**

| **Element** | **Component** |
| --- | --- |
| **Selecting quantities** | |
| What quantities to elicit | Type of parameter |
|  | Type of quantity |
|  | Selection criteria |
|  | Principles for describing quantities |
|  | Decomposition |
|  | Handling dependence |
| Encoding judgements | General approach |
|  | Use of visual aids |
| **Selecting experts** | |
| Number of experts | Number of experts |
| Selecting experts | Roles within SEE |
|  | Desired characteristics for those provide judgements |
|  | Identification procedure |
|  | Selection procedure |
|  | Possible selection criteria |
| **Training and preparation** | |
| Pilot the protocol | Pilot exercise |
| Training and preparation for experts | What to cover in training |
| **Level and conduct of elicitation** | |
| Mode of administration | Location |
| Level of elicitation | Level of elicitation |
| Feedback and revision | Type of feedback |
|  | What to feed back |
|  | Opportunity for revision |
| Interaction | Opportunity for interaction |
| Rationales | Rationales |
| **Aggregation, analysis & post elicitation** | |
| Aggregation | Aggregation |
|  | Aggregation approach |
| Fit to distribution | Fit |
|  | Distribution |
|  | Fitting method |
| Feedback on process | Feedback from experts on process |
| Adjusting judgements | Methods for adjusting judgements |
| Documentation | What to include |
| **Managing biases** | |
| Managing heuristics and biases | Biases relevant for SEE |
|  | Bias elimination or reduction strategies |
| **Validation** | |
| Validation | Characteristics/measures |

**Level of agreement on recommendations and choices in SEE guidelines**

| Element | Component | Agreement level | Explanation |
| --- | --- | --- | --- |
| **Selecting quantities** | | | |
| What quantities to elicit | Type of parameter | Some disagreement | Guidelines agree that observable quantities are preferred, but disagree on whether directly eliciting model parameters is an acceptable choice. |
|  | Type of quantity | Disagreement | Guidelines offer conflicting recommendations on whether eliciting probabilities (compared with other uncertain quantities) is an acceptable choice. |
|  | Selection criteria | Some agreement | Fewer than five guidelines discuss this, but they agree selection criteria should be defined. |
|  | Principles for describing quantities | Some agreement | Some guidelines describe slightly different principles (e.g., asking clear questions, ensuring uncertainty on elicited parameters impacts the final decision or model), but they do not conflict. |
|  | Decomposition | Agreement | The guidelines that discuss decomposing the variables of interest all agree it should be a choice. |
|  | Handling dependence | Some agreement | The guidelines that discuss dependence agree it should be avoided if possible or addressed separately, but they discuss a range of methods for considering dependence. |
| Encoding judgements | General approach | Disagreement | Guidelines recommend and discuss different, conflicting methods for encoding judgements. |
|  | Use of visual aids | Some agreement | Fewer than five guidelines discuss this, but they agree visual aids can be a useful choice. |
| **Selecting experts** | | | |
| Number of experts | Number of experts | Agreement | The experts agree that multiple experts are important, with most guidelines recommending around 5-10 experts. |
| Selecting experts | Roles within SEE | Agreement | The guidelines are very consistent in their description of the roles involved with elicitation. |
|  | Desired characteristics for those provide judgements | Some agreement | Characteristics discussed in the guidelines are largely consistent, aside from differing views on if normative expertise is a requirement or just desired. |
|  | Identification procedure | Some agreement | Recommendations differ but do not conflict across the guidelines. Agency guidelines tend to offer more detail. |
|  | Selection procedure | Some agreement | Recommendations differ but do not conflict across the guidelines. Agency guidelines tend to offer more detail. |
|  | Possible selection criteria | Some agreement | Recommendations differ but do not conflict across the guidelines. |
| **Training and preparation** | | | |
| Pilot the protocol | Pilot exercise | Agreement | Almost all guidelines recommend conducting a pilot exercise. |
| Training and preparation for experts | What to cover in training | Some agreement | The lists of what should be included in training vary across guidelines but do not conflict. |
| **Level and conduct of the elicitation** | | | |
| Mode of administration | Location | Some agreement | Most guidelines agree that face-to-face administration is preferred, though remote options may be pragmatically useful alternative in some situations. |
| Level of elicitation | Level of elicitation | Disagreement | Guidelines recommend and discuss conflicting levels of elicitation. |
| Feedback and revision | Type of feedback | Some agreement | Recommendations differ but do not conflict across the guidelines. |
|  | What to feed back | Some agreement | Recommendations differ but do not conflict across the guidelines. |
|  | Opportunity for revision | Some agreement | Guidelines either recommend revision take place following an elicitation (as part of an iterative process or immediately following the elicitation) or further in the future, following a draft report or additional data collection. |
| Interaction | Opportunity for interaction | Disagreement | Guidelines offer conflicting recommendations about when and how to facilitation interaction between the experts. |
| Rationales | Rationales | Agreement | Almost all guidelines recommend collecting expert rationales in some form. |
| Aggregation, analysis and post-elicitation | | | |
| Aggregation | Aggregation | Agreement | All guidelines discuss aggregation as a recommendation or valid choice. |
|  | Aggregation approach | Disagreement | Guidelines offer conflicting recommendations on the approach and method to aggregate judgements. |
| Fit to distribution | Fit | Some disagreement | The guidelines make few recommendations, but their choices differ. |
|  | Distribution | Some agreement | Fewer than five guidelines discuss this, but they generally agree that many parametric distributions could be chosen. |
|  | Fitting method | Some agreement | Fewer than five guidelines discuss this, but they generally agree that choices include minimum least squares and method of moments. |
| Feedback on process | Feedback from experts on process | Some agreement | Fewer than five guidelines discuss this, and they recommend complementary approaches. |
| Adjusting judgements | Methods for adjusting judgements | Some disagreement | Fewer than five guidelines discuss this, but they offer different perspectives. |
| Documentation | What to include | Some agreement | The lists of what should be included in final documentation vary across guidelines but do not conflict. |
| Managing biases | | | |
| Managing heuristics and biases | Biases relevant for SEE | Some agreement | The lists of potential biases vary across guidelines but do not conflict. |
|  | Bias elimination or reduction strategies | Some agreement | The list of possible strategies vary across guidelines but do not conflict. |
| Validation | | | |
| Validation | Characteristics/measures | Disagreement | The guidelines differ in their definitions of validity and discussion of how the concept can be operationalised in an elicitation. |

**Summary of principles applied to choices for SEE in HCDM**

|  | **Element** | **Key messages from critique** | **Principles support** | **Principles do not support** |
| --- | --- | --- | --- | --- |
| **Preparation and design** | Selecting quantities | Different quantities can be elicited that provide information on any single parameter of interest.  Also relevant is handling dependence, selection criteria, principles for describing quantities and decomposition/disaggregation.  Lack of evidence on how that choice should be guided.  The choice is largely driven by the practical constraints of the context. | Types of quantities   - - Observables such as probabilities (expressed as proportions or frequencies) | - Measures of central tendency in isolation - Odds ratios - Credible ranges |
|  |  |  | Dependency   - Ask only about independent variables - Express dependent variables in terms of independent variables - Use separate dependence elicitation methods |  |
|  |  |  | Choice of parameters   - Definition of a selection criteria, such as minimal assessment of each possible uncertain parameter and sensitivity analysis to see which uncertain parameters have the biggest impact |  |
|  |  |  | Wording   - Avoid vagueness - Ask questions in a manner consistent with how experts express their knowledge - Use neutral wording - Do not use leading questions |  |
|  |  |  | Decomposition | No decomposition |
|  | Methods to encode judgements | The FIM, the roulette or chips and bins method has previously been used in HCDM. The VIM, has also had limited use in HCDM, utilising quantiles as opposed to the bisection method.  There is no empirical evidence to support which of these two methods is most appropriate in HCDM. | - Fixed interval methods – all forms - Variable interval methods – all forms |  |
|  | Managing biases | No studies have explicitly examined the effectiveness of debiasing techniques in this context. The appropriateness of many suggested methods for debiasing are uncertain. | - Give experts practice and feedback - Provide training on biases - Frame questions to minimize bias and ambiguity - Identify biases through discussion with expert - Provide relevant background evidence - Ask for upper/lower bounds first - Ask experts to specify the credible interval they have provided - Minimize and record conflicts of interest among the experts - Require the experts address conflicting information - Collect rationales from experts - Report anonymous results - Include external experts - Anticipate likely biases |  |
|  | Validation | There is uncertainty about which method to validate is more appropriate in this context.  Methods to reduce variability may not be appropriate, in the interests of reflecting any between expert variation. | - Faithfully capturing experts’ beliefs - Fitness for purpose - Internal review - External review - Coherence - Consistency | - Calibration - Calibration & informativeness scoring |
|  | Selecting experts | Lack of evidence in HCDM to make definitive statement about particular approaches.  Is a need to include all three types of roles for ‘experts’: the facilitator, expert providing priors and generalists to advise on design etc.  Desired characteristics for those providing judgements are above all a level of substantive experience and a willingness to participate. Other characteristics may be beneficial, in particular normative expertise but may be difficult to ensure in HCDM.  Training and careful design can mitigate against the need for some of these.  Identifying relevant experts in HCDM is more likely to be driven by practical constraints, however ensuring a generalizable and wide sample is preferred. | Roles in SEE   - Facilitator - Expert | - Generalists |
|  |  |  | Desired characteristics   - Substantive expertise - Willingness to participate | - Normative expertise - Ability to understand questions - Ability to apply skills |
|  |  |  | Identification   - Recommendations by peers, either formally or informally - Research outputs - Known experience - RFP to seek out experts - Experience - Profile matrix |  |
|  |  |  | Selection   - Disclosure of personal and financial interests - Pursue diversity | - Formal selection criteria developed and applied - Review CVs - Profile matrix |
|  |  |  | Criteria   - Reputation - Experimental experience - Publication history - Diversity in background - Conflicts of interest - Awards - Balancing different viewpoints - Peer assessment (such as GEM) | - Balance of internal and external experts (e.g., include at least 2 external experts) |
|  |  |  | Number  No definitive guidance on number but seems to suggest at least 5-9 experts |  |
|  | Pilot exercise | The ability to conduct a pilot may be driven by the constraints in HCDM. | Use of piloting | No piloting |
|  | Training and preparation for experts | Training is essential for non-normative experts, although there is uncertainty about what should be contained within the training.  Details about how elicited distributions will be used may not be possible to feedback back to time constraints in HCDM. | - Probability, including subjective probability - Motivation for elicitation - Description of what is required from experts - Outline of process - Outline of questions - Example and practice questions - Review of potential biases - Motivation of elicitation | - Description of performance assessment - Introduction to dependence - List of relevant information - How results will be used - The full protocol |
| **Elicitation** | Level of elicitation | Discussion may not always be feasible due to the constraints of HCDM. Instead it may be possible to do this remotely via a Delphi, specifically after individual distributions have been elicited.  Group interaction may introduce biases, such as overconfidence. | - Individual - Combination | - Consensus |
|  | Mode of administration | The mode of administration may be driven by the constraints of HCDM. | - Face-to-face - Remote |  |
|  | Feedback to experts and revision | It is uncertain which types of information should be presented and at which stage of the SEE.  The information relayed to experts may be driven by their level of normative skills. | Type of feedback   - Graphical feedback - Distributions from other experts - Summaries of aggregated distributions - Rationales - Qualitative discussion of elicited values - Written description of experts rationales | - Fitted distribution - Performance scores - Results using elicited values - Future data - The draft elicitation report - Decision resulting from the expert judgement |
|  |  |  | Opportunities for revision   - A set number of elicitation/feedback rounds from the outset - Update after future data is collected - Update for revisions/clarifications after circulating draft elicitation report - Individuals update during or after a session based on graphics or other information on fitted distribution |  |
|  | Opportunity for interaction | There is little practical experience in HCDM with different methods of interaction between experts. | - No interaction - Group discussion prior to individual elicitation - Group discussion and group elicitation - Group discussion following individual elicitation (with opportunity for revision) - Remote, anonymized interaction |  |
|  | Feedback from experts on process | Feedback is often undertaken in SEE for HCDM, although there are not consistent approaches to do this. The practicalities of conducting SEE in HCDM, may dictate if the method of feedback is practically plausible. |  | - Ask experts to appraise the elicitation exercise after completing it. - Get feedback on the procedure if future data collection contradicts elicitation results |
|  | Rationales | There is no practical experience in HCDM with different methods of providing rationales. | Collect/record rationales from experts (about how they made their judgments) | No mention of rationales |
| **Aggregation, analysis and post-elicitation** | If/how to aggregate | Behavioural methods of aggregation may be practically difficult in the context of HCDM, both in terms of convening experts but also in terms of the provision of experienced facilitators. | - Mathematical using linear opinion pooling - Combination | Behavioural |

|  | Fit to distribution | In HCDM, fitting of a smooth distribution would seem appropriate.  The choice of parametric distribution is uncertain. There is a lack of evidence in HCDM on the fitting process in SEE. Limited evidence suggests that standard distributions, such as the Beta will often be sufficient.  More complex approaches may be appropriate, however these can be complex to implement in general software. | Fitting | Not fitting |
| --- | --- | --- | --- | --- |
|  |  |  | Distributional form   - Normal - Beta - Other conjugate family | - Uniform - Triangular - Uniform over elicited intervals |
|  |  |  | Selection criteria   - Minimum least squares - Method of moments - Other approaches |  |
|  | Adjusting judgements | There is a lack of practical experience in HCDM to inform the choice of adjustment method. Given the role of facilitator in SEE in HCDM it would seem inappropriate for the facilitator to adjust themselves using arbitrary criteria. | Adjust/not adjust |  |
|  |  |  | - Calibrate - Adjust to improve coherence | Analyst adjustment and feedback |
|  | Documentation | In order to inform a decision making process in HCDM, a SEE should document all details, including elicitation questions, the responses, fitting process, level of elicitation, interaction, revision and validation | Thorough documentation | Less detailed/no documentation |
