# Supplementary material for: Reference Case Methods for Expert Elicitation in Health Care Decision Making
Source: Med Decis Making. 2021 Jul 16;42(2):182–93. doi: 10.1177/0272989X211028236 (PMC8777312; doi:10.1177/0272989X211028236)
Supplement: sj-docx-2-mdm-10.1177_0272989X211028236 – Supplemental material for Reference Case Methods for Expert Elicitation in Health Care Decision Making [file sj-docx-2-mdm-10.1177_0272989X211028236.docx]

**Title:** Developing a reference protocol for expert elicitation in healthcare decision making

**Authors:** Laura Bojke,^1^ Marta Soares,^1^ Karl Claxton,^1^ Abigail Colson,^2^ Aimée Fox,^1^ Chris Jackson,^3^ Dina Jankovic,^1^ Alec Morton,^2^ Linda Sharples,^4^ Andrea Taylor^5^

**Affiliations:**

^1^ Centre for Health Economics, University of York, York, UK

^2^ The Department of Management Science, University of Strathclyde, Glasgow, UK

^3^ MRC Biostatistics Unit, University of Cambridge, Cambridge, UK

^4^ London School of Hygiene and Tropical Medicine, London, UK

^5^ Leeds University Business School, Leeds, UK

**Corresponding Author:**

Laura Bojke,

Centre for Health Economics, University of York, Heslington, York, YO10 5DD, UK

[Laura.bojke@york.ac.uk](mailto:Laura.bojke@york.ac.uk)

00441904321416

**Conflict of Interest:**

Competing interests: AM declares private consulting for AstraZeneca and Office of Health Economics, not related to the current work. AM and AC were previously supported by a European Union FP7 project which received in-kind support from pharmaceutical companies, including Astellas, Roche and AstraZeneca. LB declares private consulting for Bresmed and Roboleo, not related to the current work.

**Key words:**

Expert-elicitation, health, decision-making, transparency, uncertainty.

**Abstract**

*Background*

Many decisions in healthcare aim to maximise health, requiring judgements about interventions that may have higher health effects but potentially incur additional costs (cost-effectiveness framework). The evidence used to establish cost-effectiveness is typically uncertain and it is important that this uncertainty is characterised.

In situations where evidence is uncertain, the experience of experts is essential. The process by which the beliefs of experts can be formally collected in a quantitative manner is structured expert elicitation (SEE). There is heterogeneity in the existing methodology used in healthcare decision making (HCDM). A number of guidelines are available for SEE. It is not clear if any of these are appropriate for HCDM.

*Objectives*

The overall aim was to establish a protocol for SEE to inform HCDM. The objectives are:

- Provide clarity on methods for collecting and using experts judgements.
- Consider where alternative methodology may be required in particular contexts.
- Establish preferred approaches for elicitation on a range of parameters.
- Determine which elicitation methods allow experts to express uncertainty.
- Determine the usefulness of the reference protocol developed.

*Methods*

A mixed methods approach was used: systemic review, targeted searches, experimental work and narrative synthesis. A review of existing guidelines for SEE was conducted. This identified the approaches used in existing guidelines (the “choices”) and determined if dominant approaches exist. Targeted review searches where conducted for: selection of experts, level of elicitation, fitting and aggregation, assessing accuracy of judgements and heuristics and biases.

To sift through the available choices, a set of principles that underpin the use of SEE in HCDM was defined using evidence generated from the targeted searches, quantities to elicit, experimental evidence and consideration of constraints in HCDM. These principles, including fitness for purpose and reflecting individual expert uncertainty, where applied to the set of choices to establish a reference protocol. An applied evaluation of the developed reference protocol was also undertaken.

*Results*

For many elements of SEE, there was a lack of consistency across the existing guidelines.

In almost all choices, there was a lack of empirical evidence supporting recommendations and in some circumstances the principles are unable to provide sufficient justification for discounting particular choices. It is possible to define reference methods for Health Technology Assessment (HTA). These include: a focus on gathering experts with substantive skills, eliciting observable quantities and individual elicitation of beliefs. Additional considerations are required for decision makers outside of HTA, for example at a local level, or for early technologies. Access to experts may be limited and in some circumstances group discussion may be needed to generate a distribution.

*Limitations*

The major limitation of the work conducted here, lies not in the methods employed in the current work, but the evidence available from the wider literature relating to how appropriate particular methodological choices are.

*Conclusions*

The reference protocol is flexible in many choices. This may be a useful characteristic, as it is possible to apply this reference protocol across different settings. Further applied studies, which use the choices specified in this reference protocol, are required.

*(word count: 500)*

# Table of Contents

[Table of Contents	iii](#_Toc15566561)

[List of Tables	iii](#_Toc15566562)

[List of Figures	iii](#_Toc15566563)

[Glossary	iii](#_Toc15566564)

[Abbreviations	v](#_Toc15566565)

[Plain English Summary	vii](#_Toc15566566)

[Scientific Summary	viii](#_Toc15566567)

[Chapter 1 Background	1](#_Toc15566568)

[Chapter 2 Good practice in SEE: Learning from the available guidance	8](#_Toc15566569)

[Section 2.1 Introduction	8](#_Toc15566570)

[Section 2.2 Methods	8](#_Toc15566571)

[Section 2.3 Included SEE guidelines	9](#_Toc15566572)

[Section 2.4 Analysis of the elicitation process	9](#_Toc15566573)

[Section 2.5 Identifying elicitation variables	23](#_Toc15566574)

[Section 2.5.1 What quantities to elicit	23](#_Toc15566575)

[Section 2.5.2 Encoding judgements	24](#_Toc15566576)

[Section 2.6 Identifying and selecting experts	25](#_Toc15566577)

[Section 2.7 Training and preparation	26](#_Toc15566578)

[Section 2.8 Conducting the elicitation	26](#_Toc15566579)

[Section 2.8.1 Mode and level of elicitation	26](#_Toc15566580)

[Section 2.8.2 Feedback and revision	27](#_Toc15566581)

[Section 2.8.3 Interaction	27](#_Toc15566582)

[Section 2.8.4 Rationales	28](#_Toc15566583)

[Section 2.9 Post-elicitation	28](#_Toc15566584)

[Section 2.9.1 Aggregation	28](#_Toc15566585)

[Section 2.9.2 Fit to distribution	30](#_Toc15566586)

[Section 2.9.3 Other post-elicitation components	30](#_Toc15566587)

[Section 2.10 Managing heuristics and biases	31](#_Toc15566588)

[Section 2.11 Considering the validity of the process and results	31](#_Toc15566589)

[Section 2.12 Conclusions	32](#_Toc15566590)

[Chapter 3 Expert elicitation in different decision making contexts	33](#_Toc15566591)

[Section 3.1 Introduction	33](#_Toc15566592)

[Section 3.2 Levels of decision making	33](#_Toc15566593)

[Section 3.2.1 Individual practitioners and local ‘population level’ decision makers	34](#_Toc15566594)

[Section 3.2.2 National decision makers	36](#_Toc15566595)

[Section 3.2.3 Research commissioners	39](#_Toc15566596)

[Section 3.3 Conclusions	40](#_Toc15566597)

[Chapter 4 Challenges in structured elicitation in HCDM	42](#_Toc15566598)

[Section 4.1 Introduction	42](#_Toc15566599)

[Section 4.2 Methods	42](#_Toc15566600)

[Section 4.3 Aspects related to the design of the SEE	42](#_Toc15566601)

[Section 4.4 Experiences with the conduct of the exercise	44](#_Toc15566602)

[Section 4.5 Experiences with the analyses and interpretation of elicited evidence	44](#_Toc15566603)

[Section 4.6 Conclusions	45](#_Toc15566604)

[Section 4.7 Discussion	45](#_Toc15566605)

[Chapter 5 Reviewing the evidence: expert selection, level of elicitation, fitting and pooling	47](#_Toc15566606)

[Section 5.1 Introduction	47](#_Toc15566607)

[Section 5.2 Identifying literature	47](#_Toc15566608)

[Section 5.3 Selection of experts	48](#_Toc15566609)

[Section 5.4 Level of elicitation (individual versus group)	50](#_Toc15566610)

[Section 5.5 Fitting & pooling	54](#_Toc15566611)

[Section 5.5.1 Distribution choice and fitting	54](#_Toc15566612)

[Section 5.5.2 Mathematical aggregation	56](#_Toc15566613)

[Section 5.6 Assessing the expected accuracy of experts judgements	57](#_Toc15566614)

[Section 5.7 Conclusion	59](#_Toc15566615)

[Chapter 6 Reviewing the evidence: Heuristics and biases	62](#_Toc15566616)

[Section 6.1 Introduction	62](#_Toc15566617)

[Section 6.2 Cognitive and motivational biases	63](#_Toc15566618)

[Section 6.2.1 Cognitive biases	63](#_Toc15566619)

[Section 6.2.2 Motivational biases	64](#_Toc15566620)

[Section 6.2.3 Overconfidence bias	64](#_Toc15566621)

[Section 6.3 Addressing psychological biases in in SEE	64](#_Toc15566622)

[Section 6.4 Technical bias reduction strategies	65](#_Toc15566623)

[Section 6.5 Behavioural bias reduction strategies with consistent support	65](#_Toc15566624)

[Section 6.5.1 Consider more information	65](#_Toc15566625)

[Section 6.5.2 Feedback	66](#_Toc15566626)

[Section 6.5.3 Avoid unnecessary anchors	67](#_Toc15566627)

[Section 6.5.4 Reduce bias through expert selection	67](#_Toc15566628)

[Section 6.6 Behavioural bias reduction techniques with conflicting evidence	68](#_Toc15566629)

[Section 6.6.1 Bias warnings and training	68](#_Toc15566630)

[Section 6.6.2 Fixed value versus fixed probability methods	68](#_Toc15566631)

[Section 6.6.3 Face-to-face versus online elicitation	69](#_Toc15566632)

[Section 6.7 Conclusions	69](#_Toc15566633)

[Chapter 7 Quantities to elicit	70](#_Toc15566634)

[Section 7.1 Introduction	70](#_Toc15566635)

[Section 7.2 Overview of probability-, rate- and hazard-type parameters	71](#_Toc15566636)

[Section 7.2.1 Simple probability and conditional probability parameters	71](#_Toc15566637)

[Section 7.2.2 Transition probability parameters in discrete-time STMs	71](#_Toc15566638)

[Section 7.2.3 Time to event and survival	72](#_Toc15566639)

[Section 7.2.4 Continuous time-to-event decision models	73](#_Toc15566640)

[Section 7.2.5 Repeated event rates	74](#_Toc15566641)

[Section 7.3 Eliciting probability, rate and hazard-related parameters	74](#_Toc15566642)

[Section 7.4 Current practices in elicitation	75](#_Toc15566643)

[Section 7.4.1 Identification of examples	75](#_Toc15566644)

[Section 7.4.2 Simple and conditional probability or odds	76](#_Toc15566645)

[Section 7.4.3 Transition probability parameters in discrete-time STMs	77](#_Toc15566646)

[Section 7.4.4 Time to event and survival	78](#_Toc15566647)

[Section 7.4.5 Treatment effects on time-to-event distributions	79](#_Toc15566648)

[Section 7.4.6 Elicitation of hazards or of parameters of a time to event distribution directly	81](#_Toc15566649)

[Section 7.5 Steps and considerations in defining the quantities to elicit	81](#_Toc15566650)

[Section 7.6 Discussion	83](#_Toc15566651)

[Chapter 8 Three methodological experiments on the elicitation of subjective probabilistic belief	85](#_Toc15566652)

[Section 8.1 Introduction	85](#_Toc15566653)

[Section 8.2 General approach to the experiments	86](#_Toc15566654)

[Section 8.3 Methods	87](#_Toc15566655)

[Section 8.3.1 Overview of the experimental approach	87](#_Toc15566656)

[Section 8.3.2 Experiment 1: comparing different methods of elicitation	89](#_Toc15566657)

[Section 8.3.3 Experiment 2: Are individuals’ able to ‘extrapolate’ from their knowledge-base?	90](#_Toc15566658)

[Section 8.3.4 Experiments 3: To understand how individuals review their own probabilistic assessments when presented with Delphi-type summaries.	92](#_Toc15566659)

[Section 8.4 Methods of analyses	94](#_Toc15566660)

[Section 8.4.1 Outcomes and metrics used	94](#_Toc15566661)

[Section 8.4.2 Methods	96](#_Toc15566662)

[Section 8.5 Results	97](#_Toc15566663)

[Section 8.5.1 Description of the sample recruited	97](#_Toc15566664)

[Section 8.5.2 Experiment 1	98](#_Toc15566665)

[Section 8.5.3 Experiment 2	101](#_Toc15566666)

[Section 8.5.4 Experiment 3.1	102](#_Toc15566667)

[Section 8.5.5 Experiment 3.2	105](#_Toc15566668)

[Section 8.6 Conclusions	107](#_Toc15566669)

[Section 8.6.1 Key findings from the experiments	107](#_Toc15566670)

[Section 8.6.2 Limitations and suggestions for future research (using the same experimental approach)	109](#_Toc15566671)

[Section 8.6.3 Experimental approach vs. using almanac quantities	110](#_Toc15566672)

[Chapter 9 Consideration of the methodological choices emerging from existing guidelines	111](#_Toc15566673)

[Section 9.1 Introduction	111](#_Toc15566674)

[Section 9.2 Principles underpinning the use of SEE to inform HCDM	111](#_Toc15566675)

[Section 9.2.1 Principle 1. Transparency	112](#_Toc15566676)

[Section 9.2.2 Principle 2. Fitness for purpose	113](#_Toc15566677)

[Section 9.2.3 Principle 3. Consistency, but respecting constraints of the decision making context	113](#_Toc15566678)

[Section 9.2.4 Principle 4. Reflecting uncertainty at the individual expert level	113](#_Toc15566679)

[Section 9.2.5 Principle 5. Recognising and acting on biases	113](#_Toc15566680)

[Section 9.2.6 Principle 6. Suitability for experts who possess substantive skills, who are less likely to be normative	114](#_Toc15566681)

[Section 9.2.7 Principle 7. Recognising where adaptive skills are required	114](#_Toc15566682)

[Section 9.2.8 Principle 8. Recognising and act on between-expert variation	114](#_Toc15566683)

[Section 9.2.9 Principle 9. Promoting high performance	115](#_Toc15566684)

[Section 9.3 How do SEE elements and methodological choices reflect the principles underpinning healthcare?	115](#_Toc15566685)

[Section 9.3.1 Selecting quantities (preparation and design)	115](#_Toc15566686)

[Section 9.3.2 Methods to encode judgements (preparation and design)	117](#_Toc15566687)

[Section 9.3.3 Selecting experts	118](#_Toc15566688)

[Section 9.3.4 Pilot exercise	119](#_Toc15566689)

[Section 9.3.5 Training and preparation for experts	119](#_Toc15566690)

[Section 9.3.6 Level of elicitation (elicitation)	120](#_Toc15566691)

[Section 9.3.7 Mode of Administration (elicitation)	121](#_Toc15566692)

[Section 9.3.8 Feedback to experts and revision (elicitation)	121](#_Toc15566693)

[Section 9.3.9 Opportunity for interaction (elicitation)	122](#_Toc15566694)

[Section 9.3.10 Feedback from experts on process (elicitation)	123](#_Toc15566695)

[Section 9.3.11 Rationales (elicitation)	123](#_Toc15566696)

[Section 9.3.12 If/how to aggregate (aggregation, analysis and post-elicitation)	124](#_Toc15566697)

[Section 9.3.13 Fit to distribution (aggregation, analysis and post-elicitation)	125](#_Toc15566698)

[Section 9.3.14 Adjusting judgements (aggregation, analysis and post-elicitation)	126](#_Toc15566699)

[Section 9.3.15 Documentation (aggregation, analysis and post-elicitation)	126](#_Toc15566700)

[Section 9.3.16 Managing biases	127](#_Toc15566701)

[Section 9.3.17 Validation	127](#_Toc15566702)

[Section 9.4 Conclusions	128](#_Toc15566703)

[Chapter 10 Reference protocol for expert elicitation in healthcare	130](#_Toc15566704)

[Section 10.1 Evidence in support of a reference protocol for HCDM	130](#_Toc15566705)

[Section 10.2 How the evidence is used to generate a reference protocol for SEE in HCDM	131](#_Toc15566706)

[Section 10.3 Reference protocol for SEE in HTA	131](#_Toc15566707)

[Section 10.4 Important considerations for decision makers outside of the HTA setting	133](#_Toc15566708)

[Section 10.5 Conclusions	134](#_Toc15566709)

[Chapter 11 Applied evaluation of developed reference protocol	136](#_Toc15566710)

[Section 11.1 Background	136](#_Toc15566711)

[Section 11.2 The evaluation topic	136](#_Toc15566712)

[Section 11.2.1 Diagnosis of asthma	137](#_Toc15566713)

[Section 11.2.2 Diagnostic model developed	137](#_Toc15566714)

[Section 11.3 Description of elicited parameters	138](#_Toc15566715)

[Section 11.3.1 Time until correct diagnosis	138](#_Toc15566716)

[Section 11.4 Application of developed reference protocol	139](#_Toc15566717)

[Section 11.4.1 Selecting the quantities (preparation and design stage)	140](#_Toc15566718)

[Section 11.4.2 Methods to encode judgements (preparation and design stage)	141](#_Toc15566719)

[Section 11.4.3 Validation (preparation and design stage)	141](#_Toc15566720)

[Section 11.4.4 Selecting experts (preparation and design stage)	141](#_Toc15566721)

[Section 11.4.5 Pilot exercise (preparation and design stage)	142](#_Toc15566722)

[Section 11.4.6 Training and preparation for experts (preparation and design stage)	142](#_Toc15566723)

[Section 11.4.7 Level of elicitation (elicitation stage)	142](#_Toc15566724)

[Section 11.4.8 Mode of administration (elicitation stage)	142](#_Toc15566725)

[Section 11.4.9 Feedback to experts and revision (elicitation stage)	143](#_Toc15566726)

[Section 11.4.10 Opportunity for interaction (elicitation stage)	143](#_Toc15566727)

[Section 11.4.11 Feedback from experts on process (elicitation stage)	143](#_Toc15566728)

[Section 11.4.12 If/how to aggregate (aggregation, analysis and post-elicitation)	144](#_Toc15566729)

[Section 11.4.13 Fit to distribution (aggregation, analysis and post-elicitation)	144](#_Toc15566730)

[Section 11.4.14 Data Protection and Anonymity (aggregation, analysis and post-elicitation)	144](#_Toc15566731)

[Section 11.5 Results	144](#_Toc15566732)

[Section 11.5.1 Elicitation Results	146](#_Toc15566733)

[Section 11.6 Feedback from experts on the process (including rationales and validation)	146](#_Toc15566734)

[Section 11.6.1 Observability of the quantity asked	146](#_Toc15566735)

[Section 11.6.2 Completion of exercise	146](#_Toc15566736)

[Section 11.6.3 Wording of the question	147](#_Toc15566737)

[Section 11.6.4 Value of interaction	147](#_Toc15566738)

[Section 11.6.5 Value of facilitator	147](#_Toc15566739)

[Section 11.6.6 Experts rationales	147](#_Toc15566740)

[Section 11.6.7 General feedback from experts	148](#_Toc15566741)

[Section 11.6.8 Practicality of conducting the SEE process	148](#_Toc15566742)

[Section 11.7 Conclusion	148](#_Toc15566743)

[Chapter 12 Discussion & conclusions	150](#_Toc15566744)

[Section 12.1 Conclusions on evidence generated	150](#_Toc15566745)

[Section 12.2 Key considerations for using the reference protocol in HCDM	152](#_Toc15566746)

[Section 12.3 Key areas for further research	154](#_Toc15566747)

[Section 12.4 Limitations of the work conducted	156](#_Toc15566748)

[Chapter 13 Acknowledgements	158](#_Toc15566749)

[Chapter 14 Contributions of authors	159](#_Toc15566750)

[Chapter 15 Patient and public involvement	160](#_Toc15566751)

[Chapter 16 References	161](#_Toc15566752)

# List of Tables

[Table 1 Summary of the elicitation elements, components, and choices described in SEE guidelines	10](#_Toc15376011)

[Table 2 Level of agreement on recommendations and choices in SEE guidelines	18](#_Toc15376012)

[Table 3: Summaries of selected survival distributions	73](#_Toc15376013)

[Table 4: List of published examples of eliciting probability, rate and time to-event parameters	76](#_Toc15376014)

[Table 5 : Wording of questions in experiment 2	91](#_Toc15376015)

[Table 6 Sample characteristics	97](#_Toc15376016)

[Table 6 Response to question 1 about the ease of completion.	100](#_Toc15376017)

[Table 7 Response to question 2 about method preference.	100](#_Toc15376018)

[Table 8 Proportion of participants who revised their priors.	103](#_Toc15376019)

[Table 9 Accuracy of initial priors compared between participants who did and did not revise their priors: mean (SD) [median and (interquartile range)] of accuracy metric over participants	103](#_Toc15376020)

[Table 10 Proportion of participants who revised their priors.	105](#_Toc15376021)

[Table 11 Results of experiment 3.2: Outcomes in participants who did and did not revise their priors.	106](#_Toc15376022)

[Table 12 Key principles of SEE in HCDM	111](#_Toc15376023)

[Table 13 A reference case for HTA	132](#_Toc15376024)

[Table 14 Additional issues in generating a reference case outside of HTA	133](#_Toc15376025)

[Table 15 Application of reference case: Parameters elicited in DAR^196^	138](#_Toc15376026)

[Table 16 Application of reference case: Summary of experts recruited	145](#_Toc15376027)

[Table 17 Application of reference case: Elicitation Results	145](#_Toc15376028)

[Table 18 Areas for further research on SEE in HCDM	154](#_Toc15376029)

# List of Figures

[Figure 1 General schematic for SEE	4](#_Toc15376030)

[Figure 2 Summary of project activities	5](#_Toc15376031)

[Figure 3 The elicitation process	10](#_Toc15376032)

[Figure 4 Levels of expert elicitation*	51](#_Toc15376033)

[Figure 5 Elicited points on a cumulative distribution function (CDF) and alternative fitted distributions	55](#_Toc15376034)

[Figure 6 Example of a transition diagram for a state-transition model (STM)	72](#_Toc15376035)

[Figure 7 Example of eliciting conditional probabilities for a decision tree	77](#_Toc15376036)

[Figure 8 Example of eliciting transition probabilities for a novel setting in a discrete-time STM	78](#_Toc15376037)

[Figure 9 Snapshot of the R shiny app (1)	87](#_Toc15376038)

[Figure 10 Snapshot of the R shiny app (2)	87](#_Toc15376039)

[Figure 11 Snapshot of the R shiny app (4)	91](#_Toc15376040)

[Figure 12 Snapshot of the R shiny app (5)	91](#_Toc15376041)

[Figure 13 Illustrative example of the scenarios evaluated in experiment 3.1	93](#_Toc15376042)

[Figure 14 Illustration of the scenarios defined for experiment 3.2	94](#_Toc15376043)

[Figure 15 Distribution of bias, lnSDR and lnKL. (SDR= SD elicited / SD true)	98](#_Toc15376044)

[Figure 16 Within-participant comparison of accuracy when different elicitation methods were used, for each precision scenario.	100](#_Toc15376045)

[Figure 17 Within-participant comparison of accuracy with and without extrapolation, for different levels of extrapolation.	102](#_Toc15376046)

[Figure 18 Within-participant comparison of accuracy to initial priors and extent of revision, for different types of group summaries.	104](#_Toc15376047)

[Figure 19 Within-participant comparison of accuracy and extent of revision (using selected outcome metrics), for different types of group summaries.	106](#_Toc15376048)

**List of supplementary material**

Supplementary material 1: Review of SEE guidelines

Supplementary material 2: Design of the experiments

Supplementary material 3: Analysis of the experiments

Supplementary material 4: Applying choices to the principles for HCDM

Supplementary material 5: The evaluation of the protocol

# Glossary

**Adaptive expertise**

The expert’s ability to adapt their knowledge to new situations for which they do not have prior experience, such as entirely new medical interventions or patients with different characteristics.

**Aleatory uncertainty**

Uncertainty due to randomness. Inherently irreducible and unpredictable in nature.

**Behavioural aggregation**

The process of grouping together individual experts to generate an overall, **consensus** aggregate distribution

**Beta-binomial**

The binomial distribution in which the probability of success at each trial is fixed but randomly drawn from a beta distribution.

**Biases**

Systematic errors in the processes that people use to make judgements. The biases most commonly referred to in SEE are cognitive and motivational biases.

**Bisection method**

A method of elicitation that can be used for any type of continuous univariate distribution. The expert is asked to divide the interval into two equally likely intervals, for example the interval containing the minimum and the median.

**Calibration**

A process to determine the accuracy of a probabilistic judgement. Estimates of expected calibration can be used to weight expert judgements.

**Central tendency**

A measure of the “centre” or typical value for a probability distribution. Examples are the mean, median and the mode.

**Chips and bins**

A graphical representation of the **fixed interval method** for elicitation Experts are asked to give an interval for an uncertain quantity and then place ‘chips’ in ‘bins’ which divide this interval. The chips represent the weight of their belief.

**Choice(s)**

See Elements

**Consensus**

The act of reaching agreement. In SEE this refers to agreement between experts on a distribution.

**Credible range**

A summary of a probability distribution for an uncertain quantity, describing an interval within which the quantity falls with a particular probability.

**Decomposition**

See **disaggregation**

**Delphi method**

A structured communication technique based on several rounds of questionnaires, feedback and revision. The modified Delphi (EFSA) is a form of Delphi method used to elicit an uncertain quantity.

**Dependence**

A statistical relationship between two random variables.

**Domain-specific**

Relating to a particular discipline, e.g. health, education, engineering, or subdivision, e.g. oncology, geriatrics.

**Disaggregation**

Dividing into constituent parts. In SEEE this refers to **decomposition** of a complex quantity into less complex, **observable quantities.**

**Element(s)**

The SEE process comprises numerous elements which encompass several possible components for which choices need to be made. The selection of experts is an example of a SEE element for which the analyst needs to make choices regarding the different components such as how many expert to include, how to recruit the experts and the type of expertise the expert should possess and so on.

**Epistemic uncertainty**

Uncertainty which arises primarily from limited or imperfect knowledge. It is, in principle, reducible by obtaining more or better information.

**Expert**

The individual (s) from whom subjective beliefs are sought. Experts may be defined as such on the basis of their **substantive, normative or adaptive expertise.**

**Facilitator**

An unbiased, impartial individual that works with experts to obtain their **subjective beliefs.** This may involve coordinating active discussion between experts to achieve a consensus, or less hands-on guidance to enable individuals to provide their own judgements.

**Fit for purpose**

Here ‘fir for purpose’ relates to the suitability of a technique or results for its designated role or purpose. In the context of HCDM this will often involve future statistical analysis or modelling.

**Fixed interval method**

A method of elicitation in which experts are presented with an interval and asked to assess the probability that the quantity will fall into that interval.

**Frequencies**

The observed number of successes or failures out of a finite number of trials.

**Hazard**

The probability of transition in a short time interval divided by the length of the interval, in the limit, as this length becomes shorter.

**Kullback-Liebler**

A measure of the difference between two distributions. In the context of elicitation, this is a measure of the information lost when the true distribution is approximated by the elicited distribution^13^.

**Level of elicitation**

Describes either an elicitation conducted at the individual level (which may be followed by **mathematical aggregation)** or at the group level **(behavioural aggregation).**

**Heuristics**

Mental shortcuts that ease the cognitive load of making a decision, e.g. [rule of thumb](https://en.wikipedia.org/wiki/Rule_of_thumb), an [educated guess](https://en.wikipedia.org/wiki/Ansatz), an [intuitive](https://en.wikipedia.org/wiki/Intuition) judgment, a “[guesstimate](https://en.wikipedia.org/wiki/Guesstimate)”, [profiling](https://en.wikipedia.org/wiki/Profiling_(information_science)), or [common sense](https://en.wikipedia.org/wiki/Common_sense).

**Linear opinion pooling**

A mechanistic rule for combining probabilities or distributions elicited from two or more sources into a single probability or distribution. In linear opinion pooling the single distribution is calculated as the unweighted linear average of individual distributions.

**Mathematical aggregation**

Combining the beliefs of individual experts using a mathematical rule, such as **linear opinion pooling.**

**Model based economic evaluation (MBEE)**

An evaluation of cost-effectiveness which employs some form of decision model or statistical model.

**Normative expertise**

The expert’s ability to accurately assess and clearly communicate their beliefs in probabilistic form.

**Observable quantities**

A quantity that could be estimated as a simple function of observed data, if that data were available. For example, the probability of an outcome, which can be estimated by the observed frequency of the outcome. This contrasts with composite quantities such as odds ratios which are complex functions of observable data and generally more difficult for an expert to conceptualise.

**Parameters**

A variable within an analysis, for example a **MBEE** or a regression model.

**Precision**

A measure of statistical variability or statistical bias. Repeated measures are said to be precise if the values are close together. Calculated as the reciprocal of the variance.

**Probabilistic**

Relating to probabilities. Involving quantities whose values are uncertain, or which may take multiple possible values. Probabilistic sensitivity analysis is used to explore the consequences of uncertainty in **parameter** inputs in **MBEE.**

**Probability**

A measure of the likelihood/chance of occurrence of a particular event. Can take values between 0 and 100%.

**Proportion**

The number of something in comparison to the whole, e.g. the proportion of females in the population. Can take values between 0 and 100%.

**Quantiles**

Points on a probability distribution which divide it into continuous intervals. The 50^th^ quantile is known as the *median*.

**Relative risk**

The ratio of the probability of an event occurring in the exposed or treated group versus the probability of the event occurring in the non-exposed group.

**Structure Expert Elicitation (SEE)**

Refers to a formal documented process by which experts beliefs (priors) are obtained in a quantitative form.

**Subjective beliefs**

An individual’s own beliefs/opinions about an uncertain quantity, which may be expressed as a distribution. If this is prior to data collection/availability, this is called a “prior” distribution.

**Substantive expertise**

An expert is said to be a substantive expert if they possess skills/knowledge pertaining to a particular domain or subject within that domain.

**Survival function**

Defined as the probability that the time T that an event (e.g. death) occurs is greater than t, P[T>t]. It can be defined as $S(t) = exp\{-\int_{0}^{t} h(u)du\} = exp\{H(t)\} ,$where $\int_{0}^{t} h(u)$ is the cumulative hazard function, H(t). Survival can alternatively be described using the probability density function for the survival times, f(t), using the following relationship: S(t) = 1 - F(t)= 1 - ∫f(t)dt , where ∫f(t)dt is the cumulative distribution function, F(t). The hazard and the probability density functions can also be used together to determine survival S(t) = f(t)/ h(t).

**Variance**

A measure of the spread of a random variable.

**Variable interval method**

A method to elicit a distribution. The expert is asked to express the quartiles or credible intervals of a distribution, e.g. tertiles are used in the **bisection method.**

**Validity**

Generally refers to the quality of making logical sense. In SEE, validity can mean that the exercise captured what the experts believe, or that the expressed quantities correspond to reality, or are consistent with the laws of probability, or are internally coherent.

# Abbreviations

HTA	Health Technology Assessment

NICE	National Institute for Health and Clinical Excellence

RCT	Randomised Control Trial

SEE	Structured Expert Elicitation

SHELF	Sheffield Elicitation Framework

HCDM	Healthcare decision-making

DAR	Diagnostic Assessment Report

EFSA	European Food Safety Authority

EPA	Environmental Protection Agency

IFA	Institute and Faculty of Actuaries

NRC	Nuclear Regulation Commission

GP	General Practitioner

CCG	Clinical Commissioning Group

LA	Local Authority

DHSC	Department of Health and Social Care

MRC	Medical Research Council

PHE	Public Health England

IFR	Individual Funding Request

SMC	Scottish Medicines Consortium

AMWSG	All Wales Medicines Strategy Group

HPA	Health Protection Agency

PHP	Public Health Programme

STA	Single Technology Appraisal

NIHR	National Institute for Health Research

MBEE	Model Based Economic Evaluation

FIM	Fixed Interval Method

VIM	Variable Interval Method

PEGs	Prior Elicitation Graphical Software

GEM	Generalised Expertise Measurement

E-SQ	Expert-Selection Questionnaire

IDEA	Investigate, Discuss, Estimate, Aggregate

CDF	Cumulative Distribution Function

STM	State Transition Model

DES	Discrete Event Simulation

HRQoL	Health Related Quality of Life

TPM	Transition Probability Matrix

CrIs	Credible Intervals

KL	Kulback Leibler

MCMC	Markov Chain Monte Carlo

SDR	Standard Deviation Ratio

lnSDR	Log of Standard Deviation Ratio

lnKL	Log of Kulback Leibler

SCHARR	Sheffield School of Health and Related Research

FeNO	Fractional Exhaled Nitric Oxide

BTS	British Thoracic Society

SIGN	Scottish Intercollegiate Guidelines Network

FTE	Full-Time Equivalent

# **Plain English Summary**

*Background*

Decisions in health care aim to maximise health, requiring judgements about treatments. The evidence used to make these judgement is typically uncertain.

In these situations, the experience of experts is essential. Structured expert elicitation (SEE) collects beliefs from experts. There are different guidelines available for SEE, however it is not clear if any of these be can be used in healthcare decision making (HCDM), for example in considering if a treatment should be made available in the NHS.  This project aimed to develop a guidance for SEE to inform HCDM.

Methods

Reviews and experimental techniques were used to gather a list of methods to conduct SEE. The suitability of these choices in HCDM was then determined by comparing these with a set of standards that support the use of SEE in HCDM was generated.

*Results*

Different guidelines prefer different approaches to conduct SEE. There is a lack of evidence available to determine which of these methods is most appropriate across the whole of HCDM.

It is possible to define reference protocol methods that could be used in a particular type of HCDM, Health Technology Assessment (HTA). This includes: gathering experts with knowledge of the clinical area, asking experts about things that they observe in clinical practice and asking experts individually for their beliefs. For decision makers working outside of HTA, for example at a local level, or for treatments that are not yet available to patients, these choices may not be appropriate.

*Conclusions*

This flexibility of this guidance is a useful feature. It is possible for different decision makers in healthcare to interpret the reference protocol for their own circumstances.

# Scientific Summary

*Background*

At the forefront of decisions in healthcare is the aim of maximising health, requiring judgements about interventions that may have higher health effects but potentially incur additional costs. The evidence used to establish cost-effectiveness is typically uncertain, for example the evidence may not be on ‘final’ outcomes (e.g. cancer products licensed on evidence of progression-free survival), or the evidence base may not be well developed (e.g. in diagnostics, medical devices, early access to medicines scheme). It is important that the uncertainty in this evidence is characterised. If not, any analysis using this evidence may give decision makers a misleading view of the risks associated with their decision.

In situations where evidence is subject to uncertainty, the experience of experts may be necessary essential. To ensure accountability in the decision, these expert judgements should be made explicit and incorporated transparently into the decision making process. The process by which the beliefs of experts can be formally collected in a quantitative manner is structured expert elicitation (SEE). If conducted in an appropriate manner, SEE can characterise uncertainties associated with the cost-effectiveness of competing interventions and assess the value of further evidence. This may be the approach best suited to a transparent decision making process.

There is an increasing interest in SEE as new technologies are assessed progressively closer to their launch on the market. SEE is also valuable for ‘early modelling’ of new interventions or unknown diseases for which little or no evidence is available. A review of applied studies in health care decision making (HCDM) found heterogeneity in methodology used and a lack of consideration for any existing guidance on the topic. ^1^

No standard guidelines exist to conduct expert elicitation in health technology assessments (HTA) but there are a number of generic guidance documents, some of which have been used in HTA. The most notable of these are the Sheffield Elicitation Framework (SHELF) and Cooke’s classical method. It is not clear if any of the existing guidelines, generic and domain specific are appropriate for us in HCDM.

*Objectives*

The overall aim of this report was to establish a reference protocol or guideline for the elicitation of experts judgements to inform HCDM. To achieve this overall aim, the report focussed on the following objectives:

1. Providing clarity on the methods for collecting and using experts judgements within an assessment of cost-effectiveness.
2. Exploring where alternative methodology may be required in particular context/constraints e.g. time.
3. Establishing preferred approaches for elicitation for a range of parameters and a range of decision-making contexts.
4. Determining which elicitation methods allow experts express parameter uncertainty as opposed to variability.
5. Determining the applicability and usefulness of the reference protocol developed within a case-study application.

For (4), statistical experiments were conducted. The aim of these experiments was threefold: (1) to evaluate alternative methods of elicitation and how they perform in representing parameter uncertainty; (2) to explore individuals’ ability to extrapolate from their knowledge base; and (3) to explore how individuals revise their answers when presented with group summaries.

*Methods*

To achieve these objectives, a mixed methods approach was used, combining formal systematic review, targeted searches, experimental work and narrative synthesis. Specifically, first a systematic review of existing guidelines for formal elicitation, published in either the peer-reviewed or grey literature, was conducted. This identified the approaches used in existing guidelines (the “choices”) and determined if dominant approaches evolve. Less formal, targeted searches were also conducted to determine the state of the evidence on choices relating to: the selection of experts, level of elicitation, fitting and aggregation, assessing expected accuracy of experts judgements and heuristics and biases. The advantages and disadvantages of each available choice for these elements were extracted from the papers and potential constraints to their application in HCDM determined.

HCDM is not a homogenous domain, as different decision makers face different constraints and this may have implications for expert elicitation methodology. The contexts in which SEE in HCDM may be conducted, is therefore discussed in detail and conclusions made regarding the use of a reference protocol for SEE. Alongside this, a systematic review of SEE applications in cost-effectiveness modelling was undertaken. This details the challenges that were reported by the authors conducting these analyses. When available, the basis for the methodological choices made in each application is extracted. This also provided a view of the current scope of the landscape with regards to applied SEE in HCDM.

When designing a SEE, deciding what quantities to elicit is a major challenge. There is no guidance covering the spectrum of quantities that may be appropriate to elicit to inform HCDM, including measures of treatment effects and baseline event rates. To address this lack of guidance, a review was undertaken of alternative quantities that can be elicited to inform the probability-related or time to event-related parameters commonly used in HCDM.

The statistical experiments, conducted to explore multiple uncertainties in SEE methodology, utilised a simulated learning process (e.g. Wang 2002). Individual’s knowledge was determined by recorded observations. The ‘dataset’ observed then determines participants’ belief about the quantity of interest, from which accuracy can be measured. This approach allows the conditions of the experiment to be defined, e.g. equal vs different knowledge-base, and isolate potential determinants, e.g. precision. Participants were shown random observations from a statistical model representing an abstract medical problem. Following this they were asked to express their beliefs’ regarding treatment effectiveness. All participants (72) were students at the University of York, the large majority of which were undergoing clinical training. The exercises was delivered face-to-face and financial incentives were offered according to accuracy. The experiments measured:

- Bias: difference in the means of the true and elicited (and fitted) distributions;
- Uncertainty: ratio of the SDs of the two distributions.
- Kullback-Leibler divergence (KL): information lost when one distribution is approximated by anotheranother^13^.
- Participants preference for alternative methods.

Given the full range of evidence generated, on which to base a reference protocol for SEE in HCDM, it was necessary to use this evidence to generate a set of principles that underpin the use of expert elicitation in HCDM. Available choices, from the review of guidelines, are considered in light of these principles and any empirical evidence available to support the choices. This informs the reference protocol, by discounting or supporting particular choices.

The work also included an applied evaluation of the developed reference protocol. This uses an existing cost-effectiveness model, in which SEE was used to generate initial estimates of uncertain parameters. In addition to demonstrating the usefulness of the reference protocol in navigating the SEE process, the practicality of SEE is determined using narrative feedback form experts and by generating estimates of resources required to design and conduct the SEE.

Finally, a dissemination workshop was convened, which explored the usefulness, and challenges in using SEE in HCDM. It was also used to refine, using discussion, a set of recommendations for further research.

*Results*

A comprehensive list of elements and choices for SEE was developed by reviewing existing protocols (work package 1). This covered the design, implementation and analysis stages of SEE. The review showed that for many elements of the SEE, there was a lack of consistency across the existing guidelines. Targeted searches also revealed that the majority of choices are not supported by any empirical evidence, both specific to HCDM and more generally.

Empirical evidence, generated by the experiments conducted here (work-packages 2 and 3), determined that there is little difference between variable interval methods (VIM) and fixed interval methods (FIM) methods to encode judgements, in terms of procedural performance. Therefore a decision maker can consider either of these choices suitable. This experiment also determined that participants did not adjust uncertainty levels sufficiently to reflect differences in the underlying heterogeneity of the populations; in particular, uncertainty was consistently underestimated in the case of high heterogeneity. This case is frequently encountered in healthcare settings. The experiments also sought to explore extrapolation beyond data observed and updating of priors after presentation of group summaries – issues which feed into multiple choices for SEE. It was difficult to form definitive conclusions given that the experiments were underpowered for these elements. The experiments did provide some evidence that experts changed their estimates in a rational way when provided with estimates from others, suggesting that group discussion or feedback may be useful. Extrapolation outside of the observed sample does not seem to affect accuracy, suggesting that it is reasonable to ask experts about patients and practices in which they do not have direct clinical experience, or for whom there is no relevant literature.

In order to sift through the available choices, a set of principles that underpin the use of SEE in HCDM was defined using evidence generated from targeted searches, experimental evidence on methods to encode judgements and consideration of the constraints on the decision making processes in health (work package 1). These nine principles are:

1. Transparency
2. Fitness for purpose
3. Consistency, but respecting constraints of the decision making context
4. Reflecting uncertainty at the individual expert level
5. Recognising and acting on biases
6. Suitability for substantive experts who are less likely to be normative
7. Recognising where adaptive skills are required
8. Recognising between-expert variation
9. Promoting high performance

Not all principles for SEE in HCDM were relevant for all elements. The most relevant principles for each element and components within SEE were considered.

In almost all choices, there is a lack of empirical evidence and in some circumstances the principles are unable to provide sufficient justification for discounting particular choices (work package 1). It is, however, possible to define reference methods that could be used in a more narrowly defined area of HCDM, namely HTA. These include:

- Focus on gathering substantive expertise or experience. Normative skills can be developed during the training session as part of the SEE.
- Simple observable quantities should be elicited where possible; ratios or complex parameters such as regression coefficients should not be elicited directly.
- Minimize and record conflicts of interest among the experts. Include experts external to the SEE task, i.e. not those involved in developing the task.
- Dependence between variables should be captured in SEE. Expressing dependent variables in terms of independent variables is preferable when experts do not have strong normative skills.
- Use of either VIM or FIM work well, however decision makers should aim for consistency across applications.
- Beliefs should be elicited from experts individually, even if a group interaction follows.
- Between-expert variation should be explored explicitly.
- Following fitting, a summary of the individual distributions should be obtained using linear pooling.
- Interaction should be face-to-face where possible to allow a facilitator to deliver training to the expert.
- Training is crucial and should focus on avoiding bias and expressing uncertainty.
- All methodological choices for the SEE must be documented and justified.

Additional considerations are required for decision makers outside of HTA, for example at a local level, or for early technologies that have yet to progress through the regulatory process. Access to experts may be limited and in some circumstances, group discussion may be needed to generate a distribution.

The application of the case-study, a diagnostic model for asthma, explored practical issues. This highlighted sufficient information needs to be presented to the experts. The level of information presented to the experts and the wording of this information is paramount in ensuring that the quantity of interest is observable to the expert. When deciding on the information to provide to experts it may be useful to consult existing policies. With regards to time constraints, the applied evaluation was undertaken over a 7 month period and involved 3 analysts in varying proportions. Overall this equated to 5 months full time equivalent researcher time.

*Limitations*

The major limitation of the work conducted here, lies not in the methods employed, but the evidence available from the wider literature, on which to base the set of choices and determine how appropriate these are. Concluding on the suitability of the choices available from the existing guidelines is challenging due to the lack of empirical evidence to support specific choices. Instead, it was necessary to develop principles for SEE in HCDM, using the sources of evidence as described above and published guidelines for good SEE. Using only the principles, in the absence of empirical evidence, meant that it was not always possible to give definitive conclusions on choices.

*Areas for further research*

In considering the appropriateness of choices for SEE in HCDM and exploring how these choices may be affected by the context in which the SEE is applied, there are several areas in which further research is required before definitive statements can be made regarding their appropriateness for a reference protocol. Researchable questions in these areas include the following:

- Which methods for expert recruitment are most practical and what are the challenges?
- What training strategies can be used to minimise bias?
- Which methods for eliciting dependent quantities work best for non-normative experts?
- Which consensus approach works best in HCDM in practice and for which types of quantities and decision makers?
- Should individual priors be combined when there is significant expert variation? If so, how?

At the dissemination workshop, participants were asked to discuss areas for further research, specifically considering what decision makers in HCDM may require when determining a reference protocol for SEE for use within their setting. Participants were not asked to define which research topics are highest priority for their setting. Selecting experts, minimising bias, adaptation to specific setting in which SEE may be applied, for example choosing individual or group elicitation, appropriate wording of questions, methods for multivariate elicitation and what information should be presented to the experts to help them formulate their beliefs. Some of these topics would benefit from empirical research and others may be resolved though application of the proposed reference protocol to HCDM, including in settings with a range of constraints.

*Conclusions*

SEE can offer opportunities in HCDM, particularly reimbursement decisions supported by model based economic evaluation (MBEE). SEE allows the uncertainty in the evidence used to populate these models to be characterised, or where evidence is completely lacking, provides additional information needed to reach a decision.

The work described in this report has attempted to generate evidence which is useful for analysts and decision makers in HCDM. SEE conducted in this context to date, has not used a set of consistent methods, and above all, has not considered the implications of the choices made when designing and conducting a SEE. To improve the accountability of HCDM the procedure used to derive expert judgements should be transparent.

The reference protocol presented here is intended to serve as a guide to good practice and reporting, and is flexible in many choices rather than being prescriptive regarding methods. It can therefore be thought of as a reference guide. This was necessary due to the lack of empirical data specific to HCDM and more generally in SEE. This may be a useful characteristic, as it is possible to apply this reference protocol across different settings.

*Funding details*

This work was funded by the Medical Research Council. HEE: Developing a reference protocol for expert elicitation in health care decision making. Bojke L. 2017. Reference: MR/N028511/1.

# Chapter 2 Background

In the UK, decisions about the use of health care interventions are made by various NHS organisations as well as the immediate beneficiaries, namely patients. In England, these NHS organisations include the National Institute for Health and Care Excellence (NICE), NHS England and Public Health England. At the forefront of these decisions is the aim of maximising health, calling for judgements about the interventions that are expected to lead to higher health effects. Where resources are limited, additional costs incurred will impact on access to care for other patients, and health foregone in this way should also be taken into account (a cost-effectiveness framework).^2^

Although Randomized Control Trials (RCTs) have been described as the principle source of evidence for such decision-making, RCTs have considerable limitations including a lack of external validity, short study periods to assess long-term treatment effect and invalid generalizations of findings outside the study group.^3-5^ Also RCTs are not possible or ethical in some situations.

These limitations also impact the use of RCTs for urgent health issues for which decisions need to be made promptly on the basis of limited and often imperfect available data.^6^ Health Technology Assessments (HTAs) traditionally use decision-modelling methods that gather different forms of evidence by defining mathematical relationships between a varied set of input parameters, in a way that describes aspects of the history of the disease of interest and the impact of the intervention.

Uncertainty in the evidence is pervasive in cost-effectiveness modelling and the analysis may be biased if uncertainty in the model inputs is not reflected. Uncertainty can be distinguished as epistemic or aleatory.^7, 8^ Aleatory uncertainty arises due to randomness i.e. unpredictable variation in a process and expert knowledge cannot reduce this type of uncertainty.^7^ Therefore, it is sometimes referred to as irreducible uncertainty. Epistemic uncertainty is due to imperfect knowledge and it can be reduced with sufficient study and therefore, expert judgement may be useful in its reduction.^7^ Additional evidence can reduce uncertainty and provide a more precise estimate of cost-effectiveness. By quantifying uncertainty, it is possible to assess the potential value of additional evidence, inform the types of evidence that might be needed, and consider restricted use until the additional evidence becomes available.^9^

In some situations, several input parameters in the decision model may have only limited empirical data. For example, the evidence may not be on ‘final’ outcomes (e.g. cancer products licensed on evidence of progression-free survival), or the evidence base may not be well developed (e.g. in the areas of diagnostics, medical devices, early access to medicines scheme, or public health). In these situations, judgements are required for a decision to be reached regarding that parameter. To ensure accountability in the decision, these judgements should be made explicit and incorporated transparently into the decision making process, an inherently Bayesian view on decision-making. Formal methods to quantify prior beliefs in the form of experts judgements exist, and are termed Structure Expert Elicitation (SEE) methods.^8^

Structural Expert Elicitation (SEE) is a process that allows experts to express their beliefs in a statistical, quantitative form. If conducted in an appropriate manner, SEE is the best approach to characterise uncertainties associated with the cost-effectiveness of competing interventions and assess the value of further evidence. SEE methods have been used in disciplines including weather forecasting and reliability analysis within engineering^10^ but the research findings in these disciplines are often interpreted as contradictory, in particular the appropriateness of generating consensus amongst experts.^11^ In terms of SEE in healthcare, NICE uses expert judgement across all guidance-making programmes but, expert elicitation (compared to expert opinion) is used less frequently.^12^ Existing timelines and consequent time constraints are reported as the common obstacles when conducting expert elicitation in healthcare.^12^

There is an increasing interest in SEE, as HTAs are conducted progressively closer to the launch of the intervention of interest.^13^ SEE is also essential for ‘early modelling’ of new interventions or unknown diseases for which little or no evidence is available.

No standard guidelines exist to conduct expert elicitation in HTA but there are a number of generic guidance, some of which have been used in HTA.^14, 15^ The most notable of these is the Sheffield Elicitation Framework (SHELF)^15^. This is a package of documents, templates and software for eliciting probability distributions. The method begins by eliciting judgements from each expert individually and then elicits a single probability distribution from the group of experts. Cooke’s classical method is another generic technique that has been applied in HTA. This method primarily focuses on the synthesis of multiple experts beliefs. Patients are scored based on their performance on calibration questions (questions for which experts do not know true values) and their assessments are weighted according to their scores.^16^ The third generic guidance applied in HTA is the DELPHI method. This is an iterative survey that provides feedback from the experts over successive rounds providing an opportunity for consensus as experts review their opinions based on new information from their peers.^17^

While generic processes have been applied in HTA (*Figure 1*), there is an absence of a published guidance that is specific to HTA. Certain elements of the generic guidance may not be appropriate in a HTA context due to resource and time constraints that are inherent in HTA.

At present, an analyst needs to be aware of a number of key issues to consider when designing, conducting and analysing an elicitation exercise. In terms of the design, the analyst must decide what quantities to elicit. This will largely be informed by the requirements of the decision model. As a rule, experts should be asked to express their beliefs about observable quantities such as probabilities rather than unobservable quantities i.e. moments of a distribution or covariates. Once the quantities have been chosen, the next choice will be based on which method(s) will be employed to express the parameters. Possible methods include fixed or variable interval methods. The analyst must then choose which experts should be recruited to elicit these judgements. Once the beliefs have been elicited, a decision must be made on how to synthesis the beliefs.

There is heterogeneity in the existing methodology used in HTA. Given the lack of guidance, there is a need to develop a standard set of principles to guide the design and conduct of expert elicitation in HTA. It is essential that the elicited information represent how uncertain experts are about the current state of knowledge regarding a parameter of interest. There is a need to reflect the range of reasonable judgements that may be expressed across experts (between-expert variation) and determine how decision makers use these elicited judgements in the decision making process.

**Figure 1 General schematic for SEE**

The overall aim of this report was to establish a reference protocol or guideline for the elicitation of experts judgements to inform healthcare decision-making (HCDM). To achieve this overall aim, the report will focussed on the following objectives:

- Providing clarity on the methods for collecting and using experts judgements within an assessment of cost-effectiveness.
- Demonstrating where alternative methodology may be required in particular context/constraints e.g. time.
- Establishing preferred approaches for elicitation for a range of parameters and a range of decision-making contexts.
- Determining which elicitation methods allow experts express parameter uncertainty as opposed to variability.
- Determining the applicability and usefulness of the reference protocol developed within a case study application.

The initial research protocol outlined two additional objectives: to establish the accuracy of consensus-based methods in generating representations of uncertainty, and to establish the accuracy of alternative methods of mathematically pooling the individual judgements of experts. The objectives were subsequently refined to explore individual factors that can affect the accuracy of consensus-based methods, in particular: to explore individuals’ ability to extrapolate from their knowledge base, and to explore how individuals revise their answers when presented with group summaries. Further details on the reason for these deviations is provided in Chapter 8 .

To achieve these objectives, the activities of this project were split into three work packages and an evaluation. The activities of the project are summarised below in Figure 2.

**Figure 2 Summary of project activities**

**OBJECTIVE:**

**ESTABLISH A PROTOCOL FOR EXPERT ELICITATION IN HEALTHCARE DECISION MAKING**

**Rationale**:

1. Need for standardised protocol to design and conduct expert elicitation
2. Need to represent how uncertain experts are about the current knowledge of a certain parameter
3. Reflect range of reasonable judgement expressed across experts (between-expert variation)
4. Decision makers need to be able to use the elicited judgements

**WP1: Appraisal of existing protocols for eliciting distributions**

Review of design, conduct and analysis

Critical Appraisal of protocols

**WP2: Eliciting distributions to represent uncertainty**

Experiment to determine how well quantitative expressions derived from different methods match descriptions of uncertainty

**WP3: Understanding and appropriately characterising between-expert variation**

Experiment to establish the accuracy of a single consensus-based method

Establish the accuracy of alternative methods of mathematically pooling the individual judgements of experts

**Applied evaluation of developed reference case**

- Apply the reference case in a retrospective manner to a case-study
- Explore any practical issues throughout the SEE process
- Evaluate time and cost elements of using the protocol within a ‘real time’ decision
- Workshop to agree a protocol with key policy makers

Specifically the remaining chapters in this report provide the following:

*Chapter 2* reviews existing guidelines for formal elicitation (SEE). This review identifies the approaches used in existing guidelines and aims to identify whether dominant approaches evolve in terms of the choices that need to be made in the elicitation process.

In light of this review, *Chapter 3* considers contexts for structured elicitation in HCDM. Different contexts may influence the requirements and feasibilities of expert elicitation and so *Chapter 3* discusses this in detail and identifies the potential constraints in decision-making in healthcare and discusses the implications for expert elicitation methodology.

*Chapter 4* is a review of SEE applications in cost-effectiveness modelling. The chapter summarises the basis for the methodological choices made in each application and details the challenges that were reported by the authors.

*Chapter 5* reviews the evidence on the potential choices that are available for different components of the elicitation process. This focuses on the elements: selection of experts, level of elicitation, fitting and aggregation and adjusting judgements. This chapter discusses the advantages and disadvantages of each available choice and identifies any potential constraints to their application in cost-effectiveness analyses.

Heuristics and biases are concerns that are predominant across all elements in SEE and SEE should be conducted in such a way that minimises these errors. *Chapter 6*reviews the existing evidence on heuristics, biases and de-biasing techniques that are of most relevance to HCDM.

*Chapter 7* discusses what quantities to elicit. This chapter provides a list of alternative quantities that can be elicited to inform certain types of parameters that are commonly used in healthcare. This is particularly relevant in cost-effectiveness analyses, as parameters are often complex constructs, such as relative treatment effects or time to events, which experts will not directly observe in practice. *Chapter 7* compiles a list of alternative quantities that may be elicited to inform specific parameters.

*Chapter 8* provides the experimental plan for experiments that were conducted as part of this research. The aim of these experiments was threefold: (1) to evaluate alternative methods of elicitation and how they perform in representing parameter uncertainty; (2) to explore individuals’ ability to extrapolate from their knowledge base; and (3) to explore how individuals revise their answers when presented with group summaries. The results and interpretation of these experiments is then presented.

*Chapter 9* discusses the methodological choices for each of the different components of SEE: design, conduct and analysis. Managing biases and validity assessment are then considered as overarching concerns for throughout the SEE process. In order to conclude on their suitability for HCDM, *Chapter 9* first presents a set of principles that underpin the use of expert elicitation in HCDM. Available choices are considered in light of these principles and any empirical evidence available to support the choices.

Chapters 2 to 9 are then used to generate a reference protocol for HCDM (*Chapter 10*). This presents the choices that are supported by the principles for HCDM, and/or empirical evidence in this domain. Given the paucity of empirical evidence relating to HCDM, it was necessary to define this for a specific type of HCDM, HTA. Considerations when using the reference protocol outside of this context are also presented.

*Chapter 11* describes the applied evaluation of the developed reference protocol. This uses an existing cost-effectiveness model, in which SEE was used to generate initial estimates of uncertain parameters. In addition to demonstrating the usefulness of the reference protocol in navigating the SEE process, the practicality of SEE is determined using narrative feedback form experts and by generating estimates of resources required to design and conduct the SEE.

The report closes with discussion and conclusions *(Chapter 12*) based on the findings of this research. The feedback from a dissemination workshop, exploring the usefulness and challenges in using SEE in HCDM are reported. The limitations of the research and areas of further research are also discussed here.

# Chapter 3 Good practice in SEE: Learning from the available guidance

## Section 3.1 Introduction

Over the last few decades, SEE has been used in areas such as natural hazards, environmental management, food safety, healthcare, security and counterterrorism, economic and geopolitical forecasting, and risk and reliability analysis. All of these areas require consequential decisions be taken in the face of significant uncertainty about future events or scientific knowledge.

How judgements are elicited is critical to the quality of the resulting judgements and hence the ultimate decisions and policies. Methods for SEE should be suitable for specific contexts and understood by content experts to be useful to decision makers. Example applications and recommended practices do exist in certain fields, but the specifics vary.

In developing a reference protocol for SEE specific to the needs of HCDM, the methodological recommendations and choices, which exist in other fields, need to be understood. This chapter surveyed existing best practices for SEE as reflected in published elicitation guidance to identify areas of consensus, places where no consensus exists, and other gaps. Identifying areas of commonality across current guidance can support elicitation practice in areas that lack context-specific guidance such as HCDM. The recommendations and choices for the SEE process identified in this chapter are further explored in *Chapter 5, Chapter 6, Chapter 7* and *Chapter 8*, and their suitability for HCDM is considered in *Chapter 9*.

## Section 3.2 Methods

To identify areas of agreement and disagreement in elicitation practice, both domain-specific and generic elicitation guidelines were systematically reviewed according to the search strategy and screening process detailed in **Error! Reference source not found.**. A *SEE guideline* is defined as a document, either peer-reviewed or in the grey literature, that advises on the design, preparation, conduct, and analysis of a structured elicitation exercise. The review focussed on SEE guidelines rather than applications to determine a full list of the possible methodological options rather than relying on the partial reporting available in applications.

To constrain the scope of this review, guidelines needed to concern explicitly probabilistic judgements and offer guidance on more than one stage of the elicitation process. Literature relating to only one element of elicitation is considered in the targeted searches discussed in *Chapter 5* and *Chapter 6*. When the same or similar author lists published multiple guidance documents making similar recommendations, only one version was included. An extraction template was used to collect information from each guideline. The extracted data was analysed to create an overview of all the stages, elements, and choices involved in an elicitation and to understand where current advice across guidelines conflicts or agrees. Where the guidelines agree, we assume this represented best practice which can be taken forward within the HCDM context, as applicable. Where the guidelines disagree, we sought additional evidence to support the development of a reference protocol for HCDM (*Chapters 3-8*). .

## Section 3.3 Included SEE guidelines

The searches identified 16 unique SEE guidelines (**Error! Reference source not found.***, Table 2*). Five of the guidelines are generic and aim to inform practice across disciplines, and 11 focus on specific domains. Six of the domain-specific guidelines are agency white papers or agency-sponsored peer-reviewed articles and are tailored to the specific decision-making processes the agencies govern. Agencies issuing guidelines include the European Food Safety Authority (EFSA), U.S. Environmental Protection Agency (EPA), the Institute and Faculty of Actuaries (IFA), and the U.S. Nuclear Regulatory Commission (NRC). Both the IFA and NRC have published two distinct guidelines. The 10 guidelines not connected to agencies are based on reviews of existing evidence and practice about elicitation methods (two guidelines), reflections on personal experience and practice (three guidelines), or combinations of review and reflection (five guidelines).

Two of the agency SEE guidelines were included with caveats. First, the EFSA guideline covers three distinct elicitation methods, but the Classical Model and SHELF are presented in other guidelines, so only the portions of the EFSA document related to the EFSA Delphi method are included in this review.^17^ Second, the EPA guideline is a White Paper released for public review that was not intended to be the final agency report on the subject.^18^ However, a final version was never released, and thus the document is widely cited in elicitation literature and has served as a de facto guideline as nothing has superseded it.

## Section 3.4 Analysis of the elicitation process

Although the characterization of the process, including the number and categorization of steps, differed among the 16 guidelines, the underlying elicitation process described, depicted in *Figure 3*, was remarkably similar.

**Figure 3 The elicitation process**

At each step of the elicitation process, analysts are faced with a variety of methodological choices. *Table 1* provides the full list of choices described in the 16 guidelines, and *Table 2* summarises the level of agreement in the recommendations and choices discussed for each element. The following sections discuss the variety of methodological recommendations for each stage made across the guidelines. See *Supplementary material 1, Tables 4-15* for further detail.

**Table 1 Summary of the elicitation elements, components, and choices described in SEE guidelines**

| Element | Component | Choices |
| --- | --- | --- |
| **Identifying elicitation variables** | | |
| What quantities to elicit | Type of parameter | 1. Elicit observable quantities 2. Elicit required model parameters directly |
|  | Type of quantity | 1. Proportions 2. Frequencies 3. Probabilities 4. Odds ratios |
|  | Selection criteria | 1. Define selection criteria (probabilities, consequences, constraints, etc) 2. Minimal assessment of each possible uncertain parameter and sensitivity analysis to see which uncertain parameters have the biggest impact |
|  | Principles for describing quantities | 1. Ask clear and well-defined questions 2. Ask questions in a manner consistent with how experts express their knowledge 3. Uncertainty in the elicited variables should impact the model and/or decision 4. Use neutral wording |
|  | Decomposition/disaggregation | 1. Decompose variables of interest to aid experts in the elicitation task 2. Don’t decompose variables for the experts |
|  | Handling dependence | 1. Express dependent variables in terms of independent variables 2. Use conditional probabilities 3. Use other dependence elicitation methods |
| Encoding judgements | General approach | 1. Fixed interval method:    - Roulette or chips and bins method    - Ask for % that falls within a specific range 2. Variable interval method:    - Quantiles (Quartiles, Tertiles, 5%, 95% & median, 17%, 83% & median, other)    - Bisection    - Plausible probabilities (Lowest plausible probability, Highest plausible probability, Best guess for the probability)    - Plausible quantities (Upper and lower plausible bounds, best guess, degree of belief)    - NUSAP (Numeral, Unit, Spread, Assessment, Pedigree) 3. Hybrid fixed/variable interval methods 4. Summary statistics, moments, measures of central tendency 5. Elicit evidence, not parameter values, and analyst/facilitator defines probability distribution that reflects the body of evidence 6. Other |
|  | Use of visual aids | 1. Use to aid elicitation task 2. Do not use |
| **Identifying and selecting experts** | | |

| Number of experts | Number of experts | | 1. Depends on application 1. Options mentioned in different guidelines: about 10; about 5 specialists and 2-3 generalists; 10-20; 6-e12; at least 4; 8 a “rule of thumb”; 5-9 | |
| --- | --- | --- | --- | --- |
| Selecting experts | Roles within SEE | | 1. Facilitator (assessor, analyst, coordinator): prepare and conduct elicitation 2. Expert (technical expert, specialist, subject-matter expert): provide judgments (and/or evidence) 3. Generalists: may provide judgments, advise on design, or help with the elicitation | |
|  | Desired characteristics for those providing judgements | | 1. Normative expertise 2. Substantive expertise 3. Willingness (interest and availability) to participate 4. Ability to understand questions 5. Ability to apply skills 6. Notability | |
|  | Identification procedure | | 1. Recommendations by peers, either formally or informally 2. Research outputs 3. Known experience 4. RFP to seek out experts 5. Profile matrix to identify types of expertise required | |
|  | Selection procedure | | 1. Disclosure of personal and financial interests 2. Pursue diversity in opinions, specialisation, area, institution, etc. 3. Pursue diversity in age, gender, culture 4. Formal selection criteria developed and applied 5. Send potential experts a questionnaire 6. Review CVs of possible experts and have a committee select accordingly 7. Match possible experts against profile matrix | |
|  | Possible selection criteria | | 1. Reputation 2. Experience and qualifications 3. Publication history 4. Diversity in background 5. Conflicts of interest 6. Awards 7. Balancing different viewpoints and managing group dynamics 8. Peer assessment (such as GEM) 9. Convenience 10. Balance of internal and external experts (e.g., include at least 2 external experts) | |
| **Training and preparation** | | | | |
| Pilot the protocol | Pilot exercise | | 1. Pilot 2. No mention of pilot | |
| Training and preparation for experts | What to cover in training | | 1. Probability, including subjective probability, and related concepts 2. Motivation for elicitation 3. Description of what is required from experts 4. How results will be used 5. Elicitation questions 6. Example and practice questions 7. Review of potential biases 8. Relevant background information, data, and sources 9. Review assumptions and definitions used in the elicitation 10. Description of performance assessment (if relevant) 11. Introduction to dependence (if relevant) | |
| **Conducting the elicitation** | | | | |
| Mode of administration | | Location | | 1. Face-to-face    - 1-on-1    - Group    - Plenary 1. Remote (web, mail, email, phone, video conference, etc) |
| Level of elicitation | | Level of elicitation | | 2. Individual 3. Group 4. Combination (individual assessment followed by group discussion and assessment) |
| Feedback and revision | | Type of feedback | | 1. Graphical feedback 2. Fitted distributions 3. Written description of the expert’s rationale 4. Rationales from other experts 5. Data collected in the future 6. Discussion of elicited values 7. The expert’s performance scores 8. Result of using elicited values in the model 9. Decision resulting from the expert judgment 10. Draft elicitation report |
|  |  | What to feed back | | 1. The individual’s judgments 2. Aggregated group judgments 3. Judgments from other individual experts |
|  |  | Opportunity for revision | | 1. Iterate elicitation/feedback rounds 2. Update after future data is collected 3. Update for revisions/clarifications after circulating draft elicitation report |
| Interaction | | Opportunity for interaction | | 1. No interaction 2. Group discussion prior to individual elicitation 3. Group discussion and group elicitation 4. Group discussion following individual elicitation (with opportunity for revision) 5. Remote, anonymized interaction |
| Rationales | | Rationales | | 1. Collect/record rationales from experts (about how they made their judgments) 2. Collect/record rationales from decision makers (about how they used the expert judgments) |
| Aggregation | | Aggregation | | 1. Aggregate 2. Don’t aggregate    - Analyst provides a distribution that captures knowledge from all experts (the Kaplan approach)    - Only use individual distributions |
|  |  | Aggregation approach | | 1. Mathematical    - Opinion pool: equal-weighting, performance-based weighting (with seed questions), analyst-defined weighting (based on rationales, expert qualifications, or other criteria)    - Bayesian aggregation 2. Behavioural 3. Combination 4. Other |
| Fit to distribution | | Fit | | 1. Fit to parametric distribution 2. Use non-parametric approaches 3. Don’t fit at all |
|  |  | Distribution | | 1. Uniform 2. Triangular 3. Uniform over elicited intervals 4. Normal/beta/other parametric distribution |
|  |  | Fitting method | | 1. Minimum least squares 2. Method of moments 3. Other |
| **Post-elicitation** | | | | |
| Feedback on process | Feedback from experts on process | | 1. Get feedback on the procedure if future data collection contradicts elicitation results 2. Ask experts to appraise the elicitation exercise after completing it | |
| Adjusting judgements | Methods for adjusting judgments | | 1. Do not adjust experts’ assessments 2. Possible adjustments    - Calibrate experts’ assessments    - Adjust to improve coherence (described by Lindley et al. (1979))    - Small adjustments allowed, if they are fed back to the experts    - Drop an expert from the panel | |
| Documentation | What to include | | 1. Elicitation questions 2. Responses from individual experts (if elicited) 3. Description of process and assumptions for fitting a distribution 4. Discussion of elicitation procedure (and justification for choices made) 5. Rationales 6. Evidence related to elicited quantities 7. Aggregated judgements and/or consensus curves 8. Discussion of use/impact of elicitation results 9. Recording of session(s) 10. List of experts 11. Definitions and assumptions 12. The process for updating judgments | |
| **Managing heuristics and biases** | | | | |
| Managing heuristics and biases | Biases relevant for SEE | | 1. Cognitive biases    - Overconfidence    - Representativeness    - Availability    - Anchoring and adjustment    - Conservatism    - “law of small numbers”    - Hindsight bias    - Discrepancy between expert’s beliefs and responses (conscious or unconscious)    - Location errors    - Tacit assumptions    - Inconsistency 2. Motivational biases    - Management bias    - Expert bias    - Social pressure    - Group think    - Impression management    - Wishful thinking    - Misinterpretation    - Misrepresentation | |
|  | Bias elimination or reduction strategies | | 1. Give experts practice and feedback 2. Identify biases through discussion with experts 3. Provide training on biases 4. Frame questions to minimize biases and ambiguity 5. Provide relevant background evidence 6. Ask for upper/lower bounds first 7. Ask experts to specify the credible interval they have provided 8. Minimize and record conflicts of interest among the experts 9. Require the experts address conflicting information 10. Collect rationales from experts 11. Report anonymous results 12. Anticipate likely biases 13. Ask experts about evidence, not the probability 14. Avoid numbers in questions | |
| **Considering the validity of the process and results** | | | | |
| Validation | Characteristics of validity and supporting actions | | 1. Faithfully capturing experts’ beliefs  - Provide feedback (graphical feedback often mentioned) - Calibration could be a pragmatic proxy - Test that the question is understood  2. Fitness for purpose 3. Calibration  - Ask questions with realizations (i.e., seed questions) that allow calibration to be tested  4. Calibration and informativeness scoring on seed questions (i.e., the Classical Model)  - Score experts according to calibration and informativeness - Use scores as basis for performance-based weights (related to Aggregation choices) - Score both individual experts and combinations of experts  5. Coherence  - Ask for sets of probabilities that allow coherence to be tested - Overfitting (asking for one more summary than is needed) - Ask for rationales from experts  6. Consistency  - Ask for rationales from experts (and check for inconsistencies) - Provide feedback - Derive/give feedback on density function during elicitation - Multiply/integrate decompositions during elicitation - Use different elicitation methods and compare results  7. Internal peer review of process and/or results 8. External peer review of process and/or results | |

**Table 2 Level of agreement on recommendations and choices in SEE guidelines**

| Element | Component | Agreement level | Explanation |
| --- | --- | --- | --- |
| **Identifying elicitation variables** | | | |
| What quantities to elicit | Type of parameter | Some disagreement | Guidelines agree that observable quantities are preferred, but disagree on whether directly eliciting model parameters is an acceptable choice. |
|  | Type of quantity | Disagreement | Guidelines offer conflicting recommendations on whether eliciting probabilities (compared with other uncertain quantities) is an acceptable choice. |
|  | Selection criteria | Some agreement | Fewer than five guidelines discuss this, but they agree selection criteria should be defined. |
|  | Principles for describing quantities | Some agreement | Some guidelines describe slightly different principles (e.g., asking clear questions, ensuring uncertainty on elicited parameters impacts the final decision or model), but they do not conflict. |
|  | Decomposition | Agreement | The guidelines that discuss decomposing the variables of interest all agree it should be a choice. |
|  | Handling dependence | Some agreement | The guidelines that discuss dependence agree it should be avoided if possible or addressed separately, but they discuss a range of methods for considering dependence. |
| Encoding judgements | General approach | Disagreement | Guidelines recommend and discuss different, conflicting methods for encoding judgements. |
|  | Use of visual aids | Some agreement | Fewer than five guidelines discuss this, but they agree visual aids can be a useful choice. |
| **Identifying and selecting experts** | | | |
| Number of experts | Number of experts | Agreement | The experts agree that multiple experts are important, with most guidelines recommending around 5-10 experts. |
| Selecting experts | Roles within SEE | Agreement | The guidelines are very consistent in their description of the roles involved with elicitation. |
|  | Desired characteristics for those provide judgements | Some agreement | Characteristics discussed in the guidelines are largely consistent, aside from differing views on if normative expertise is a requirement or just desired. |
|  | Identification procedure | Some agreement | Recommendations differ but do not conflict across the guidelines. Agency guidelines tend to offer more detail. |
|  | Selection procedure | Some agreement | Recommendations differ but do not conflict across the guidelines. Agency guidelines tend to offer more detail. |
|  | Possible selection criteria | Some agreement | Recommendations differ but do not conflict across the guidelines. |
| **Training and preparation** | | | |
| Pilot the protocol | Pilot exercise | Agreement | Almost all guidelines recommend conducting a pilot exercise. |
| Training and preparation for experts | What to cover in training | Some agreement | The lists of what should be included in training vary across guidelines but do not conflict. |
| **Conducting the elicitation** | | | |
| Mode of administration | Location | Some agreement | Most guidelines agree that face-to-face administration is preferred, though remote options may be pragmatically useful alternative in some situations. |
| Level of elicitation | Level of elicitation | Disagreement | Guidelines recommend and discuss conflicting levels of elicitation. |
| Feedback and revision | Type of feedback | Some agreement | Recommendations differ but do not conflict across the guidelines. |
|  | What to feed back | Some agreement | Recommendations differ but do not conflict across the guidelines. |
|  | Opportunity for revision | Some agreement | Guidelines either recommend revision take place following an elicitation (as part of an iterative process or immediately following the elicitation) or further in the future, following a draft report or additional data collection. |
| Interaction | Opportunity for interaction | Disagreement | Guidelines offer conflicting recommendations about when and how to facilitation interaction between the experts. |
| Rationales | Rationales | Agreement | Almost all guidelines recommend collecting expert rationales in some form. |
| Post-elicitation | | | |
| Aggregation | Aggregation | Agreement | All guidelines discuss aggregation as a recommendation or valid choice. |
|  | Aggregation approach | Disagreement | Guidelines offer conflicting recommendations on the approach and method to aggregate judgements. |
| Fit to distribution | Fit | Some disagreement | The guidelines make few recommendations, but their choices differ. |
|  | Distribution | Some agreement | Fewer than five guidelines discuss this, but they generally agree that many parametric distributions could be chosen. |
|  | Fitting method | Some agreement | Fewer than five guidelines discuss this, but they generally agree that choices include minimum least squares and method of moments. |
| Feedback on process | Feedback from experts on process | Some agreement | Fewer than five guidelines discuss this, and they recommend complementary approaches. |
| Adjusting judgements | Methods for adjusting judgements | Some disagreement | Fewer than five guidelines discuss this, but they offer different perspectives. |
| Documentation | What to include | Some agreement | The lists of what should be included in final documentation vary across guidelines but do not conflict. |
| Managing heuristics and biases | | | |
| Managing heuristics and biases | Biases relevant for SEE | Some agreement | The lists of potential biases vary across guidelines but do not conflict. |
|  | Bias elimination or reduction strategies | Some agreement | The list of possible strategies vary across guidelines but do not conflict. |
| Considering the validity of the process and results | | | |
| Validation | Characteristics/measures | Disagreement | The guidelines differ in their definitions of validity and discussion of how the concept can be operationalised in an elicitation. |

## Section 3.5 Identifying elicitation variables

### Section 3.5.1 What quantities to elicit

SEE is often done in areas with many relevant uncertainties, and a decision has to be made about what will be elicited. Only 1 of the 16 guidelines^19^ does not provide advice on selecting what quantities to elicit. Recommendations and choices from the other guidelines are summarised in ***Error! Reference source not found.***, *Table 3*.

Five guidelines recommend that elicited variables should be limited to quantities that are, at least in principle, observable. This includes probabilities, which can be conceptualised as frequencies of an event in a sample of data (even if such data may in practice not be directly available to the expert). However, three guidelines argue that elicited quantities can be “unobservable” model parameters, such as odds ratios, provided they are well-defined and understood by the participating experts.

Parameters are here described as “unobservable” if they are complex functions of observable data, such as odds ratios. The guidelines list many types of quantities or parameters that can be elicited, including physical quantities, proportions, frequencies, probabilities, and odds ratios. They give few recommendations, though, aside from Cooke and Goossens^20^ recommending experts not be asked about uncertainty about probabilities, but that questions be reframed as uncertainty about frequencies in a large population. Choy et al. also recommends against eliciting probabilities directly^21^, but two other guidelines list it as a possible choice. *Chapter 7*further considers the possible types of quantities relevant for HCDM.

Three of the guidelines recommend formal processes for selecting what to elicit, and several guidelines describe principles the elicited quantities should adhere to. Principles discussed include that questions should be clear and well-defined with neutral wording, asked in a manner consistent with how experts express their knowledge, and only be elicited when the uncertainty impacts on the final model and/or decision.

Some SEE guidelines describe two issues related to the quantities to elicit: disaggregation and dependence. Five guidelines suggest disaggregating or decomposing a variable makes the questions clearer and the elicitation easier for experts. Five guidelines also discuss the importance of considering dependence between variables. When dependence is discussed, guidelines recommend reframing dependent items in terms of independent variables wherever possible. If dependence cannot be avoided, the elicitation task will be more complicated, but they recommend assessing conditional scenarios or using other elicitation framing and related techniques to estimate dependence.

### Section 3.5.2 Encoding judgements

In addition to choosing what questions to put to experts in an elicitation, analysts must also choose how questions will be put to experts. That is, how will experts be asked to assess their uncertainty about the unknown quantities?

Three guidelines--all agency documents--either do not discuss methods for encoding judgements at all^22, 23^ or offer no advice (i.e., neither recommendations nor a list of choices) on the matter.^24^ ***Error! Reference source not found.****, Table 4* summarises the recommendations and choices described by the other 13 guidelines.

Most approaches can be classified as either fixed or variable interval. Fixed interval techniques (discussed in 6 of the 16 guidelines) present experts with a specific set of ranges, and the experts provide the probability the quantify falls within that range. A popular fixed interval technique is the roulette or "chips and bins" method, in which experts construct histograms that represent their beliefs. Variable interval methods (recommended by five guidelines and discussed in another five), in contrast, give the experts set probabilities and ask for the corresponding values. Popular variable interval methods include the bisection and other quantile techniques. These methods are described further in *Chapter 8*.

Two guidelines recommend methods that cannot be classified as either fixed interval or variable interval. The Investigate, Discuss, Estimate and Aggregate (IDEA) protocol utilizes a combination approach, asking experts to provide a minimum, maximum, and best guess for each quantity as well as a "degree of belief" that reflects the probability the true value falls between the minimum and maximum. Experts may all provide assessments for different credible ranges, and the analyst standardizes them to an 80% or 90% credible interval using linear extrapolation.^25^

Kaplan’s method takes a very different approach.^19^ Rather than asking experts to encode their beliefs in a way that can be transformed or interpreted as a probability distribution, the method requires that experts only discuss evidence related to the quantity of interest, and then a facilitator creates a probability distribution that reflects the existing evidence and uncertainty.

In addition to the core encoding method, three guidelines also discuss that physical or visual aids can be used by the elicitor(s) to assist with the encoding process.^18, 26, 27^

Despite the variety of encoding methods discussed, none of the guidelines present empirical or anecdotal evidence or other justification for their recommendations or choices. *Chapter 8* provides new evidence relating to the choice of encoding method.

## Section 3.6 Identifying and selecting experts

Recommendations and choices related to identifying and selecting experts are summarised in ***Error! Reference source not found.****, Tables 5-6*. Only one guideline does not discuss the number of experts to include in an elicitation.^23^ The others either explicitly recommend or imply that judgements will be elicited from multiple experts. The range of how many experts should be included spans from 4 experts^20^ to 20^25^. The EPA White Paper is the only guideline that gives considerations beyond practical concerns for how many experts to include in an exercise. It observes that if opinions vary widely among experts, more experts may be needed. On the other hand, if the experts in a field are highly dependent (based on similar training or experiences, for example), adding more experts has limited value.^18^ The risk of dependence between experts is only discussed in three other guidelines.^22, 26, 28^

Most guidelines do not address how many facilitators or analysts should be involved in an elicitation. The few that do state that 2 or 2-3 facilitators is ideal, with the facilitators having different backgrounds or managing different tasks during the elicitation.^18, 20, 24, 29, 30^

Identifying and selecting experts is discussed in all but three guidelines.^19, 21, 28^ Recommendations from the other 13 guidelines overlap considerably. Common criteria relate to reputation in the field, relevant experience, the number and quality of publications, and the expert's willingness and availability to participate. Normative expertise is listed as desired by five guidelines, but three specify that it is not a requirement.

Five guidelines recommend all potential experts disclose a list of their personal and financial interests, often noting that interests should be recorded but will not automatically disqualify an expert from participating, as that may impose too extreme a limit on the pool of possible experts. Eight guidelines recommend the group of experts included in an elicitation should reflect the diversity of opinions and range of fields relevant to the elicitation topic. The agency guidelines tend to provide more details on identifying and selecting experts, with four describing optional procedures producing a longlist of possible experts that are then winnowed down based on agreed upon selection criteria. While many guidelines suggest identifying experts through peer nomination, Meyer and Booker^31^ cautions that this process can, if not well-managed, lead to issues related to experts only nominating other people with similar views. *Chapter 5* considers the broader literature on selecting and identifying experts.

## Section 3.7 Training and preparation

Recommendations and choices related to identifying and selecting experts are summarised in ***Error! Reference source not found.****, Table 7*. Nine guidelines either explicitly recommended piloting the elicitation protocol with a subject-matter expert not participating in the exercise or imply that piloting will be done. The remaining seven guidelines did not discuss piloting.

Only one guideline offered training as a choice^19^; the other 15 all requiring at least some form of training. Recommendations and suggestions for what should be included in expert training are largely consistent across the guidelines and cover issues related to elicitation generally and the subject-matter at hand specifically. Commonly recommended aspects of training include: an introduction to probability and uncertainty, an overview of the elicitation process, an introduction to heuristics and biases, the aim and motivation for the elicitation, information on how elicitation will be used, relevant background information, and details of any assumptions or definitions used in the elicitation. Four guidelines recommend using practice questions to ensure experts understand the elicitation process.

Most guidelines do not discuss what, if any, training should be provided to the elicitation facilitator(s) or other roles involved in conduction an elicitation. Five guidelines, including four generic guidelines, provide material meant to assist the facilitator, including sample text and forms.^17, 20, 25, 29, 31^

## Section 3.8 Conducting the elicitation

### Section 3.8.1 Mode and level of elicitation

Recommendations and choices about the mode of administration and level of elicitation (group or individual) are summarised in ***Error! Reference source not found.****, Table 8*.

Elicitations can be conducted in-person, in either individual interviews or group workshops, or remotely, via the internet, email, mail, phone, video conferencing, or other means. Nine guidelines recommend in-person elicitation, and only one recommends remote elicitation. Eight guidelines list remote elicitation as a choice, recognizing that it may be logistically easier to arrange than an in-person elicitation.

The mode of administration may be governed by whether a method elicits judgements from individual experts (i.e., each expert provides an individual assessment) or groups (i.e., a group of experts provides a single assessment). Of the 16 guidelines, only Choy et al.^21^ does not discuss the level of elicitation. Group-level elicitation is only recommended by Kaplan,^19^ who recommends a process where experts discuss the evidence relevant to an elicitation variable and then the facilitator proposes a probability distribution that matches the input provided by all of the experts. Individual-level elicitation is recommended by five guidelines, and two guidelines recommend a combination approach, wherein individual assessments are elicited first and then the group works to provide a communal assessment that reflects the diversity of opinion in the group. *Chapter 5* provides more detail on individual- versus group-level elicitation.

### Section 3.8.2 Feedback and revision

All but one guideline^31^ discussed the importance of feedback and revision, but three did not provide information on how it should be done.^23, 26, 27^ The other guidelines discuss a range of possible feedback methods, which can provide information on an individual’s judgements, the aggregated group judgements, or a summary of what the other experts provided. Recommendations and choices about the mode of administration and level of elicitation are summarised in the ***Error! Reference source not found.****, Table 9*.

Knol et al^27^ is the only guideline that warned of a possible negative impact of feedback and revision, cautioning that it can cause unwanted regression to the mean in the experts revised assessments. None of the guidelines recommend against providing feedback and opportunities for revision in any form. The feedback of group summary judgements is investigated in *Chapter 8*.

### Section 3.8.3 Interaction

Recommendations and choices regarding interaction and rationales are summarised in the ***Error! Reference source not found.****, Table 10*. Three guidelines did not explicitly discuss interaction between the experts.^20, 21, 32^ While no guidelines recommended avoiding interaction, seven say no interaction is a possible choice. Interaction is closely related to level of elicitation, with guidelines recommending group discussion prior to individual elicitation, group discussion prior and during a group elicitation, and group discussion following an individual elicitation. One guideline recommended interaction be limited to a remote, anonymous, facilitated process.^17^ Other guidelines also described these options as choices.

Although the guidelines disagreed about if and how interaction should be managed in an elicitation, many do present more justification for the recommendations or choices around interaction than they do for other methodological choices. The benefits of interaction between experts is that it minimizes the differences assessments that are due to different information or interpretation^27^ and allows analysts to explore correlation between experts.^28^ The drawbacks, though, are that it can allow strong personalities to carry too much weight^26-28^, the experts may feel pressure to reach a consensus^26^, there may be risk of confrontation^28^, and interaction can encourage groupthink, resulting in the experts being overconfident.^23^ Practical considerations can also guide the choice of if and how to include interaction, as individual interviews may take more time, but a group workshop may be more expensive.^27^ These issues are further discussed in *Chapter 5*.

### Section 3.8.4 Rationales

Only one guideline presented collecting the experts rationales during an elicitation as a choice rather than a recommendation.^31^ The other 15 guidelines all recommend collecting rationales because they help analysts and decision makers understand what an answer is based on^23, 26, 28^, provide a check of the internal consistency of an expert’s responses ^26^, record any assumptions^30^, and may help limit biases.^21^ The information collected in rationales can also be useful for peer review or for future updating of the judgements.^23^

One guideline also recommended collecting rationales from the decision maker about how they use the expert judgement results.^32^

## Section 3.9 Post-elicitation

### Section 3.9.1 Aggregation

Even when eliciting judgements from multiple experts, it can be important to have a single distribution that reflects the beliefs of the experts that can be used in modelling. Recommendations and choices on aggregation methods are summarised in ***Error! Reference source not found.****, Table 11*. Five guidelines presented aggregation as a choice, but the remaining 11 recommended aggregation always be done.

Aggregation can be behavioural or mathematical. In behavioural aggregation, experts interact with the goal of producing a single, consensus distribution. Mathematical aggregation involves the facilitator(s) eliciting individual assessments from the experts and then combining them into a single distribution through a mathematical process. Two guidelines recommend behavioural aggregation. Kaplan^19^ recommends a process that includes group-level elicitation and behavioural aggregation: the experts discuss the evidence relevant to an elicitation variable, the facilitator suggests a probability distribution that reflects the diversity of evidence on the subject, and then the process concludes when there is consensus from the experts about the proposed distribution. The SHELF method recommends an initial round of individual-level elicitations followed by expert discussion designed to produce a single distribution that represents how a "rational independent observer" would summarise the range of expert opinions.^29^

Four of the guidelines recommended variations on mathematical aggregation. Three guidelines recommended combining expert judgements in a linear opinion pool that equally weights all of the experts. Cooke and Goossens^20^ is the only guideline that recommends mathematical aggregation with differential weights for the experts. They suggested a method whereby the experts are scored and weighted according to their performance assessing a set of seed questions, which are items that are unknown to the expert but known to the facilitator.

Budnitz et al.^33^ recommend a unique approach wherein the analysts determine the aggregation method during an elicitation, based on an evaluation of how the process is unfolding and determining what is most appropriate. They recommend a behavioural aggregation-based consensus is the best choice, but believe it is not appropriate in all situations. The analysts can also decide to use mathematical aggregation with equal weights or analyst-determined weights or a process similar to that recommended by Kaplan,^19^ in which the analysts supply a distribution they believe captures the discussion and evidence presented by the experts.

Like interaction, several of the guidelines give more background to help guide an analyst in his or her choice of method. The main drawback of aggregation, according to Tredger et al.,^23^ is that it can lead to a result that no one believes. Two guidelines warn that the expert selection is of increased importance if an elicitation will use mathematical aggregation with an opinion pool, particularly equal weights, as increasing the number of experts with similar beliefs will result in those beliefs having more influence in the final, aggregated distribution.^24, 26^ Garthwaite et al.^26^ also suggest opinion pools may be problematic as they result does not represent any one person or group’s opinion, but Bayesian weighting requires a lot of information on the decision maker’s views of the experts opinions. Finally, several guidelines discuss that the possible issues around behavioural aggregation are linked to the challenge of properly managing group interactions, the topic discussed next. The broader literature on aggregation is discussed in *Chapter 5*.

### Section 3.9.2 Fit to distribution

Recommendations and choices on fitting to distribution are summarised in the ***Error! Reference source not found.****, Table 12*. Analysts can fit the elicited data to a probability distribution either as part of the elicitation or during post-elicitation analysis of the data. Possible choices, discussed in about half of the guidelines, include fitting to a parametric distribution, using non-parametric approaches, or just use the information directly elicited from the experts.

None of the guidelines recommended specific distributions be used in fitting, but they say the analysts should choose based on the nature of the elicited quantity and the information provided by the experts. Cooke and Goossens^20^ describe probabilistic inversion, a method that can be done if the observable elicited variable needs to be transformed into a distribution on an unobservable model parameter. *Chapter 5* explores issues of fitting judgements to distributions in more detail.

### Section 3.9.3 Other post-elicitation components

Recommendations and choices related to the other post-elicitation components are summarised in the ***Error! Reference source not found.****, Table 13*. Only 2 guidelines discussed obtaining feedback from the experts on the elicitation process. Walls and Quigley recommended analysts ask experts what could have been done differently if new data is later collected that differs from the experts judgements.^28^ The EFSA Delphi recommended that analysts give experts a questionnaire with the opportunity to provide general comments on the elicitation questions and process.

None of the guidelines recommended that analysts should adjust experts assessments, but five describe related choices, such as manually adjusting assessments, dropping an expert from the panel, or adjusting assessments to be more accurate, which is recommended against by two guidelines.

Documenting the elicitation process and results is the only elicitation element discussed by all 16 guidelines. Although the specific recommendations regarding what to include in the final documentation varies across the guidelines, they do not conflict. The guidelines typically recommend documentation include the elicitation questions, experts individual (if elicited) and aggregated responses, experts rationales, and a detailed description of the procedures and design of the elicitation, including the reasoning behind any methodological decision. Many of the agency guidelines are more prescriptive about what documentation should entail, and some provide detailed templates.^17, 18, 32^

## Section 3.10 Managing heuristics and biases

Expert judgements are affected by a variety of heuristics and biases.^34, 35^ Morgan^36^ argues that these biases cannot be completely eliminated, but that the elicitation process is designed to minimize their influence on the results. The 16 reviewed guidelines discussed 11 different cognitive biases and 8 motivational biases that can affect an elicitation. A list of the biases discussed and possible actions to minimize them can be found in ***Error! Reference source not found.****, Table 14*.

Most of the bias-reducing actions mentioned by SEE guidelines are only discussed in one or two, but the actions do not conflict with one another. The most frequently recommended actions are to frame questions in a way that minimizes biases (discussed in five guidelines) and to ask for the upper and lower bound first, to avoid anchoring (discussed in three guidelines). While most guidelines offer some recommendations for mitigating and managing biases, they present little to no empirical evidence to support that their recommended actions have the intended effect. The broader literature on heuristics and biases is reviewed in *Chapter 6*.

## Section 3.11 Considering the validity of the process and results

Four guidelines do not discuss how to ensure the validity of elicited results^17, 19, 29, 31^, and the other 12 guidelines present a range of perspectives on what is meant by validity, summarised in ***Error! Reference source not found.****, Table 15*. Validity can mean that the exercise captured what the experts believe (even if that is later proven false).^26^ It can also refer to whether the expressed quantities correspond to reality^20, 25, 26, 28^, are consistent with the laws of probability^26, 28^, or are internally consistent.^22, 27^ Some guidelines—all agency documents—also view validity as mostly concerned with the process rather than the results and suggest an elicitation is valid if it has been subjected to peer review.^18, 24, 32^ Recommendations and choices for handling validity differ across the guidelines and can involve actions at any stage of the elicitation process, depending on what definition of validity the guideline seeks to achieve.

## Section 3.12 Conclusions

The SEE guideline review reveals a developing body of work designed to guide elicitation practice. Although the guidelines evolved separately in different fields, they largely agree on issues around what quantities to elicit, expert selection, the importance of piloting the exercise and training experts, face-to-face elicitation being preferable to remote modes, the importance of collecting rationales from the experts alongside the quantitative assessments, fitting assessments to distributions, the key role documentation plays in supporting and communicating an elicitation exercise, and how to manage heuristics and biases. The guidelines recommend different approaches for encoding judgements, using individual- or group-level elicitation, aggregating judgements, and managing interaction between the experts. Although the guidelines agree that validation is important, they disagree on what actions an analyst can take to encourage or demonstrate validity. Finally, some areas seem under-discussed. Dependence between questions, for example, is a complicated issue that could be critically important when interpreting elicitation results, but little guidance exits on the topic.

The elicitation choices identified in this review are further considered in *Chapters Chapter 5, Chapter 6, Chapter 7* and *Chapter 8*, and their suitability for use in the HCDM context is evaluated in *Chapter 9*.

# Chapter 4 Expert elicitation in different decision making contexts

## Section 4.1 Introduction

Challenges in the conduct of SEE in HCDM are discussed in *Chapter 4*. The challenges identified in the applied examples were largely practical and related to the design of the SEE for that particular task. There are, however, much broader challenges and opportunities, which relate to the decision making context in which SEE is applied. These issues are discussed in this chapter.

The specificities of the context in which expert elicitation is conducted should be distinct from the principles and methods employed. That is, best practice should always be regarded as an appropriate starting point regardless of the context. Doing so, however, may ignore many important factors which influence the choice of method employed for an elicitation. A reference protocol which does not at least consider context specific constraints, is unlikely to be used widely or may be only restricted to a sub-set of decision makers, perhaps those operating at a national level.

In considering how a reference protocol for expert elicitation in HCDM might be utilised in practice, it is important to understand how different decision making contexts may influence the requirements for and practicalities of, expert elicitation. In particular there may be practical constraints in certain contexts that imply the use of a second best methodology. Some of these issues are explored in the evaluation (see *Chapter 11*), however this chapter considers the range of decision making contexts more generally, highlights the potential constraints and the implications for SEE methodology. Given the lack of experience with SEE in formal decision making processes, a formally review of the challenges and constraints faced by different HCDM’s is unlikely to be informative. Instead this chapter is intended as a discussion, rather than a formal review. It draws on observations and experiences of the project team and the wider advisory group.

## Section 4.2 Levels of decision making

In England reimbursement decision making bodies can be described at three levels, implying the population they serve and the jurisdiction for their decision making activities.^37^ These are:

1. Individual practitioners, such as General Practitioners (GPs), secondary care clinicians and local decision makers, such as Clinical Commissioning Groups (CCGs), Local Authorities (LAs) and hospital trusts
2. National decision makers, such as NICE, Department of Health and Social Car (DSHC), NHS England, Public Health England
3. Research commissioners, including organisations such as NIHR and the Medical Research Council (MRC) but also industry sponsors of research.

Reimbursement bodies range from local practitioners and commissioners to national decision makers (2). Here individual and local decision makers (1) are grouped together as many of the constraints are relevant in both contexts. In addition, there are multiple organisations which commission research (3), potentially including SEE, these can also be regarded as decision makers.

### Section 4.2.1 Individual practitioners and local ‘population level’ decision makers

#### Section 4.2.1.1 Features

There are a number of decisions that are made on an individual practitioner – patient level in the NHS and other health care systems. These usually concern a patient’s course of treatment and the most effective, and sometimes cost-effective, choice given the particular circumstances. Such decisions are made in a primary care setting, usually involving a GP, and a secondary care setting, usually involving a consultant or other medical specialist. Such decision makers may also make choices for groups of patients, for example in deciding which device to purchase within a hospital or organising surgical lists.

HCDM occurs at a population level in several forms. In England within primary care, a CCG (see below) supports decision making between GPs and their patients through local guidance such as the referral support system (RSS)[see ^38^ for an example]. This may also extend to services offered within secondary care, e.g. referrals for further testing/investigations. For both primary and secondary care there may be relevant guidance produced by NICE to support decision making. Individual practitioners and secondary care clinicians are also influenced by their professional bodies/councils. NHS England, as well as commissioning for primary care, also produces key strategic guidance for CCGs to support them to fulfil their duties to their respective populations. So, although individual health care professionals in the NHS make decisions about individual patients, this is very much governed by the organisations that are intended to harmonise provision of services across England and encourage best and most cost-effective provision of services.

The NHS also works closely with social services, and individual practitioners in this respect include social workers, residential care homes and carers. Within public health, services are delivered across the NHS, social care and local authorities (LAs) and many are supported by the work of Public Health England (PHE). Public health practitioners, including nutritionists, smoking cessation coordinators, and teenage pregnancy coordinators, are again employed by the NHS, PHE, NHS England and individual LAs and CCGs, and as such work within their codes of practice and adhere to appropriate guidance regarding provision of services. LA Public Health is also accountable to Public Health England.^37^

#### Section 4.2.1.2 Constraints

The particular constraints in these context relate to the degree of autonomy that individual practitioners/CCGs have in making decisions regarding individual patients or groups of patients within their jurisdiction. Since the abolition of GP fund holding (in 1997/98)^39^ and subsequent changes to commissioning before the 2014 care act, individual practitioners are more constrained with regards to patient level decision making.

In both CCGs and LAs there are significant budget constraints. Whilst the average CCG’s budget grew by 3.4% in 2016/2017^40^ there are a number of new pressures on CCGs which require them to cutback or reorganise local services. These include cutbacks to public health and social care funding. Post the first two years of the move of public health to LAs there are services which LAs may be forced to reduce investment in, some of which have implications for public health.^41^

CCGs and LA are also constrained by budget cycles, which are typically 1-3 years. There may be an incentive to replace activities that cannot prove ‘value’ within these time frames with those that have a higher immediate payoff, for example less investment on prevention.

Both CCGs and LAs face multiple competing demands for money and resources. There are big differences across regions with regards to commissioning of and participation in research. Some CCGs and LAs work with health economists and are therefore directly involved in either commissioning participating in or understanding the results of cost-effectiveness evidence and the implications for their population. Others do not have access to such resources.

#### Section 4.2.1.3 Implications for expert elicitation in this context

We are not aware of any examples where formal SEE has been used to support decision making of the system, although of course, judgement is used routinely in the clinical and management settings every day. (This does not preclude that such elicitations have not been done, of course, but if so, they have not been documented.) Indeed, it might initially seem practically unfeasible to use SEE to support decision-making at the individual practitioner level, particularly given that many decisions are made on a national level with implementation at a local level. However, there are still a number of decisions that can be made by individual practitioners or groups of practitioners/local commissioners, many of which may rely on assumptions and opinion rather than experimental data. For example in considering individual cases and episodes of care, such as for procedures and services not routinely funded by the NHS, an individual funding request (IFR) panel, will consider specific cases for reimbursement, for example cosmetic services.^42^ Those conducting SEE to inform other decision making process may also reply on individual practitioners to act as experts. In some circumstances the SEE may be required to consider parameters for a specific patents rather than at a population level, for example in the IFR process. This can have implications for how a SEE is designed, specifically elicitation of uncertain and communication with experts on how to express their uncertainty.

SEE undertaken in this context must also adapt to the practical constraints, in particular it may not be possible to invest significant amounts of time and resource into SEE and availability of experts to inform often practice level decision making maybe be limited. Such experts are unlikely to possess any normative skills or have any experience with SEE. Group based SEE may be a challenge in this context, as well as individual SEE which requires face-to-face interaction. It may be necessary to trade off recruiting large numbers of experts for face-to-face SEE with obtaining larger numbers through a remote SEE.

### Section 4.2.2 National decision makers

#### Section 4.2.2.1 Features

In England the DHSC governs health and social care matters and has responsibility for some elements not covered separately by Scottish, Welsh or Northern Irish governments. ^37^ The DSHC itself takes responsibility for a number of services and activities provided by the NHS, and are also supported by a number of agencies and public bodies. The DSHC provides a mandate to NHS England to help guide its decisions regarding the allocation of resources, commission specialist services and its strategic direction. NHS England oversees commissioning aided by four regional offices. It has responsibility for commissioning contracts for GPs, pharmacists, and dentists and supporting CCGs in their commissioning roles.

There are a number of special health authorities and other bodies which are either part of the NHS or closely associated with it. They include the NICE and the Prescription Pricing Authority. These organisations are either accountable to the Secretary of State, or have formal agreements with the DHSC. In general they provide national services. NICE was set up in 1999 as a special health authority.^43^ Officially NICE only has jurisdiction in England and is supported in considering its guidance in Scotland and Wales by the Scottish Medicines Consortium (SMC) and the All Wales Medicines Strategy Group (AWMSG) respectively. NICE provides guidance on a range of healthcare products and services, including: pharmaceuticals, diagnostics, medical devices and public health interventions. In compiling evidence to generate these guidance it often relies on the use of expert opinion in some form. A review of practices relating to the use of evidence elicited from experts across NICE guidance-making programmes, was recently published^44^ and concluded that “NICE uses expert judgement across all its guidance-making programmes, but its uses vary considerably”. In addition it agreed that “there is no currently available tool for expert elicitation suitable for use by NICE”.

Working alongside NICE on public health issues are PHE, which was formed in 2013 and took over the role of a number of other health bodies, including the Health Protection Agency (HPA).^45^ PHE generates and interprets evidence and therefore there is potential for it to utilise SEE. Like the Public Health Programme at NICE, the evidence base it considers is more likely to be low quality and/or sparse, and therefore the opportunities for SEE may be significant

#### Section 4.2.2.2 Constraints

The likes of the DSHC, NICE and PHE are required to make decisions about reimbursement, best practice and access across the whole of their population. Therefore decisions have to be relevant across different, perhaps heterogeneous, populations.

The separation of research commissioning and reimbursement can also generate complexities. Decisions may be reached on the basis that further data collection may be required, however some national decision makers do not commission their own research and therefore cannot ensure that data collection takes place and/or addresses the uncertainties identified.

As with more regional decision making, national decision making is also subject to the constraints of time and resources. Although not necessarily as constrained as local commissioning cycles dictate, national decision makers do still have to generate guidance within acceptable timescales. The process of generating guidance through the NICE single technology appraisal process (STA)^45^ can take around 6-months including committee meetings. Despite the fairly rapid timescales, formal decision making processes, particularly those which imply mandatory implementation of guidance, such as the NICE technology appraisals process, require full accountability for decisions reached. The need to make decisions in a timely manner therefore cannot compromise the quality of the deliberations used to make these decisions, including any evidence generation that contributes towards this.

#### Section 4.2.2.3 Implications for expert elicitation in this context

Historically, SEE has been commissioned to support policy challenges. For example, policy on surgical equipment sterilisation to protect against the risk of new variant Creutzfeld-Jakob disease (nvCJD) prion transfer has been informed by SEE in the wake of the Bovine Spongiform Encephalophy (BSE) crisis in the UK.^46^ More recently, the European Commission commissioned SEE studies of the future antibiotic resistance (AMR) rates in four European Countries to inform policy and the UK Department of Health and Social Care is currently commissioning additional UK-focussed work in this area.^47^ Across national decision makers, the quality of evidence to inform decisions is quite heterogeneous. This can be at various stages of maturity and in some areas, for example public health, evidence may not be particularly robust. SEE could be useful to help inform decisions in these situations, although it is likely that some of the parameters required may also be difficult for experts to make judgements about, for example population uptake of a screening programme.

Indeed many examples of SEE conducted in the area of HCDM have been undertaken to inform national decision making organisations such as NICE (see *Chapter 4*). As a result there is a degree of familiarity with the approaches used and an acceptance of its limitations. NICE only makes brief reference to the use of expert opinion to generate evidence in its guide to the methods of technology appraisal.^45^ NICE do not suggest a preferred methodology for this and they have not used any consistent criteria to judge SEE submitted as part of any appraisal process.

It is true that decision makers have differing capacities to undertake SEE, specifically in reference to resourcing of SEE. Evidence generation does not constitute a significant proportion of the remit for some decision makers. Therefore, similarly to the use of SEE in local decision making, SEE undertaken in this context must adapt to the practical constraints. Timescales for evaluation are often tight and there are implications of any delay in approving a technology or service. Although SEE takes significantly less time than many other forms of empirical evidence to collect, if conducted appropriately the time resource can still be unachievable in some instances. Political cycles can generate promises around improving efficiency and accesses to NHS services. Tight turnaround for evidence to support these promises can negate the ability to undertake SEE and in this instance less formal approaches to filling data gaps may be employed.

In terms of specifics, as discussed above, decisions may have to be relevant for potentially heterogeneous populations. Eliciting uncertainty around a measure of central tendency across a heterogeneous population, can be a challenge for experts. Rather than eliciting across the entire population it may be advantageous to express quantities for multiple patient types, which will increase the size of the SEE task.

### Section 4.2.3 Research commissioners

#### Section 4.2.3.1 Features

In addition to those discussed above, there are also other decision makers not concerned with reimbursement, such as HTA, NIHR more generally and the MRC. These bodies commission research and use expert opinion in cost-effectiveness analyses and therefore any guidance on appropriate design and conduct of SEE would have implications for their practices. Industry can also commission research as part of the license and reimbursement processes.

Such decision makers, typically do not fund interventions *per se*. but instead commission effectiveness and cost-effectiveness research across their respective areas of interest. Many of these could potentially use SEE to help inform their decisions regarding which research to fund and the specific form that this research might take. One example is the use of SEE in determining sample size calculations for clinical studies.^48^ Here the SHELF framework^15^ has been used to generate prior beliefs to aid clinical study design, specifically on the probability of success (assurance parameter).

#### Section 4.2.3.2 Constraints

The scale of the commissioning of research varies across funders and within their programmes of work. Some funders are constrained to commission research with a specific area, for example clinical speciality, whereas other, such as the National Institute for Health Research (NIHR) and the MRC commission across a range of topic areas. SEE used outside of the context of a decision making (reimbursement) process may not be subject to the same constraints in terms of time or resources, however *Chapter 4* does not identify any applied examples where SEE has been the sole purpose of the research, instead SEE is likely to constitute only a small proportion of the research funding.

#### Section 4.2.3.3 Implications for expert elicitation in this context

As the rationale for the research is to reduce uncertainty, and as research priorities are inevitably contentious there seems to be a very strong case for using SEE in this context to focus research. For example, Dallow et al discuss several examples of the use of expert elicitation at GlaxoSmithcline to inform trial design and the management of the company’s research portfolio.^48^ In a similar vein, Walley et al describe a case study of Pfizer where elicitation was used.^49^ Given that research commissioners tend to focus on particular specialities, for example clinical areas, it may also be possible to generate a level of expertise to undertake SEE, both in terms of the analyst and the experts. Where it has been used in the clinical trial setting, to inform sample size calculations, an expert panel has been establish to speed up the generation of experts priors in subsequent SEE’s.

The lack of consistency between research commissioners presents a challenge for the application of SEE in this context. Not all commission cost-effectiveness studies and there is also diversity in topics which may have implications for the way in which SEE is conducted. Public Health and complex interventions, for example vaccinations programs or other non-pharmacological interventions such as service changes, may imply different methods for SEE compared to medicines (see *Chapter 10*).

## Section 4.3 Conclusions

SEE can, in principle, be applied in many different settings, across a range of types of decision makers. In practice, to date, its application has largely been restricted to informing national level HTA decisions and for the purposes of generating evidence as part of larger research projects (see *Chapter 4*). The lack of SEE at an individual practitioner and local population level is likely to be driven by resource and time constraints and the fact that constant changes to policy making at local/national level can also shift the focus on a frequent basis. One solution is to move away from the use of SEE as an ‘addition to the analysts’ toolkit’ and instead as a substitutive for other forms of evidence, for example, a systematic review or modelling exercise. This is likely to be a challenge in systems that have relied heavily on such forms of evidence to inform decision making, but may be more feasible in local decision-making settings.

Guidance on appropriate conduct of SEE in HCDM is likely to be useful in all the contexts discussed, however time constraints and lack of capacity to conduct such exercises is likely to remain a challenge, where SEE is forced to fit into existing processes. For this reason SEE is most likely to gain traction in national and multinational settings, where a capacity for such activities can be generated, simply though economies of scale.

Even within national and multi-national decisions making process, there are likely to be different challenges in conducting SEE and some of these may imply that methodological choices need to be adapted to suit that particular application. Such issues are discussed in *Chapter 10*.

# Chapter 5 Challenges in structured elicitation in HCDM

## Section 5.1 Introduction

Reimbursement decisions in health are often supported by model based economic evaluation (MBEE).^50^ There may be circumstances in which SEE is required to address data limitations in MBEEs, such as short time horizon or missing entirely.

A review of applications in this area, published in 2013,^44^ identified only a small number (14) of studies reporting the use of SEE. This review did not seek to determine the reasons for heterogeneity of approach, nor did it look at the challenges faced when conducting SEE to support MBEE in health, and inform directions for future research. In pursuit of further clarity, instead focusses on summarising the basis for methodological choices made in each application (design, conduct, and analysis) and the difficulties and challenges reported by the authors. Further details of this review are reported elsewhere^1^ and so only a summary has been presented in this chapter.

## Section 5.2 Methods

To identify applications of SEE, the 2013 review^44^ was updated (identifying studies up to April 11, 2017). Further details on the methods of the search are given elsewhere.^1^ Studies were included only if they contained an SEE to elicit uncertain parameters (in the form of a distribution) to inform MBEE in health.

The methods used in each application were extracted along with the criteria used to support methodological and practical choices and any issues or challenges discussed in the text. Issues and challenges were extracted using an open field, and then categorised and grouped for reporting.

## Section 5.3 Aspects related to the design of the SEE

In existing applications, experts beliefs were sought for only a few parameters of a decision model, often not elicited directly but calculated from one or more alternative elicited quantities. Quantities included: event probabilities, relative effectiveness, time to event and diagnostic accuracy (see Soares, et al^1^ for full details). The choice of which quantities to elicit was based on a number of criteria. The first was appropriateness for experts, specifically that parameters in decision models can be complex and may not be directly observable by experts. Second are statistical concerns. The quantities elicited should be fit-for-purpose for further analysis, for example allowing elicited evidence to be combined with any existing empirical evidence, statistically coherent and reflect any dependencies between the quantities elicited. Finally the burden to experts need to be considered. Burden can be reduced by, for example, limiting the number of target parameters to elicit or eliciting homogeneous quantities throughout the exercise.

Almost exclusively, applications have recruited health care professionals, based on the following criteria: recognition by peers,^51^ specialist knowledge or clinical experience,^52, 51,^ ^53,^ ^54,^ ^55,^ ^56,^ ^57^ based in the relevant jurisdiction,^52,^ ^51,^ ^54,55^ research experience,^51,^ ^56,^ ^57^ and lack of involvement in product development.^53^ A number of authors ^52,^ ^58,59^ recognised that health care professionals are unlikely to have knowledge of elicitation and may have only sparse quantitative skills. This has driven choices made in designing and conducting the SEE, such as training needs, method of elicitation and definition of the quantities to elicit.^52^

Many of the applications have included a varied sample of experts by recruiting them from a range of relevant specialties,^51,^ ^60,^ ^61^ clinical settings^52,^ ^51,61^ and geographical areas/countries^51,^ ^57^ to capture heterogeneity in beliefs (reflecting underlying heterogeneity in patient populations), and avoid dependency between experts.^51^ The potential for bias in expert opinion was recognised in some SEE^52,^ ^56^ with reported attempts to minimize bias in the design.^62^ Two applications make explicit efforts to avoid recruiting experts that may have motivational biases.^53,61^ Two studies provided information on cognitive biases in the training session.^52,59^

In eliciting uncertainty, applications have typically used either the FIM ^52,51,^ ^63,^ ^60,54,^ ^55,^ ^59,^ ^64,^ ^62,^ ^65^ or VIM. ^53,58,66,^ ^67,56,^ ^57,^ ^68^ Choices were justified on the basis of: pilot exercises designed for the purpose (see below), generic methods research, previous use in MBEE, and claims of lower burden or intuitiveness for experts.

Fourteen studies elicit individually from experts and aggregate mathematically, three aimed to achieve consensus amongst experts^69,66,^ ^67^ and three others did not explicitly report the method of aggregation used.^70,^ ^61,64^ None of the three studies using consensus was explicit about the reasons for choosing consensus or the process of achieving it. Authors justify the choice of mathematical aggregation based on the desirability to reflect variation within and between experts^60^, because consensus is known to lead to overconfident results (i.e., narrow distributions)^51^ and because it raises practical difficulties of convening experts and providing experienced facilitation. With regards to weighting in the mathematical approach, most of the applications reviewed claim insufficient justification for generating differential weights^52,^ ^51^ and lack of clarity on how to appropriately generate the weights^52,^ ^53,^ ^62^ and hence apply equal weighting. Five studies, however, explored unequal weighting, either based on responses to seed questions^52,^ ^54,57,^ ^59^ (performance-based weighting) or using the clinical background of experts(objective weighting).^53^

## Section 5.4 Experiences with the conduct of the exercise

No studies reported major challenges in the conduct of the SEE, despite the complexity of the task.

Some conducted a group based session, which were typically face-to-face, although studies^51,^ ^60,^ ^58,^ ^62^ departed from this format due to time constraints, geographical limitations and availability of experts. Mathematical exercises adopted a mix of formats, ranging from individual interviews to remote completion via email. Administration, were details were specified was via bespoke tools using Excel,^52,^ ^51,54,^ ^55,59,^ ^62^ paper questionnaires, a generic elicitation package(the Sheffield Elicitation Framework or SHELF)^61,^ ^67^ and a software package for the elicitation of dependency (Prior Elicitation Graphical Software, PEGS).^58^ Some exercises were explicit about piloting the tool to ensure clear wording of the questions,^52,53,^ ^55,56^ and most offered opportunities for revision and/or graphical feedback.

Five applications were explicit about training of experts^51,^ ^60,^ ^54,^ ^59^ covering: overview of the project and of the role of elicitation;^52,^ ^60,^ ^53,^ ^54,^ ^59^ quantities required and definitions;^52,60,^ ^54,^ ^59^ explanation and expression of uncertainty;^51,^ ^53^ consideration of potential biases;^52,^ ^54,^ ^59^ use of the elicitation instrument^52^ and delivery of practice exercises.^51,^ ^60,54,59^ Studies that implemented elicitation remotely generally included some form of instructions, although none reported these in detail.

## Section 5.5 Experiences with the analyses and interpretation of elicited evidence

Studies did not report details sufficiently, however validity was assessed according to: missingness, validity checks and self-reported face-validity. Some applications requested feedback from experts on: the ease of completion of the SEE,^52,^ ^51,^ ^59,62^ the basis for experts answers (to reveal the sources of evidence considered by the experts and their level of knowledge),^51^ or on self-reported face validity.^52,^ ^51,^ ^60,^ ^59^

Of the 14 studies that used a mathematical approach to aggregation, one did not generate a group estimate and instead used the responses of each expert individually.^58^ The majority linearly pooled, by averaging individual distributions. In order to pool, some applications were not explicit about how prior distributions were derived from elicited summaries. Those that were explicit used parametric distributions, with the choice of distribution either not justified or based on general MBEE literature on distribution choice for probabilistic sensitivity analyses. ^51^ To fit the distribution some applications used software^63,^ ^53^ and others a specific fitting method, such as maximum likelihood fitting.

In some applications elicited evidence was used directly as input to a cost-effectiveness model.^51,^ ^60,^ ^58,^ ^69,^ ^71,^ ^59^ Where external evidence existed on elicited parameters, some authors present both sources separately using scenarios,^60,^ ^70^ while others combine them using Bayesian updating. ^52,70,67^ Three authors^63,^ ^58,^ ^55^ explored use of individual experts beliefs and found that results and associated allocation decisions varied between experts.

## Section 5.6 Conclusions

This critical review demonstrates that reporting is poor (as also identified elsewhere),^72^ and there is a lack of consensus on methodology. A number of principles from the elicitation literature are expected to generalise to the MBEE setting, such as the need for piloting and training; however, for many other areas of SEE, it is not clear that methods used in other disciplines translate to HTA.

## Section 5.7 Discussion

The review highlights a number of specificities/constraints that can shape the development of guidance and target future research efforts in this area. Firstly, there exists important between-expert variation. In other disciplines, variation is generally linked to different levels of bias and hence regarded as undesirable, warranting the use of strategies to reduce or discourage variation, such as consensus methods. The majority of applications in MBEE, however, expect wide variation in the beliefs of multiple experts due to genuine heterogeneity in the populations’ experts draw upon.

Secondly, substantive experts in HTA are health professionals who may not be trained in quantitative subjects, unlike other areas of science in which elicitation is used such as engineering or meteorology. Further research on SEE should consider the appropriateness of alternative methods of elicitation (e.g. chips and bins, or bisection method) for the potentially less normative experts, or on how to facilitate the elicitation of complex parameters, including dependency. Some of the applied examples seek for assurance on the validity of the particular exercise. It is, however, not clear how such an assessment should proceed. Examples have used self-reported face-validity assessments, sensitivity analyses, and performance weighting (calibration). Particularly for performance weighting, despite a growing (generic) literature discussing the validity of this approach (see for example),^73,74,75^ the applied literature struggles with supporting the methodological choices that need to be made.

Finally, while it is generally agreed that SEE should be designed and conducted in a way that minimises the use of heuristics and other sources of bias, there is little integration in the applied literature of the findings from behavioural research. A recent review placing special emphasis on debiasing techniques^76^ is a helpful resource to be reflected in future research.

# Chapter 6 Reviewing the evidence: expert selection, level of elicitation, fitting and pooling

## Section 6.1 Introduction

Each element of a SEE process encompasses several possible components for which choices need to be made, with each choice successively impacting on the next. If inappropriate choices are made for some component, the process can provide inaccurate or misleading judgements. Despite this risk, there is little or no empirical evidence which compares many of the alternative choices available when designing, conducting and analysing a SEE.

The aim of this chapter was to review the SEE literature to identify the possible choices and related evidence available in four components of the SEE process. Following discussion with the advisory group, the following four were chosen:

1. Selection of experts
2. Level of elicitation
3. Fitting and aggregation
4. Assessing the expected accuracy of experts judgements

## Section 6.2 Identifying literature

The use of SEE in HCDM and cost-effectiveness modelling is still evolving. Considering this, literature from both a cost-effectiveness context and other disciplines are reviewed in this chapter. While this chapter is not a systematic review, a semi-structured approach was employed to identify the relevant SEE literature and to summarise existing evidence. The targeted searches for the SEE elements listed above were conducted using the same semi-structured approach.

Literature was initially searched by reading a selection of well-known books and papers addressing SEE.^8, 14, 31, 77^ This approach defined each component and identified the associated choices available for each one. Following this, more recent literature on SEE was then explored to investigate the availability of any recommended choices based on sound principles or evidence for the identified components.^33, 44, 58, 59^ The guidelines reviewed in *Chapter 2* were also included if they provided evidence. Papers that were specific to the four components were also included in the review.) For applications of elicitation in the context of HCDM, research from Leal et al (2007),^51^ Bojke et al (2010) ^54^ and Soares et al (2011)^52^ were consulted, along with a review of elicitation methods in cost-effectiveness analysis.^1^

Each section describes the requirements for each part of the process, identifies the choices available and presents any evidence or principles to inform the choices. In *Chapter 9*, choices recommended in this chapter are considered against a set of principles underpinning elicitation in HCDM.

## Section 6.3 Selection of experts

An ‘expert’ is defined as someone who has great knowledge of the subject domain^8^ and who is competent in the practical application of this knowledge.

The SEE literature recognises several choices regarding to expert selection, relating to;

1. *Finding experts with the relevant skills*. The literature recommends that an expert possesses substantive skills in the target domain and normative skills regarding the expression of uncertain probabilities.^44, 59^ Ideally the expert will have specific knowledge in the field, together with a broad perspective.^59^ Where evidence is less developed, the expert will require adaptive skills to elicit judgements.^8^
2. *Measuring competencies in these skills*. Substantive skills can be identified and measured using social indicators of expertise or peer- and self-assessment tools, such as the Generalised Expertise Measurement (GEM)^14^ and the Expert-Selection Questionnaire (E-SQ),^92^ respectively. As normative expertise is more generic, the quality of probability judgement can be measured against seed questions.^14, 54, 59^
3. *Identifying experts*. The elicitation literature reports the following criteria for sourcing possible experts: recognition by peers,^51^ specialist knowledge or clinical experience,^51-57^ current work in the target domain,^51-54^ research output^51, 56, 57^ and lack of involvement in the product of interest. ^53^
4. *Recruiting experts*. Literature reports recruiting experts with a formal nomination process to develop a long-list of potential experts.^20, 22, 93^ More recent literature describes the use of a profile matrix listing the essential and desirable characteristics that are required. ^94^
5. *Identifying the optimal number of experts*. The analyst needs to decide whether to recruit multiple experts, and if so, how many, or one ‘top’ expert.

In the literature, substantive and normative skills are the most frequently referenced skills while the role of adaptive skills is not addressed as often. We think that adaptive skills can be of particular importance in a HCDM context where experts may need to elicit judgements on new or emerging technologies in which they do not have a great deal of experience.

There are concerns in the literature regarding the methods by which experts skills can be measured, with one source branding these measures as subjective, particularly when referring to social indicators of expertise.^80^ Peer and self-assessment tools have also been queried in terms of how accurately they measure substantive expertise.^52, 57, 59^ When measuring normative skills, selecting appropriate seed questions is difficult to assess (see Section 5.6). Until the relative advantages of the different methods for identifying and measuring substantive and normative skills are better understood, no technique in particular can be recommended.

The SEE literature suggests the recruitment of experts will be largely influenced by the target domain. In the healthcare literature, recruited experts are largely clinicians or professionals practicing in the target domain.^44, 51, 52, 54, 59, 95^ The recruitment strategies summarised above come from outside a HCDM context. Consequently, their applicability to elicitation in healthcare may not be appropriate.

There are not consistent recommendations on sourcing and recruiting experts, possibly because strategies are domain-dependent making it difficult to find a strategy that is appropriate across different contexts. Some of the processes of expert recruitment reported in literature, such as research output, can be interpreted as social indicators of expertise. These indicators have been subject to critique (Burgman reference) and should be treated with caution if used for expert selection.

Many reported influential factors can impact the optimal number of experts to recruit, such as the number of experts available with the relevant expertise,^51^ time and budget constraints and mode of administration of the SEE process.^56, 59^ Smaller samples are recommended for face-to-face modes of administration. The SHELF and Classical methods are optimally conducted with between five to ten experts as there are diminishing returns to accuracy improvement with more experts.^94^ In contrast, larger sample sizes are possible using remote methods of administration, as done in the Delphi method.^96^ When determining the optimal number of experts, heterogeneity amongst the experts also needs to be accounted for. It is logical to think that in a HCDM context, particularly for a new intervention or unknown condition, the pool of available experts will be limited. Despite the fact that SEE is less costly than primary data collection, the financial and time resources that are available for the design, conduct and analysis will dictate the number of experts that should be recruited.

Selecting the optimal number of experts is one of the few components of expert recruitment that have been studied empirically. Budescu and Chen (2015)^33^ assessed the benefits of adding additional experts, concluding that the best performance is found using between three to 16 experts, with around six being optimal. This assumes that all experts perform to the best of their ability. If there is a redundancy in expertise, the number of experts will need to be greater than six.^33^

## Section 6.4 Level of elicitation (individual versus group)

Generally, judgements from multiple experts will be sought in the SEE process. These judgements can be elicited individually from experts or from a group of experts.

When choosing the level of elicitation, the SEE literature suggests the analyst will need to consider the following choices and their associated principles;

1. The expert may provide their own judgement individually without interacting with other experts and this is referred to as *individual* level elicitation.^17^
2. Alternatively, experts are encouraged to interact with one another in a group to discuss the uncertain quantity until they achieve consensus.^8^ This is called *group* level elicitation.
3. A third approach uses a *combination of individual and group level* elicitation where experts first provide judgements individually and then engage in a facilitated face-to-face group discussion until they reach a consensus(SHELF method).^15^ This approach can also be conducted remotely using an iterative survey with several rounds of elicitation where each expert has access to the opinion of others through a highly restricted level of interaction (Delphi method).^17, 97^ On receiving the new information from peers, experts are given the opportunity to reach consensus using this remote method.

Figure 4 presents these different levels of expert elicitation describing their level of interaction, consensus and how uncertainty is quantified.

Levels of Elicitation

GROUP

DELPHI

INDIVIDUAL

Highly restricted interaction

No interaction

INTERACTION

High level of facilitated interaction

Opportunity for remote consensus as experts review opinions in the face of new information from peers

No opportunity for consensus. Experts elicit judgement individually

Following discussion of individual elicitations, face-to-face consensus

CONSENSUS

**Figure 4 Levels of expert elicitation***

*Information taken from EFSA: Guidance on Expert Knowledge Elicitation in Food and Feed Safety Risk Assessment.^17^

SEE literature suggests that individual level elicitation is the most effective approach when eliciting expert beliefs,^8, 77^ as it reduces the risk of bias due to experts influencing each other^60^ and promotes accountability to the decision maker. Individual level elicitation is also recommended for its transparency, in particular where expert judgements are available for peer review.^27^ Despite these advantages, one of the concerns associated with this approach is that experts may not feel confident in expressing uncertainty individually compared to a group situation.^53^ The literature reports that experts involved in group level elicitation are more confident about their decisions compared to experts at individual level elicitation. ^81^

Group level elicitation provides a substantial exchange of information amongst experts^51, 92^ and consequently, it is expected that more informed experts will have greater influence in the group.^81^ When aiming to achieve consensus face-to-face, a facilitator is required. The role of the facilitator is to engage the entire group and protect the process from becoming dominated by a subset of experts.^8^ If group elicitation is not monitored by an experienced facilitator, the interaction may pressure experts into reaching a consensus, and some experts may suppress their opposing beliefs.^51, 58^ Another concern associated with group level elicitation is that convening experts from various geographical areas at a time and place suitable for all can prove to be problematic. ^51^ Depending on appropriateness, the Delphi method can help overcome this issue as this method is conducted remotely using web-based survey tools. This approach is adequate when little but crucial information is required and a quick response is needed.^17^

In the healthcare literature, a 2013 review by Grigore reports individual elicitation is the dominant approach with 13 of the 14 included studies reporting individual level elicitation.^51, 52, 54, 55, 58, 64,53,^ ^56,^ ^60,^ ^62, 66, 68, 70^ Only two of these studies justify why the particular level was selected. Bojke et al (2010) report choosing individual level elicitation rather than group level to capture uncertainty within and between experts 19.^54^ Leal (2007) reports choosing it based on guidelines for HDCM developed by Philips et al in 2004.^51, 77^

As discussed in the introduction of this section, choices made for each component can have an effect on subsequent components of the SEE process, in particular, the chosen level of elicitation is related to the mode of administration and the method of aggregation used in the post-elicitation phase.

In terms of mode of administration, individual level elicitation is relatively versatile. Various platforms are reported when using the this approach; face-to-face^53, 60, 64, 68^ computer-based^56^ and email. ^51^ Given these possibilities it is not surprising that the literature indicates individual level of elicitation can be conducted face-to-face or remotely, and may be facilitated ^26, 52, 60^ or non-facilitated.^51, 54, 56^ In contrast, group level elicitation is not as flexible. At the group level, experts must be brought together, usually in one location^26^ and the elicitation must be guided by an experienced facilitator. Thus, this approach can be time consuming and costly. Once the experts are organized, two modes of administration are reported in the literature for group level elicitation; paper-based^57, 60^ and excel-based.^52^

As discussed in *Chapter 2*, the SEE process may strive to achieve a unique distribution that reflects the beliefs of all experts. This process is described as either “behavioural” or “mathematical” aggregation, and is related to the level of elicitation^8^ ^8, 81^ Behavioural aggregation relies on interaction between the experts to create a single distribution that reflects either the experts consensus beliefs or how an independent, rational observer would summarise the collective opinions of the experts. Mathematical aggregation, in contrast, combines individual assessments from several experts (who may or may not have interacted) into a combined group assessment based on an algorithm or mathematical process. Methods of mathematical aggregation are discussed in Section 5.5.2.

Behavioural aggregation can involve either the elicitation of a single group distribution, or elicitation of individual judgements followed by interaction to produce a consensus distribution (O’Hagan et al. 2006). Both Clemen and Winkler (1999) and O’Hagan et al. (2006) recognize that it may not always be desirable to strive for a consensus distribution.^8, 81^ Both suggest that in such cases, mathematical aggregation can be applied at the end of group interaction, thus combining behavioural and mathematical approaches. The EFSA Delphi method and IDEA protocol are both examples of this.^17, 25^ Clemen and Winkler (1999) ^81^ state that the benefit from group interaction is the sharing of information and not the forced consensus. They also believe that individual probability assessments are useful for understanding the range of expert opinion and conducting sensitivity analysis, thus supporting combined aggregation approaches or behavioural approaches that also involve individual elicitation (such as the SHELF method. ^29^

The empirical evidence on the merits of individual and group level elicitation is dated and may not be entirely relevant to HCDM. When solving problems that require ‘originality and insight’, Fogel (1967) commends the use of an interactive group.^82^ This is supported by Seaver (1976) who performed a comparative study of the three methods; individual elicitation, group interaction and the Delphi method and reports that the interactive group produced a larger number of ideas compared to the Delphi method.^83^ Stael Von Holstein (1971) report that results using the individual level approach are judged to be poorer than results from an interactive group.^84^ However, Meyer and Booker (1991)^92^, emphasise that these studies were not applying this method for deep problem-solving, the type of problem-solving for which it is most suited.

While there is a lack of empirical evidence available comparing individual and group level elicitation in healthcare, individual level elicitation is the most commonly adopted approach in the healthcare literature and the most recently recommended choice in guidelines for decision-making in healthcare. ^77^ The cost of using group-level elicitation will depend on the context, crucially, how physically dispersed the experts are. There may be a particular case for using group level elicitation: when the problem structure is unclear and there is a need for experts to develop a consensus problem structure and to specify the elicitation questions; when experts have distinctively different disciplinary backgrounds and knowledge bases (e.g. practising clinicians versus epidemiologists) and so require discussion to assess each other’s claims to expertise; or when it is expected that the experts will work together repeatedly and so group-level elicitation may also serve a team-building purpose.

## Section 6.5 Fitting & pooling

This section concerns the translation of the information elicited from one or more experts into a probability distribution representing the evidence on an uncertain quantity to inform decision-making. The following choices can arise:

1. To obtain a probability distribution from a single expert’s belief (or a behavioural aggregation process), should we pre-specify the form of the distribution and elicit its parameters directly, or elicit characteristics of an unspecified distribution, such as quantiles?
   a. If a distribution is pre-specified, what distribution should this be, and how should its parameters be elicited?
   b. If characteristics of an unspecified distribution have been elicited, what distribution should be fitted to the elicited information, and how?
2. If individual views are elicited from multiple experts, and we choose to mathematically aggregate them into a single distribution representing the overall spread of views, how should this be done?

### Section 6.5.1 Distribution choice and fitting

Methods of eliciting parameters of common distributions have been described, e.g. by Winkler (1967) and O’Hagan et al (2006), for example, the Beta distribution for probabilities.^98,^ ^78^ Only a small number of these methods have been evaluated and compared, and O’Hagan et al (2006) recommended much more work before advocating one particular method for use in practice.^78^

The disadvantage of pre-specifying a distribution is that it may not fit the expert's belief. Instead of eliciting parameters of pre-specified distributions, we may elicit characteristics of an unspecified distribution. Typically the expert is asked either for the quantiles that contain a given probability mass (e.g. median and credible intervals, as in the "bisection" method), or the probability masses that lie within a given set of quantiles (e.g. the “chips and bins” method) (see *Chapter 8)*. Either way, the elicited data consist of a set of points on a cumulative distribution function (CDF) (*Figure 5*). To obtain a fully specified distribution from these, the points could simply be interpolated, as described by O’Hagan et al. ^78^ More commonly, a parametric family of distributions is specified, followed by identifying the parameters which best fit the elicited data. The advantage compared to interpolation is that the fitted CDF is not necessarily assumed to pass through the elicited points, acknowledging that the expert may not be fully confident in the precise values that they provide. The “best fitting” parameters can be determined by numerical methods, such as least squares, as in SHELF,^99^ Leal et al (2007)^51^ and Thall et al (2017),^85^ which is justified by maximum likelihood principles. The red line in *Figure 5* shows the CDF of the best-fitting Beta distribution determined by this method. A related approach is the method of moments, an approximation to maximum likelihood, used by Bojke et al (2010)^54^ and Soares et al (2011)^52^ for two-parameter distributions.

**Figure 5 Elicited points on a cumulative distribution function (CDF) and alternative fitted distributions**

Standard conjugate families (such as the Beta or Normal) can be combined easily with future observed data using Bayesian inference. As an alternative to standard families, which may not fit the elicited data well, Bornkamp and Ickstadt (2009) proposed to fit a penalized spline function. ^86^ This method was implemented in their R package “SEL”. O’Hagan et al (2006) recommend a process of “feedback”, where the fitted distribution is presented to the expert with the opportunity to revise it to better reflect their beliefs.^78^ However in practice, there may not be sufficiently many elicited points on the CDF to identify distributions that fit better than standard ones.

As well as the expert not being fully confident in the precise values they provide, there may be multiple distributions which fit the elicited data equally well.^100^ Bayesian nonparametric methods to handle both these forms of uncertainty have been developed, see Oakley and O’Hagan (2007), Gosling et al. (2007), Moala and O’Hagan (2010) and Daneshkah et al. (2017).^101,^ ^102,^ ^87,^ ^88^ These methods generally require Markov Chain Monte Carlo (MCMC) simulation to implement, and, as far as we are aware, there is no accessible software to implement them. Such methods tend to be computationally intensive, which may not allow the fitted distribution to be instantly “fed back” to the expert during an elicitation session.

### Section 6.5.2 Mathematical aggregation

Mathematical aggregation methods fall into two general approaches: Bayesian combination (or “supra-Bayesian”) methods, and “axiomatic” (or “opinion pooling”) methods.

In Bayesian combination, the decision maker or modeller treats each expert assessment as new data and uses it to update his/her own distribution for the unknown parameter.^103^, ^104^ The resulting distribution thus represents the beliefs of the modeller given the elicited data.^78^ This is difficult to apply in practice, though, due to the detailed information required on biases in and dependences between the experts assessments.^105,^ ^81,^ ^78^ See, e.g. Lipscomb et al. (1997), Albert et al (2012), West and Crosse (1992), Gelfand, Mallick and Dey (1995) for some examples. ^106,^ ^107,^ ^108,109^

In opinion pooling methods, the aggregated distribution is an average of the distributions from each expert. “Linear pooling” uses an arithmetic average and was the most common aggregation approach used in our review of elicitation in HCDM.^1^ The alternative “log pool” uses a geometric average, and harmonic averaging has also been used.^110,111^ Past work has shown that there is no mathematical formula that can simultaneously satisfy a number of potentially desirable criteria,^112,^ ^81^ so there is no obvious justification for one combination rule over another. O’Hagan (2006) observe that log pooling discounts values found implausible by at least one expert, leading to a distribution concentrated on areas of agreement, while a linear pool encompasses all values that any expert finds plausible, leading to a broader distribution. Harmonic averaging gives an even more concentrated result than log pooling.^78^ Hammitt et al (2013) performed simulation studies to compare linear pooling with other mathematical aggregation methods, in situations where the experts beliefs were generated by a known mechanism, concluding that linear pooling performed worst, but it is unclear whether this mechanism holds generally.^113^ In HCDM, we would argue that the broader distribution from linear pooling is preferable, as it acknowledges the uncertainty arising from between-expert variation. This could motivate further research to obtain observed data on the uncertain quantity, and strengthen the evidence base for decision-making.

The weights applied to each expert’s belief in an opinion pool are commonly chosen to be equal. Alternatively they could represent an estimate of expected accuracy, determined using seed questions (discussed in the final section of this chapter), prior assessments of the expert’s background^80,^ ^114,^ ^115^ or agreement of the expert’s elicited data with subsequently-observed data on the quantity of interest,^115^ though observed data would not generally be available in a health care context. . More technically advanced methods that use only the elicited data to form weights are presented by, e.g., Ranjan and Gneiting (2010), Rufo et al (2012) and Hora and Kardes (2015).^116,^ ^117,118^ Essentially, these adjust the simple linear or log pools to give a better expected balance of overall bias and over / under-confidence.

Meta-analysis methods have also been considered for pooling expert beliefs^68,^ ^54^ but have been argued to be inappropriate,^54^ since they assume each expert’s view is fully based on evidence that no other experts have seen.

## Section 6.6 Assessing the expected accuracy of experts judgements

This section focuses on estimating the expected accuracy of the judgements elicited from an expert or group of experts, compared to the truth. The accuracy of a probabilistic judgement is often referred to as “calibration”. ^89,^ ^14,^ ^78^ The SEE literature recognises a number of different choices in this area, relating to:

1. *Measuring calibration using scoring rules.* When experts answer questions about quantities that have “realizations” (that is, known answers), called “calibration” or “seed” questions, the accuracy of their elicited judgements can be estimated by scoring rules that compare the elicited assessments to the realizations, a practice first proposed by Winkler and Murphy (1968). ^119^ The most common strictly proper scoring rule used in SEE is that of Cooke’s Classical Model, which has been used to elicit judgements and measure calibration in over 100 expert panels.^16^ More detail on this method is available elsewhere (e.g., Cooke 1991; Cooke and Goossens 2000; Quigley et al. 2018).^14,^ ^20,^ ^120^
2. *Using the scoring rule to create and/or evaluate combinations of experts.* In the Classical Model, scoring rules are also the mechanism for creating performance-based weights for mathematically aggregating expert assessments. The Classical Model’s scoring rule can also enable the evaluation of combinations of the experts. ^120^ In practice, this is typically done by comparing scores from the performance-weight linear combination of experts to the equal-weight linear combination to see which has better performance. This is done both within a study, to inform the choice of using performance- or equal-weights in the final reported results, ^90^ and across studies, to evaluate the method more broadly (Cooke and Goossens 2008^121^; Colson and Cooke 2017^73^; Quigley et al. 2018^120^).
3. *Deciding how many seed questions should be used and how relevant seed questions are identified.* Multiple seed questions are needed to assess the accuracy of elicited probability distributions,^78^ since poor calibration based on one seed question may indicate bad luck rather than bad performance. Identifying appropriate seed questions is a challenge because the questions must be closely related to the target questions but unknown to the experts participating in the elicitation. Seed questions are used to assess an expert’s skill in quantifying uncertainty, so they should not just be a test of the expert’s ability to recall established facts or familiar quantities.

Scoring rules for sets of variables, rather than individual variables, have been argued to be preferable, as the latter does not depend on the distribution of the realization. ^122^ The Brier score, for example, is a scoring rule for individual variables that was recently used to score expert forecasts of geopolitical events.^123,124^ Cooke (2014)^122^, however, provides simple counterexamples that demonstrate the issues with this approach to scoring. Strictly proper scoring rules are rules in which an expert maximizes their score by stating their true beliefs. As the objective of an elicitation is to capture the beliefs of the experts, it is critical that, if scoring rules are used to measure calibration, they must be strictly proper. ^122^ An improper scoring rule may reward an expert for providing assessments more extreme than their real beliefs.

Two studies compare scores from the performance-weight and equal-weight combinations of experts in 78 total applications of the Classical Model, and they find that the performance-weight combination is consistently both more informative and more statistically accurate than the equal-weight combination. ^121,16^ These studies are based on in-sample comparisons, where the same set of questions are used both to calculate the expert weights and evaluate the performance of the method. Out-of-sample validation, in contrast, would estimate performance using external data. However, data rarely become available on elicited quantities of interest (which is why elicitation is needed). An alternative approach is cross-validation, where the set of seed questions are divided into a training set and a test set. Expert scores are calculated based on the training set and then the performance-weight combination is evaluated on its performance on the test set. The most recent and extensive cross validation study of the Classical Model done to date found that the performance-weight combination of experts outperforms the equal-weight combination in 26 of the 33 studies.^73^ An evaluation of separate data, based on expert forecasts of the probability of various geopolitical events, also concluded that the accuracy of an expert’s assessments can be predicted by past performance on related questions, supporting the use of performance-based expert weighting (Hanea et al. 2018). ^121^

In the Classical Model, because the scoring rule is asymptotically proper, there is no theoretical basis for the number of seed questions required, but at least ten seeds is the recommended rule of thumb.^14^, ^20^ Simulations of expert scores show that using ten seed questions allows an analyst to distinguish between a well-calibrated and a slightly overconfident expert.^120^ Another paper argues that significantly more seed questions are needed, but it incorrectly understands the Classical Model’s scoring to be for the purpose of hypothesis testing, rather than for discriminating between experts^94^

Seed questions commonly come from four sources: future measurements, unpublished measurements, unfamiliar information from standard datasets, or combining or comparing different datasets.^120^ They discuss examples of each of these strategies from past applications of the Classical Model.

Although seed questions should be related to the subject of the elicitation, there is no clear test to measure if a question is “close enough” to the target questions. In practice, Classical Model elicitations focus on ensuring that the link between seed and target questions is strong enough that the problem owner, experts, and knowledgeable reviewers accept the resulting unequal weights of similarly qualified and knowledgeable experts. The Classical Model also recommends specific sensitivity analysis to identify if any seed questions have a large impact on the results.^20^

## Section 6.7 Conclusion

Different methods for selecting and recruiting experts are recommended in the SEE literature with very little empirical comparison. In terms of the skills that experts should possess, we believe adaptive skills are important in SEE in HCDM, given the potentially novel nature of some of the technologies the expert may make their judgements on. Yet there is a lack of acknowledgment of this skill in the existing literature. This is explored further in a HCDM context in *Chapter 8*. While some recommendations, such as the construction of a Profile Matrix, appear very useful, including such an exercise within the conduction of an expert elicitation process in HCDM may not be feasible due to time constraints on the overall project for which the elicitation is being conducted. With few conclusive recommendations, the findings of this targeted search suggest further analysis is required relating to the selection of experts in expert elicitation.

Individual level elicitation is conducted without interaction between experts, while group elicitation requires experts to interact to discuss the uncertain quantity. The relative merits of individual and group-level elicitation for HCDM are unclear, yet there are guidelines recommending individual level elicitation as more appropriate given the complex nature of quantifying uncertainty (likely to apply to HCDM) and the importance of seeing and understanding differences between experts. Despite these recommendations, the findings of this targeted search suggest that further research is required in this area.

Elicited data consisting of points on a CDF should be converted into a smooth distribution representing the assumed state of belief. To represent one expert’s belief, numerically fitting standard distributions such as the Beta to the elicited data will often be sufficient. If the number of elicited points is small, then more elaborate models would be difficult to identify. Standard distributions are simpler to implement, but more complex approaches are worth considering, particularly if they can be shown to give a better fit to the elicited data. Spline regression models can be fitted instantly in general software, however more experience of these is needed. More complex Bayesian non-parametric approaches can better represent uncertainty about the full belief distribution given the elicited data, however more guidance and accessible software are required before these can be recommended for routine use in HCDM.

To mathematically aggregate elicited data from multiple experts, linear pooling is simple to implement, and allows all experts views to be considered by the decision maker. While possibly difficult to interpret, as the final distribution does not represent any one person’s or group’s beliefs,^78^ it gives a conservative estimate of the extent of uncertainty, which can motivate future research (such as clinical trials) to obtain more evidence to support decision making. More guidance and accessible software would be needed before recommending more advanced pooling methods for use in HCDM.

The practice of weighting experts judgements according to estimates of their expected performance or calibration, particularly as implemented in the Classical Model, has been widely applied and studied, and has been found to improve the accuracy of aggregated expert assessments (Cooke and Goossens 2008; Colson and Cooke 2017, 2018).^121,^ ^16, 73^ However, the method has been largely underexplored in HCDM. After reviewing past HCDM-related elicitations, Soares et al. (2018) conclude that further research is needed to support the use of performance-based weighting in this area.^1^ Past elicitations in HCDM that have tested the use of performance-based weighting used four or fewer seed questions to evaluate the approach. Applications are needed that use the recommended 10 or more seed questions to better evaluate if performance-based weighting in this domain has the same benefits that have been identified across other application areas. Finally, future analysis of Classical Model applications would be beneficial to identify strategies for identifying and testing seed questions that have been useful specifically in applications with heterogeneous experts from a variety of disciplines and fields.

# Chapter 7 Reviewing the evidence: Heuristics and biases

## Section 7.1 Introduction

Formal models of judgement and decision making hold that judgements of probability and utility should be assessed using all of the information available to the decision maker, with the application of appropriate statistical rules.^125^ However, humans are not perfect information processors. The amount of information processed can be affected by time pressure, limitation

s in cognitive capacity, lack of motivation and personal desire for a particular outcome. When it comes to probabilistic reasoning specifically failure to recognise when a statistical rule should be applied and unfamiliarity with the processes for making statistical inferences, all mean that probability judgements do not always conform to normative rules.^126^ Experts, being human, are not immune from this. Indeed, even amongst highly educated populations, awareness of how to make simple statistical inferences can be limited.^127^ In the context of HCDM, those practitioners with the greatest relevant knowledge and expertise (e.g. nurses, physiotherapists), may not necessarily have a high level of training in statistics or experience with elicitation.

Humans often make judgements using simple rules of thumb (or “heuristics”).^126, 128^ These strategies are usually effective in appropriately guiding judgement^129^, especially amongst experts who have a large base of experience and knowledge to draw on.^130^ However, in some contexts they can lead to systematic errors, known as “biases”. SEE should seek to elicit probability judgements in a way that minimises the effect of these systematic errors. This is increasingly recognised in the literature on HCDM, where SEE can be used to inform health policy and treatment recommendations.^13, 44, 59, 97, 131^ However, while heuristics, biases and strategies for bias reduction have been widely studied in the broader risk, judgement and decision making literature, there is a dearth of evidence for HCDM and what does exist has not been summarised in this context.

This chapter reviewed evidence relating to the psychological biases of greatest relevance to SEE for HCDM, specifically evidence on how these can be minimised. First outlining key cognitive and motivational biases that have the potential to negatively impact on the quality of expert elicitation for HCDM are outlined (see Section 6.2), and then potential strategies for addressing them (see Section 6.3) through technical measures (see Section 6.4) and behavioural bias reduction techniques (. Reflecting the fact that some behavioural bias reduction techniques have a large amount of evidence to support them, while others are more tentative, techniques are categorised according to those for which a high degree of consensus exists and those for which evidence is lacking or conflicted. Finally, the key recommendations are summarised in Section 6.7.

## Section 7.2 Cognitive and motivational biases

A distinction may be drawn between cognitive biases, which result from how information is processed, and motivational biases that come about due to preferences for particular outcomes. ^76, 132^ Both have been implicated in systematic overconfidence, which poses a threat to calibration in SEE.

### Section 7.2.1 Cognitive biases

Cognitive biases arise when decision makers do not process the full range of information available to them. This may result from limitations in cognitive capacity, time pressure, or a lack of motivation to expend cognitive effort on a task. They may also arise due to decision makers lacking the normative skill to make appropriate probabilistic inferences. In the context of SEE, cognitive biases of particular importance include availability and anchoring and insufficient adjustment; firstly, because they are both implicated in overconfidence, which leads to the systematic underestimation of uncertainty in probability judgments. Secondly, because unlike biases that may result from deficits in substantive knowledge of a subject area, or from a lack of knowledge about how to reason with statistical information, both have the potential to affect expert judgement ^76, 133^.

In making probabilistic judgements people may rely on how easily examples of an outcome come to mind as a guide to how likely it is (the availability heuristic).^134^ While this is often a good guide to frequency, it means that probability judgements can easily be distorted by very recent or very prominent events. ^135^ For instance, a clinician may focus on particularly memorable examples of treatment success or failure when making probability judgements, neglecting instances that come less readily to mind. Availability bias has been linked to the systematic underestimation of uncertainty.^136^ Anchoring and insufficient adjustment occurs when people fix (“anchor”) on an initial value, and fail to sufficiently adjust their estimates away from it to provide an accurate judgement. For example in judging the success of an intervention, a clinician may “anchor” on a value provided by a source that they know to be flawed (e.g. a poor quality empirical study), and fail to sufficiently adjust their own experienced-based estimate from this point, despite being aware of the flaws and adjusting in the right direction.^128^ Anchoring has proved challenging to debias, with even arbitrary and irrelevant values being found to affect judgement (see Kahneman^126^ for overview). experts In SEE this can decrease accuracy in judgements of location and central tendency (e.g. mean, median).

### Section 7.2.2 Motivational biases

Motivational biases, sometimes referred to a “self-serving” biases, result from being invested in a specific outcome (e.g. a particular treatment being successful) (see Bazerman, 2008^132^ for discussion). In situations where individuals are aware of potential conflicts of interest and strive to make objective and honest judgements, motivational biases can still distort judgements through rendering some information and experiences more salient (cognitively ‘available’) and easier to recall than others. Confirmation bias for instance, leads individuals to focus on information that is consistent with their existing beliefs and preferences, and subject it to a less critical appraisal than inconsistent information. Desirability bias (also referred to as “optimistic bias” or “wishful thinking”), leads people to overestimate the likelihood of positive outcomes. Undesirability bias meanwhile leads to an overestimation of the likelihood of negative outcomes and worst case scenarios (e.g. due to a focus on taking a precautionary approach). These biases result from motivated reasoning rather than a lack of knowledge or experts.^76, 132^ Hence, they have the potential to adversely affect the outcomes of structured expert elicitation. In HCDM, where those with greatest knowledge of a particular treatment or procedure, may be those most invested

### Section 7.2.3 Overconfidence bias

As a consequence of limiting the amount of information considered by decision makers, both availability^136^ and confirmation bias^138^, may lead to the uncertainty surrounding future outcomes being underestimated. This is known as “overconfidence bias”. It leads to interval judgements and probability distributions that are too narrow (e.g. estimates of 80% confidence intervals containing fewer than 50% of subsequent realisations). Overconfidence is prevalent amongst experts as well as novices^36, 139^, making it an important consideration for any form of SEE

## Section 7.3 Addressing psychological biases in in SEE

Strategies for reducing psychological biases could be said to fall into three categories: technical (e.g. using formal statistical procedures to correct for systematic errors in judgement), directly changing individual behaviour and perceptions (e.g. through training, incentives, feedback) and changing the structure of the judgement or decision task (e.g. how questions are asked).^140, 141^ In practice however they represent two fundamental approaches: post-hoc statistical techniques to make corrections after the fact, most notably through calibration (discussed in *Chapter 5*) (technical); and interventions to change judgement and behaviour (behavioural).

In reviewing approaches for reducing psychological bias (or ‘debiasing’), we restricted our search to studies that provide empirical evidence for the efficacy of bias reduction in the context of SEE. For this reason, we have excluded papers that suggest approaches, but do not present empirical evidence to support them. We also exclude studies that focus on biases in decision from description (i.e. where choices can be made through analysis of a complete information set) rather than elicited judgements. Relevant papers that did not appear in the searches, but that were cited in the papers identified were examined and included where appropriate. A potential weakness of this approach is that bias reduction techniques that are relevant to SEE, but that do not mention expert elicitation directly, may have been missed if they were not cited in other papers identified through the search. However, a full review of the heuristics and biases literature, which often focusses on novice rather than expert judgement, is beyond the scope of this targeted search.

## Section 7.4 Technical bias reduction strategies

These are commonly discussed with respect to overconfidence. These can involve statistical bias correction and the weighting of experts based on their performance on seed questions, as is the case in Cooke’s Classic Model.^142-144^ These approaches do not require interventions at the individual or task level, as the procedures are applied post-hoc. However, they do rely on the availability of appropriate seed question from which the level of experts propensity to overconfidence can measured.^145^ This may be relatively easy in contexts where past realisations of the same or similar target variables are available (e.g. probabilistic weather forecasting). In HCDM however it could prove challenging to implement, as contextually similar seed variables with appropriate realisations are not always readily available. Likewise, HTA brings together diverse sets of experts who have specialist knowledge of specific treatments, interventions or procedures. They are not therefore guaranteed to have similar expertise on the subject of seed questions.^52^

## Section 7.5 Behavioural bias reduction strategies with consistent support

Given the challenges in applying technical approaches to bias reduction outlined above, it is important for those implementing SEE in the context of HCDM to consider behavioural approaches. In this section we outline bias reduction strategies for which there is consistent empirical support. In the next section we briefly discuss debiasing approaches for which there is conflicting evidence.

### Section 7.5.1 Consider more information

It has been found that individuals with a greater predisposition towards open minded thinking demonstrate better calibration on judgement tasks.^146^ Increasing the amount of information considered by participants may therefore be effective in countering these biases. Behavioural bias reduction techniques that prompt experts to consider more information (increasing the range of possibilities considered) have perhaps been the most frequently tested in the context of expert judgement.

Early research with student samples failed to find added value from instructing groups of participants to consider why their estimates may be wrong, or appointing one member to be a “devil’s advocate”.^147^ However, more structured approaches have had far greater success. ^138, 148, 149^ Soll and Klayman^138^ found that asking student participants to separately give lowest plausible estimates, highest plausible estimates and median estimates for almanac question with which students were likely to have some familiarity, led to lower levels of overconfidence than simply asking for a single 80% confidence interval. It was suggested that making people consider lowest, highest and median estimates sequentially focusses attention on a wider range of possibilities than asking for a single range (e.g. forcing participants to think of reasons why a value might be below (or above) a specific value. Building on this, Haran et al.^148^ found that further increasing the number of considerations by asking participants to make judgements about the likelihood of different local seasonal temperature intervals reduced overconfidence. Adding a fourth step to the procedure suggested by Soll and Klayman^138^, Speirs‐Bridge et al.^149^ found that ranges were widened further when participants (epidemiologists and ecologists) were asked how likely it was that the “true” value would fall within their specified range, and allowed to revise their estimates accordingly. This is consistent with research suggesting that people may be better at evaluating confidence intervals than providing ^150, 151^. More recently, Ferreti et al.^152^ noted reductions in overconfidence when environmental science students instructed to (a) actively think of reasons why their initial highest and lowest estimates of sea level rise may be incorrect; and (b) consider their willingness to place hypothetical bets on elicited confidence intervals.

Together, these studies provide strong evidence that structuring tasks in a way that increases consideration of a wider range of possibilities can reduce bias and improve calibration. They demonstrate that confidence intervals should not be elicited as a single stage process. Lower and upper bounds should be elicited individually^138, 149^, or multiple smaller intervals should be considered individually. ^148^ Likewise, they show that participants should be given the opportunity to evaluate and adjust their confidence intervals.

### Section 7.5.2 Feedback

There is extensive evidence that receiving repeated feedback on one’s judgements both improves accuracy and reduces overconfidence. ^36, 126, 145^ Experts, such as weather forecasters, who receive direct and timely feedback on the accuracy of their judgements tend to be well calibrated in their domain of expertise^153^, although this does not result in a domain general improvement.^141^ One suggestion for reducing the overconfidence bias in expert elicitation is to provide feedback on a set of practice questions.^154^ A challenge in doing this is the fact that domain specific seed variables may be more readily available in some contexts than others (e.g. past realisations in forecasting tasks). Hence, while this approach may be broadly effective in improving the calibration of expert judgement, it could be difficult to implement in some HTA contexts where identifying appropriate seed questions that a diverse set of experts will be familiar with could be challenging. Nonetheless, in cases where these are available, the existing evidence suggests that providing feedback seed questions can reduce overconfidence.

### Section 7.5.3 Avoid unnecessary anchors

Ensuring that elicitation materials do not contain unnecessary anchor values is a “common sense” approach to reducing biases cause by anchoring and insufficient adjustment.^76^ For instance, elicitation tools should not feature pre-set values that participants are then asked to adjust to match their views. However, it may not always be possible to eliminate anchors entirely. In the case of ‘carry over’ effects, for example, experts may use their own judgement on a previous question as an anchor.^155^ While there is some evidence to suggest that self-generated median anchors do not threaten accuracy and calibration to the same extent as those that are externally imposed,^138, 156^ Morgan^36^ advises that measures of central tendency (i.e. the median) only be elicited after lower and upper bounds have been estimated. Hence, while it may not be possible to eliminate all potential anchor values in an elicitation task, a clear recommendation to avoid unnecessary anchors can be made. Likewise, when eliciting confidence intervals, eliciting lower and upper bounds before the median, may reduce the tendency to anchor on the median value.

### Section 7.5.4 Reduce bias through expert selection

Addressing biases through expert selection means that experts are included or excluded based on their potential susceptibility to bias (see *Chapter 5*). As noted above, motivational biases such as desirability bias and confirmation bias are difficult to eliminate. Restricting participation to those without any conflicts of interest is therefore one recommended approach to reducing motivational^76^ biases. In HCDM this may be challenging, as those with the greatest knowledge about a particular treatment or technology may also be those with the greatest vested interest in the elicitation’s outcome.^44^ Rejecting those with any conflict of interest or strong opinions may eliminate those with greatest relevant knowledge. In such cases an alternative strategy is to ensure that a range of viewpoints are represented in the sample, with the intention of “balancing out” or at least diluting the effect of motivational biases.^76^

## Section 7.6 Behavioural bias reduction techniques with conflicting evidence

### Section 7.6.1 Bias warnings and training

Within the HCDM literature that considers heuristics and biases, training is the most commonly referenced approach to behavioural debiasing.^97^ Simply warning experts not to be biased (e.g. by stating that many people make their confidence intervals too narrow) is largely ineffective.^147, 156, 157^ However, in-depth training on the nature of biases, and strategies for avoiding them has been found to be more effective. Where biases occur due to experts not being familiar with rules for using and expressing probabilities, training on how to do so can reduce errors.^158^ Likewise, educating participants about biases and explicitly outlining strategies for combating them (i.e. through systematically considering more information) reduced overconfidence in a study of petroleum engineering students.^159^ However, this education programme was not effective for reducing anchoring, possibly because the student sample lacked the substantive knowledge of the field to give a more accurate value. Nonetheless, in a study with a general population sample^160^ found evidence that interactive training interventions, explaining what anchoring and confirmation bias were, reduced instances of these biases on post-intervention tests relative to pre-intervention tests. These tests comprised tasks from the wider literature found to elicit the psychological biases^161-163^ Hence, while the available evidence on the effectiveness of warnings and training for reducing psychological biases is not always consistent, it does provide an indication of the conditions under which bias avoidance training may be effective. Firstly, it must go beyond simple warnings and admonitions not to be bias and explain the causes and consequences of biases. Second, it should provide instruction as to how to avoid bias (e.g. consider why upper and lower bounds may be incorrect). Third, it can only be useful if participants have the substantive expertise to produce accurate responses.

### Section 7.6.2 Fixed value versus fixed probability methods

A small number of studies have examined whether fixed value (where one must allocate probabilities to potential values of a target variable) or fixed probability (where one allocates values of the target variable to probabilities) affect overconfidence. In eliciting cumulative probability judgements from students regarding forecast variables with which they were expected to have some familiarity (i.e. local temperature and the Dow Jones), Abbas et al ^164^ found less evidence of overconfidence using the fixed value method. However, Ferreti et al.^152^ found that this resulted in relatively little improvement in performance. Hence, while there is some evidence that fixed value approaches may reduce overconfidence, this is limited.

### Section 7.6.3 Face-to-face versus online elicitation

In one recent study it was found that face-to-face elicitation of energy demand with sectoral experts led to lower overconfidence than online elicitation.^165^ However, this finding was not replicated in a recent comparison of face-to-face and online SEE.^59^

## Section 7.7 Conclusions

The objective of this review has been to synthesise existing knowledge on the effectiveness of different behavioural bias reduction techniques for expert elicitation, focussing specifically on their potential usefulness in the context of HCDM. While the efficacy of some of these approaches remains under-tested, the following five recommendations are supported based on the available evidence.

- Confidence intervals should not be elicited as a single stage process, as doing so leads participants to focus on a narrow set of salient possibilities. Instead, lower bounds, upper bounds and median values should be elicited separately. Eliciting lower and upper bounds before median values may also prevent participants from anchoring on median values.
- Participants should be allowed to evaluate and revise their confidence intervals or probability distributions.
- In selecting experts those with pronounced conflicts of interest should be excluded. However, excluding all participants who may have strong feelings or vested interests in the outcome, may result in the exclusion of those individuals with the greatest expertise in the subject. Hence, it is important to ensure that different viewpoints will be represented.
- Where suitable seed questions are available these may be useful in providing practice feedback to participants on their performance, and thus reduce overconfidence. However, care should be taken to ensure that all participants will have familiarity with the topic of these seed questions.
- Bias training may reduce biases, but only if this goes beyond simple warnings, and explains what bias is and provides strategies for avoiding it.

# Chapter 8 Quantities to elicit

## Section 8.1 Introduction

Health care decision making (HCDM) is underpinned by (i) evidence of clinical- and cost-effectiveness from randomised trials, to support regulatory approval of drugs, and (ii) decision modelling based on clinical and epidemiological evidence, to support reimbursement decisions. *Chapter 4* provided a review of published applications in this area in order to determine the reasons for methodological choices made in published scientific literature (design, conduct, and analysis) and the challenges faced by the authors when conducting SEE. This chapter discusses different choices available for the specific quantities to elicit in SEE, particularly those related to simple and conditional probabilities of events, as well as parameters to inform survival rates and other time-to-event variables. Recommendations are made based on statistical theory underlying commonly adopted models and a series of targeted reviews of literature reporting current SEE practice.

Whilst data collected from trials typically aims to inform inference on a single probability, rate and hazard-related parameter, decision models^166^ combine a number of these to describe how different courses of action (for example treatments) affect patients’ progression through disease stages. Such models typically belong to one of three types.^167^ Decision trees, defined using simple and conditional probabilities, describe a set of possible pathways each assigned a probability that is influenced by the treatment being considered. Discrete-time state-transition models (STMs), such as discrete-time Markov chains, define the disease process using a finite set of health states, known to have distinct health and cost implications, and patients transit between states through time. The speed of progression is defined using a set of transition probabilities. Decision models can also be defined in continuous time models, and can be STMs^168^ or discrete-event models^169, 170^ (DES). STMs in continuous time are defined using transition rates. DES models use a number of events and use survival distributions to determine the time between events. For alternative treatments, STM and DES models determine the time spent in the different health states. To evaluate differences in lifetime quality-adjusted life years and costs (i.e. cost-effectiveness) from these models, costs and the health related quality of life (HRQoL) are attributed to time spent in each health state (or between events). In decision trees, the cost and HRQoL of each pathway is weighted by its probability.

To inform decision making, either based on single parameters, such as in clinical trials, or based on multiple parameters as in decision modelling, empirical and/or elicited evidence may be used. Quantitative expression of ‘individuals’ beliefs regarding a parameter (or parameters) of interest, should be expressed as probability distributions. This chapter gives examples of alternative quantities that can be elicited to inform the probability-related or time to event-related parameters commonly used in HCDM.

## Section 8.2 Overview of probability-, rate- and hazard-type parameters

First the main parameters of interest for HCDM and the relationships between them are described.

### Section 8.2.1 Simple probability and conditional probability parameters

The probability of a discrete event that an individual may experience once, for example incidence of a disease in a specified time interval and post-operative mortality, can be represented by a single parameter $\pi=p(E)$. Probabilities may be altered by (or associated with) a particular attribute, e.g. treatment, another event or a particular characteristic of the individual. In a conditional probability, the event D is conditioned on the specific value the attribute takes. Conditional probabilities arise, for example, in diagnostics where the sensitivity of a test reflects the probability of testing positive conditional on having the disease, or in logistic regression analyses (say) where the coefficients represent how the outcome of interest is affected by certain attributes, such as age and disease severity.

Where the event of interest has more than two levels, a set of probability parameters is relevant, which are constrained to sum to one given the fact that the categories are mutually exclusive. The probabilities of potential events can be modelled using a Multinomial distribution or, alternatively, expressed as conditional Binomials.

### Section 8.2.2 Transition probability parameters in discrete-time STMs

STMs define a set of health states and describe transitions between them (for example, alive to dead) over a prolonged time horizon. In discrete time STMs, such as Markov chains, the total follow-up period is divided into a number of short consecutive time intervals (cycles). The speed at which transitions between the health states occur is governed by probabilities of the occurrence of the transitions in a particular cycle, termed transition probabilities.

An important feature of discrete-time STMs is that, in any cycle, they typically consider transitions from the current state to one of several health states or competing events. Moreover, individuals may re-enter previously occupied health states. Consider the simple example of a homogeneous (through time) three state-transition model in *Figure 6***Figure 6**. From state A individuals may transit to either health state B or die (state Death) – these are, in this context, competing events. From health state B individuals are allowed to move back to state A (i.e. backwards transitions are allowed). Death is an absorbing state since once entered, it cannot be exited. To evaluate this discrete-time STM, a transition probability matrix, or TPM (as illustrated in *Figure 6***Figure 6**) needs to be defined, where each row of the matrix must sum to one, so that that all individuals in a particular state at a cycle c are allocated to the allowed states at cycle c+ 1. Transition probabilities may not change over time or, alternatively, cycle-dependent transition probabilities may be specified.

| Model diagram |  | transition probability matrix (TPM) | | |
| --- | --- | --- | --- | --- |
|  |  | A | B | D |
| A  B  Death | A | P[X_c_=A\|X_c-1_=A] | P[X_c_=B\|X_c-1_=A] | P[X_c_=D\|X_c-1_=A] |
|  | B | P[X_c_=A\|X_c-1_=B] | P[X_c_=B\|X_c-1_=B] | P[X_c_=D\|X_c-1_=B] |
|  | D | 0 | 0 | 1 |

**Figure 6 Example of a transition diagram for a state-transition model (STM)**

### Section 8.2.3 Time to event and survival

Survival and time to event outcomes are defined in continuous time typically using a hazard function, h(t), representing the rate of the event which can take any value above zero. The hazard function can also be used to calculate the survival function, S(t). The mean or expected value of T is the area under the survival curve and can be derived by integrating the survival function.

A number of parametric statistical models can be used to specify the time-to-event distribution, the simplest being the Exponential, which assumes the hazard is constant for all times, h(t)=λ. In this case, λ can be interpreted as the event rate per unit time. The mean and median times to an event occurring in the Exponential model are respectively 1/λ and ln(2)/λ, where ln(2) is the natural logarithm of 2, (approximately equal to 0.69). S(t) for the exponential model is exp(-λ*t), where t is the relevant time-frame for the survival estimate (*Table 3*). In many circumstances, a constant hazard is unrealistic, and more complex parametric models than the Exponential are required – examples are the Weibull, Gompertz, Log-Logistic, Log-Normal and the Generalised Gamma, for details see, for example, reference ^171^. *Table 3* describes key functions and summaries for parametric models commonly used.

**Table 3: Summaries of selected survival distributions**

| Distribution | Hazard function | Survival function | | Summaries of the time to event distribution | | |
| --- | --- | --- | --- | --- | --- | --- |
|  |  | P[T>t] | | Mean | Median | Variance |
| Exponential | λ | exp(-λ*t) | λ>0 | 1/λ | ln(2)/λ | 1/λ^2^ |
| Weibull | pλt^p−1^ | exp(-(λt)^p^ ) | λ>0, p>0 | Г(1+1/p)/ λ | (ln(2))^1/p^ /λ | {Г(1+2/p) – Г(1+1/p)^2^ }^2^ /λ^2^ |
| Gompertz | exp{α + βt} | exp(-ε(e^bt^-1)) | ε>0, b>0 | Requires integration | (1/b)* ln[(1/ε) *ln(1/2)+1] | Complex |

Г is the Gamma function

Use of continuous time to event parameters in discrete-time STMs

Despite many analyses using a discrete time model, hazard functions from continuous time to event models are often used to derive their parameters. The simplest way to consider a time-varying hazard is to use a value for the hazard that changes between cycles but is constant within each cycle. Transition probabilities for different cycles are then derived to define a discrete time Markov chain that approximates the continuous time estimates using the following relationship:

P[t<T≤t+c] = P[T≤t+c|T>t] = 1- S(t+c)/S(t).

### Section 8.2.4 Continuous time-to-event decision models

Two types of continuous-time models are currently used more often in health care cost-effectiveness analyses: continuous-time STMs and discrete event simulation models. For both types, the clock reset model is adopted^172^, where all transition probabilities are expressed in terms of time spent in the current state. Continuous-time STMs are typically represented by state transition diagrams of the type exemplified in *Figure 6*, however these models are defined by a matrix of transition intensities which are derivatives of transition probabilities with respect to time (at time zero) and may vary with the length of time spent in the currently-occupied state or time spent in the study overall. The discrete time-evolution of the probability of transiting between health states can be evaluated using a system of (partial) differential equations defined on the transition intensities called the Kolmogorov equations.^173^

Discrete-event models are informed by the times at which discrete *events* occur. These models define (through a parametric time-to-event model) the hazard of exiting each particular health state (independently of where to), which determines the mean time spent in that particular health state. Separately, additional probability parameters define the arrival state. Discrete-event models are typically evaluated by Monte Carlo simulation.^170^

### Section 8.2.5 Repeated event rates

Some models may represent events that occur multiple times, for example, rejection episodes in transplant patients, infections or other exacerbations in chronic lung diseases, hypoglycaemic episodes in diabetics, asthma attacks, or seizures in epileptics. These may be represented by a process that counts the number of events over a given time-interval, called a Poisson process governed by a rate parameter, λ, representing the number of events occurring per unit of time (independently of time, in a homogenous process). The time taken between consecutive pairs of events of a homogeneous process is Exponentially distributed with a parameter λ. The common situation in which event rates differ between individuals may be modelled using a negative binomial distribution, which requires that an additional parameter representing inter-individual variation.

Repeated events could reasonably be represented in a discrete-time STM if the time intervals are sufficiently short,^174^ or one could construct an STM where transition to states that represent these events can occur repeatedly. The probability of an event per unit of time, or the number of events occurring by time period (which may vary with time) can both be considered in informing these quantities.

## Section 8.3 Eliciting probability, rate and hazard-related parameters

The relationships described in Section 7.2demonstrate that target parameters for elicitation (for example transition matrices in STM or time to event distributions) may be described using a number of alternative quantities. This is because the underlying phenomena described, of progression of a disease process, inherently involves multiple events happening in continuous time and models that do not explicitly consider time (decision trees) or those in discrete-time constitute simplifications of a more complex underlying process. Whilst models simplify reality, this does not mean that the evidence (empirical or elicited) that informs these models cannot be gathered in a way that more closely reflects the underlying processes. For example, a discrete-time STM can be based on inference obtained from empirical time to event data modelled using a particular (continuous time) survival function. Conversely, simpler evidence on a probability (for example, reflecting one point in the survival function) can also be used (alongside assumptions or other evidence) to inform continuous time models that use the entire survival function. To inform one or more parameters of interest, an elicitation need not be restricted to directly eliciting the model parameters; instead, a range of related quantities can be considered.

When eliciting survival functions, for example, if a constant hazard can be assumed, then prior elicitation can be achieved via the mean time to the event. Alternatively, the median time to the event may be more intuitive, since experts may have a clearer picture of the time when the first half of the individuals have experienced the event compared to the second half, some of whom may have very long times to the event. Alternatively, the proportion π of individuals who experience the event by a particular time t can be elicited. In this case, the parameter from the exponential can be calculated as,

$\lambda= \frac{-ln(1-\pi)}{t}$ ,

Which, in turn, can be used to calculate the full survival function S(t). If a more complex survival function reflecting a time dependent hazard is used, its parameters can be elicited from experts, either directly, or more likely indirectly from survival or conditional survival estimates for two or more time points, though there may be multiple ways of accomplishing this.

## Section 8.4 Current practices in elicitation

### Section 8.4.1 Identification of examples

The aim here is to find examples for illustration, rather than provide a comprehensive review. To identify examples, we initially drew on three reviews that had been used by the authors in recent overviews of SEE for clinical trials or decision modelling.

1. A previous review of studies that included SEE to elicit distributions of parameters included in health care decision models was updated^1^. This review excluded studies that generated utility estimates for health states using preference elicitation.
2. A review of experimental studies that used Bayesian survival analyses, most of which were trials in cancer patients and described randomised trials, whilst half were in diseases described as rare^175^. Only one of the 28 applications elicited priors from experts^176^.

A systematic review of 33 studies that used elicitation of prior beliefs for Bayesian analysis was reassessed in order to extract information on the validity, reliability and responsiveness of quantities that were elicited^177^. In order to identify a wide range of applications, other targeted (non-systematic) searches were undertaken. These targeted searches sought Bayesian studies including elicitation of simple and conditional probabilities, survival rates, and other time-to-event variables, as well as reviewing recent technology assessment reports that stated that Bayesian methods, including SEE, were used. The references for all the applications considered in this paper are listed in *Table 4* by type of quantity elicited. Since many reports lack clarity in how the quantities elicited related to the target parameters of interest, the next section discussed is structured according to the type of quantities elicited.

**Table 4: List of published examples of eliciting probability, rate and time to-event parameters**

| **Quantities elicited** | **Examples** |
| --- | --- |
| Simple and conditional probability or odds (see Section 7.2.1) | Decision modelling healthcare literature: ^52, 53, 56, 58-61, 67, 69, 70^  Broader health literature:^178-180^ |
| Transition probabilities of an STM (see Section 7.2.2) | Decision modelling healthcare literature: ^52, 63, 181, 182^ |
| Time to event and survival (see Section 7.2.3) | Decision modelling healthcare literature: ^51, 52, 57, 58, 64, 65, 69, 70^  Broader health literature:^48, 176, 183-190^ |
| Hazards or parameters of the hazard function (see Section 7.2.4) | Outside Health: ^191^ |
| Repeated event rates | No studies found |

### Section 8.4.2 Simple and conditional probability or odds

In decision modelling, the elicitation of simple probabilities is relatively common^52, 56, 59-61, 67, 69, 70^, for example, proportion of individuals susceptible to clinical infection^66^, perioperative mortality^69^ or prevalence of cervical cancer recurrence.^60^ Some of these papers elicit independently for different subgroups^56, 60, 66, 67^ or for different durations of treatment.^61^ One example of the elicitation of a prevalence was also found in the broader health literature.^178^

Applications in decision modelling also elicit probabilities conditional on other events, for example to inform decision trees. An example is provided in Garthwaite *et al*^58^, where an elicitation exercise was designed to inform a decision tree in which more than two outcomes in a single branch were possible.

The authors used conditional probabilities to re-structure the tree and elicited a set of conditional Binomials. A simple extension of the basic structure is presented in *Figure 7*.

Diagnostic

testing

Novel test (B)

Standard test (A)

Treatment intervention

a)

b)

| **Questions elicited** | **Re-structured tree** |
| --- | --- |
| (a) What proportion of patients referred for investigation of symptoms do not undergo diagnostic testing (i.e., go straight to treatment intervention)?  (b) What proportion of the patients referred for testing undergo novel test B, rather than standard test A? |  |

**Figure 7 Example of eliciting conditional probabilities for a decision tree**

The same authors^58^ also considered the need to more formally elicit how the probabilities depend on covariate values. The approach to the elicitation of dependencies was based on conditional probabilities: experts were asked about the quantity of interest by conditioning on a set of values of the covariate(s). These assessments were then analytically transformed to determine regression coefficients using a generalized piecewise-linear model. Specially developed software based on graphical displays was used (Prior Elicitation Graphical Software^16^).

Two examples^53, 60^ elicited probabilities related to diagnostic accuracy parameters. Despite requiring sensitivity and specificity, both studies elicited probabilities conditional on test results. One ^60^ elicited the proportion of false positives and false negatives (independently), and the other^53^ the proportion of true positives and true negatives.

*Treatment effects on probabilities or odds*

A few examples were found in the broader health literature eliciting absolute difference in probabilities, ^184, 188^ or ratios of probabilities (i.e. relative risks).^179, 180^

### Section 8.4.3 Transition probability parameters in discrete-time STMs

Soares et al^52^ elicited several quantities assuming conditional independence, but one strategy used in this study extended such the approach to ensure that elicited quantities were consistent with existing relevant data. In their applied example, a particular health state representing healing could be achieved in two ways – either by a wound being left open to heal or via further surgery to close the wound edges. The existing data did not distinguish between these healing types. In order to delineate experts beliefs regarding surgery from beliefs about healing, the probability of closure surgery conditional on healing outcomes was elicited. Denoting the closure surgery event as S and healing as H, the authors elicited: the probability of patients having had surgery given that they healed, P[S|H]; and the probability of healing in patients who received surgery, P[H|S]. By knowing the unconditional probability of healing, P[H], and applying Bayes’ theorem, the probability of receiving closure surgery was calculated as: P[S] = (P[S|H]*P[H])/ P[H|S].

Wilson et al^181^ elicited a total of 12 STM transitions on the progression of undiagnosed melanoma between cancer stages or death. The authors considered each row of the TPM as parameters of a Multinomial distribution. Experts were asked to distribute a cohort of 100 patients according to the stages they would be in six months later. These values were described as medians, and the software restricted the values introduced to sum to 100. The participants were then asked to elicit the lower and upper limits of 95% credibility intervals (CrIs) for each stage; for these no restriction was imposed on the values provided. The participants elicited in the same way for all other starting health states.

Cao et al^63^ took a similar approach to Wilson et al^181^ but elicited membership of each health state at a particular point in time. Experts were initially presented with a diagram of the model with relevant empirically derived numbers for standard of care, and were then asked to revise these for a new care setting, as exemplified in*Figure 8.* Cao et al ^63^ elicit in ‘discrete-time’ but use the elicited quantities to inform a continuous-time model with the same structure. They argue that transition rates are complex and not-observable quantities, and hence did not elicit these directly.

| State 1  State 2  Death  a%  b%  (100-a)%  (100-b)% | QII (a): Under standard care, out of all the patients who left State 1 within 1 year, a% moved to State 2 and (100-a)% died. Can you adjust these numbers to reflect the proportion of events that you expect to observe after 1 year of follow-up under the new care setting? |
| --- | --- |
|  | QII (b): Under standard care, out of all the patients who left State 2 within 1 year, b% moved to State 1 and (100-b)% died. Can you adjust these numbers to reflect the proportion of events that you expect to observe after 1 year of follow-up under the new care setting? |

**Figure 8 Example of eliciting transition probabilities for a novel setting in a discrete-time STM**

Vargas et al ^182^ also inform the transition probabilities of an STM, but the publication provides little detail on how these quantities were elicited.

### Section 8.4.4 Time to event and survival

Some studies elicited summaries of time to event distributions, particularly median^69^ and mean survival.^57^ Survival functions have also been elicited to inform transition probabilities in a discrete-time STM.^51, 52, 64, 65^ Some studies elicited event probabilities at a single time point.^51, 64, 65^ For example, Leal et al 2007^51^ asked: “*If 100 hypertrophic cardiomyopathy patients were stratified as low/medium risk at the age of 18, how many would be classified as high risk at age 50*”. Some applications elicited multiple event probabilities without eliciting dependency between them. Examples are Poncet et al,^65^ that elicited separately for different subgroups of patients, and Speight et al,^64^ that elicited probabilities of sequential cancer progression (e.g. pre-cancer to stage I, stage I to stage II, and so on).

To explore time-dependency and derive a full survival function, some studies elicited conditional probabilities at two or more points.^52, 58^ One such study^52^ elicited a first point in the survival function at six months and, for those who did not have the event at that time point, the proportion who would have the event between six and 12 months. By assuming conditional independence between the elicited quantities, the authors were able to incorporate time dependency in the decision model without generating incoherent probability statements. They also argued that, even if hazards are found to be very similar in both periods (i.e. no evidence of time dependency), experts may be more uncertain about outcomes in the longer term, so that it may still be important to elicit for separate time periods. Garthwaite et al^58^ used a similar strategy but partitioned the time scale into 4 intervals.

### Section 8.4.5 Treatment effects on time-to-event distributions

Experts may judge survival with a comparator treatment informative of survival with the treatment of interest, i.e. hazards should not be assumed independent.^183^ To elicit priors for relative treatment effects (hazard ratios), a number of authors^176, 183, 184, 186-189^ elicit the absolute difference in event probabilities (at a single time point) between treatment and comparator. Most of these authors convert the absolute difference onto the log hazard scale assuming a value for the baseline hazard (example in ^188, 189^). The study by Ren et al^183^ considered eliciting absolute differences in survival under time dependency, and proposed eliciting the following quantities:

- survival with the comparator at a particular time point, t_0_,
- the difference in survival for the comparator between times t_1_ and t_0_ (where t_1_ > t_0_),
- the difference in survival between the treatment of interest and the comparator at t_0_, and
- the difference is survival for the treatment of interest between times t_1_ and t_0_.

Note that this method also relies on a form of conditional independence.

Other authors^52, 190^ asked experts to elicit absolute survival probabilities with the treatment of interest conditional on a given fixed value for the comparator (in Soares et al^52^ the value selected was the elicited mode for survival with the comparator whereas in ^190^ the fixed value was provided to the participant). Soares et al^52^ elicited relative effectiveness for multiple treatments independently, and White et al^190^ evaluated treatment effects in the presence of possible interactions across different patient groups.

Dallow et al^48^ argue that to better manage the tendency for experts to be over-optimistic, experts should be first asked to judge the probability that the drug has a true positive effect and then to judge the distribution of this effect size under the assumption that the drug does have a favourable effect. They then formed a mixture distribution to represent the overall prior for the treatment effect.

Chaloner et al^185^ aimed to specify a bivariate distribution for two hazard ratios (two treatments in relation to a common comparator), eliciting survival probabilities with the support of a graphical dynamic tool. Analogously to Soares et al^52^, the authors initially ask experts to elicit absolute survival probabilities for each treatment conditional on an initial model value for the comparator (conditional independence is assumed throughout); specifically, experts are asked for their upper and lower quartiles. The relationship between survival probabilities elicited for a treatment and control and the hazard ratio under a proportional hazard model -- {log(-log(1-p)}=β + log{-log(1-p_0_)}, where p0 and p are the elicited survivals for the control and treatment, respectively, and beta the treatment effect -- allow the elicited summaries to be expressed in terms of treatment effects. These are used to calculate initial values for parameters of a type B bivariate extreme value distribution describing the treatment betas. The distribution is defined on the means and variances of the marginal distributions and on an *m* parameter, with m=1 reflecting independence between coefficients. To pick an initial value of *m*, the expert is additionally asked about the probability that the survivals for each treatment being larger than their respective marginal medians. If the two parameters are independent, this probability is 0.25. To reflect correlation (note that only positive correlation is allowed), values for this probability can be higher than 0.25 (up to 0.5) and the value for m can be directly determined from these. The expert is presented with plots of each marginal distribution (for the probability parameter), a contour plot of the joint prior distribution of the survival probabilities with approximate confidence regions, and a dialog box with five sliders (for each parameter of the bivariate distribution). Changing the value of m in the slider does not change the marginal distributions but it does change the contour plot. The sliders allow the expert to adjust interactively the parameter values and see the consequences directly. The authors recommend repeating the elicitation process for a different follow-up interval; under proportional hazards, the distributions on the regression coefficients should be equal.

### Section 8.4.6 Elicitation of hazards or of parameters of a time to event distribution directly

The only example of directly eliciting time to event distributions related to reliability assessment in engineering.^191^ Singpurwalla presented methods for eliciting a single Weibull distribution, and proposed directly eliciting its shape parameter (alpha), which characterises whether hazards increase, decrease or remain constant over time. We note also that the shape parameter can be expressed as a function of the hazard ratio h2 associated with a doubling of time: alpha = 1 + log2(h2), so the shape might be derived by eliciting h2. Singpurwalla argued that the scale parameter is difficult to interpret and instead of eliciting it directly, chose to elicit median survival time, making the simplifying assumption that the two parameters are independent. Indeed, the scale is related to the mean and median but only has a simple relationship (independently of the shape) when the shape parameter is 1 (Exponential distribution).

## Section 8.5 Steps and considerations in defining the quantities to elicit

The examples reviewed identify a range of ways to elicit probability, rate- and hazard-type parameters used in current practice. Whilst most did not directly elicit the parameter of interest, there was often little justification for the quantities chosen for the elicitation. Following critical review of the literature described in Section 7.4and investigator experience of SEE, some generic guidance on how to determine appropriate quantities to elicit was developed and agreed during discussions within the research team and is presented here.

Step 1: Develop a clear understanding of the statistical or decision model, so that, ultimately, quantities elicited are fit-for purpose (i.e. accurately represent the relevant context, including, population, setting, interventions, outcomes and time horizon).

Prior to defining the quantities to elicit, the target parameters of interest for decision-making must be defined. This can be done independently from the elicitation and requires two assessments. The first is to specify the model (statistical model or decision model), by defining a list of input parameters required and outputs produced by the model, to inform the decision. Models are developed specifically to best represent decision problems involving particular types of health care strategies (for example, diagnostics, drugs, complex interventions, screening strategies, infectious disease prevention). Within a well-defined set of model input parameters, the second assessment identifies which inputs have strong empirical evidence and, of those that do not, which might benefit from explicit priors being elicited from experts. The level of uncertainty, and whether it ultimately impacts on the health care decision, is a critical consideration here. Value of information approaches ^192^ can help to identify those parameters that are most influential, and prioritise parameters for elicitation.

Step 2: Consider breaking down (decompose) the target quantities for the elicitation into quantities that are simpler and that reflect what experts are likely to observe

The parameters defining statistical or decision models can be complex, for example, hazards and intensities are difficult concepts. In contrast, eliciting a probability of having the event of interest by a set of specific times, for example, survival up to one year, is more intuitive, and might represent data that experts observe directly. From the resulting elicited probabilities, and under some assumptions, survival and hazard functions can be constructed.

Step 3: Consider what sets of related target parameters are required, and define quantities to elicit in a way that ensures coherence between the quantities elicited and the parameters they inform.

Target parameters, and the quantities elicited, may be related in a number of ways: total survival is a compound function of simple and conditional probabilities, number of people infected is a combination of exposure rates and infectivity, and positive predictive value is a function of sensitivity and prevalence. Relationships between target parameters, between quantities elicited, and between the target parameters and the quantities elicited need to be understood and accommodated, so that statistical coherence of the priors generated is ensured. Eliciting two points in the survival curve unconditionally does not guarantee consistent results, and should be avoided.

Coherence is important when eliciting multinomial outcomes or discrete-time STM probabilities. If multinomial probabilities are elicited independently with uncertainty, they may not sum to one. As an alternative, a Multinomial can be re-expressed a set of conditional Binomials.^58^ Or Multinomial probabilities can be elicited directly by eliciting expected proportions in each health state and an effective sample size that informs uncertainty.^78^ Consistency in the quantities defined and elicited is important, and can either be ensured by design or verified using consistency checks built into the elicitation tool.

The relationships and constraints identified above can generate dependencies. But other forms of dependency are also relevant, such as dependencies between quantities or between quantities and known covariates. Dependencies should be considered and accommodated, either by re-expressing target parameters as conditionally independent quantities, or by formally eliciting dependency. Note that dependencies between quantities may arise from some experts being prone to eliciting higher values across the board than others.

Step 4: Consider what the expert may not observe, e.g. censoring, heterogeneity.

Analogously to empirical studies, it is common for clinicians not to observe times to event for all patients in their clinical practice: there may be competing events that removes patients from the risk of the event of interest, or patients may change practitioner or become hospitalised and not be observed after a certain time point. Experts will find it difficult to elicit quantities under heavy censoring. Hence, we believe that experts asked about summaries of a time to event distribution will find the median more intuitive than the mean, and that shorter time points may be necessary when eliciting survival (at the expense of need for more extensive extrapolation).

Step 5: Where more complex quantities need to be elicited (for example, bivariate treatment effects), consider using dynamic graphical displays.

Graphical displays used in the elicitation did help the experts to provide parameter values whilst visualizing probability distributions on quantities that were more intuitive to them. The graphs also provided useful instant feedback.

Step 6: Where alternative quantities of interest can reasonably be elicited, or graphical displays used, pilot the different options with a small number of the relevant experts.

Piloting is essential to both choose the most intuitive set of quantities for the elicitation, and to optimise the quantities using the principles above (for example, validate the time point at which a survival probability can be reasonably asked under censoring), and optimise the wording of the questions for clarity.

## Section 8.6 Discussion

Survival or time-to-event models, and transition probability/intensity matrix based models, pose challenges for elicitation, as inputs are typically complex constructs that may involve several correlated parameters. Instead of target parameters being directly elicited, these may be decomposed into related quantities that are simpler and observable to experts. This chapter aims to review current practice of elicitation to identify the quantities elicited for these types of parameters. Given the low specificity of search terms, efficient targeted searches were employed, giving a good overview of what is published up to date. Current practice is heterogeneous, with different quantities used to elicit the same type of target parameters, for example survival distributions and how these vary with treatment and other predictors. Some general guiding principles for determining appropriate quantities to elicit are developed here. Further research could refine these recommendations, particularly if there are multiple options or if implementation of principles is unclear.

One may need to retrieve hazards, survival functions and relative effectiveness parameters from the elicited priors. Whilst this is not the focus of this chapter, it is an important aspect that would benefit from further guidance, as there may also be multiple ways of estimating the whole distribution to the level of detail required for decision modelling.

# Chapter 9 Three methodological experiments on the elicitation of subjective probabilistic belief

## Section 9.1 Introduction

Research looking at important methodological choices in elicitation is mostly inconclusive, for example on which experts to engage, how to most appropriately elicit distributions, or whether group interaction increases the accuracy of group judgements. The underlying challenge of methodological research in this area is that beliefs are inherently unobservable: the accuracy of elicited judgements in representing the experts beliefs cannot be directly established as heterogeneity in knowledge (i.e. the fact that individuals’ beliefs differ) cannot be easily disentangled from the lack of ‘normative’ skills (i.e. individuals not being able to represent their beliefs accurately in probabilistic terms), and there is no “gold standard” perfect elicitation procedure.

This chapter describes the approach used in three experiments aimed at generating evidence that can support some of the methodological choices in elicitation. It first describes the general approach for the experiments, and then details aspects of their design. The experiments explore alternative methodological choices in SEE:

- The first experiment compares two methods of elicitation (bisection versus chips and bins),
- The second evaluates whether individuals are able to accurately extrapolate from their knowledge-base to different populations, and
- The third experiment (comprising of two separate sub-experiments) explores how individuals revise their own probability assessments when presented with Delphi-type group summaries.

The objectives were chosen to address some of the key methodological challenges for conducting elicitation in HCDM, reported in Chapters 4-7.

The objectives in experiments 2 and 3 differ to those outlined in the funding proposal. We had initially thought of exploring the accuracy of consensus-based methods and use the experimental set-up to evaluate alternative methods of mathematically pooling priors elicited from individual experts. Consensus-based methods can be affected by many factors, including facilitators’ input, individuals’ experience, their ability to adjust (or extrapolate) their knowledge and beliefs, and the composition of the sample of experts whose consensus is sought, including their personalities, probabilistic accuracy and between-expert agreement. When planning for experiment 1, we realised that a sample of the size we would be able to recruit, would not allow for meaningful inferences under these objectives (as randomised groups would have to be formed). Moreover, the review in Chapter 5 highlighted that there was no good evidence on a key question: how do individuals revise their own assessments after some form of interaction. This is crucial for all methods that require individuals to revise their assessments (both consensus methods and controlled interaction methods such as Delphi). For these reasons, the objectives of experiments 3 were updated to explore this, and used the more controlled interaction in a Delphi-type environment. Experiment 2 was a natural extension from experiment 1 and aimed to explore how experts deal with heterogeneity in knowledge, which was one of the objectives set-out in the funding proposal.

The variation from the protocol was approved by the project team and by the advisory group, and the revised objectives are described in further detail in Section 8.3.3 and Section 8.3.4.

## Section 9.2 General approach to the experiments

The aim of these experiments was to compare alternative design choices in SEE. An approach in which the individual’s knowledge is determined by observations from a simulated (virtual) learning process (Wang, 2002) is employed. This process is reflective of the learning process (by observation) we may expect of experts in health, typically health carers that observe patients over time. Additionally, if the simulated learning process constitutes the single source of information participants receive, elicited probabilities can therefore be directly compared to the posterior probability distribution implied by the observed dataset (and any prior beliefs, even if vague) – i.e. accuracy can be measured. The ability of this experimental approach to control the subjects’ knowledge means that the conditions of the experiment can determined, which allows testing specific hypotheses, and standardising the task to reduce between-participant variation.

Finally, in this approach, and given that the same information is provided to all participants, systematic differences in the elicited distributions across individuals can directly be attributed to different levels of normative ability, reflecting the skills needed to extract information from observations and quantify the resulting beliefs in probabilistic terms. The particular dimension of normative ability captured in these experiments is referred to as ‘probabilistic’ accuracy.

The following methods section summarises the protocol for the experiments which is presented in full in ***Error! Reference source not found.****.*

## Section 9.3 Methods

### Section 9.3.1 Overview of the experimental approach

The game and target question

Participants were shown a number of observations generated randomly from a statistical model, which were recorded. The context was that of an abstract generic medical problem so that all knowledge was acquired from the game and not influenced by external information or separately acquired prior beliefs. In summary, participants in the experiment were asked to act as practitioners over a number of clinic days (the number of days is defined in each experiment, see details ahead). In each day, participants cared for a variable number of (simulated) patients (between 6 and 13, randomly sampled). Participants were presented with two pills and asked which they would use to treat patients that day (*Figure 9)*.

**Figure 9 Snapshot of the R shiny app (1)**

Once the participant chose the pill to use that day, a new screen appeared (*Figure 10*) showing how many of the patients achieved symptom relief (number of successes).

**Figure 10 Snapshot of the R shiny app (2)**

After observing a number of clinic days, participants were asked about the effectiveness of the most effective pill – the target question for the elicitation – phrased as: “About pill <<>>, the most effective of the two pills... If you were able to treat all patients in the population, what proportion would you expect to become symptom-free?”.

Before running the game, all participants were shown 6 pre-determined runs of the game, 3 on each pill, to mitigate against sensitivity of results to different uninformative or vague priors that subjects may use. These were equal across all subjects.

Participants and monetary incentives

Given that the subjects’ knowledge-base is ‘built up’, it is more important that participants are representative of the type of normative skills we expect from experts in health care. Hence, students at the University of York with a health background or undertaking clinical training were recruited. The recruitment target was 64 participants, based on the requirements for experiment 1 and constrained by available funding. No formal sample size calculation was undertaken given the lack of evidence on the potential magnitude of effect size and its variance. Information on participants was collected, such as age, gender, the Berlin Numeracy Test^193^ and the Scott and Bruce's General Decision-making Style questionnaire.^194^ Monetary incentives for performance were used as students may not be as motivated to complete the task as might be expected of professionals. The reward was individualised, and incentivised both the learning from playing the game and accuracy in the elicitation– description of the incentives is provided in the experimental protocol, Supplementary material 2.

Metrics for comparison

The aim was to find a metric or set of metrics that allow comparison of the elicited distribution against the posterior distribution, calculated from the prior and data provided to participants. Bias was defined as the mean of the elicited (and fitted) distribution minus the mean of the true distribution; uncertainty, defined as the ratio of the standard deviation of the elicited distribution to the standard deviation of the true distribution; and the Kullback-Leibler (KL) divergence, a measure of the information lost when the true distribution is approximated by the elicited distribution^13^.

Other aspects of conduct

The experiments were conducted in a number of face-to-face sessions, each lasting around 2 hours. Subjects were, however, asked to complete all the tasks individually (i.e. the game and the elicitations). The games and elicitations were conducted in a tool developed for purpose in the SHINY package for R.^195^ A number of pilot exercises were undertaken to evaluate the feasibility of the experiments, time to completion, the optimal experimental conditions (e.g. value of the probability parameters), and to test the tool developed. A bespoke training package was developed and delivered to participants. University level ethics approval was sought from the host institution and granted prior to the conduct of the experiment.

### Section 9.3.2 Experiment 1: comparing different methods of elicitation

The aim of this experiment was to assess how well the elicited probability distributions derived from two different methods of elicitation, the bisection and the chips and bins (or histogram), both widely used for SEE in HCDM,^1^ reflect description(s) of bias and uncertainty. The bisection method is a VIM and asks experts to give the three quartiles of the distribution. The chips and bins method is a FIM that defines a larger number of intervals (typically up to 20 bins) and asks the expert to distribute a fixed number of chips across these intervals. The more chips placed in a particular interval, the stronger the belief that the true value of the quantity of interest lies in that interval. Both methods were preceded by asking participants for bounds (see **Error! Reference source not found.** for further details on how the two methods and questions regarding bounds were implemented). In HCDM, the general understanding is that the bisection method returns wider representations of uncertainty (argued to be more appropriate in representing within-expert uncertainty) than the chips and bins, although the latter has been said to be more intuitive for less quantitative experts to grasp.^1,^ ^59^

Given this experiment uses the set-up described in Section 8.2, where participants observe data directly on the target question, this experiment focusses on how well individuals express their uncertainty, and not on bias. Different levels of uncertainty are defined by varying the number of clinic days participants observe with the pill of interest and by assuming, or not, over-dispersion in the probability parameter. The number of clinic days observed is 25 in the higher precision scenario and 10 in the lower precision scenario. The high precision scenarios uses a Binomial model (no overdispersion), and the low precision scenario a Beta-Binomial model with an effective sample size (α+β of the Beta distribution) assumed a value of 2. In this context, over-dispersion implies participants observe greater variation in the probability of success between clinic days.

The experiment used a full factorial design with all 4 combinations of the two levels: precision scenario and method of elicitation. The experiment used a repeated measure design, with participants’ beliefs elicited for all four combinations. In each repetition, different probability values (p_0_ values) were used of: 0.3, 0.4, 0.6 and 0.7. Hence a 4x4 Graeco-Latin design was implemented.

At the end of the experiment, participants were also asked if they found each of the methods (generally) easy to complete (Response options: easy, challenging, very difficult) and if they had any preferences regarding the elicitation method used (“*If, in a future elicitation, you were given a choice between chips and bins or bisection, which would you choose*?” - Response options: chips and bins, bisection, indifferent. *Please justify your choice* - Response in free text). An open text box for further comments was also provided.

### Section 9.3.3 Experiment 2: Are individuals’ able to ‘extrapolate’ from their knowledge-base?

Variation in elicited judgements across experts may arise from experts having a different knowledge-base from which they form their beliefs. To provide a probability distribution for a common target quantity, individual experts need to adjust (‘extrapolate’) their beliefs using some form of analytical reasoning. A simple example is where a health care professional observes a sample comprising two subgroups, but the subgroup distribution observed is different to that of the overall target population of the decision question. The expert has a knowledge base that is relevant (in that he/she observes both subgroups) but when their belief at the population level is elicited they need to adjust (or re-weight) what they directly observed. This experiment examines how well individuals make such adjustments, by looking at whether, in the case of extrapolation, accuracy of the extrapolation is associated with non-extrapolated accuracy and with the extent of the extrapolation (difference in the split between observed and target populations).

This experiment used a different set-up from that of experiment 1. At each clinic day, participants were shown a number of patients that were sampled randomly. However, here patients were from two groups (S1 and S2) (*Figure 11*). Participants were told that one group had a better chance of symptom relief than the other, but at the beginning of the experiment participants did not know whether this was S1 or S2. The number in each subgroup was generated randomly from a Binomial distribution on each clinic day. Different probability parameters for this Binomial were examined reflecting odds of being in groups S1:S2 of 80:20, 70:30 and 60:40.

**Figure 11 Snapshot of the R shiny app (4)**

Two new pills were available in clinical practice, and these were used by the participant in exactly the same way as in experiment one. Once the participants chose the pill they wished to use, they observed outcomes for the two subgroups (*Figure 12***Figure 12**). The number of successes was governed by a Binomial model (as in the high precision scenario in experiment one), with probability 0.35 in the largest subgroup and 0.65 in the smallest. Participants observed 15 clinic days with the pill of interest.

**Figure 12 Snapshot of the R shiny app (5)**

Participants were asked to provide their beliefs first for a target population with same split as they had observed and then for a 50:50 split (*Table 5***Table 5**). Participants were not told which split was assigned in their observed experiment.

**Table 5 : Wording of questions in experiment 2**

*“About pill A, the most effective of the two pills...*

*Suppose that the patients you have just observed were representative of the general population, that is, the split of S1 and S2 patients is unchanged.*

*If you were able to treat all patients in the population with this pill, what proportion would you expect to become symptom-free?”*

*“Now suppose that that the general population is different to the sample you observed. Suppose that subgroup S1 makes up 50% of the population and subgroup S2 makes the other 50%.*

*If you were able to treat all patients in the population with the same pill, what proportion would you expect to become symptom-free? “*

In terms of design for this experiment, participants were randomised to receive one of the three scenarios described above; randomisation was by block according to the method of elicitation.

### Section 9.3.4 Experiments 3: To understand how individuals review their own probabilistic assessments when presented with Delphi-type summaries.

Experiments 3 aimed to gain an understanding of how individuals revise elicited distributions when presented with Delphi-type summaries, loosely based on the recent modified EFSA Delphi method that allows quantifying of uncertainty in the form of probability distributions.^17^ As with the original Delphi, this method makes use of multiple (sequential) questionnaires (called ‘rounds‘), and at every round experts are fed back an anonymised summary of the information collected in the previous round. This form of interaction between experts is controlled, and advocates of the Delphi method argue that it allows for the benefits of the sharing of information without the risks of personal factors influencing judgements inappropriately. In contrast to the original Delphi, the modified EFSA version does not aim to achieve consensus; instead, after all rounds are completed, a final distribution is obtained using mathematical aggregation with equal weighting.

Despite the benefits of reduced interaction between elements of the group, how individuals revise their estimates in a Delphi process is not well understood.^196^ This is the focus of the two sub-experiments conducted here.

#### Section 9.3.4.1 Experiment 3.1: Is the extent of revision associated with discrepancy with the group, and does the individual’s probabilistic accuracy determine the extent of revision?

This experiment aimed to evaluate if low performers (in terms of probabilistic accuracy) revised their answers to a greater extent (to approximate the group’s distribution) than high performers. If this were true, then over multiple rounds of Delphi the group distribution would be expected to converge to a more accurate distribution than initial estimates mathematically pooled—i.e. the iterative process may dilute the effect of low or extreme performers. How different features of the group distribution shown to participants determined the extent of revision, was also explored.

This experiment uses the set-up for the high precision scenario in experiment 1, where participants run the game and are first asked to elicit the directly observed target question. Participants then received one of three types of group summary of the quantity of interest: concordant with their initial probability distribution, or discordant and either more or less precise than their own (*Figure 13*). The group distributions were hypothetical (groups were not formed), and were defined relative to the individual’s elicited distribution (but always towards the true distribution).

Figure 13 Illustrative example of the scenarios evaluated in experiment 3.1

After observing the group summaries, participants were asked if they wished to revise their elicited distributions in light of the group summary.

In terms of the design of the experiment, participants were randomised to receive one of the three scenarios described above; randomisation was undertaken by block according to the method of elicitation.

#### Section 9.3.4.2 Experiment 3.2: How does between-expert variation within a group affect individuals’ revision?

In this experiment, participants were presented with disaggregated results for each member of a group to examine its effect on individual’s revision. For such, two scenarios were generated based on exactly the same linearly pooled group distribution (which was discordant with the individual’s): one scenario defined higher levels of within-expert uncertainty (but concordant central estimates) and another scenario defined higher levels of between-expert variation (with discordant central estimates but higher precision in individual distributions) (*Figure 14***Figure 14**). This experiment aimed to examine how individuals revise their estimates when presented with either of these scenarios.

This experiment used the same set-up as experiment 3.1, however, instead of a single group distribution, participants were presented with the distributions of three other individuals and then asked whether they would like to revise their judgements in light of this information. The group distribution was not presented to the participant. The mean for the group was discordant with the individual’s elicited distribution and was the same for the two scenarios – see **Error! Reference source not found.** for more details on how this was operationalised.

Participants were randomised to receive one of the two scenarios described above; randomisation was by block according to the method of elicitation.

Scenario 2: Others in group are discordant amongst themselves but present lower within-expert uncertainty

Scenario 1: Others in group are concordant amongst themselves but present a higher within-expert uncertainty

**Figure 14 Illustration of the scenarios defined for experiment 3.2**

## Section 9.4 Methods of analyses

### Section 9.4.1 Outcomes and metrics used

All experiments required a measure of participants’ accuracy in elicitation. Experiments 3.1 and 3.2 also require a measure of the likelihood of revision and of the extent of revision. Accuracy was evaluated by comparing the elicited distribution to the theoretical posterior distribution implied by the prior and data provided to participants.

The elicited summaries consisted of a set of quantiles and probability masses that define points on the CDF of the quantity of interest. The target quantity for elicitation was a probability, thus by definition takes a value in the range [0,1]. Fully-specified distributions were derived from the elicited summaries by fitting a Beta distribution to the bounds (assumed to represent the 98% confidence interval) and the elicited summaries, using least squares on the elicted CDF points. Distributions were fitted in R, using the *fitdistr* function in the SHELF package.^195^

Methods for deriving the posterior distributions varied depending on the precision scenario, i.e. on whether the Binomial (high precision) or the Beta-Binomial model (low precision) was used. With the Binomial model (used in experiments 1, 2, 3.1 and 3.2) the posterior distribution was obtained using conjugacy with a Beta prior. In experiment 2 (Section 8.3.3), the posterior distribution of the extrapolated quantity was obtained using conjugacy within each subpopulation, then sampling from each distribution according to the ratio between them to derive the posterior (the R code is provided in ***Error! Reference source not found.Error! Reference source not found.***). For the Beta-Binomial model (used solely in the low precision scenario in experiment 1), the posterior distribution for combining the Beta-Binomial data with the Beta prior was obtained by MCMC and approximated by a kernel density estimate.

Three different accuracy metrics were used to compare the elicited distribution to the posterior:

- Difference in the means of the elicited and posterior distributions, which here represents a measure of bias. The absolute value of the difference in means (absolute bias) was also used to capture how much the mean deviates from the true proportion using a metric that is independent of the direction of bias.
- The ratio of standard deviations (SDR) between the elicited distributions and the posterior distribution measure was used as a measure of how well the elicited distribution represented the true level of uncertainty; this was presented either in the natural scale or in the log scale (SDR or lnSDR). Values above 1 on the natural scale (and above 0 on the log scale) indicate underconfidence (overestimation of uncertainty) and values below 1 on the natural scale (and below zero on the log scale) indicate overconfidence (underestimation of uncertainty). Absolute lnSDRs of the difference between true and elicited distributions were also used to capture how accurately the elicited distribution represented uncertainty, independently of the direction of any inaccuracy.
- KL divergence is a measure of the information lost when one distribution is approximated by another^13^. The KL was computed using numerical methods of integration (details in *Error! Reference source not found.*). KL can take any value between 0 and infinity, where zero indicates that the two distributions are identical (i.e. the elicited is without error), and values higher than zero indicate that the elicited distribution is less accurate.

In experiments 3.1 and 3.2, the proportion of participants that revised their priors was observed (see *Chapter 9, Section 9.3.8*for details). The extent of revision was calculated only for those who revised, by comparing the elicited distributions before and after revision. The three different metrics outlined above were also used to determine the extent of revision, but their interpretation differs:

- Mean of revised distribution minus mean of originally elicited distribution. Positive values indicate the revised mean moved away from the originally elicited distribution towards the group mean, negative values mean the revised mean moved in the opposite direction to the group mean.
- The ratio of the standard deviation of the revised distribution to the standard deviation of the original distribution. This is greater than 1 (or lnSDR > 0) when the participant became less certain, and vice versa.
- KL divergence of the revised distribution from the original distribution. This combines both bias and uncertainty, and higher values indicate a greater extent of revision.

Note that, for brevity, this chapter presents a set of results on select metrics of outcomes, focussing on bias, lnSDR and lnKL. Results for the full set of outcome metrics is presented in ***Error! Reference source not found.***.

### Section 9.4.2 Methods

The full factorial design means that quantities of interest can be computed and relationships of interest can be displayed from simple plots and summaries of the data. Means (and standard deviations), medians (and interquartile ranges) and histograms were used to describe each metric of accuracy. Comparisons between methods were illustrated using scatter plots for within-participant accuracy. For example, for experiment 1 the accuracy with Chips and Bins was plotted against accuracy for Bisection (x and y axis, respectively), with precision scenarios highlighted using different markers.

For experiments 3.1 and 3.2, the proportion of participants that revised was compared across different randomised groups and different elicitation methods. The extent of revision was evaluated using empirical summaries and scatter plots.

Linear and generalised linear modelling was also used to confirm the conclusions drawn in this chapter, to provide estimates with confidence intervals for particular quantities of interest and to identify any effect of covariates such as period effects. These models and results are fully detailed in ***Error! Reference source not found.***.

## Section 9.5 Results

### Section 9.5.1 Description of the sample recruited

In total, 72 participants completed the experiments in 8 sessions (4 to 24 participants per session).^[1]^ Participants earned on average £30.6 (range £20 - £40). The sample characteristics are shown in *Table* 6. In summary, participants were on average 22.3 years old, 80.5% were female, and 80.6% were undergraduates. Half of the sample was very or somewhat confident in using probabilities, and the average BNT score was 3.7 out of 7. On average, participants scored higher in the ‘rational’, ‘intuitive’ and ‘dependent’ decision making styles, than in the ‘avoidant’ and ‘spontaneous’.

**Table 6 Sample characteristics**

| N | | 72 |
| --- | --- | --- |
| Mean age (sd) | | 22.3 (5.7) |
| Male (n) | | 19.4 (14) |
| UG (n) | | 80.6 (58) |
| Year of study (sd) | | 1.6 (0.8) |
| Percentage with qualifications in quantitative subjects (n) | A level | 38.9 (28) |
|  | AS level | 9.7 (7) |
| Percentage confident in using probabilities (n) | Very confident | 4.2 (3) |
|  | Somewhat confident | 45.8 (33) |
|  | Neither confident nor unconfident | 27.8 (20) |
|  | Somewhat unconfident | 16.7 (12) |
|  | Very unconfident | 5.6 (4) |
| BNT score, out of 7 (sd) | | 3.7 (1.6) |
| Score on Scott and Bruce’s Decision Style Inventory, out of 5 (sd) | Rational | 4.1 (0.5) |
|  | Intuitive | 3.5 (0.6) |
|  | Dependent | 3.7 (0.8) |
|  | Avoidant | 2.5 (1) |
|  | Spontaneous | 2.5 (0.8) |

### Section 9.5.2 Experiment 1

In Experiment 1, 288 priors were elicited from the 4 games played by each of the 72 participants. *Figure 15* shows the observed distribution of bias, lnSDR and lnKL scores (results for the full set of outcome metrics are shown in ***Error! Reference source not found.****, Figure 1*. Bias was symmetrical around the value of zero, suggesting participants were equally likely to over- and underestimate proportions. This was expected in the context of this experiment as participants were observing evidence directly on the target question. In high precision scenarios, participants were more likely to be under-confident (lnSDR greater than 0), while the opposite was the case in low precision scenarios. The standard deviation of the lnKL scores was high in relation to its mean.

|  | High precision & Low precision   |  |
| --- | --- | --- |

**Figure 15 Distribution of bias, lnSDR and lnKL. (SDR= SD elicited / SD true)**

Figure 16**Figure 16** presents scatterplots of the pairwise comparisons of participants’ accuracy when different elicitation methods were used (see ***Error! Reference source not found.***, *Figure 2* and *Table 2*).  Results on bias showed widely scattered points, so that variability in responses dominated any systematic differences between people. Mean and median bias are close to zero and comparable across precision scenarios. There is, however, a higher dispersion of bias values in the low precision scenario (plot a), which means *absolute* bias is higher on average in this scenario (results for absolute bias in ***Error! Reference source not found.***, *Figure 2*). Bias and absolute bias are comparable between the two elicitation methods.

For uncertainty (plot b), the results within each high precision scenario suggest that there may be a weak correlation between responses from the same participant, particularly in the high precision scenario. As highlighted in the descriptive histograms in *Figure 15*, lnSDR differed between the high and low precision scenarios. In the scatter plot here, this is evidenced by a clear separation of points. In the low precision scenario, the two elicitation methods result in similar mean lnSDR (the blue point is on, or close to, the diagonal line). In the high precision scenario, bisection priors are much more likely to have higher SDRs (higher proportion of points above the diagonal line than below), that is, responses are more likely to be underconfident.

There is no clear correlation between responses from the same person for KL divergence (plot c). The KL distribution appears more widely dispersed in low precision scenario. In the high precision scenario, the two methods appear to result in similar mean accuracy (the red point is on the diagonal line). The relevance of any observed differences in KL is unclear due to the high variability in scores.

c)

a)

b)

**Figure 16 Within-participant comparison of accuracy when different elicitation methods were used, for each precision scenario.**

Further detailed results of analyses are presented in ***Error! Reference source not found.***. Note that the modelling confirms the empirical results.

#### Section 9.5.2.1 Participants’ preference for each method

The results (*Table 7* and *Table 8* suggest that participants were more likely to find the Bisection method difficult or challenging, and were also more likely to prefer Chips and Bins to Bisection.

**Table 7 Response to question 1 about the ease of completion.**

|  | Easy, % (n) | Challenging, % (n) | Difficult, % (n) |
| --- | --- | --- | --- |
| Bisection (n=72) | 23.6% (17) | 66.7% (48) | 9.7% (7) |
| Chips and Bins (n=72) | 43.1% (31) | 51.4 % (37) | 5.6% (4) |

**Table 8 Response to question 2 about method preference.**

|  | Bisection | Chips and Bins | Indifferent |
| --- | --- | --- | --- |
| Preferred, % (n out of 72) | 31.9% (23) | 65.3% (47) | 2.8% (2) |

### Section 9.5.3 Experiment 2

In total, 72 participants played 72 games and 144 priors were elicited (72 for a target question not requiring extrapolation and 72 for a different target question requiring extrapolation). The overall bias, SDR and KL scores in initial priors (compared to the truth) were comparable to those in high precision scenarios in experiment 1 (see ***Error! Reference source not found.***).

*Figure 17* compares participants’ accuracy between priors elicited without and with extrapolation, see ***Error! Reference source not found.****, Figure 4* and *Table 5* for results on the complete set of outcome metrics). Results suggest that mean bias is close to zero. There is no suggestion that bias, SDR or KL differ with and without extrapolation, and no suggestion that these results differ between the three different extents of extrapolation. The scatter plots for lnSDR, however, indicate a moderate correlation between lnSDRs of distributions elicited from the same person with and without extrapolation.

b)

a)

**Figure 17 Within-participant comparison of accuracy with and without extrapolation, for different levels of extrapolation.**

### Section 9.5.4 Experiment 3.1

In total, 72 priors were elicited for the initial quantity and 32/72 (44%) participants updated their priors (i.e. revised) on seeing the group response. The overall bias, SDR and KL scores in initial priors were comparable to those in high precision scenarios in experiments 1 and 2 (see ***Error! Reference source not found.***). The high variability of KL between participants makes results on this metric difficult to interpret, and hence these are omitted throughout (but available in Appendices).

#### Section 9.5.4.1 Likelihood of revision

*Table* **9**8 shows the proportion of participants who revised their priors, by type of group summary. The table shows that participants were more likely to update their priors when the group distribution was discordant from their own prior, and when the group prior was more certain for the same level of discordance. The probability of revision with Chips and Bins (20/35, 57%) appears to be higher than with Bisection (12/37, 32%) (see ***Error! Reference source not found.****, Table 12).*

**Table 9 Proportion of participants who revised their priors.**

|  | Concordant (N=25) | Discordant, equally uncertain (N=24) | Discordant, more certain (N=23) |
| --- | --- | --- | --- |
| Proportion who revised their prior (n) | 20% (5) | 54.2% (13) | 60.9% (14) |

Detailed results of the regression analysis are presented in ***Error! Reference source not found.***. Consistently with the empirical results, the models suggest that participants were significantly more likely to revise their priors when the group was discordant, when the group was more certain, and when using Chips and Bins compared to Bisection. Participants’ level of uncertainty on the initial prior (lnSDR for the initial prior) had a significant effect on the likelihood of revision (with participants expressing more uncertainty in their initial prior showing a higher likelihood of revision), while the effects for bias and KL divergence were not significant.

#### Section 9.5.4.2 Accuracy in initial priors of participants who did and did not revise

**Table 10***Table* **10***9***Table 10** shows summaries of accuracy (on initial priors) for participants who did and did not revise their priors (selected outcomes presented, for the full set of outcomes see ***Error! Reference source not found.****, Table 15*). The results suggest that there is no notable difference in the initial level of bias between those who revised and those who did not. Those who revised were perhaps slightly more uncertain in their initial priors (higher lnSDR), although the difference is small.

**Table 10 Accuracy of initial priors compared between participants who did and did not revise their priors: mean (SD) [median and (interquartile range)] of accuracy metric over participants**

| **Mean (SD)**  **[median (IQR)]** | **Concordant (N=25)** | | **Discordant, equally uncertain (N=24)** | | **Discordant, more certain (N=23)** | |
| --- | --- | --- | --- | --- | --- | --- |
|  | **Revised (n=5)** | **Not revised (n=20)** | **Revised (n=13)** | **Not revised (n=11)** | **Revised (n=14)** | **Not revised (n=9)** |
| **Bias** | -0.011 (0.088)  [-0.039 (-0.068 to 0.044)] | -0.004 (0.068)  [0.005 (-0.017 to 0.025)] | 0.018 (0.081)  [0.031 (-0.036 to 0.043)] | 0.013 (0.067)  [0.02 (-0.004 to 0.044)] | 0.016 (0.068)  [0.011 (-0.027 to 0.059)] | 0.033 (0.054)  [0.032 (0.003 to 0.074)] |
| **lnSDR** | 1.14 (0.25)  [1.04 (0.95 to 1.22)] | 1.03 (0.39)  [1.10 (0.67 to 1.36)] | 1.21 (0.42)  [1.23 (1.01 to 1.32)] | 0.94 (0.46)  [1.05 (0.62 to 1.29)] | 0.95 (0.49)  [1.097 (0.656 to 1.235)] | 0.72 (0.64)  [0.94 (0.57 to 1.21)] |

#### Section 9.5.4.3 Extent of revision in those who revised

*Figure 18* shows accuracy in initial priors and extent of revision, using bias and lnSDR in initial priors. A more complete set of outcome metrics are presented in ***Error! Reference source not found.***.

The average change in mean was highest when the group was discordant and more certain than the participant, and the lowest when the group was concordant with the participant. Participants who saw group summaries concordant with their own prior, on average revised to a more certain prior (lower lnSDR). Some participants who saw group priors discordant with their own but with similar uncertainty became more certain and others less certain. Almost all participants who saw group priors discordant but more precise than their own, and chose to revise their prior, revised to express a more certain prior.

**Figure 18 Within-participant comparison of accuracy to initial priors and extent of revision, for different types of group summaries.**

Finally, for all outcome measures, there is no evidence that the extent of revision was different for different levels of probabilistic accuracy, as the extent of revision was distributed fairly evenly across the x-axes.

### Section 9.5.5 Experiment 3.2

In total, 72 priors were elicited on the initial quantity, and 27/72 (38%) participants updated their priors upon seeing priors from three other individuals in the group. The bias, SDR and KL scores in initial priors were comparable to those in high precision scenarios in experiments 1 and 2 (see ***Error! Reference source not found.***).

#### Section 9.5.5.1 Probability of revision

The proportion of participants who revised their priors, per type of group response and by elicitation method is shown in *Table* **11**10. This shows that participants were more likely to update their priors when participants within the group were consistent and that participants may be more likely to revise their priors when using Chips and Bins, which conforms to findings from experiment 3.1.

**Table 11 Proportion of participants who revised their priors.**

|  | Consistent (N=34) | | Inconsistent (N=38) | |
| --- | --- | --- | --- | --- |
| Proportion who revise their prior (n) | 52.9% (18) | | 23.7% (9) | |
|  | Bisection (n=18) | C+B (n=16) | Bisection (n=20) | C+B (n=18) |
| Proportion who revise their prior (n) | 44.4% (8) | 62.5% (10) | 0% (0) | 50% (9) |

Detailed results of the logistic regression analysis on likelihood of revision are presented in ***Error! Reference source not found.***. Consistently with the data summaries, the models suggest that participants were more likely to revise their priors when participants in the group were consistent with each other, and when using Chips and Bins compared to Bisection. Furthermore, the model coefficients suggested that the effect of probabilistic accuracy on participants’ likelihood of revision was not significant.

#### Section 9.5.5.2 Accuracy of initial priors in participants who did and did not revise

*Table* **12**11 shows the accuracy of initial priors in participants who did and did not revise their priors (results for all metrics are presented in ***Error! Reference source not found.****,* Table 15). The results suggest that there is no notable difference in bias or uncertainty between those who revised and those who did not.

**Table 12 Results of experiment 3.2: Outcomes in participants who did and did not revise their priors.**

| **Mean (sd)**  **[median (IQR)]** | **Consistent (N=34)** | | **Inconsistent (N=38)** | |
| --- | --- | --- | --- | --- |
|  | **Revised (n=18)** | **Not revised (n=16)** | **Revised (n=9)** | **Not revised (n=29)** |
| **Bias** | 0.036 (0.064)  [0.026 (-0.007 to 0.081)] | 0.023 (0.07)  [0.017 (-0.005 to 0.048)] | 0.038 (0.078)  [0.066 (-0.04 to 0.102)] | 0.046 (0.082)  [0.039 (0.006 to 0.104)] |
| **lnSDR** | 1.01 (0.55)  [1.17 (0.71 to 1.38)] | 0.82 (0.68)  [0.92 (0.53 to 1.21)] | 0.93 (0.34)  [0.88 (0.74 to 1.00)] | 0.90 (0.66)  [1.11 (0.67 to 1.29)] |

#### Section 9.5.5.3 Extent of revision in those who revised

*Figure 19* shows the accuracy of initial prior and the extent of revision for different types of group summary (see complete set of results in ***Error! Reference source not found.****, Figure 8* and *Table 16*). The average change in mean was higher when group members were consistent with each other (but inconsistent with the participant). Absolute change in mean, however, appears to be more comparable between the two groups (see ***Error! Reference source not found.***).

When shown group members that were consistent with each other, some participants revised to become more uncertain and others less uncertain. When shown group members that were inconsistent with each other, most participants who revised became more certain (all white points but one are below the ‘no change’ line, and the mean change in uncertainty (blue triangle) is below the line).

**Figure 19 Within-participant comparison of accuracy and extent of revision (using selected outcome metrics), for different types of group summaries.**

For a complete set of results see **Error! Reference source not found.**, Figure 8.There was no evidence for any of the outcome metrics that the extent of revision was different for different levels of probabilistic accuracy, as the extent of revision was distributed fairly evenly across the x-axis – this is consistent with findings from experiment 3.1.

## Section 9.6 Conclusions

The experiments described and implemented here use an innovative design to gain insights into how individuals express their knowledge using distributions and on how individuals revise their judgements when presented with other summary information (in a Delphi-type process). The experimental approach allowed the knowledge of each individual participant to be standardised, and this meant a structural (or mechanistic) understanding of how individuals express and revise probabilistic judgements could be acquired.

### Section 9.6.1 Key findings from the experiments

Experiment 1 imposed a knowledge base directly on the target quantity. Hence, and as expected, it showed no evidence of systematic bias across participants and no evidence of a differential effect of the methods on bias. However, it showed that participants do not adjust sufficiently their expressions of uncertainty: participants gave overconfident priors for the lower precision scenario and under confident priors for the higher precision scenario. Both methods performed similarly in lower precision, but bisection seems to generate more uncertain distributions in higher precision. KL divergence (a measure that combines both bias and uncertainty, and represents how well overall the elicited distribution represents the true distribution) presents high variability and hence throughout all experiment results, appears not to be sensitive to the changes in uncertainty detected in SD ratios.

Experiment 2 implemented a knowledge base that was relevant for the target question, but that required some adjustment (or extrapolation). Given that a single observation per participant was obtained, this experiment relied on a smaller number of observations than experiment 1. Experiment 2 generated no evidence that extrapolation, or its level, affects bias, expressions of uncertainty or overall accuracy.

However, it is difficult to give definitive messages from the experiment. It is possible that the experiments lacked power to detect the difference in accuracy when extrapolation is required. Furthermore, the experiment only explores one ‘type’ of extrapolation, where experts are required to derive a weighted average for two probabilities after observing all information required to make the adjustment. In practice, relationships between conditional probabilities can be more complex, and not fully observed; it is not clear whether the findings from the experiments would generalise when more complex extrapolation is required.

Experiments 3 looked at how and why individuals revise their answers when presented with a Delphi-type summaries. In experiment 3.1, individuals elicited for the target question and were then presented with a group summary that could be discordant with their own belief. Results show that participants were more likely to revise their priors when the group was discordant with their own beliefs, and when the group was more certain than they were. Also, participants were more likely to revise their priors when using chips and bins than bisection. There is no evidence that those who revised have a significantly different accuracy to those that who did not revise. Participants who revised, revised the mean of their priors to a greater extent when the group was discordant and when the group was more certain. When the group prior was concordant with the participant’s, the few who revised their priors on average became more certain. When the group prior was discordant with the participant’s, and equally uncertain, the participant was equally likely to revise his/her prior in both directions (some became more certain, others less certain). When the group was discordant and more certain than the participant, he/she was more likely to revise to express a more certain prior.

In experiment 3.2, instead of a group distribution, individuals were presented with the individual priors from the elements of a group that, overall, was discordant with the individual. Results show that participants were more likely to update their priors when the group members were consistent among themselves (although with wider within-participant uncertainty) than when the group elements are inconsistent among themselves (although more precise). Participants’ extent of revision was also affected with participants revising more (towards the group mean) if the elements of the group are concordant (but more uncertain) than if these are discordant (but more precise). When shown group members that were discordant amongst themselves, participants who revised their priors expressed less uncertainty. When shown a set of concordant priors, revisions went in both directions.

Overall, it is apparent that individuals changed their estimates in a rational way when provided with estimates from others (i.e. when everyone else was discordant, individuals were more likely to change their response, if others were uncertain, individuals were less likely to change); however, our results did not show large differences in the likelihood of revision between individuals with different levels of accuracy -- a key mechanism for revisions to lead to increased accuracy.

Our experiments did not explore the effect of group interaction on experts’ revision. Clarifying how group interaction affects the likelihood and extent of revision, and the accuracy of a group estimate would have to be carefully studied, controlling for aspects related to the format of feedback and effects of interaction.

### Section 9.6.2 Limitations and suggestions for future research (using the same experimental approach)

The three experiments implemented aimed to evaluate different aspects of elicitation, and used purposely restrictive set-ups (for example, experiment 1 imposed an equal knowledge base across individuals and the target elicitation referred directly to the observed quantity) to reduce between individual variation (i.e. random error). Whilst such set-ups may be seen as limited, these constitute a starting point whose design can be extended in the future to focus on other aspects important for real life elicitations. For example, the experiments implemented here used a simulated (virtual) learning process in isolation to determine the individual’s beliefs over the target quantity of interest, and in this way allow accuracy to be directly assessed. Whilst such learning from observation is a critical source of knowledge for practitioners in health care, our target experts, it is unlikely that, in practice, this is the only source of knowledge used by individuals in formulating beliefs, i.e. health carers may also draw on published evidence, peer contact or other related evidence or experience. Hence, a follow-on from these experiments could introduce other sources of information besides the simulated observations, to not only understand how individuals use multiple sources and whether this affects the accuracy of the distributions provided, but also to determine how to ask individuals to describe their reasoning for the judgements provided.

Another possible extension to this study relates to examining which individual’s characteristics may be associated with accuracy. The sample recruited for these experiments was homogeneous, and hence gives limited insight into how individual’s characteristics may be associated with accuracy. Whilst examining participant characteristics was not the focus of these experiments, future research could expand on the pool of experts to identify individuals that may be more accurate, and/or understand how individuals can be trained to become more accurate.^197^

### Section 9.6.3 Experimental approach vs. using almanac quantities

Published methodological research into elicitation has tended to focus on using ‘almanac’ quantities, defined as question that relate to uncertain events that will be realised in the future (e.g. rainfall tomorrow or survival of individual patients), facts (e.g. the distance between the earth and Mars) or to summaries of datasets (population or sample-based).^198^ Individuals are typically asked to express their subjective probabilities for these quantities and performance is measured as the deviation between the known value of the quantity and the elicited probabilities. Given the nature of almanac questions, beliefs about them may be formed on the basis of known facts coupled with analytical reasoning (for example, reasoning about the distance between Mars and Earth could be based on the knowledge that it would take a spacecraft one year to reach Mars).

The nature of almanac questions, however, differs inherently from the nature of the quantities typically elicited in health care and other areas where the typical target question relates to unknown parameters of a statistical model, quantities not expected to be known with certainty – for example, the expected probability that UK patients with a wound heal in 6 months when treated with X. Additionally, one of the main sources of information in determining the beliefs of substantive experts (particularly in health care) are observations of patients and their outcomes (e.g. from published studies or own direct observations). This evidence is itself uncertain and may also be biased, heterogeneous and lack generalisability (likely sources of concern in reasoning in HCDM).

Further, whilst individuals are expected to have some level of epistemic uncertainty about their answer to almanac questions, the accuracy of the elicited prior in representing this uncertainty cannot be established directly. Instead, multiple almanac questions are often used and the frequency of true values that fall outside the elicited credible regions is used as a measure of performance. Finally, responses to almanac questions can be inaccurate either if beliefs are themselves inaccurate or if, even with accurate beliefs, the individual struggles to express these in probabilistic terms. While the absolute accuracy of an elicited uncertainty measure cannot be determined using almanac questions, the *relative* accuracy of different elicitation methods, for example, could be compared by randomising individuals to different methods, since randomisation will ensure that any systematic differences between groups will be due to the elicitation methods. However, this may require a prohibitively large sample size.

# Chapter 10 Consideration of the methodological choices emerging from existing guidelines

## Section 10.1 Introduction

SEE is a process involving many different elements and subsequent methodological choices. A comprehensive list of these elements and choices has been developed and reported in *Chapter 2*. The intention of this chapter was to sift through the available choices with a view to building a reference-case for HCDM (see *Chapter 10*). The specificities of the domain area may help inform some of the methodological choices and so first, the set of principles that underpin the use of elicitation in HCDM is defined (Section 9.2). Each element of SEE then refers to these principles to describe the extent to which the available choices are appropriate for HCDM (Section 9.3). In doing so, it draws on evidence from the Chapters 3-8 of this report.

## Section 10.2 Principles underpinning the use of SEE to inform HCDM

A set of principles underpinning the use of SEE to inform HCDM were developed based on the findings from chapters 3-8 (referenced in Table 12). Each of these chapters contributes towards the elements covered in the principles. These principles also reflect considerations for SEE as reported by Cooke (1991)^14^, which reflect ‘good practice’ in SEE more generally and are widely referred to in the SEE literature. The full list of principles was refined to generate nine distinct principles. These are identified in *Table 132***Table 13** and detailed next.

**Table 13 Key principles of SEE in HCDM**

| **Principle** | **Key message** | **Source if evidence to support principle** |
| --- | --- | --- |
| **1. Transparency.** | SEE should be transparent and reproducible. | *Chapter 3* |
| **2. Fitness-for-purpose** | Elicited information should be fit-for-purpose to be used as an input to further analysis. | *Chapter 3*, *Chapter 4*, *Chapter 7* |
| **3. Consistency, but respecting constraints of the decision making context.** | The SEE needs to adapt to the practical and logistic constraints faced by different contexts/decision-making bodies, but maintain a level of consistency in methods used across evaluations. | *Chapter 3* |
| **4. Reflecting uncertainty at the individual expert level** | SEE must seek to elicit uncertainty in experts judgements. | *Chapter 8* |
| **5. Recognising and acting on biases** | SEE must recognise common expert biases and employ strategies to minimise these. | *Chapter 6* |
| **6. Suitability for substantive experts who are less likely to be normative** | SEE must utilise methods that are appropriate for experts with lower levels of normative skills. | *Chapter 3*, *Chapter 7*, *Chapter 8* |
| **7. Recognising where adaptive skills are required** | When required SEE must employ methods that incorporate or promote the adaptive skills of experts. | *Chapter 3*, *Chapter 5* |
| **8. Recognising between-expert variation** | SEE must attempt to capture any between-expert variation, understand the reasons why it exists, and explore its potential impact on the decision. | *Chapter 5* |
| **9. Promoting high performance** | SEE must motivate experts to best express their beliefs about a quantity of interest. | *Chapter 5* |

### Section 10.2.1 Principle 1. Transparency

*Chapter 3* surmised that, due to the fact that many SEE’s are conducted as part of a wider evaluation, e.g. cost-effectiveness modelling, word count limitations in journal articles often mean that reporting of SEE in HCDM is often insufficiently detailed.^72^ In many instances there is insufficient opportunity to report on the detail of the SEE, particularly the methodological choices made. Systematic and transparent reporting of SEE helps to improve the validity of the resulting expert judgements, allows the SEE to be peer-assessed, and supports others who use the judgements in their own analysis. Where there are word count limitations, a separate appendix should be used to report all details of the SEE, ideally comprised of an elicitation protocol, a summary of the conduct of the exercise, and of its results. More generally in Bayesian analyses, minimum reporting criteria have been published^199^. In addition reporting guidelines for SEE for model based cost-effectiveness evaluation have been published^72^, although these do not reflect any emergence of a reference protocol for SEE in HCDM, i.e. they may not reflect all the elements of SEE.

### Section 10.2.2 Principle 2. Fitness for purpose

*Chapter 7* showed that cost-effectiveness analysis, alongside other HCDM areas, typically requires judgements on a relatively large number of parameter types, including probabilities, transition probabilities, relative treatment effects, costs and HRQoL scores. This has implications for the quantities used to elicit each parameter as there is the need to i) ensure coherence between the multiple quantities elicited (i.e. to avoid dependency or explicitly elicit this) and ii) ensure these are adequate given the structural constraints imposed by the cost-effectiveness model and target population. Elicited information should therefore be fit-for-purpose to be used as an input to further analysis (e.g. disease modelling, risk assessment model or cost-effectiveness decision modelling).

### Section 10.2.3 Principle 3. Consistency, but respecting constraints of the decision making context

*Chapter 3* discusses the different potential audiences/analysts for SEE, from local level decision makers to national or international decision makers, including reimbursement agencies and research funders. These different decision makers have quite different capacities to conduct SEE and incorporate it into their decision-making processes. For many decision makers, SEE is very likely to be subject to constraints such as timelines, budget and availability of experts. This means that concessions on aspects of design and conduct of SEE are likely to be required. For example, fewer parameters may need to be elicited, a less time consuming method of elicitation may be needed (at the expense of exacerbating bias) or a remote exercise may need to be conducted. It is important that flexibility be retained in a reference protocol for SEE in HCDM, but that the implications of the choices made are explored.

### Section 10.2.4 Principle 4. Reflecting uncertainty at the individual expert level

As discussed in *Chapters Chapter 1* and *Chapter 4*, and explored in *Chapter 8*, judgements elicited from experts need to reflect the imperfect knowledge they have (referred to as epistemic uncertainty). An important concern is that, when reflecting on their own experiences, experts may instead include some level of variability in their judgements. Variability refers to the fact that individual responses to an intervention will differ between patients with the same observed characteristics within the population. A comparison of methods, chips and bins and bisection, to enable experts to express uncertainty as opposed to variability, is conducted in *Chapter 8*.

### Section 10.2.5 Principle 5. Recognising and acting on biases

As discussed in *Chapter 6*, there are many biases and heuristics (cognitive shortcuts that individuals often use when asked for complex judgements) that apply to SEE, including overconfidence/under confidence, over-extremity, discrimination, or susceptibility to base rate neglect. There are techniques available to reduce associated biases, which may help mitigate against their effect (see *Chapter 6*), however these have not been applied in the context of HCDM. Efforts should be made to integrate the findings and recommendations from behavioural research on what biases and heuristics can play an important role in SEE. SEE should be designed and conducted in a way that minimises the use of heuristics and other sources of bias and appropriate training should be given to experts.

### Section 10.2.6 Principle 6. Suitability for experts who possess substantive skills, who are less likely to be normative

*Chapter 5*, expert selection, concludes that substantive experts in HCDM are often health professionals, who are unlikely to have had extensive experience of quantifying their knowledge of healthcare outcomes, which may compromise their normative skills. They are, however, often subject experts and are recruited to take part in a SEE based on their substantive expertise. This is not typical of some of the other areas of science in which elicitation is commonly used; hence, methods of SEE employed in other domains may not be directly suitable in HCDM or additional training may need to be delivered before their use. For example, in the choice of method of elicitation e.g. graphical methods, such as the chips and bins method, have been claimed as more intuitive than the bisection method. Additionally, particularly in this context, it may be preferable to elicit only quantities that may be observable, and to recognise concerns over the elicitation of dependency.

### Section 10.2.7 Principle 7. Recognising where adaptive skills are required

*Chapter 5*, identifies very little evidence to clarify the role of adaptive skills, however, given the multiple purposes for SEE (*Chapter 1*), it is proposed that adaptive skills may be relevant in SEE for HCDM. In particular, it may be necessary to use SEE to inform HCDM in early cost-effectiveness modelling or early stage trial design. In this situation, experts may not be familiar with the target quantity/population for elicitation but are substantive experts in one or more related quantities. In this case, the SEE relies on the adaptive skills of experts and it is important that expert selection and/or training activities accommodate this.

### Section 10.2.8 Principle 8. Recognising and act on between-expert variation

*Chapter 5*discusses the issue of between-expert variation and the different methods for SEE, which deal with this variation (level of elicitation). In the context of HCDM, this variation is common; however, its causes are poorly understood. In the context of HCDM, there may be genuine heterogeneity in the populations experts draw upon to formulate their judgements and this may contribute to between-expert variation. In this case, it is desirable to reflect this variation in the pooled distribution, whether through group consensus or mathematical aggregation methods. There should also be efforts made to understand why between-expert variation is present, for example if this reflects heterogeneity in clinical observations such as patient severity. In some circumstances it may not be appropriate to combine judgements from experts where there is heterogeneity.

### Section 10.2.9 Principle 9. Promoting high performance

*Chapter 5* discusses the need to recruit experts that are motivated to undertake the SEE task optimally and that they have some kind of altruistic reason for providing their honest beliefs, i.e. to improve population health. In HCDM, experts may be motivated to undertake the task to the best of their abilities as a result of their interest in the topic area and improving population health through better HCDM. It may be the case, however, that not all experts within a SEE will perform as well. As well as promoting high performance a SEE may want to explore any differences in expert performance that emerge.

## Section 10.3 How do SEE elements and methodological choices reflect the principles underpinning healthcare?

This section considers the choices available for the different elements of SEE identified from the guidelines review (see *Chapter 2*). Not all principles for SEE in HCDM are relevant for all elements. The most relevant principles for each element and components within these are considered in the sections below.

These principles are applied to each of the choices, within components and elements, in the order in which they are presented in *Chapter 2*, with managing biases and validity overarching considerations throughout the process. Each section provides a summary of what choices the principles support. This is summarised presented for all components in the table in ***Error! Reference source not found.****.*

### Section 10.3.1 Selecting quantities (preparation and design)

Different quantities can be elicited that provide information on any single parameter of interest. There are a number of issues relevant when determining the choice of quantity to elicit. The choices for quantity are presented in *Chapter 2* and then considered in further detail in *Chapter 7*.

Key in HCDM is that the elicited information should be fit-for-purpose (principle 2) and describe experts uncertainty regarding the quantity of interest (principle 4). There is a lack of empirical evidence on whether to elicit directly observable or non-observable parameters in HCDM. In theory, so long as the elicited distributions can be used to provide information on the parameter of interest, either may be appropriate. However, experts in HCDM are often required for their subject expertise and they may be less likely to possess high levels of normative skills (principle 6). For this reason it may be advantageous to elicit less complex quantities which require high levels of normative skills, for example relative risks. It may also be relevant to consider how other empirical evidence are reported in the literature, i.e. how it is expressed statistically, particularly if synthesis with elicited quantities is required (principle 2). The applied literature tends to suggest observables are preferred in this context (see *Chapter 4*) and the existing guidelines consistently support this choice (see *Chapter 2*).

Adding another layer of complexity is the issue of dependent quantities. Where dependence exists between multiple elicited quantities, and experts can express it, it is appropriate to use dependence elicitation methods. Dependency can be elicited by expressing dependent variables in terms of independent variables or by eliciting conditional probabilities, and they have been used in this way in previous applications (see *Chapter 4*). More complex dependence elicitation methods such as regression-based techniques and other specialized techniques have not been applied in HCDM to date and it is unclear if these would be appropriate for HCDM experts (principle 6).

The choice of quantities of interest may also be guided by the practical constraints of the context.

In HCDM, there is often a need to generate quantities relatively quickly to inform decision-making (principle 3), perhaps reducing time available for training, particular on a face-to-face basis. In these circumstances, it may be advantageous to elicit dependent variables in terms of independent variables. In addition, when describing quantities, efforts to reduce cognitive burden on the experts, such as avoiding vagueness and asking questions in a manner consistent with how experts express their knowledge, may be preferable.

The principles support the following:

- Criteria to determine the choice of parameters, including minimal assessment of each possible uncertain parameter (sensitivity analysis) to identify which have the biggest impact (principle 3).
- Types of quantities: observable quantities such as probabilities (expressed as proportions or frequencies, but not more complex quantities such as higher moments of a distribution, odds ratios, or credible ranges (principle 2, 3).
- Dependency: ask only about independent variables, express dependent variables in terms of independent variables or use dependence elicitation methods (principle 6)
- Wording: avoid vagueness; ask questions in a manner consistent with how experts express their knowledge; use neutral wording, avoiding leading questions, decompose into simpler quantities where possible (principle 3).

### Section 10.3.2 Methods to encode judgements (preparation and design)

To inform HCDM, SEE should reflect the complexity of the further analysis it is informing, and of the other evidence supporting it (principle 2), for example the requirement to elicit for multiple parameters where there may be evidence of dependencies between them (see 9.3.1). In order to be practical in different contexts/decision-making bodies (principle 3), the methods used to elicit beliefs need to be easy to implement and not require extensive training. The suitability of the alternative choices must also recognise differences in normative skills of experts (Principle 6).

Existing guidelines suggest both the FIM and VIM to encode judgements. To date there has been a lack of empirical evidence on which method works better in this context, whilst providing accurate representations of experts beliefs, in particular of their uncertainty (principle 4). Both methods have been applied in HCDM (see *Chapter 4*). The experiments presented in *Chapter 8* sought to explore the use of these two methods in HCDM and compare them in terms of procedural performance, where there is no heterogeneity in knowledge. Little difference between VIM and FIM was found, particularly under conditions of low precision, the situation most likely in HCDM. There was a preference for the FIM by experts. This may be because the VIM requires experts to express their uncertainty using quantiles, which may be summaries of a distribution less familiar to experts. For both methods the expert should be trained to understand how to express uncertainty (principle 4).

The principles support the following choices:

- All forms of FIM or VIM: a decision maker can choose either but apply these consistently in their setting (principle 4, 6).
- Training: this should be provided to experts and focus on how to express uncertainty (principle 4).

### Section 10.3.3 Selecting experts

As part of the documentation (Section 9.3.15), the process for selecting and recruiting experts should be reported (principle 1); including details of the numbers of experts approached and the number that declined to take part (*see Section 9.3.15*, *Chapter 2,* and *Chapter 6*).

The existing guidelines suggest that features including normative expertise, substantive expertise and willingness to participate, can be used as defining characteristics to select experts. However, the constraints of conducting SEE in HCDM may dictate that the selection and recruitment of experts focus on only one or two key characteristics (principle 3). In particular, it is worth noting that health care professionals with the relevant substantive expertise may be limited in number, and therefore more opportunistic methods for recruitment may be required, such as peer nomination (see *Chapter 5* for examples). In some instances, adaptive skills may be required for a SEE, particularly in the case of new and emerging technologies (principle 7). The challenge in attempting to recruit experts that possess high levels of adaptive skill is that this characteristic is not well defined in the literature (see *Chapter 5*).

Defining an ‘unbiased’ expert poses a challenge and indeed, it may be impossible to do so (principle 5). *Chapter 6* suggests that the SEE can seek to recruit experts that are free from motivational biases by collecting disclosure of personal and financial interests and conflicts of interest. This may be a challenge as those with the greatest knowledge about a particular treatment or technology, and greatest willingness to participate, may be those with the greatest interest in the SEE. An alternative strategy therefore is to ensure that a range of viewpoints are represented in the sample, with the intention of “balancing out” or at least diluting the effect of motivational biases (see *Chapter 6*).

Between-expert variation may exist and the methods used to select experts must attempt to capture the range of plausible beliefs (principle 8). Identification of experts through recommendations by peers, either formally or informally, may generate a pool of experts that are all similar. Instead it may be preferable to recruit experts through research outputs, known experience or profile matrix. The SEE can also seek diversity in background, a balance of different viewpoints and a balance of internal and external experts. A larger number of experts may help to ensure that the selection of experts available fulfils these criteria (see *Chapter 2* suggests at least 5 experts).

The principles support the following choices:

- Selecting experts on the basis of their substantive expertise and willingness to participate (principle 3).
- Recruitment: recruit experts that are free from motivational biases where possible. In all instances collection information on personal, financial and conflicts of interest (principles 1, 5).
- Method to recruit: a range of methods are available to recruit experts. Which even method is used, this should strive for diversity in the pool of experts (principle 8).
- Number of experts: include at least 5 experts (principle 8).

### Section 10.3.4 Pilot exercise

All existing guidelines agree that a SEE should include a pilot of the exercise and omitting a pilot could actually cost time rather than saving it (principle 3). The pilot can be used to explore which method best reflects uncertainty at the individual level. If the training is also piloted, the analyst/facilitator can also use this opportunity to gain feedback from experts on how capable they felt using methods to express their uncertainty (e.g. VIM or FIM) and make revisions to the SEE if required (principle 4).

The pilot can also be used to determine the appropriateness of the SEE for those experts recruited, particularly if the sample of experts have low levels of normative skills (principle 6). This can involve piloting of alternative ways of formulating the questions, which quantities are used (see Section 9.3.1) or the method to encode judgements (see Section 9.3.2).

The principles support the following choices:

- Piloting: this should be undertaken prior to the task. Use of feedback to revise the SEE (principles 3, 4, 6)

### Section 10.3.5 Training and preparation for experts

A proportion of the SEE should be spent on delivering training, as it is unlikely that HCDM experts will have had any previous experience of SEE. Training and preparation should focus on enabling non-normative, but substantive experts, to express their beliefs appropriately (principle 6). This should focus on giving them the tools and information to express their uncertainty at the individual level (principle 4). Non-normative experts may be wary of the SEE task and this may have implications for how confident they are at expressing their beliefs. Some experts may express over confident distributions for fear of being judged (see *Chapter 6*). Training therefore plays a role in minimising biases (principle 5), and, although the evidence, in the context of HCDM, is weak, there are some suggestions from the literature that training can be efficacious in reducing the effect of anchoring, adjustment in interval, confirmation bias and overconfidence (see *Chapter 6*).

The existing guidelines (see *Chapter 2* do not provide a definitive list of what should be covered in training and the elements included will be driven, in part, by the specific application, for example description of quantities, description of performance measurement and dependence. Some elements, such as how results will be used, motivation of elicitation and the full protocol, may not be possible to include due to the time constraints likely in HCDM (principle 3). In addition, a list of relevant information is only typically used as part of a group process or where there are efforts to standardise the level of substantive skills across experts. The (core elements): description of what is required from experts, outline of process, outline of questions, example and practice questions and assumptions and definitions used in the elicitation, should not be compromised (see Section 9.3.9).

The principles support the following choices:

- Training: this should be delivered and should focus on: 1) enabling experts to experts their uncertain belief, 2) minimising bias (principles 3, 4, 5, 6).

### Section 10.3.6 Level of elicitation (elicitation)

Judgements from multiple experts are preferred in a SEE (see Section 9.3.3). Existing guidelines are inconsistent w.r.t the level of elicitation – individual or group based. Group discussion can aid less substantive and normative experts, however, face-to-face discussion can be resource and time intensive (principle 3). Access to trained facilitators for group level elicitation may be scarce within HCDM (see *Chapter 3*).

Interaction between experts can also introduce biases (see Section 9.3.16) (principle 5). The act of striving for consensus can potentially eliminate some of the between expert variation; the potential for ‘groupthink’ (see *Chapter 6*). A group process should aim to reflect both individual level uncertainty and between expert variability in the aggregated distributions (principles 4 and 8) and there may be greater potential to explore variation in experts beliefs with a group based approach, but only if face-to-face. In HCDM, there may be a lack of experienced facilitation, thus it may not be possible to do this. In these circumstances, an individual level elicitation may be more appropriate. A large sample, which may be required to ensure representativeness, may be a challenge for a group-based exercise, particularly if face-to-face. An individual level elicitation can ask experts to express how they formulate their beliefs, however it is a challenge to them incorporate these differences into the resulting aggregate distribution.

Group elicitation via remote means may be practical in some circumstances. As discussed in Section 9.3.11, interaction between experts can be beneficial for non-normative experts (principles 6 and 9). Remote group elicitation can help to mitigate against dominate experts. Individual level elicitation, whilst avoiding this situation, can be daunting for experts that have not undertaken such tasks previously (non-normative).

The principles support the following choices:

- Level of elicitation: elicit from experts individually (principles 3, 4, 8).
- Role of consensus: where required, should first conduct individual elicitation followed by group consensus (principles 6, 9).

### Section 10.3.7 Mode of Administration (elicitation)

A number of alternative modes of administration have been used in HCDM (see *Chapter 4*), however many of the existing guidelines agree that face-to-face administration is preferred (see *Chapter 2*). It is thought to promote good performance (principle 9) and maximise engagement with experts. Face-to-face elicitation is required for some consensus methods (*Chapter 5*), however it is not necessary for a mathematical approach.

The constraints in HCDM (principle 3) are the biggest factor in driving the method chosen. If a large number of experts is sought (see Section 9.3.3), in order to generate timely results, face to face elicitation may be prohibitively time and resource expensive. The constraints of HCDM do not imply that a particular vehicle is used, i.e. paper based on computer based questionnaire, however in order to record information elicited effectively, the majority of applications in the context have used a computer based exercise, either developed for that unique purpose or using existing ‘off the shelf’ software (see *Chapter 4*).

The principles support the following choices:

- Administration: can conduct SEE using face-to-face or remote administration (principles 3, 9).

### Section 10.3.8 Feedback to experts and revision (elicitation)

Feedback and opportunity for revision can be used as a strategy to minimise bias (principle 5 and *Chapter 6*). The guidelines are consistent in recommending that feedback and opportunity for revision takes place but differ w.r.t what to feedback. The process should be made explicit and documented appropriately; including the number of feedback rounds and what is fed back (principle 1).

For non-normative experts (principle 6) graphical feedback could be useful, whereas more complex summarises such as fitted distributions, performance scores or results using elicited values may not be appropriate. Experts may find distributions from other experts, summaries of aggregated distributions, rationales, future data, the draft elicitation report or qualitative discussion of elicited values, useful, however it is not clear how these could improve the SEE unless they are accompanied by the opportunity for revision. If the feedback allows experts the opportunity to revise their distributions, it may be a useful process as this can help to promote high performance and distinguish between high and low-performing experts (principle 9).

Feeding back distributions of other experts is common in group-based approaches (see *Chapter 5*) and it may incentivise less high performing experts to revise their distributions; however this may be driven by how uncertain they are about their beliefs. There is also the possibility that high performing experts will also revise their distributions, potentially generating less accurate pooled or group summaries.

The principles support the following choices:

- Feedback: this should be offered to experts with the possibility of revision. What to feedback will depend on the SEE task and the types of experts include. Graphical feedback may be useful for non-normative experts (principles 1, 6, 9).

### Section 10.3.9 Opportunity for interaction (elicitation)

Interaction is intrinsically linked with the level of elicitation and as such, many of the principles relevant for Section 9.3.6are relevant for how the interaction process works. Interaction can allow experts to share information so that differences in expert opinion are not the result of experts having different information or interpreting questions differently (principle 9). In addition, it is important to note that, remote and controlled interaction, such as that promoted with Delphi type processes, can avoid some of the biases of group exercises (principle 5) and can be preferable from a practical point of view (principle 3). However, remote elicitation can encourage experts not to take responsibility for their expressed beliefs (self-serving bias). As with group and individual methods, there is also a lack of evidence on how the revision process can affect the accuracy of the final individual distribution (principle 9). For consensus SEE, a group based face to face session may help to promote the beliefs of experts with better performance and reflect between expert variation (principles 8 and 9) (*Chapter 6*).

The principles support the following choices:

- Interaction: this should follow on from an individual elicitation where practically feasible and useful (principle 9).

### Section 10.3.10 Feedback from experts on process (elicitation)

In addition to feedback from the facilitator and/or other experts (Section 9.3.8), a SEE can encourage feedback from experts on the process, either qualitatively through an interview or questionnaire, or through some kind of quantitative ranking. This is linked to obtaining rationales (Section 9.3.11), however more broadly relates to the elicitation process rather than the beliefs about quantities. Only a limited number of guidelines discuss obtaining this type of feedback (see *Chapter 2*) and a limited number of applied studies have attempted to collect this information in HCDM (see *Chapter 4*). Given the lack of practical experience and empirical evidence, it is difficult to be prescriptive about how this type of feedback might work in the context of HCDM and it may be driven by the time and resource constraints of the task (principle 3). It may be valuable to ascertain what elements of the SEE experts found challenging. This information could then be used in designing future exercises (principle 1). In addition, the information gleaned from experts during the feedback could be used to discriminate between low and high performers (principle 9), however this would be on the basis of their subjective assessment, rather than on the basis of a quantitative measures of accuracy such as calibration (see Section 9.3.14andSection 9.3.17). Overall it is not clear how this form of feedback would be beneficial to the SEE task or improve the resulting distributions.

The principles support the following choices:

- Asking experts for feedback: should only ask the experts to appraise the SEE process if there is a clear reason for doing so (principle 1).

### Section 10.3.11 Rationales (elicitation)

Almost all guidelines recommended collecting qualitative data from experts on how they formulated their judgements. Experts in HCDM may possess different levels of normative and substantive skills and the setting in which they work may expose them to different clinical experience, which can drive their beliefs. It is therefore important that the rationales for the beliefs given are collected (principle 1). This information can then be considered. This can inform an assessment of validity of the elicited beliefs.

The methods used to collect rationales will be driven by the mode of administration (seeSection 9.3.7). Interaction between the analyst and the expert on a one to one basis can encourage experts to explain in greater detail their rationales. Where SEE is conducted remotely it may be advantageous to use prompts such as multiple choice questions to encourage experts to reveal any detail about their rationales.

The principles support the following choices:

- Rationales: these should be collected and recorded from experts about how they made their judgments (principle 1).

### Section 10.3.12 If/how to aggregate (aggregation, analysis and post-elicitation)

In order to generate distributions that are fit for purpose (principle 2), aggregation is preferred over no aggregation. With respect to the choice of aggregation method, *Chapter 5* concludes that, on the basis of the evidence available, in terms of ‘accuracy’ including representation of uncertainty, mathematical and behavioural aggregation perform similarly. There is also no evidence to support the specific type of behavioural aggregation method used. For mathematical aggregation, simple mathematical decision rules, like a linear opinion pool with equal weights are the most commonly applied in HCDM (see *Chapter 4*) and are straightforward to implement (principle 3). Mathematical approaches allow experts to express their uncertainty and then, if appropriate aggregation approaches are used, this feeds through into the overall distribution achieved (principle 4).

Mathematical aggregation does not require experts to converge to a group distribution, therefore allowing variability between experts to be reflected within an overall distribution, either using opinion pooling or Bayesian methods (principle 8). A mathematical process can elicit the reasons for the distributions expressed, however it cannot use these quantitatively in generating a single overall distribution, unless the reasons for these distributions are reflected in the seeds that are generated as part of a calibration process (principle 8, seeSection 9.3.17). Calibration-based performance weighting, which has received little attention in the HCDM literature (see *Chapter 5*) ‘solves’ any between-expert variation in performance by differentially weighting experts according to their performance on ‘seed’ scores (see *Chapter 5*). Generating differential weights in HCDM, is, however, problematic as discussed in *Chapter 5*. Further research on weighting methods within HCDM is needed to advise if and when choices beyond equal weighting are warranted.

The principles support the following choices:

- Aggregation: Mathematical aggregation or individual elicitation followed by behavioural aggregation (principle 4, 8).
- Method of aggregation: Use of linear pooling methods (principle 8), including equal weighting of experts distributions (principle 3).

### Section 10.3.13  Fit to distribution (aggregation, analysis and post-elicitation)

As part of the aggregation procedure, post elicitation, statistical distributions need to be fitted to elicited data (see *Chapter 5*). The choice of parametric distribution is uncertain. There is a lack of evidence in HCDM on the fitting process in SEE. Limited evidence suggests that standard distributions, such as the Beta will often be sufficient. More complex approaches may be appropriate, however these can be complex to implement in general software (principles 2 and 3).

The fitting process should ensure that uncertainty, at the individual expert level, is reflected (principle 4) and to do this the distribution used should capture the experts distributions as closely as possible. It is also important that the aggregation respects between-expert variation (principle 8). It is difficult to be prescriptive about which distribution is most suitable, as this will be driven by the quantity elicited and how experts have expressed their beliefs, i.e. the shape of the distribution, however the resulting distributions must generate quantities that can be used within further analysis (principle 2), for example, without transformation.

The principles support the following choices:

- Fitting: distributions should be fitted to experts elicited beliefs (principle 2).
- Which distribution: this will depend on the quantity and how the beliefs are represented, however distributional forms such as normal, beta and other conjugate family will often be appropriate (principle 2, 3, 4).
- Fitting criteria: the use of minimum least squares, method of moments or other approaches, to select the appropriate distribution (principle 2, 3, 4).

### Section 10.3.14  Adjusting judgements (aggregation, analysis and post-elicitation)

Experts may possess differential levels of normative, substantive and adaptive skills, which may result in differential performance. None of the existing guidelines discuss methods to adjust for ‘performance’ post elicitation (see *Chapter 2*) even if they do refer to a validation process (see Section 9.3.17).

The methods used for SEE should motivate experts to express their true beliefs about a quantity of interest and quantify differential performance between experts (principle 9), implying that adjusting judgements is preferred to not adjusting, if it generates more accurate pooled distributions. Without objective measures to quantify performance, however, adjustment may instead resolve variability between experts, which is not desirable in HCDM, where variation may exist for valid reasons (principle 8).

The principles support the following choices:

- Adjustment: this should not focus on simply reducing variability between experts (principle 8).

### Section 10.3.15 Documentation (aggregation, analysis and post-elicitation)

In order to inform an explicit decision making process in healthcare a SEE must report on all elements of the process and justify the choices made in determining these choices (principle 1). There is no agreed list of what should be presented, emerging from the existing guidelines (see *Chapter 2).*However, recently, guidance, not described as a guideline, reported on what information should be in HCDM (see *Chapter 4*). Iglesias, et al^72^ suggest 16 criteria for a SEE and 11 criteria for a Delphi study specifically. These largely accord with the items identified from the existing guidelines, although for Delphi surveys they also suggest a description of the literature review and the number of rounds performed. They do not specifically advocate reporting on details of how uncertain quantities are measured (VIM, FIM) or any training methods used.

It is important to note that many applications of SEE are conducted alongside cost-effectiveness modelling or some other form of evaluation, and therefore the amount of material that could be reported may be vast. *Chapter 4* showed that, as a consequence, many details of a SEE are often omitted from a published manuscript or report. It may therefore be advantageous to specify a minimal set of documentation, such as that suggested by Iglesias, et al^72^.

The principles support the following choices:

- Documentation: this should be thorough and covers all aspects of the SEE design, conduct and analysis (principle 1).

### Section 10.3.16 Managing biases

In striving to minimise bias, efforts should be made to identify which biases are likely for the sample of experts included (principle 1), and relevant strategies to minimise these (bias reduction techniques) should be employed (principle 5). *Chapter 2* does not suggest specific techniques for addressing individual types of biases or heuristic, and instead gives multiple suggestions across the range of biases.

*Chapter 6* suggests that it is difficult to recommend particular bias-reduction techniques over others, as what works best will depend on the context and what biases are most apparent. Given the recommendations for training made in Section 9.3.5, it would seem appropriate to extend this training to cover issues of bias, but going beyond simple warnings. Allowing experts to practice expressing their beliefs using either VIM or FIM, followed by feedback, may also reduce the probability for some of the biases.

The principles support the following choices:

- Anticipate likely biases: for the sample of experts included and specific task. Discussion with experts can help to identify potential biases (principle 1, 5).
- Frame questions to minimize bias and ambiguity. This can include asking experts to first specify the credible interval (upper and lower bounds) and provision of relevant background evidence (principle 1, 5).
- In selecting experts: minimize and record conflicts of interest among the experts. Include experts external to the SEE task, i.e. not those involved in developing the task (principle 1, 5).
- Focus training: on biases and expressing uncertainty and give experts practice and feedback using either the FIM or VIM (principle 1, 5).
- During the task: experts should address conflicting information and provide their rationales (principle 1, 5).

### Section 10.3.17 Validation

The guidelines differ in their definitions of validity and discussion of how the concept can be operationalised in an elicitation. Commonly discussed elements of validity include that the elicitation captures what experts truly believe or that the expressed probabilities reflect reality. Certain elements of validation accord with the section relating adjusting judgements (see Section 9.3.14), however a number of existing guidelines describe validation of the process rather than the results of SEE. The method used for validation should strive to explore the implications of between-expert variation and attempt to understand why it is present (principle 8).

Understanding how experts formulate their beliefs and why experts present heterogeneous beliefs, can potentially improve the validity of the SEE (principles 1 and 8). The following choices could fulfil this purpose: provision of feedback, testing that the question is understood, fitness for purpose, assessing the accuracy of judgements (see *Chapter 5*), coherence testing, rationales, checks for inconsistencies and internal and external peer review. Faithfully capturing experts beliefs should always be the aim of SEE; however where there is no data to explicitly validate this, there is no way of checking if the resulting distributions represent experts beliefs.

Fitness for purpose (principle 2) states that the validation process should generate distributions that can be used in HCDM. To this end one of the possible validation processes described by the review of guidelines is fitness for purpose, which evaluates if the elicitation process provides an appropriate level of precision for the given decision context. Internal and external review can also be used to determine if the resulting distributions are valid. It is not clear how the other methods of validation, calibration, coherence, consistency, calibration and informativeness scoring, can be used to determine if the SEE generates useable distributions.

The principles support the following choices:

- Capturing experts beliefs: the elicited beliefs should be fit for purpose. This could be assessed by coherence and consistency (principle 1, 2).
- Review: both internal and external review (principle 2, 8)

## Section 10.4 Conclusions

This chapter considers the choices available from the review of existing guidelines for SEE (see *Chapter 2*) and distinguishes where there is empirical support for the choices, the choices are considered ‘appropriate’ according to the principles for SEE in HCDM, or where there is neither support from the empirical evidence nor the principles. *Chapters Chapter 5* and *Chapter 6* show that there many choices in SEE, for which there is no empirical support. In addition, the principles applied to the choices, in some circumstances, are unable to provide sufficient justification for discounting particular choices and/or preferring choices above others. For example, on the methods to minimise bias, multiple approaches are available, including training on biases, collecting rationales, and specifying credible intervals. Whilst all of the approaches are potentially valuable, a lack of empirical comparison of the techniques in the context of HCDM, makes it difficult to say conclusively which techniques are most appropriate. Indeed, as with many of the choices in HCDM, the specific application and constraints (see *Chapter 3*) may be a major driving factor in defining the choices for the SEE.

# Chapter 11  Reference protocol for expert elicitation in healthcare

## Section 11.1 Evidence in support of a reference protocol for HCDM

As stated in the objectives outlined in *Chapter 1*, existing protocols, evidence on specific methods for SEE, consideration of the decision making contexts and the results of the experimental work are combined to propose a reference protocol for SEE in HCDM.

*Chapter 9* considers the choices available from the review of existing guidelines for SEE and concludes that, according to the principles for SEE in HCDM, in some circumstances, it is not possible to conclude on the appropriateness of particular choices. One of the uncertain elements is the method to encode judgements, specifically the choice between FIM and VIM approaches. The applied literature on SEE in HCDM (see *Chapter 4*) shows that both approaches have been used, and there are no grounds, based on the principles, to conclusively recommend one choice over another. The experiments presented in *Chapter 8*sought to explore the use of these two methods in HCDM as a primary aim. The chips and bins method was chosen as the FIM and the bisection as the VIM, since these methods have been widely used in HCDM. The experiments also explored experts ability to extrapolate their knowledge and how experts priors are affected by group summaries. Specifically the three experiments sought to determine:

1) How the VIM and FIM methods compare in term of procedural performance, where there is no heterogeneity in knowledge.
2) Understand whether an individual’s ‘ability to extrapolate’ is related to (‘procedural’) performance.
3) How individuals review their answers in response to a Delphi-type group interaction, and establish whether (‘procedural’) performance determines the extent of revision.

*Chapter 8* suggests that there is little difference between VIM and FIM in terms of procedural accuracy, particularly under conditions of low precision, the situation most likely in HCDM. In terms of extrapolating beyond the data observed by the experts and updating of priors after presentation of group summaries, it is difficult to give definitive messages given that the experiments were not powered for these elements. It is apparent that individuals changed their estimates in a rational way when provided with estimates from others (i.e. when everyone else was discordant, individuals were more likely to change their response, if others were uncertain, individuals were less likely to change), and so group discussion or feedback may be useful, although it does not necessarily produce more accurate distributions. The need for extrapolation outside of the observed sample and the level of extrapolation does not seem to affect accuracy; therefore it may be reasonable to ask experts about patients and practices in which they do not have direct clinical experience, or for whom there is no relevant literature, and instead experts are required to adapt from one setting to another.

## Section 11.2 How the evidence is used to generate a reference protocol for SEE in HCDM

For many of the elements of SEE, multiple choices remain and further research would be necessary to form a preference for different methods in the context of HCDM. Nevertheless, it is important to recognise where there are choices that are emerging as ‘best practice’ in HCDM, and how these contribute towards the development of a reference protocol in this context.

Within HCDM there are multiple opportunities for the use of SEE, from local-level prioritisation to strategic planning for emerging threats. The areas in which it has, perhaps, been applied the most frequently (see *Chapter 4*) is in national level reimbursement, price negotiation and clinical guideline development, an area collectively referred to as HTA. It therefore seems appropriate to think about how the evidence presented, considered and generated in *Chapters 2-9* could be translated into a reference protocol for SEE in HTA. Moving on from this, how decision makers, outside of this setting, can determine the suitability of the reference protocol for their needs can then be discussed. Where substantial uncertainty around recommendations remains, further research may be required. These are considered in *Chapter 12*.

## Section 11.3 Reference protocol for SEE in HTA

SEE has been applied in HTA (see *Chapter 4*). However, there are no examples where those developing the exercise have systematically worked through the choices available for each element, and most importantly considered if these choices are appropriate given the intended purpose of the SEE. A reference protocol, even with caveats for particular applied settings, may help to eliminate some of this heterogeneity in methods used.

*Table 14*3 draws on *Chapters Chapter 8* and *Chapter 9* to suggest choices that are appropriate to consider in HTA, specifically assessments at a national or multinational level. Whilst these are intended to reflect emerging ‘best practice’ in HTA, given the infancy of SEE applied to HCDM, it is important to recognise that a degree of flexibility on choices may be warranted. In cases where alternative choices are employed, efforts should be made to justify why and describe where the methods used were preferable in that particular application. Although empirical evidence is lacking, given the principles of SEE in HCDM, discussed in *Chapter 8*, decision makers should consider the following choices when determining their own reference protocol for SEE:

**Table 14 A reference protocol for HTA**

| **Element** | **Reference methods suggested** |
| --- | --- |
| Experts | 1. Recruitment will be driven by the context, however the SEE should pursue diversity, representing the full range of valid experts beliefs. Experts should be willing to participate.  2. Focus on gathering substantive expertise or experience. Normative skills can be developed during the training session as part of the SEE.  3. Minimize and record conflicts of interest among the experts. Include experts external to the SEE task, i.e. not those involved in developing the task.  4. At least 5 experts should be included in the SEE. |
| Quantities elicited | 1. Simple observable quantities should be elicited where possible; ratios or complex parameters such as regression coefficients should not be elicited directly.  2. Dependence between variables should be captured in SEE. Expressing dependent variables in terms of independent variables is preferable when experts do not have strong normative skills.  3. Wording should be clear and quantities should be decomposed where this means a better fit with experts mental models. |
| Approach to elicitation | 1. Beliefs should be elicited from experts individually, even if a group interaction follows.  2. Although interaction between experts can be structured through face-to-face sessions, constraints in HCDM, such as a lack of experienced facilitators, will usually mean that this will take place via a Delphi style remote process.  2. Between-expert variation should be explored explicitly. |
| Method | Both VIM or FIM work well, however decision makers should aim for consistency across applications. |
| Aggregation | 1. Statistical distributions should be fitted to experts individually-elicited judgements.  2. Following fitting, a summary of the individual distributions should be obtained using linear pooling with equal weighting of experts.  3. Any adjustments applied should be to improve coherence and consistency not reduce variability. Internal and external review can be used to assess validity. |
| Delivery | 1. Face-to-face where possible to allow a facilitator to deliver training to the expert.  2. Feedback to experts should be given during the SEE. Following feedback, experts should be given an opportunity to revise their distributions, either during or after a SEE session. |
| Training & piloting | 1. Training is crucial and should focus on avoiding bias and expressing uncertainty.  2. Piloting should be undertaken. |
| Rationales & documentation | 1. Rationales for how the experts made their judgements should be collected post SEE.  2. All methodological choices for the SEE must be documented and justified. |

## Section 11.4 Important considerations for decision makers outside of the HTA setting

Most HCDM occurs within a HTA setting and at a national level, but elicitation may also be useful for other decision makers, wishing to consider how a reference protocol for their setting may emerge, for example at a local level, or for early technologies that have yet to progress through the regulatory process. In addition, particular types of HTA may encounter additional challenges, for example in rare diseases or genomics. In such settings, a potential reference protocol should consider the following additional issues summarised in *Table 15*4.

**Table 15 Additional issues in generating a reference protocol outside of HTA**

| **Element** | **Reference methods suggested** |
| --- | --- |
| Experts | 1. Researchers may have limited access to sufficient experts, for example in rare diseases, therefore expert recruitment may be more challenging and have to rely on peer nomination.  2. Adaptive skills may be required for new technologies since indirect evidence may outweigh directly relevant evidence (e.g. childhood diseases may be informed by adult versions with some extrapolation). |
| Approach to elicitation | Group discussion may be needed to generate a distribution, for example in early technologies or when eliciting more abstract/complex (non-observable) quantities cannot be avoided, for example relating to service delivery, public health programmes or patient pathways. |
| Method | FIM may be more appropriate for less normative experts or where training cannot be done face-to-face |
| Aggregation | 1. Pooling methods, other than linear pooling, may better reflect expert variability. Further research is needed to explore which methods are more appropriate in these circumstances.  2. Weighting may be preferable in some circumstances, for example where experts represent different disciplines or contribute different perspectives on the elicited quantities and therefore considerable heterogeneity is anticipated, but a single agreed consensus distribution is required. Weighting may be achieved implicitly through consensus or explicitly through performance weighting, although it is difficult to see how performance scores would be generated in this context. |
| Delivery | Practical constraints may dictate remote delivery of SEE, for example though video conferencing. |

## Section 11.5 Conclusions

This chapter draws together evidence from the preceding chapters to generate elements for a reference protocol for SEE in HTA. Given the infancy of the methods in HCDM and the limited application in this context, it is not possible to be prescriptive regarding methods beyond the more narrowly defined HTA setting. Even within this setting, the reference protocol provides a framework for decision makers to use when generating their own reference protocol, rather than representing a set of guidelines that can be implemented without further consideration of their suitability. Whilst this is the case, given that the methods suggested in this reference protocol are declared most appropriate for HTA on the basis of its defining characteristics, which determine the principles for SEE in HCDM, deviations from the reference protocol should be justified and any limitations discussed in the documentation provided to support the SEE.

There are a number of methodological choices which may involve additional complexities and/or considerations, when used outside of HTA. These are discussed in this chapter and then further in *Chapter 12*. Such choices include the use of consensus aggregation methods, as opposed to individual elicitation, and remote elicitation as opposed to face-to-face. Decision makers outside of HTA at a national level, are recommended to consider these issues when generating reference protocol.

Finally, this chapter proposes a number of areas in which further research is warranted. This is not a comprehensive list and instead reflects important areas in which the existing reference protocol cannot make recommendations without further research. Some of these areas may require further practical applications of SEE, such as strategies to recruit experts, whereas others may require experimental research, such as that reported in *Chapter 8*.

In addition to the specific methods that require further research, there are some general issues relating to the use of SEE in HCDM; for example, when to elicit and in which areas it is most appropriate. These issues are discussed in *Chapter 12*.

# Chapter 12 Applied evaluation of developed reference protocol

## Section 12.1  Background

This chapter demonstrates the reference protocol described in *Chapter 10* by conducting an applied SEE. Originally, the intention of this chapter was to apply the developed reference protocol by performing an elicitation exercise within a decision making process in ‘real time’. Members of the research team form an Assessment Group for NICEs technology and diagnostic assessment processes and thus intended to conduct a live elicitation exercise to inform a forthcoming appraisal. However, at the time the reference protocol was developed and ready to be applied, there were no upcoming appraisals with which the team could conduct the elicitation exercise. Consequently, there was a deviation from the original intention of a ‘real time’ elicitation exercise and the reference protocol was applied in a retrospective manner.

This applied evaluation is based on a diagnostic assessment report (DAR) conducted in Sheffield in the School of Health and Related Research (ScHARR).^200^ ScHARR were commissioned by the NIHR HTA programme to produce a model to assess the diagnostic accuracy, clinical effectiveness and cost-effectiveness of three handheld Fractional Exhaled Nitric Oxide (FeNO) monitors: NIOX MINO (Aerocrine), NIOX VERO (Aerocrine) and Nobreath (Bedfont Scientific).^200^ The analysis aimed to assess the cost-effectiveness of FeNO testing in the diagnosis of asthma in adults and children. However, in the cost-effectiveness model, there were a number of parameters that were missing which were subsequently estimated using SEE. Detailed documentation describing the methods used to obtain and analyse these experts judgments are not included in the report. Without this information, the elicitation process appears to be unstructured, meaning the credibility of the elicited parameters remains unclear. The purpose of this chapter was to apply the reference protocol to this case study and to explore any practical issues.

## Section 12.2 The evaluation topic

Here the focus is on a model developed to assess the cost-effectiveness of NIOX MINO, NIOX VERO or NObreath in the diagnosis of asthma in adults and children. In order to illustrate the patient pathways in asthma diagnosis, the next section describes how asthma is currently diagnosed in healthcare.^200^

### Section 12.2.1 Diagnosis of asthma

Detailed guidelines on the diagnosis of asthma have been published and updated by the British Thoracic Society (BTS) and the Scottish Intercollegiate Guidelines Network (SIGN).^200^ The diagnosis of asthma is a clinical one and there is no standard definition of the condition, nor is there a single gold standard recommendation on how it should be diagnosed. The diagnosis of asthma in children is based on recognising a characteristic pattern of episodic symptoms in the absence of an alternative explanation. Lung function tests are less useful due to variability and the inability of very young children to perform these tests reliably. For both children and adults, the BTS/SIGN guidelines indicate that the severity of asthma should be judged according to symptoms and the amount of medication required to control symptoms.^200^ Asthma is generally diagnosed in primary care.^200^

### Section 12.2.2  Diagnostic model developed

The diagnostic model determines the expected costs and health losses associated with the misdiagnosis of asthma. Misdiagnosis has different implications for those patients who are false-negative and for patients who are false-positive. For patients who are false-positive, sub-optimal treatment means receiving treatment with asthma medication which will provide no health benefit to the patient (because they do not have the underlying disease). This means there is an additional cost to the NHS without additional health benefits to the patient. In addition, a patient with a false-positive asthma diagnosis may have other more serious pathology, which remains undetected.^200^

For patients who are false-negative, sub-optimal treatment means not receiving treatment with asthma medication, when in reality the patient would have benefitted from the treatment. Until the diagnosis is corrected, the patient may suffer from poor asthma control and hence lower HRQoL due to asthma symptoms (without experiencing an exacerbation and also by increasing the amount of the time that a patient experiences an exacerbation). Hospitalizations due to exacerbations can be costly to the NHS; hence, a patient with undiagnosed asthma may be more costly to the NHS than a patient who is correctly treated for asthma. These patients may also go on to receive expensive and unnecessary tests such as imaging and referrals to specialists until their misdiagnosis is corrected.^200^

An incorrect false-negative diagnosis may be corrected later following an asthma exacerbation, due to continued asthma-related symptoms that trigger subsequent appointments and investigation, or due to clinical reconsideration of asthma after tests for other conditions produce negative findings. Similarly, an incorrect false-positive diagnosis may be corrected later due to the continued non-occurrence of exacerbations, a generally high level of HRQoL at very low treatment dosages, thus indicating that medications are currently being taken by the patient may be unnecessary, or due to continued deterioration due to other more serious underlying pathology. The diagnostic model is intended to reflect the implications of test sensitivity and specificity on subsequent costs and health consequences for the full range of diagnostic options within the available evidence base.^200^

The diagnostic model is a simple decision tree (***Error! Reference source not found.***, *Figure 1*). The model estimates the probability that a patient will be diagnosed as true-positive, false-negative, true-negative or a false-positive. The model makes the simplifying assumption that incorrect diagnoses (false-negative and false-positives) are resolved by subsequent tests after some period of time.

## Section 12.3 Description of elicited parameters

*Table 16*5 shows the parameters in the diagnostic model for which evidence was unavailable. In the DAR, the parameter values are provided but there is no documentation detailing how these values or assumptions were reached. The parameter for *time until correct diagnosis* is the only parameter for which the DAR explicitly states that an elicitation process was used to inform these parameters. Subsequently, the remainder of this evaluation focusses on that parameter only.

**Table 16 Application of reference protocol: Parameters elicited in DAR^200^**

| **Parameter** | **Source** |
| --- | --- |
| **Diagnostic model parameters** | |
| (1) Resource cost parameters | |
| Number additional primary care tests: false-positive | Structural assumptions based on expert opinion |
| Number additional secondary care tests: false-positive |  |
| Number additional laboratory visits: false-positive |  |
| Number additional primary care tests: false-negative |  |
| Number additional secondary care tests: false-negative |  |
| Number additional laboratory visits: false-negative |  |
| (2) Diagnosis QALY gain/loss parameters | |
| Time until correct diagnosis (years) – false positive | Expert opinion |
| Time until correct diagnosis (years) – false negative |  |

Source: From Table 64 in the DAR –page 284

### Section 12.3.1 Time until correct diagnosis

As described in the DAR and in Section 11.2.2of this chapter, a false-negative or a false-positive diagnosis of asthma impacts on HRQoL costs, depending on the type of diagnosis. The cost effectiveness results are particularly sensitive to assumptions about the duration of time required to resolve misdiagnosis. The elicitation conducted by ScHARR focussed on two questions that were presented to experts:

- *“For someone who has been incorrectly diagnosed as 'not asthmatic', how long on average do you think it will take for this incorrect diagnosis to be corrected? What is your 95% confidence interval around this average?”*
- *“For someone who has been incorrectly diagnosed as 'asthmatic', how long on average do you think it will take for this incorrect diagnosis to be corrected? What is your 95% confidence interval around this average?”*

Six experts were recruited to provide their responses to these questions but only four experts provided answers. Of the four experts who provided a response, the first expert was the only expert to provide a quantitative estimate. This expert estimated time to correct resolution for a false-negative diagnosis is in the region of 4-12 months while time to correct a false-negative diagnosis may take 12 months or longer. It is important to take into account, the expert noted considerable uncertainty around this estimate. The fourth expert deemed this estimate as “not unreasonable” but this expert also declared these quantities as “*unknowable”*. The remaining three experts provided qualitative responses rather than quantitative estimates declaring the parameters of interest as “*impossible to answer”, “unknowable”*.

From the qualitative answers provided by the experts, it is clear that the posed questions do not capture the complexity of an asthma diagnosis and are not representative of the thought-process that an expert may go through to reach a quantitative estimate. In the case of a false-negative diagnosis, this complexity relates to the “*chronicity”* and “*persistence”* of the asthma. In the case of a false-positive diagnosis, the complexity refers to the misdiagnosis never being resolved due to the patients themselves deciding not to *“just stop going back to the doctor”.*

The following sections of this chapter demonstrate how the reference protocol described in *Chapter 10* was applied. The applicability and practicality of the reference protocol is explored.

## Section 12.4 Application of developed reference protocol

The following sections describe the application of the developed reference protocol in *Chapter 10*. ***Error! Reference source not found.*** provides the protocol that was designed for this SEE process.

### Section 12.4.1 Selecting the quantities (preparation and design stage)

The choice of quantity considered the following three objectives:^52^ fitness for purpose; directly observable and homogeneity in the quantities elicited. Eliciting the same summaries throughout will reduce the burden of training.^201^

The time it takes to resolve an incorrect asthma diagnosis for both false-negative and false-positive cases is elicited. Due to the complexity of asthma diagnosis, the parameters were not directly elicited but were calculated from a number of alternative elicited quantities (decomposing the quantities). The quantities elicited relate to the *probability of an event* (i.e. number of patients returning to the healthcare service) *at different time-points*.

Based on the qualitative feedback from the experts in the DAR, it was clear that there were a number of aspects of the condition that needed to be incorporated into the questions to ensure that these were asked in a manner which would be consistent with how the expert thinks about the condition. In terms of asthma, these characteristics relate to the level of chronicity and persistency of symptoms.

Following personal communication with a General Practitioner (GP) in the University of York, it was decided that specific patient vignettes would be described and presented to the experts at the beginning of each question.

These were presented separately for false-negative and false-positive adults and children. Each described a type of patient based on varying levels of symptom severity (mildly persistent symptoms, moderately persistent symptoms or severely persistent symptom). The experts’ were asked to express the proportion of the type of patient described returns to the healthcare service at certain time-points since their first diagnosis. Eliciting by severity and for adults and children separately is intended to reflect the heterogeneity of the asthmatic population, raised in the DAR elicitation.

Variation in the time to correct diagnosis for both false-negative and false-positive patients is anticipated and thus the questions were asked based on two separate time-points for both types of incorrect asthma diagnosis. This approach also supported the assumption made in the model that all incorrect asthma diagnoses are resolved. For false-negative patients the time-points included were 6 and 12 months while for false-positive, the time-points were 12 and 24 months. ***Error! Reference source not found.*** presents the questions and preambles provided to the experts.

### Section 12.4.2 Methods to encode judgements (preparation and design stage)

To elicit uncertainty two methods of elicitation were explored in *Chapter 8*: the Chips and Bins method and the Bisection method. *Chapter 10* concluded that either of these methods is appropriate for HCDM, and thus here the recruited experts completed one of these methods of elicitation; either Chips and Bins method **or** Bisection method.

The reference protocol in *Chapter 10* states the both VIM and FIM work well but decision makers should aim for consistency across application. The evaluation in this chapter used both methods as it was intended to further explore the usability of the two methods with actual health care professionals.

### Section 12.4.3 Validation (preparation and design stage)

At the end of the SEE, experts were asked if they were confident the answers they gave reflected their views and uncertainties. Response options were ‘yes’, ‘not sure’ and ‘no’. If they responded, ‘no’ or ‘not sure’, they were asked to provide more detail as to why in an open question. Other forms of validation were not used.

### Section 12.4.4 Selecting experts (preparation and design stage)

As asthma is generally diagnosed in primary care, it was assumed Primary Care GPs would have substantive knowledge in the diagnosis of asthma. Consequently, Primary Care GPs were recruited as the experts. Experts were not expected to have any normative skills (see *Chapter 10*). The experts were recruited using recommendation from peers.

As this work is a retrospective evaluation of the reference protocol developed, rather than a SEE per se, a smaller number of experts, than usually recommended, were recruited. Four experts were recruited. The intention was to utilize the protocol rather than generate evidence and if sufficient time were available more experts would have been recruited. However, as mentioned at the beginning, this chapter focused on the design elements of the SEE rather than the practical conduct. Two experts completed the Chips and Bins Method and two experts completed the Bisection Method. These were allocated randomly by the facilitator.

At the beginning of the exercise, experts were asked to provide some background information about themselves. This included, the number of years they have been working in general practice and how they commonly diagnose an adult or child who they suspect may have asthma. The experts were asked to identify whether they use an objective test (spirometry, reversibility testing), a clinical evaluation or both methods.

### Section 12.4.5 Pilot exercise (preparation and design stage)

The wording of the questions was piloted for clarity and adequacy. The pilot exercise was sent to two GPs and feedback sought. Following feedback the questions were modified, specifically the wording of the questions.

### Section 12.4.6 Training and preparation for experts (preparation and design stage)

A narrated power-point training session was embedded within exercise. The training session described the objectives of the elicitation exercise, clarified concepts such as uncertainty, familiarised the experts with the quantities elicited, described and explained the impact of bias and heuristics, and trained experts on the methods of elicitation used.^201^

Experts were also reminded throughout the SEE that they were to elicit uncertainty on their estimate rather than thinking about variability across this heterogeneous group of patients

### Section 12.4.7 Level of elicitation (elicitation stage)

Each expert elicited their judgements individually without interaction with other experts. Eliciting judgements individually reduced the risk of estimates being biased by a subset of experts. In the SEE elicitation literature, there are concerns that experts may not feel confident in eliciting judgements individually, however, the experts in this SEE process elicited their beliefs on a condition that they encounter regularly in general practice. Concerns regarding individual level elicitation and lower confidence amongst experts generally arises when dealing with problems/technologies or conditions that are new or unknown to the experts (see *Chapter 6*).

### Section 12.4.8 Mode of administration (elicitation stage)

The elicitation exercise was administered via a computer-based method using a *de novo* tool in Excel. The evaluation used a mixture of face-to-face and remote forms of administration. Despite using individual level elicitation, a facilitator was present, either in person or on the phone at the time the expert completed the exercises. The purpose of this was to gather as much feedback as possible on the elicitation process (see Section 8.5.5). For example, the time it took to complete the exercise or to record any difficulties the expert had when completing the process.

### Section 12.4.9 Feedback to experts and revision (elicitation stage)

Once experts expressed their beliefs and completed each question, they were presented with graphical feedback of what their estimates looked like (see *Chapter 10*). In the Chips and Bins Methods, experts were able to see how the grid looked once they have placed all of their chips on it. Similarly in the Bisection method, experts were able to see the breakdown of the different values they provided (median, upper and lower quartile etc.). The individual level of elicitation that was chosen meant that group consensus was not required and consequently, group feedback to the experts was not necessary. Both methods had a reset button. Once the expert completed each question, they had an opportunity to click reset and begin that particular question again.

### Section 12.4.10 Opportunity for interaction (elicitation stage)

Given the individual level of elicitation that was chosen, there was no opportunity for interaction between the experts.

### Section 12.4.11 Feedback from experts on process (elicitation stage)

Qualitative feedback on the elicitation process was collected from the experts. This was collected by the facilitator using a feedback questionnaire post exercise. The feedback questions assessed the following concepts;

- observability of the quantity asked
- based on a 5-point scale, assess how easy or difficult the experts found the completion of the exercise
- based on a 5-point scale, evaluate whether the wording of the questions were easy or difficult to understand
- whether the provided training is sufficient
- whether the expert would prefer to have some interaction with a colleague or another expert (if they were to complete the exercise again)
- if they would be willing to complete this exercise again without a facilitator

The feedback also asked experts to suggest improvements they think necessary for any future SEE. In addition, any useful comments or suggestions made by the expert throughout the SEE were also collected. Section 11.4.11discusses the feedback from the experts. While not collected as part of the feedback questionnaire, at the end of each section in the exercise, rationales from the experts about how they made their judgements were collected. This form of validation helps to highlight if experts understood the task and responded as best they could.

### Section 12.4.12 If/how to aggregate (aggregation, analysis and post-elicitation)

As an individual level of elicitation was chosen, mathematical aggregation should be applied to generate the distributions, specifically linear opinion pooling using equal weighting of experts (see *Chapter 10*). In this application, the intention was to explore the use of the reference protocol, rather than generate a single distribution relating to the uncertain quantities of interest, and therefore this aggregation is not undertaken.

### Section 12.4.13 Fit to distribution (aggregation, analysis and post-elicitation)

A Beta distribution would be fitted to experts distributions as these relate to probabilities.

### Section 12.4.14 Data Protection and Anonymity (aggregation, analysis and post-elicitation)

Experts were asked to give their opinions individually (not in groups). The information provided, including personal details, is kept anonymous and confidential, stored securely and only accessed by those carrying out the study.

## Section 12.5 Results

The number of years the experts have been working in general practice ranged from 6 to 35 years (*Table* 17*16*). When asked how they would usually test an adult with suspected asthma, all four experts reported using both an objective test (e.g. FeNO, Spirometry or reversibility testing) and a clinical evaluation. When diagnosing a child with suspected asthma, three of the four experts reported using just a clinical evaluation and one expert reported using both a clinical evaluation and an objective test. Expert 1 reported that for this questions and the remainder of the questions in the evaluation, the age of the child population should be defined as follows: <1 (should not have any diagnosis as their lungs will not be developed), 1 to 4 years, 5 to 14 years, older than 14 years. This expert then went on to explain that the older the child the more likely the chance a GP could use an objective test in addition to a clinical evaluation to diagnose asthma.

Table 17 Application of reference protocol: Summary of experts recruited

| **Expert** | **Years GP** | **Asthma diagnosis (Adult)** | **Asthma diagnosis (Child)** | **Elicitation Method** | **Mode** | **Completion time** |
| --- | --- | --- | --- | --- | --- | --- |
| GP1 | 6 | Test and clinical evaluation | Clinical evaluation | Chips and Bins | Face-to-face | 1 Hour |
| GP2 | 35 | Test and clinical evaluation | Both | Bisection | Face-to-face | 45 mins |
| GP3 | 24 | Test and clinical evaluation | Clinical evaluation | Bisection | Remote | 1.5 Hours |
| GP4 | 30 | Test and clinical evaluation | Clinical evaluation | Chips and Bins | Face-to-face | 1 Hour |

Table 18 Application of reference protocol: Elicitation Results

| **Expert** | **Patient type (by severity)** | **False-positive (adults)** | | **False-positive (children)** | | **False-negative (adults)** | | **False-negative (children)** | |
| --- | --- | --- | --- | --- | --- | --- | --- | --- | --- |
|  |  | 12 months | 24 months | 12 months | 24 months | 6 months | 12 months | 6 months | 12 months |
| GP1 | Severe  Moderate  Mild | 30, 70  30, 70  10, 60 | 60, 90  45, 75  10, 65 | Same judgements as adult patients | | 60, 99  45, 85  0, 55 | 99, 100  99, 100  99, 100 | 70, 100  60, 100  10, 60 | 99, 100  99, 100  99, 100 |
| GP2 | Severe  Moderate  Mild | 5, 25  15, 35  30, 50 | 99, 100  99, 100  35, 55 | 5, 10  5, 15  20, 30 | 99, 100  99, 100  99, 100 | 70, 90  40, 60  35, 45 | 99, 100  50, 70  40, 50 | 85, 95  80, 90  50, 60 | 99, 100  99, 100  99, 100 |
| GP3 | Severe  Moderate  Mild | 90, 100  50, 90  25, 50 | 90, 100  60, 80  25, 50 | Same judgements as adult patients | | 90, 100  60, 90  1, 50 | 90, 100  45, 80  1, 50 | Same judgements as adult patients | |
| GP4 | Severe  Moderate  Mild | 75, 100  60, 90  30, 70 | 75, 100  65, 95  35, 75 | 83, 100  66, 99  33, 77 | 83, 100  72, 100  39, 83 | 75, 100  60, 90  30, 70 | 75, 100  65, 95  35, 75 | 83, 100  66, 99  33, 77 | 83, 100  72, 100  39, 83 |

### Section 12.5.2 Elicitation Results

As discussed in Section 11.4.12, the intention of this evaluation was to explore the use of the reference protocol rather than generate a single distribution. Therefore, *Table 18* simply presents the ranges (upper and lower limits) reported by the experts for false-positive and false-negative adults and children based on different patients types (by severity). Taking the complexity of asthma into account, it is expected that a lower proportion of patients (adults and children) with mildly persistent symptoms will return to the healthcare service compared to patients with severely or moderately persistent symptoms. It is also expected that at the second time-point, a higher proportion of patients will return to the healthcare service compared to the first time-point for each patient type. When comparing the ranges for adults and children, it is expected that overall, a higher proportions of children will return to the healthcare service due to parents concern. For the most part, these expected ranges were reported by the experts, which indicate that the experts understood what was being asked of them and that this concept was something they could think about in their own general practice experience.

When comparing the ranges questions based on false-positive adults and children at the first time-point, the ranges provided by GP2 seem to move in the opposite direction as to what is expected. However, in this experts experience, children are less likely to come back at this time-point as they are easier to diagnose at an earlier stage compared to adults.

## Section 12.6 Feedback from experts on the process (including rationales and validation)

### Section 12.6.1 Observability of the quantity asked

Three of the four experts reported that the selected quantity was observable; that the proportion of patients returning to the healthcare service within a particular time period was something they could express their opinion about. However, GP3 said this was not something that he could think about because, in his opinion, misdiagnosis of asthma does not happen that often.

### Section 12.6.2 Completion of exercise

GP1 completed the Chips and Bins method. This GP explained that the method at first seemed daunting but after completing one of the questions, deemed the method as straightforward. Both GP2 and 3 completed the Bisection method but reported conflicting feedback on the completion of the exercise. GP2 found the Bisection method easy to complete while GP3 reported the method as very difficult to complete.

### Section 12.6.3 Wording of the question

When asked whether the wording of the questions were easy or difficult to understand, GP2 and GP4 reported the wording as *easy to understand* while GP1 and 3 found the wording *very difficult to understand*. GP1 provided further detailed feedback on this and suggested that the preambles should include more detail in terms of defining the severities of asthma, i.e. more description. All GPs identified the training session as sufficient and GP1 suggested a practice exercise would be useful in the training session.

### Section 12.6.4 Value of interaction

When asked if they thought interaction with other experts or colleagues would be useful; GPs 2, 3 and 4 thought this would be beneficial. Their reasoning for this was that to hear other experts rationales, to ensure all experts are making judgement on the same issue and that a small group of experts allowed to interact and achieve a consensus would give a more rounded view. However, GP4 did emphasise that it would be important to avoid the interaction from becoming dominated by one expert in the group. GP1 did not think interaction with other colleagues was important in this case. This expert was of the opinion that GPs should be adequately familiar with asthma to confidently answer the question independently.

### Section 12.6.5 Value of facilitator

Experts were asked that if they were to complete the exercise again, would they be happy to complete it without the use of a facilitator. Three out of the four experts said yes they would be happy for the exercise to be non-facilitated. GP3 stated that if the process was not facilitated, the requirements of the task would be unclear.

### Section 12.6.6 Experts rationales

When reporting on their thought-processes, all experts considered similar patients they encounter in general practice. One of the experts explained that when a patient has an asthma diagnosis noted on their records, the patient is be invited to attend an annual visit for a respiratory check-up. In the GPs experience, 85% of these patients will return for their annual visit and subsequently, this expert reported using this figure as a guideline when making the judgements. Three of the four experts stated they were confident that the answers they gave in the exercise reflected their own views and uncertainty. GP3 was not sure of this and explained that the relevance of the questions to clinical practice was not obvious. In addition, the expert found the exercise repetitive due to the two time-points making the exercise tedious to complete.

### Section 12.6.7 General feedback from experts

Experts provided general comments and improvements for future SEE processes. When completing the Chips and Bins method, GP1 found the method cognitively challenging. The expert provided an upper and limit for each question but did not fill in the grid using the chips due to the grid being different sizes for each question and varying chips available. While the facilitator acknowledged this was correct and that the grid size and the amount of chips available are dependent on the range given by the expert, the expert did not place the chips in the bins to show certainty/uncertainty on the proportions.

As described in Section 11.4.1, two time-points were used in the false-negative and false-positive descriptions. Given the layout of the exercise, experts had to scroll back to the initial time point if they wanted a reminder of the previous judgement they made before providing their judgement at the second time-point. Two of the experts said it would be more accommodating if the first time-point was visible while answering the second as the first judgement would serve as a benchmark. In essence, the experts suggested that for future elicitation processes, if questions are a follow-on from a previous question, it would be useful if the previous judgements were easily accessible.

### Section 12.6.8  Practicality of conducting the SEE process

The design and conduct of the SEE was undertaken over a 7 month period (August 2018 to February 2019 - excluding any form of aggregation or fitting (see Section 11.4.12) and involved three researchers over that time-period. In terms of analyst resources, this included one 0.6 full time equivalent (FTE) for the duration of the process, with the addition of a 0.1 FTE for the final 3 months and an additional 0.5 FTE for the remaining 2 months. This covered development of the questions and subsequent piloting of the wording of the questions; developing the training sessions and developing the excel-based elicitation exercise. Expert recruitment accounted for one month of the study period (January 2019). Administration and completion of the elicitation exercises along with the write-up was conducted during the final month of the process (February 2019).

## Section 12.7 Conclusion

The aim of this chapter was to apply the reference protocol to a case-study and to explore any practical issues. This highlighted a number of key issues in the SEE process relevant in a HCDM context. It is clear from this SEE process and the feedback provided by the experts, that sufficient information needs to be presented to the experts. The level of information presented to the experts and the wording of this information is paramount in ensuring that the quantity of interest is observable to the expert. When deciding on the information to provide to experts in a HCDM context, based on the rationales provided by the experts in this process, it may be useful to consult existing policies.

In a HCDM context, a SEE process will be subject to timeline constraints. Certain available choices in SEE may result in a more lengthy process for example face-to-face modes of administration or interaction between experts. Careful consideration must be given to these choices to achieve accurate judgements from experts but also to make efficient use of available time. In terms of interaction between experts, the feedback from experts in this SEE process indicates that consideration needs to be given to the potential value of interaction between experts. Depending on the context of the SEE, interaction between experts may be more essential, therefore justifying a lengthier process. For example, if the SEE process is focusing on a new drug or a rare disease, interaction between experts may be more significant than a process focussing on an established drug or a commonly encountered condition or illness. In the latter, experts will be more familiar with the context and should therefore have the ability to independently provide a response in a confident manner.

# Chapter 13 Discussion & conclusions

This chapter discusses the evidence that has been generated in Chapters 2-11. It goes on to consider how the reference protocol for SEE may be used by policy makers to define their own reference protocol that reflects their particular constraints. To do this this it considers the feedback from a workshop convened as part of the project. Areas for further research emerging from the work conducted, and discussed at the workshop, are also discussed. Finally the limitations of the work are noted.

## Section 13.1 Conclusions on evidence generated

SEE can offer opportunities in HCDM, particularly reimbursement decisions supported by MBEE. SEE allows the uncertainty in the evidence used to populate these models to be characterised, or where evidence is completely lacking, provides additional information needed to reach a decision.

The work described in this report has attempted to generate evidence which is useful for analysts and decision makers in HCDM. SEE conducted in this context to date, has not used a set of consistent methods, and, above all, has not considered the implications of the choices made when designing and conducting a SEE. To improve the accountability of HCDM the procedure used to derive expert judgements should be transparent.

A reference protocol for SEE in HCDM is proposed in *Chapter 10*. The reference protocol is intended to serve as a guide to good practice and reporting, rather than being prescriptive regarding methods, and thus it is intended more as a guidance rather than a protocol. This was necessary due to the lack of empirical evidence underpinning methods choices specific to HCDM. Instead choices were considered according to the principles for SEE in HCDM, set out in *Chapter 9*. These nine principles were developed based on findings from Chapters 3-7, which consider: the constraints in HCDM, how SEE has been applied in the literature and challenges faced, evidence relating to particular methodological aspects of design and conduct and considerations in choosing alternative quantities that can be elicited. The principles also reflect ‘good practice’ in SEE more generally, as reported by Cooke (1991).^14^

As stated in *Chapter 10*, the lack of evidence relating to HCDM, and a paucity of applied studies, meant that the reference protocol focuseed on the more narrowly defined setting of national level HTA. Whilst this encompasses a range of activities that could include SEE, it does not reflect more complex settings which could pose additional challenges. For example, HCDM at a local level, for early technologies that have yet to progress through the regulatory process, or for specific types of HTA, including rare diseases or genomics. These settings may require a different approach to elements such as recruiting experts, level of elicitation and delivery. It is recommended that such decision makers consider the elements of the reference protocol and how these translate to their setting. In doing so they can determine a reference protocol of their own.

The experiments, described in *Chapter 8*, suggested that there is little difference between VIM and FIM methods to encode judgements. The reference protocol therefore stated that a decision maker can consider either of these choices suitable, however consistency across applications is preferred, i.e. they should choose either VIM or FIM and use this throughout their decision making processes. The experiments also sought to explore extrapolation beyond data observed and updating of priors after presentation of group summaries – issues which feed into multiple choices for SEE. It was difficult to form definitive conclusions given that the experiments were underpowered for these elements. To make definitive statements regarding these aspects of SEE, further experimental data would need to be collected. However, the experiments provided some evidence that experts changed their estimates in a rational way when provided with distributions from others, suggesting that group discussion or feedback may be useful. Extrapolation outside of the observed sample does not seem to affect accuracy, suggesting that it is reasonable to ask experts about patients and practices in which they do not have direct clinical experience, or for whom there is no relevant literature.

*Chapter 11* applies the reference protocol for SEE in HCDM to an existing NICE appraisal. This relates to a diagnostic model for asthma developed as part of the NICE diagnostics programme. The recommendations from the reference protocol were used to determine the following aspects of the SEE: selection of quantities, method to encode judgements, validation, selection of experts, piloting and training, level of elicitation, model of administration, feedback, interaction and post elicitation aggregation. Quantities relating to incorrect diagnosis of asthma were collected from a sample of experts. Time taken to develop and conduct the SEE was recorded and feedback on the elicitation was also sought from experts.

There were only small numbers of experts on which to base conclusions, however from those that completed the SEE, the VIM and FIM seemed to be equally challenging for experts to complete. All four experts found the training to be useful. A facilitator was used in the SEE, however three participants stated that they would be happy to complete the exercise without the facilitator and three stated that they would prefer interaction with another expert, so long as other experts were familiar with asthma and its diagnosis. This finding may be worth considering in settings where it is not possible to gain access to a facilitator. There may be value in allowing experts to interact aside from its use to generate consensus, such as developing a common problem structure or sharing knowledge and information between experts (see Section 5.4). The need for interaction between experts in particular settings, for example in rare diseases, was discussed in *Chapter 10*.

## Section 13.2 Key considerations for using the reference protocol in HCDM

To consider how the reference protocol may be used by HCDMs, a workshop was convened. HCDM stakeholders, including practitioners, policy makers and methodologists, attended. Feedback is described in further detail in ***Error! Reference source not found.***. Briefly, the workshop considered the acceptability of the reference protocol for SEE in HCDM, how the proposed reference protocol for elicitation may be implemented and where it would be most useful. It was also used to identify priorities for further research and development of reference protocol and its use for HCDM.

There was unanimous support for a reference protocol or guidance on SEE in HCDM. Workshop attendants discussed what form this guidance should take, and which would be the most useful, specifically should it be prescriptive or guiding principles. Those involved directly with conducting SEE, tended to suggest that less prescriptive guide would be most useful, as some of the methodological decisions may be driven by the context (see *Chapter 3*), for example a specific appraisal may require SEE to generate results within an extremely short time frame, reducing the possibilities for face to face SEE. Whatever form it takes, workshop participants thought that some form of guidance would help decision makers, considering evidence generated from a SEE, or when planning to conduct their own SEE. Lack of guidance is also seen as a barrier to publishing SEE in HCDM, and therefore a reference protocol would support the dissemination of applied research in this area. It may also encourage the development of materials to assist SEE, for example generic training materials, which are currently lacking.

Workshop participants agreed that a reference protocol may be most useful when there is a lack of substantial existing evidence, such as urgent delivery systems during epidemics, or as a complement to existing data on a longer-term basis, e.g. short trial follow up. There is likely to be a need in areas which are not represented in trials, such as histopathology. The reference protocol developed here is considered appropriate for national level HTA, which is also the audience most likely to be receptive and with sufficient resource to conduct or commission SEE. Within national HTA there may be potential to use a reference protocol for SEE within clinical and public health guidelines.

The time and expertise required to conduct a SEE may be an issue for many of the formal decision making processes that exist, for example the NICE appraisals process. The evaluation undertaken in *Chapter 11* took over 5 per months FTE from start to finish. It is not clear if previous applied examples of SEE may have been forced to make particular choices on the basis of limited time and resource (see *Chapter 4*). This may compromise the quality of the SEE and therefore it may be more appropriate to extend timelines so as to incorporate well conducted SEE. Justification for this is that lots of time is spent on generating evidence and this is just one form of evidence. This may still be a challenge for some decision makers where there are multiple uncertain quantities that could be elicited, thus potentially imposing high costs. A potential solution is to choose parameters for the SEE based on the expected value of partial perfect information corresponding to each parameter, or on a less-formal sensitivity analysis determining the impact of extreme parameter values, in a cost-effectiveness model.

A number of specific issues regarding use of the reference protocol were raised during the workshop. Firstly, it was unclear who would be held accountable for SEE. Ultimately the decision makers were accountable for the decision which utilised the SEE, however experts may also be accountable for the beliefs that they express. In order to make this more explicit, participants agreed that decision makers need to have access to individual elicitation and not just a group/consensus judgement. Recognition of accountability may lead experts to alter their beliefs. Second is the issue of which experts are included in the SEE. In some circumstances recruitment of experts may have to rely on methods such as peer nomination, particularly where there are constraints on time. This may give an unrepresentative sample of views and may be more likely to result in motivational biases, perhaps due to an association with the quantities of interest. The most knowledgeable experts may not always be available for SEE, so that the aggregated distributions may not be representative of the current level of knowledge on the quantity. Thirdly the issue of choosing observable quantities may not be straightforward. Strictly speaking an observable quantity is something that can be measured, which may be the case for the majority of quantities that need to be elicited. This excludes indirectly observed quantities such as odds ratios. Experts may also have different experiences which alters their perception of what is observable and non-observable. In the evaluation (see *Chapter 11*), one of the experts stated that the quantity was not, in their opinion, observable, as misdiagnosis of asthma happens rarely, whilst the other three experts stated that the quantity was observable to them.

## Section 13.3 Key areas for further research

In considering the appropriateness of choices for SEE in HCDM and exploring how these choices may be affected by the context in which the SEE is applied, there are areas in which further research is required before definitive statements can be made regarding their appropriateness for a reference protocol. These areas were discussed at the workshop and refined following discussion. In ensuring that SEE is used consistently in HCDM and reflects the constraints of that particular setting, not all of these may represent priorities for further research. Workshop participants were not asked to prioritise topics *per se* or consider which issues are most crucial to the accuracy of SEE, and therefore the list does not reflect which topics may be most urgently required. Workshop participants were instead asked to consider what additional evidence decision makers in HCDM may require when determining a reference protocol for SEE for use within their setting. Areas of uncertainty, in the current reference protocol were: selecting experts, minimising bias, adaptation to specific setting in which SEE may be applied, for example choosing individual or group elicitation, appropriate working of questions, methods for multivariate elicitation and what information should be presented to the experts to help them formulate their beliefs.

Examples are summarised in *Table 19*. Some of these could easily be adapted into researchable questions, whereas others are much more vague and general. Some of these topics would benefit from empirical research and others may be resolved though application of the proposed reference protocol to HCDM, including in settings with a range of constraints.

Table 19 Areas for further research on SEE in HCDM

| **Decision choice and general research area** | **An example of a specific question** |
| --- | --- |
| **Selection of experts** | |
| How to determine a sample size for SEE | In individual elicitation, what is the saturation point of increasing sample size? |
| Exploration of strategies for recruiting experts in HCDM | Which methods for expert recruitment are most practical and what are the challenges? |
| Methods to assess experts skills that are appropriate for the SEE task | Where adaptive skills are required, how can these be measured and, where these skills are compromised, can training increase these skills? |
| What minimum level of normative expertise is required | What additional level of normative expertise is required when eliciting more complex quantities or where dependence exists? |
| **Biases** | |
| Training strategies | What training strategies can be used to minimise bias? |
| Recruitment | What recruitment strategies can be used to minimise expert bias, beyond minimising financial/competing interests? |
| Validation | Can the measurement of expected bias provide a mechanism to validate elicitation? |
| **Validation of experts** |  |
| Performance assessment | How many seeds are required to estimate experts expected accuracy in HCDM, and how can these be efficiently generated? |
| Calibration | To what extent might performance-based weighting improve the validity of resulting distributions? |
| Accuracy | What is the relationship between characteristics of experts and accuracy of elicited quantities? |
| **Quantities** | |
| Dependence methods | Which methods for eliciting dependent quantities work best for non-normative experts? |
| Consistency | Does elicitation of consistent quantities throughout the task improve procedural accuracy? |
| Survival parameters | How to elicit parameters of survival models, in particular uncertainty relating to these? |
| **Group elicitations and interaction** | |
| Consensus approach | Which consensus approach works best in HCDM in practice and for which types of quantities and decision makers? |
| Sample size | How many experts should be part of a consensus elicitation process, and does this differ by context? |
| **Aggregation** | |
| Distribution fitting | What methods for fitting distributions to elicited beliefs are most appropriate for particular quantities, for example more complex quantities |
| Combining priors | Should individual priors be combined when there is significant expert variation? If so, how? |

## Section 13.4 Limitations of the work conducted

Whilst the reference protocol developed here represents a significant move forward in terms of SEE applied to HCDM, there are a number of limitations of the work that are worth noting.

Firstly, in developing a reference protocol for SEE in HCDM, it was necessary to draw on multiple types of evidence, structured (systematic) review, targeted literature search, and experimental analyses. In identifying relevant evidence from the existing literature it was not possible to use systematic search methods for all reviews, and the targeted searches used a semi-structured approach (*Chapter 5*). This may have resulted in relevant studies that are less well-known in the elicitation literature also being missed in our reviews. In addition, it was not possible to conduct targeted searches for all elements of SEE.

Second, there were a number of compromises that were needed in order to generate empirical evidence relating to the choice between the FIM and VIM (*Chapter 8*). Foremost, it was necessary to use students to represent health care professionals. The design of the experiments was such that, prior clinical knowledge was not required to complete the tasks, and therefore the experts were instead meant to represent the level of normative skills that would usually be expected in HCDM. Participants were standardised according to the level of knowledge they observed form the simulated learning process. However, in practice experts in HCDM are likely to draw on multiple sources of knowledge when formulating their beliefs, i.e. health carers may also draw on published evidence, peer contact or other related evidence or experience. It was not possible to reflect these multiple forms of knowledge in exploring the performance of the methods to encode judgements. The experimental set up, more generally, may impact on the generalisability of the results.

Thirdly, it was not possible to explore all of the uncertain choices empirically through the experimental approach described in *Chapter 8*In addition to the comparison of the FIM and VIM approaches, *Chapter 8* also looked at how experts update their beliefs when presented with group summaries and extrapolation beyond data observed. It was not possible to power the experiments to detect differences for these two elements, and therefore it is difficult to reach conclusions regarding these comparisons.

The major limitation of the work conducted here, lies not in the methods employed, but the evidence available from the wider literature, on which to base the set of choices and determine how appropriate these are. Concluding on the suitability of the choices available from the existing guidelines is challenging due to the lack of empirical evidence to support specific choices. Instead it was necessary to develop principles for SEE in HCDM, using the sources of evidence as described above and published guidelines for good SEE. Using the principles, meant that it was not always possible to give definitive conclusions on choices.

This flexibility, however may be a useful characteristic of the reference protocol developed here. Trying to define a reference protocol that is useful, in that is refines the set of choices, but is sufficiently flexible that it can be applied across HCDM and considers constraints in different settings, may provide the type of guidance that is most useful at this stage. Further applied studies of SEE in HCDM, which consider the choices specified in this reference protocol, and thoroughly document these, will help to generate valuable evidence on the usefulness of the reference protocol and may also provide opportunities for empirical comparisons of some of the remaining uncertain choices, for example using the approach in *Chapter 8*.

# Chapter 14 Acknowledgements

This work was funded by a grant from the Medical Research Council (MRC): MR/N028511/1 “HEE: Developing a reference protocol for expert elicitation in health care decision making”.

We would like to acknowledge the contributions from the advisory group for the project, specifically, John Paul Gosling, Jeremy Oakley, Anthony Hatswell, Nicky Best, Matt Stevenson, Peter Ayton, Tim Bedford, Maarten Ijzerman, Rebecca Albrow, Frances Nixon, and Roger Cooke.

A workshop was convened to consider the results from the project and how these may be used in practice by decision makers. The workshop was attended by NICE chairs, NICE staff, academics, NHS England, WHO, industry representatives, other methodologists and decision makers. We would like to acknowledge and thank all that attended and participated.

We would like to acknowledge Ian Watt, Alastair Dickson, Sean O’Connell and Mark Williams for their help and participation in the expert elicitation exercise as part of the application of the developed reference protocol in *Chapter 11*.

Finally in designing the experiments conducted in *Chapter 8*, piloting was carried out on colleagues at the Centre for Health Economics. We would like to acknowledge and thank all colleagues that took part and provided valuable feedback. In addition colleagues at the Experimental Economics Lab at the University of York.

**Data sharing:**

All data requests should be submitted to the corresponding author for consideration.  Access to anonymised data may be granted following review.

# Chapter 15 Contributions of authors

Dr Laura Bojke (Reader Health economics) was responsible for day-to-day management of the project, developing the reference protocol, supervising the evaluation, running the experiments and drafting the report. Dr Marta Soares (Senior Research Fellow) was jointly responsible for the day-to-day running of the project, designed the experiments in Chapter 8 and wrote Chapter 7 with CJ and LS. Dr Chris Jackson provided statistical support for the experiments and generated measurements to assess performance in experiment 1. He wrote Chapter 7 with MS and LS, Chapter 5 with AF and AC, and worked with MS and DJ to draft Chapter 8. Professor Linda Sharples (Medical Statistics) provided senior statistical support to the project, specifically in designing the experiments in Chapter 8. She wrote Chapter 7, with CJ and MS, contributed to Chapters 4, 6 and 8. Professor Karl Claxton (Health Economics) contributed towards the design of the experiments in Chapter 8, and helped to draft materials in Chapters 9 and 10. Dr Dina Jankovic (Research Fellow) developed the statistical code for the experiments in Chapter 8, conducted the experiments with MS and LB and helped the draft Chapter 8. Dr Aimee Fox (Research Fellow) was responsible for Chapter 5, with CJ and AC, and worked with LB to conduct the evaluation in Chapter 11. She took responsibility for pulling together materials from all work packages to generate this report. Dr Andrea Taylor (Academic Fellow) was primarily responsible for *Chapter 6* and drafted this with LB and LS. She provided psychology input into the experiments conducted in Chapter 8 and comments on draft Chapters. Dr Abigail Colson (Lecturer, Management Science) took primary responsibility for the review and critique in Chapter 2. She contributed towards the evaluation in Chapter 11 and worked with AF and CJ on Chapter 5. Professor Alec Morton provided senior support for the work carried out in Chapter 2 and provided input on all Chapters and the final report.

# Chapter 16 Patient and public involvement

No PPI activities were planned as part of this project. An engagement workshop took place, involving NHS decision makers, analysts and methodologists. The details of this engagement activities are presented in Chapter 12 and Supplementary material 5.

# Chapter 17 References

1.	Soares MO, Sharples L, Morton A, Claxton K, Bojke L. Experiences of Structured Elicitation for Model-Based Cost-Effectiveness Analyses. *Value Health* 2018;**21**:715-23. <http://dx.doi.org/10.1016/j.jval.2018.01.019>

2.	Bryan S, Williams I, McIver S. Seeing the NICE side of cost-effectiveness analysis: a qualitative investigation of the use of CEA in NICE technology appraisals. *Health Economics* 2007;**16**:179-93.

3.	Bothwell LE, Greene JA, Podolsky SH, Jones DS. Assessing the Gold Standard--Lessons from the History of RCTs. *N Engl J Med* 2016;**374**:2175-81. <http://dx.doi.org/10.1056/NEJMms1604593>

4.	Chavez-MacGregor M, Giordano SH. Randomized Clinical Trials and Observational Studies: Is There a Battle? *J Clin Oncol* 2016;**34**:772-3. <http://dx.doi.org/10.1200/jco.2015.64.7487>

5.	Rothwell PM. External validity of randomised controlled trials: "to whom do the results of this trial apply?". *Lancet* 2005;**365**:82-93. <http://dx.doi.org/10.1016/s0140-6736(04)17670-8>

6.	Frieden TR. Evidence for Health Decision Making - Beyond Randomized, Controlled Trials. *N Engl J Med* 2017;**377**:465-75. <http://dx.doi.org/10.1056/NEJMra1614394>

7.	Hora SC. Aleatory and epistemic uncertainty in probability elicitation with an example from hazardous waste management. *Reliability Engineering & System Safety* 1996;**54**:217-23. <http://dx.doi.org/https://doi.org/10.1016/S0951-8320(96)00077-4>

8.	O’Hagan A, Buck C, Daneshkhah A, Eiser J, Garthwaite P, Jenkinson D*, et al.* *Uncertain judgements: eliciting experts’ probabilities*; 2006.

9.	Griffin SC, Claxton KP, Palmer SJ, Sculpher MJ. Dangerous omissions: the consequences of ignoring decision uncertainty. *Health Econ* 2011;**20**:212-24. <http://dx.doi.org/10.1002/hec.1586>

10.	Babuscia A, Cheung K-M. An approach to perform expert elicitation for engineering design risk analysis: methodology and experimental results. *J R Statist Soc* 2014;**177**:475–97.

11.	Ayyub BM. *Elicitation of expert opinions for uncertainty and risks*. Boca Raton, Fla.: CRC Press; 2001.

12.	Peel A, Jenks M, Choudhury M, Lovett R, Rejon-Parrilla JC, Sims A*, et al.* Use of Expert Judgement Across NICE Guidance-Making Programmes: A Review of Current Processes and Suitability of Existing Tools to Support the Use of Expert Elicitation. 2018;**16**:819-36. <http://dx.doi.org/10.1007/s40258-018-0415-5>

13.	Soares MO, Bojke L. Expert elicitation to inform health technology assessment. In: International Series in Operations Research and Management Science; 2018: 479-94. <http://dx.doi.org/10.1007/978-3-319-65052-4_18>

14.	Cooke RM. Experts in Uncertainty: Opinion and Subjective Probability in Science. *Oxford University Press* 1991.

15.	O'Hagan T, Oakley J. The Sheffield Elicitation Framework (SHELF). 2008.

16.	Colson AR, Cooke RM. Expert Elicitation: Using the Classical Model to Validate Experts’ Judgments. *Review of Environmental Economics and Policy* 2018;**12**:113-32. <http://dx.doi.org/10.1093/reep/rex022>

17.	European Food Safety Authority. Guidance on Expert Knowledge Elicitation in Food and Feed Safety Risk Assessment. *EFSA Journal* 2014;**12**. <http://dx.doi.org/10.2903/j.efsa.2014.3734>

18.	EPA. *Expert Elicitation Task Force White Paper*. Draft. Washington D.C.: U.S. Environmental Protection Agency; 2009.

19.	Kaplan S. 'Expert information' versus 'expert opinion.' Another approach to the problem of eliciting/combining/using expert knowledge in PRA. *Reliability Engineering & System Safety* 1992;**35**:61-72. <http://dx.doi.org/10.1016/0951-8320(92)90023-e>

20.	Cooke RM, Goossens LHJ. Procedures guide for structured expert judgement in accident consequence modelling. *Radiation Protection Dosimetry* 2000;**90**:303-9.

21.	Choy S, O’Leary R, Mengersen K. Elicitation by design in ecology: using expert opinion to inform priors for Bayesian statistical models. *Ecology* 2009;**90**.

22.	Kotra J, Lee M, Eisenberg N, DeWispelare A. Branch Technical Position on the Use of Expert Elicitation in the High-Level Radioactive Waste Program. *Division of Waste Management, Office of Nuclear Material Safety and Safeguards, US Nuclear Regulatory Commission* 1996.

23.	Tredger ERW, Lo JTH, Haria S, Lau HHK, Bonello N, Hlavka B*, et al.* Bias, guess and expert judgement in actuarial work. *British Actuarial Journal* 2016;**21**:545-78. <http://dx.doi.org/10.1017/s1357321716000155>

24.	Budnitz RJ, Apostolakis G, Boore DM, Cluff LS, Coppersmith KJ, Cornell CA*, et al.* *Recommendations for Probabilistic Seismic Hazard Analysis: Guidance on Uncertainty and Use of Experts:* no. NUREG/CR-6372 UCRL-ID- 122160. Livermore, CA: U.S. Nuclear Regulatory Commission; 1997.

25.	Hemming V, Burgman MA, Hanea AM, McBride MF, Wintle BC. A practical guide to structured expert elicitation using the IDEA protocol. *Methods in Ecology and Evolution* 2018;**9**:169-80. <http://dx.doi.org/10.1111/2041-210x.12857>

26.	Garthwaite PH, Kadane JB, O'Hagan A. Statistical Methods for Eliciting Probability Distributions. *Journal of the American Statistical Association* 2005;**100**:680-701. <http://dx.doi.org/http://dx.doi.org/10.1198/016214505000000105>

27.	Knol AB, Slottje P, van der Sluijs JP, Lebret E. The use of expert elicitation in environmental health impact assessment: a seven step procedure. *Environmental Health* 2010;**9**. <http://dx.doi.org/Artn> 19

10.1186/1476-069x-9-19

28.	Walls L, Quigley J. Building prior distributions to support Bayesian reliability growth modelling using expert judgement. *Reliability Engineering & System Safety* 2001;**74**:117-28. <http://dx.doi.org/10.1016/s0951-8320(01)00069-2>

29.	Gosling JP. SHELF: The sheffield elicitation framework. In: International Series in Operations Research and Management Science; 2018: 61-93. <http://dx.doi.org/10.1007/978-3-319-65052-4_4>

30.	Keeney R, von Winterfeldt D. Eliciting Probabilities from Experts in Complex Technical Problems. *IEEE Transactions On Engineering Management* 1991;**38**.

31.	Meyer MA, Booker JM. *Eliciting and Analyzing Expert Judgment: A Practical Guide*. Philadelphia, PA: Society for Industrial and Applied Mathematics; 2001.

32.	Ashcroft M, Austin R, Barnes K, MacDonald D, Makin S, Morgan S*, et al.* Expert Judgement. *British Actuarial Journal* 2016;**21**:314-63.

33.	Budescu DV, Chen E. Identifying Expertise to Extract the Wisdom of Crowds. 2015;**61**:267-80. <http://dx.doi.org/10.1287/mnsc.2014.1909>

34.	Gilovich T, Griffin DW, Kahneman D, Cambridge University P. *Heuristics and biases : the psychology of intuitive judgment*. Cambridge: Cambridge University Press; 2013.

35.	Kahneman D, Slovic P, Tversky A. *Judgment under uncertainty : heuristics and biases*. Cambridge; New York: Cambridge University Press; 1982.

36.	Morgan MG. Use (and abuse) of expert elicitation in support of decision making for public policy. *PNAS* 2014;**111**:7176–84.

37.	NHS. *Guide to the Healthcare System in England Including the Statement of NHS Accountability*; 2013.

38.	Kershaw A. *NHS Vale of York CCG Referral Support Service Useful Information*. York; 2017.

39.	Kay A. The abolition of the GP fundholding scheme: a lesson in evidence-based policy making. *The British journal of general practice : the journal of the Royal College of General Practitioners* 2002;**52**:141-4.

40.	Lafond S, Charlesworth A, Roberts A. *A year of plenty? An analysis of NHS finances and consultant productivity*. London; 2017.

41.	King's Fund. *Has the government delivered a new era for public health?* The King's Fund; 2015. URL: <https://www.kingsfund.org.uk/projects/verdict/has-government-delivered-new-era-public-health> (Accessed 28th March, 2019).

42.	NHS. *Interim Commissioning Policy: Individual funding requests*. England: NHS Commissioning Board; 2013.

43.	Ham C, Glenn R. Reasonable Rationing: International Experience of Priority Setting in Health Care (State of Health) In: Open University press; 2003.

44.	Grigore B, Peters J, Hyde C, Stein K. Methods to Elicit Probability Distributions from Experts: A Systematic Review of Reported Practice in Health Technology Assessment. *PharmacoEconomics* 2013;**31**:991–1003.

45.	NICE. *Guide to the process of technology appraisal*; 2014.

46.	Bennett P, Hare A, Townshend J. Assessing the risk of vCJD transmission via surgery: models for uncertainty and complexity. *Journal of the Operational Research Society* 2005;**56**:202-13. <http://dx.doi.org/10.1057/palgrave.jors.2601899>

47.	Colson AR, Megiddo I, Alvarez-Uria G, Gandra S, Bedford T, Morton A*, et al.* Quantifying uncertainty about future antimicrobial resistance: Comparing structured expert judgment and statistical forecasting methods. *PLoS One* 2019;**14**:e0219190. <http://dx.doi.org/10.1371/journal.pone.0219190>

48.	Dallow N, Best N, H Montague T. *Better Decision Making in Drug Development Through Adoption of Formal Prior Elicitation*; 2017. <http://dx.doi.org/10.1002/pst.1854>

49.	Walley RJ, Smith CL, Gale JD, Woodward P. Advantages of a wholly Bayesian approach to assessing efficacy in early drug development: a case study. *Pharm Stat* 2015;**14**:205-15. <http://dx.doi.org/10.1002/pst.1675>

50.	Drummond M. *Methods for the economic evaluation of health care programmes*. Fourth edition. edn. Oxford, United Kingdom ; New York, NY, USA: Oxford University Press; 2015.

51.	Leal J, Wordsworth S, Legood R, Blair E. Eliciting expert opinion for economic models: an applied example. *Value Health* 2007;**10**:195-203. <http://dx.doi.org/10.1111/j.1524-4733.2007.00169.x>

52.	Soares MO, Bojke L, Dumville J, Iglesias C, Cullum N, Claxton K. Methods to elicit experts' beliefs over uncertain quantities: application to a cost effectiveness transition model of negative pressure wound therapy for severe pressure ulceration. *Stat Med* 2011;**30**:2363-80. <http://dx.doi.org/10.1002/sim.4288>

53.	Haakma W, Steuten LMG, Bojke L, IJzerman MJ. Belief Elicitation to Populate Health Economic Models of Medical Diagnostic Devices in Development. *Appl Health Econ Health Policy* 2014;**12**:327-34. <http://dx.doi.org/10.1007/s40258-014-0092-y>

54.	Bojke L, Claxton K, Bravo-Vergel Y, Sculpher M, Palmer S, Abrams K. Eliciting distributions to populate decision analytic models. *Value Health* 2010;**13**:557-64. <http://dx.doi.org/10.1111/j.1524-4733.2010.00709.x>

55.	McKenna C, McDaid C, Suekarran S, Hawkins N, Claxton K, Light K*, et al.* Enhanced external counterpulsation for the treatment of stable angina and heart failure: a systematic review and economic analysis. *Health Technol Assess* 2009;**13**:iii-iv, ix-xi, 1-90. <http://dx.doi.org/10.3310/hta13240>

56.	Sperber D, Mortimer D, Lorgelly P, Berlowitz D. An Expert on Every Street Corner? Methods for Eliciting Distributions in Geographically Dispersed Opinion Pools. *Value in Health* 2013;**16**:434-7.

57.	Fischer K, Lewandowski D, Janssen MP. Estimating unknown parameters in haemophilia using expert judgement elicitation. *Haemophilia* 2013;**19**:e282-8. <http://dx.doi.org/10.1111/hae.12166>

58.	Garthwaite PH, Chilcott JB, Jenkinson DJ, Tappenden P. Use of expert knowledge in evaluating costs and benefits of alternative service provisions: a case study. *Int J Technol Assess Health Care* 2008;**24**:350-7. <http://dx.doi.org/10.1017/S026646230808046X>

59.	Grigore B, Peters J, Hyde C, Stein K. A comparison of two methods for expert elicitation in health technology assessments. *BMC Medical Research Methodology* 2016;**16**.

60.	Meads C, Auguste P, Davenport C, Małysiak S, Sundar S, Kowalska M*, et al.* Positron emission tomography/computerised tomography imaging in detecting and managing recurrent cervical cancer: Systematic review of evidence, elicitation of subjective probabilities and economic modeling. *Health Technology Assessment* 2013;**17**:7-144. <http://dx.doi.org/10.3310/hta17120>

61.	Wilson EC, Stanley G, Mirza Z. The Long-Term Cost to the UK NHS and Social Services of Different Durations of IV Thiamine (Vitamin B1) for Chronic Alcohol Misusers with Symptoms of Wernicke's Encephalopathy Presenting at the Emergency Department. *Appl Health Econ Health Policy* 2016;**14**:205-15. <http://dx.doi.org/10.1007/s40258-015-0214-1>

62.	Brodtkorb T-H. *Cost-effectiveness analysis of health technologies when evidence is scarce* Linköping University, Sweden; 2010.

63.	Cao Q, Postmus D, Hillege HL, Buskens E. Probability elicitation to inform early health economic evaluations of new medical technologies: a case study in heart failure disease management. *Value Health* 2013;**16**:529-35. <http://dx.doi.org/10.1016/j.jval.2013.02.008>

64.	Speight PM, Palmer S, Moles DR, Downer MC, Smith DH, Henriksson M*, et al.* The cost-effectiveness of screening for oral cancer in primary care. *Health Technol Assess* 2006;**10**:1-144, iii-iv.

65.	Poncet A, Gencer B, Blondon M, Gex-Fabry M, Combescure C, Shah D*, et al.* Electrocardiographic Screening for Prolonged QT Interval to Reduce Sudden Cardiac Death in Psychiatric Patients: A Cost-Effectiveness Analysis. *PLoS One* 2015;**10**:e0127213. <http://dx.doi.org/10.1371/journal.pone.0127213>

66.	Stevenson MD, Oakley JE, Lloyd Jones M, Brennan A, Compston JE, McCloskey EV*, et al.* The cost-effectiveness of an RCT to establish whether 5 or 10 years of bisphosphonate treatment is the better duration for women with a prior fracture. *Med Decis Making* 2009;**29**:678-89. <http://dx.doi.org/10.1177/0272989X09336077>

67.	Meeyai A, Praditsitthikorn N, Kotirum S, Kulpeng W, Putthasri W, Cooper BS*, et al.* Seasonal influenza vaccination for children in Thailand: a cost-effectiveness analysis. *PLoS Med* 2015;**12**:e1001829; discussion e. <http://dx.doi.org/10.1371/journal.pmed.1001829>

68.	Colbourn T, Asseburg C, Bojke L, Philips Z, Claxton K, Ades AE*, et al.* Prenatal screening and treatment strategies to prevent group B streptococcal and other bacterial infections in early infancy: cost-effectiveness and expected value of information analyses. *Health Technol Assess* 2007;**11**:1-226, iii.

69.	Girling AJ, Freeman G, Gordon JP, Poole-Wilson P, Scott DA, Ford RJ. Modeling payback from research into the efficacy of left-ventricular assist devices as destination therapy. *International Journal of Technology Assessment in Health Care* 2007;**23**:269-77. <http://dx.doi.org/10.1017/S0266462307070365>

70.	Stevenson MD, Oakley JE, Chick SE, Chalkidou K. The cost-effectiveness of surgical instrument management policies to reduce the risk of vCJD transmission to humans. *Journal of the Operational Research Society* 2009;**60**:506-18. <http://dx.doi.org/10.1057/palgrave.jors.2602580>

71.	De Persis C, Wilson S. Using the analytic hierarchy process in the assessment of the probability for an explosion to occur during the atmospheric re-entry. Proceedings of the International Astronautical Congress, IAC, abstract no. 1021.

72.	Iglesias CP, Thompson A, Rogowski WH, Payne K. Reporting Guidelines for the Use of Expert Judgement in Model-Based Economic Evaluations. *Pharmacoeconomics* 2016;**34**:1161-72. <http://dx.doi.org/10.1007/s40273-016-0425-9>

73.	Colson AR, Cooke RM. Cross validation for the classical model of structured expert judgment. *Reliability Engineering & System Safety* 2017;**163**:109-20. <http://dx.doi.org/10.1016/j.ress.2017.02.003>

74.	Eggstaff JW, Mazzuchi TA, Sarkani S. The effect of the number of seed variables on the performance of Cooke's classical model. *Reliability Engineering & System Safety* 2014;**121**:72-82. <http://dx.doi.org/10.1016/j.ress.2013.07.015>

75.	Clemen RT. Comment on Cooke's classical method. *Reliability Engineering & System Safety* 2008;**93**:760-5. <http://dx.doi.org/10.1016/j.ress.2008.02.003>

76.	Montibeller G, von Winterfeldt D. Cognitive and Motivational Biases in Decision and Risk Analysis. *Risk Anal* 2015;**35**:1230-51. <http://dx.doi.org/10.1111/risa.12360>

77.	Philips Z, Ginnelly L, Sculpher M, Claxton K, Golder S, Riemsma R*, et al.* Review of guidelines for good practice in decision-analytic modelling in health technology assessment. *Health Technol Assess* 2004;**8**:iii-iv, ix-xi, 1-158.

78.	Anthony O'Hagan, Caitlin E. Buck, Alireza Daneshkhah, J. Richard Eiser, Paul H. Garthwaite, David J. Jenkinson*, et al.* Uncertain Judgements: Eliciting Experts' Probabilities. *Wiley* 2006:338.

79.	Bolger F. The selection of experts for (probabilistic) expert knowledge elicitation. In: International Series in Operations Research and Management Science; 2018: 393-443. <http://dx.doi.org/10.1007/978-3-319-65052-4_16>

80.	Burgman MA, McBride M, Ashton R, Speirs-Bridge A, Flander L, Wintle B*, et al.* Expert Status and Performance. *PLOS ONE* 2011;**6**:e22998. <http://dx.doi.org/10.1371/journal.pone.0022998>

81.	Clemen RT, Winkler RL. Combining probability distributions from experts in risk analysis. *Risk Analysis* 1999;**19**:187-203. <http://dx.doi.org/Doi> 10.1023/A:1006917509560

82.	Fogel L. *Human Information Processing*: Prentice-Hall; 1967.

83.	Seaver D. *Assessment of Group Preferences and Group Uncertainty for Decision-Making*. California: Social Science Research Institute; 1976.

84.	Staël von Holstein C-AS. Two techniques for assessment of subjective probability distributions — An experimental study. *Acta Psychologica* 1971;**35**:478-94. <http://dx.doi.org/https://doi.org/10.1016/0001-6918(71)90005-9>

85.	Thall PF, Ursino M, Baudouin V, Alberti C, Zohar S. Bayesian treatment comparison using parametric mixture priors computed from elicited histograms. 2019;**28**:404-18. <http://dx.doi.org/10.1177/0962280217726803>

86.	Bornkamp B, Ickstadt K. A Note on B-Splines for Semiparametric Elicitation. *The American Statistician* 2009;**63**:373-7.

87.	Moala FA, O’Hagan A. Elicitation of multivariate prior distributions: A nonparametric Bayesian approach. *Journal of Statistical Planning and Inference* 2010;**140**:1635-55. <http://dx.doi.org/https://doi.org/10.1016/j.jspi.2010.01.004>

88.	Daneshkhah A, Hosseinian-Far A, Sedighi T, Farsi M. Prior elicitation and evaluation of imprecise judgements for bayesian analysis of system reliability. In: *Strategic Engineering for Cloud Computing and Big Data Analytics*; 2017:63-79. <http://dx.doi.org/10.1007/978-3-319-52491-7_4>

89.	Lindley DV, Tversky A, Brown RV. On the Reconciliation of Probability Assessments. *Journal of the Royal Statistical Society: Series A (General)* 1979;**142**:146-62. <http://dx.doi.org/10.2307/2345078>

90.	Wittmann ME, Cooke RM, Rothlisberger JD, Lodge DM. Using Structured Expert Judgment to Assess Invasive Species Prevention: Asian Carp and the Mississippi—Great Lakes Hydrologic Connection. *Environmental Science & Technology* 2014;**48**:2150-6. <http://dx.doi.org/10.1021/es4043098>

91.	Quigley J, Walls L. A Methodology for Constructing Subjective Probability Distributions with Data. In: Dias LC, Morton A, Quigley J, editors. *Elicitation: The science and art of structuring judgement*New York, NY: Springer; 2018:141-70.

92.	Meyer M, Booker J. *Eliciting and Analyzing Expert Judgement: A Practical Guide*: Academic Press; 1991.

93.	Boring R, Gertman D, Joe J, Marble J, Galyean W, Blackwood L*, et al.* Simplified Expert Elicitation Guideline For Risk Assessment Of Operating Events. *US Nuclear Regulatory Commission (NRC)* 2005.

94.	Bolger F, Rowe G. The Aggregation of Expert Judgment: Do Good Things Come to Those Who Weight? *Risk Analysis* 2015;**35**. <http://dx.doi.org/10.1111/risa.12272>

95.	Claxton K, Sculpher M, McCabe C, Briggs A, Akehurst R, Buxton M*, et al.* Probabilistic sensitivity analysis for NICE technology assessment: not an optional extra. *Health Econ* 2005;**14**:339-47. <http://dx.doi.org/10.1002/hec.985>

96.	Akins RB, Tolson H, Cole BR. Stability of response characteristics of a Delphi panel: application of bootstrap data expansion. *BMC medical research methodology* 2005;**5**:37-. <http://dx.doi.org/10.1186/1471-2288-5-37>

97.	Gosling JP. Methods for eliciting expert opinion to inform health technology assessment. 2014.

98.	Winkler RL. The assessment of Prior Distributions in Bayesian Analysis. *Journal of the American Statistical Association* 1967;**62**:776-&. <http://dx.doi.org/Doi> 10.2307/2283671

99.	O'Hagan T, Oakley JE. SHELF: the Sheffield Elicitation Framework (version 3.0). In. UK: School of Mathematics and Statistics, University of Sheffield; 2016.

100.	Gosling JP. *On the elicitation of continuous, symmetric, unimodal distributions* no. arXiv:0805.2044; 2008.

101.	Oakley JE, O'Hagan A. Uncertainty in Prior Elicitations: A Nonparametric Approach. *Biometrika* 2007;**94**:427-41.

102.	Gosling JP, Oakley JE, O'Hagan A. Nonparametric elicitation for heavy-tailed prior distributions. *Bayesian Analysis* 2007;**2**:693-718. <http://dx.doi.org/10.1214/07-ba228>

103.	Morris P. Decision analysis expert use. *Management Science* 1974;**20**:1233-41.

104.	Morris PA. Combining expert judgments: A bayesian approach. *Management Science* 1977;**23**.

105.	Jacobs RA. Methods for combining experts' probability assessments. *Neural Comput* 1995;**7**:867-88.

106.	Lipscomb J, Parmigiani G, Hasselblad V. Combining Expert Judgment by Hierarchical Modeling: An Application to Physician Staffing. *Management Science* 1998;**44**:149-61.

107.	Albert I, Donnet S, Guihenneuc-Jouyaux C, Lowchoy S, L. Mengersen K, Rousseau J. *Combining Expert Opinions in Prior Elicitation*; 2012. <http://dx.doi.org/10.1214/12-BA717>

108.	West M, Crosse J. *Modelling probabilistic agent opinion*; 1992. <http://dx.doi.org/10.2307/2345964>

109.	Gelfand AE, Mallick BK, Dey DK. Modeling Expert Opinion Arising as a Partial Probabilistic Specification. *Journal of the American Statistical Association* 1995;**90**:598-604. <http://dx.doi.org/10.1080/01621459.1995.10476552>

110.	Lichtendahl KC, Grushka-Cockayne Y, Winkler RL. Is It Better to Average Probabilities or Quantiles? *Management Science* 2013;**59**:1594-611. <http://dx.doi.org/10.1287/mnsc.1120.1667>

111.	Bamber JL, Aspinall WP, Cooke RM. A commentary on “how to interpret expert judgment assessments of twenty-first century sea-level rise” by Hylke de Vries and Roderik SW van de Wal. *Climatic Change* 2016;**137**:321-8. <http://dx.doi.org/10.1007/s10584-016-1672-7>

112.	French S. Group consensus probability distributions: A critical survey. In: Bernardo JM, editor. *Bayesian Statistics 2* Amsterdam: North-Holland.; 1985:183-97.

113.	Hammitt JK, Zhang YF. Combining Experts' Judgments: Comparison of Algorithmic Methods Using Synthetic Data. *Risk Analysis* 2013;**33**:109-20. <http://dx.doi.org/10.1111/j.1539-6924.2012.01833.x>

114.	Aspinall WP, Cooke RM. Quantifying scientific uncertainty from expert judgement elicitation. *Risk and Uncertainty Assessment for Natural Hazards* 2013.

115.	Cooke RM, ElSaadany S, Huang X. On the performance of social network and likelihood-based expert weighting schemes. *Reliability Engineering & System Safety* 2008;**93**:745-56. <http://dx.doi.org/10.1016/j.ress.2007.03.017>

116.	Ranjan R, Gneiting T. Combining probability forecasts. *Journal of the Royal Statistical Society: Series B (Statistical Methodology)* 2010;**72**:71-91. <http://dx.doi.org/10.1111/j.1467-9868.2009.00726.x>

117.	Rufo MJ, Martin J, Perez CJ. Log-Linear Pool to Combine Prior Distributions: A Suggestion for a Calibration-Based Approach. *Bayesian Anal* 2012;**7**:411-38. <http://dx.doi.org/10.1214/12-BA714>

118.	Hora SC, Kardeş E. Calibration, sharpness and the weighting of experts in a linear opinion pool. *Annals of Operations Research* 2015;**229**:429-50. <http://dx.doi.org/10.1007/s10479-015-1846-0>

119.	Winkler RL, Murphy AH. “Good” Probability Assessors. *Journal of Applied Meteorology* 1968;**7**:751-8. <http://dx.doi.org/10.1175/1520-0450(1968)007><0751:PA>2.0.CO;2

120.	Quigley J, Colson A, Aspinall W, Cooke RM. Elicitation in the classical model. In: International Series in Operations Research and Management Science; 2018: 15-36. <http://dx.doi.org/10.1007/978-3-319-65052-4_2>

121.	R C, L G. TU Delft expert judgment data base. *Reliability Engineering and System Safety* 2008;**93**:657-74.

122.	Cooke RM. Validating Expert Judgment with the Classical Model. In: Martini C, Boumans M, editors. *Experts and Consensus in Social Science*Cham: Springer International Publishing; 2014:191-212.

123.	Mellers B, Stone E, Murray T, Minster A, Rohrbaugh N, Bishop M*, et al.* Identifying and Cultivating Superforecasters as a Method of Improving Probabilistic Predictions. *Perspectives on Psychological Science* 2015;**10**:267-81. <http://dx.doi.org/10.1177/1745691615577794>

124.	Hanea AM, McBride MF, Burgman MA, Wintle BC. The Value of Performance Weights and Discussion in Aggregated Expert Judgments. *Risk Analysis* 2018;**0**. <http://dx.doi.org/10.1111/risa.12992>

125.	Morgenstern O, Von Neumann J. *Theory of games and economic behavior*: Princeton university press; 1953.

126.	Kahneman D, Egan P. *Thinking, fast and slow*: Farrar, Straus and Giroux New York; 2011.

127.	Reyna VF, Nelson WL, Han PK, Dieckmann NF. How numeracy influences risk comprehension and medical decision making. *Psychological bulletin* 2009;**135**:943.

128.	Tversky A, Kahneman D. Judgment under uncertainty: Heuristics and biases. *science* 1974;**185**:1124-31.

129.	Gigerenzer G, Selten R. *Bounded rationality: The adaptive toolbox*: MIT press; 2002.

130.	Kynn M. The 'heuristics and biases' bias in expert elicitation. *Journal of the Royal Statistical Society Series a-Statistics in Society* 2008;**171**:239-64.

131.	Bojke L, Grigore B, Jankovic D, Peters J, Soares M, Stein K. Informing Reimbursement Decisions Using Cost-Effectiveness Modelling: A Guide to the Process of Generating Elicited Priors to Capture Model Uncertainties. *Pharmacoeconomics* 2017;**35**:867-77. <http://dx.doi.org/10.1007/s40273-017-0525-1>

132.	Bazerman MH, Moore DA. Judgment in managerial decision making. 2008.

133.	McBride MF, Fidler F, Burgman MA. Evaluating the accuracy and calibration of expert predictions under uncertainty: predicting the outcomes of ecological research. *Diversity and Distributions* 2012;**18**:782-94. <http://dx.doi.org/10.1111/j.1472-4642.2012.00884.x>

134.	Tversky A, Kahneman D. Availability: A heuristic for judging frequency and probability. *Cognitive psychology* 1973;**5**:207-32.

135.	Slovic P, Fischhoff B, Lichtenstein S. Perceived risk: psychological factors and social implications. *Proc R Soc Lond A* 1981;**376**:17-34.

136.	Mehle T, Gettys CF, Manning C, Baca S, Fisher S. The availability explanation of excessive plausibility assessments. *Acta Psychologica* 1981;**49**:127-40.

137.	McBride MF, Garnett ST, Szabo JK, Burbidge AH, Butchart SH, Christidis L*, et al.* Structured elicitation of expert judgments for threatened species assessment: a case study on a continental scale using email. *Methods in Ecology and Evolution* 2012;**3**:906-20.

138.	Soll JB, Klayman J. Overconfidence in interval estimates. *Journal of Experimental Psychology: Learning, Memory, and Cognition* 2004;**30**:299.

139.	McKenzie CR, Liersch MJ, Yaniv I. Overconfidence in interval estimates: What does expertise buy you? *Organizational behavior and human decision processes* 2008;**107**:179-91.

140.	Larrick RP. Debiasing. *Blackwell handbook of judgment and decision making* 2004:316-38.

141.	Soll J, Milkman K, Payne J. A user's guide to debiasing. 2014.

142.	Clemen RT, Lichtendahl KC. Debiasing expert overconfidence: A Bayesian calibration model. Sixth International Conference on Probablistic Safety Assessment and Management (PSAM6), abstract no. 1369.

143.	Cooke R, Shrader-Frechette K. *Experts in uncertainty: opinion and subjective probability in science*: Oxford University Press on Demand; 1991.

144.	Lin S-W, Bier VM. A study of expert overconfidence. *Reliability Engineering & System Safety* 2008;**93**:711-21.

145.	Bolger F, Rowe G. There is data, and then there is data: Only experimental evidence will determine the utility of differential weighting of expert judgment. *Risk Analysis* 2015;**35**:21-6.

146.	Haran U, Ritov I, Mellers BA. The role of actively open-minded thinking in information acquisition, accuracy, and calibration. *Judgment and Decision Making* 2013;**8**:188.

147.	Plous S. A comparison of strategies for reducing interval overconfidence in group judgments. *Journal of Applied Psychology* 1995;**80**:443.

148.	Haran U, Moore DA, Morewedge CK. A simple remedy for overprecision in judgment. *Judgment and Decision Making* 2010;**5**:467.

149.	Speirs‐Bridge A, Fidler F, McBride M, Flander L, Cumming G, Burgman M. Reducing overconfidence in the interval judgments of experts. *Risk Analysis* 2010;**30**:512-23.

150.	Teigen KH, Jørgensen M. When 90% confidence intervals are 50% certain: On the credibility of credible intervals. *Applied Cognitive Psychology* 2005;**19**:455-75.

151.	Winman A, Hansson P, Juslin P. Subjective probability intervals: how to reduce overconfidence by interval evaluation. *Journal of Experimental Psychology: Learning, Memory, and Cognition* 2004;**30**:1167.

152.	Ferretti V, Guney S, Montibeller G, von Winterfeldt D. Testing best practices to reduce the overconfidence bias in multi-criteria decision analysis. System Sciences (HICSS), 2016 49th Hawaii International Conference on, abstract no. 1381, p. 1547-55.

153.	Murphy AH, Winkler RL. Probability forecasts: A survey of National Weather Service forecasters. *Bulletin of the American Meteorological Society* 1974;**55**:1449-52.

154.	Martin TG, Burgman MA, Fidler F, Kuhnert PM, Low-Choy S, McBride M*, et al.* Eliciting Expert Knowledge in Conservation Science. *Conservation Biology* 2012;**26**:29-38. <http://dx.doi.org/10.1111/j.1523-1739.2011.01806.x>

155.	Prava VR, Clemen RT, Hobbs BF, Kenney MA. Partition Dependence and Carryover Biases in Subjective Probability Assessment Surveys for Continuous Variables: Model-Based Estimation and Correction. *Decision Analysis* 2016;**13**:51-67. <http://dx.doi.org/10.1287/deca.2015.0323>

156.	Block RA, Harper DR. Overconfidence in estimation: Testing the anchoring-and-adjustment hypothesis. *Organizational behavior and human decision processes* 1991;**49**:188-207.

157.	Schall DL, Doll D, Mohnen A. Caution! Warnings as a Useless Countermeasure to Reduce Overconfidence? An Experimental Evaluation in Light of Enhanced and Dynamic Warning Designs. *Journal of Behavioral Decision Making* 2017;**30**:347-58.

158.	Arkes HR. Costs and benefits of judgment errors: Implications for debiasing. *Psychological bulletin* 1991;**110**:486.

159.	Welsh MB, Begg SH, Bratvold RB. Efficacy of bias awareness in debiasing oil and gas judgments. Proceedings of the Annual Meeting of the Cognitive Science Society, abstract no. 1352.

160.	Morewedge CK, Yoon H, Scopelliti I, Symborski CW, Korris JH, Kassam KS. Debiasing decisions: Improved decision making with a single training intervention. *Policy Insights from the Behavioral and Brain Sciences* 2015;**2**:129-40.

161.	Snyder M, Swann WB. Hypothesis-testing processes in social interaction. *Journal of Personality and social psychology* 1978;**36**:1202.

162.	Nisbett RE, Ross L. Human inference: Strategies and shortcomings of social judgment. 1980.

163.	Downs JS, Shafir E. Why some are perceived as more confident and more insecure, more reckless and more cautious, more trusting and more suspicious, than others: Enriched and impoverished options in social judgment. *Psychonomic bulletin & review* 1999;**6**:598-610.

164.	Abbas AE, Budescu DV, Yu H-T, Haggerty R. A comparison of two probability encoding methods: Fixed probability vs. fixed variable values. *Decision Analysis* 2008;**5**:190-202.

165.	Nemet GF, Anadon LD, Verdolini E. Quantifying the effects of expert selection and elicitation design on experts’ confidence in their judgments about future energy technologies. *Risk Analysis* 2017;**37**:315-30.

166.	Briggs A, Claxton K, Sculpher M. *Decision modelling for health economic evaluation*. Oxford: Oxford University Press; 2006.

167.	Brennan A, Chick SE, Davies R. A taxonomy of model structures for economic evaluation of health technologies. *Health Econ* 2006;**15**:1295-310. <http://dx.doi.org/10.1002/hec.1148>

168.	Cao Q, Buskens E, Feenstra T, Jaarsma T, Hillege H, Postmus D. Continuous-Time Semi-Markov Models in Health Economic Decision Making: An Illustrative Example in Heart Failure Disease Management. *Med Decis Making* 2016;**36**:59-71. <http://dx.doi.org/10.1177/0272989x15593080>

169.	Karnon J, Stahl J, Brennan A, Caro JJ, Mar J, Moller J. Modeling using discrete event simulation: a report of the ISPOR-SMDM Modeling Good Research Practices Task Force--4. *Value Health* 2012;**15**:821-7. <http://dx.doi.org/10.1016/j.jval.2012.04.013>

170.	Davis S, Stevenson M, Tappenden P, Wailoo A. NICE Decision Support Unit Technical Support Documents. In: *NICE DSU Technical Support Document 15: Cost-Effectiveness Modelling Using Patient-Level Simulation*London: National Institute for Health and Care Excellence (NICE)

unless otherwise stated. All rights reserved.; 2014.

171.	Collett D. *Modelling survival data in medical research*: CRC Press; 2015.

172.	Putter H, Fiocco M, Geskus RB. Tutorial in biostatistics: competing risks and multi-state models. 2007;**26**:2389-430. <http://dx.doi.org/10.1002/sim.2712>

173.	Welton NJ, Ades AE. Estimation of Markov Chain Transition Probabilities and Rates from Fully and Partially Observed Data: Uncertainty Propagation, Evidence Synthesis, and Model Calibration. 2005;**25**:633-45. <http://dx.doi.org/10.1177/0272989x05282637>

174.	Sharples LD, Taylor GI, Faddy M. A piecewise-homogeneous Markov chain process of lung transplantation. *J Epidemiol Biostat* 2001;**6**:349-55.

175.	Brard C, Le Teuff G, Le Deley MC, Hampson LV. Bayesian survival analysis in clinical trials: What methods are used in practice? *Clin Trials* 2017;**14**:78-87. <http://dx.doi.org/10.1177/1740774516673362>

176.	Miksad RA, Gonen M, Lynch TJ, Roberts TG, Jr. Interpreting trial results in light of conflicting evidence: a Bayesian analysis of adjuvant chemotherapy for non-small-cell lung cancer. *J Clin Oncol* 2009;**27**:2245-52. <http://dx.doi.org/10.1200/jco.2008.16.2586>

177.	Johnson SR, Tomlinson GA, Hawker GA, Granton JT, Feldman BM. Methods to elicit beliefs for Bayesian priors: a systematic review. *Journal of Clinical Epidemiology* 2010;**63**:355-69. <http://dx.doi.org/10.1016/j.jclinepi.2009.06.003>

178.	Hutton JL, Owens RG. Bayesian Sample Size Calculations and Prior Beliefs About Child Sexual Abuse. *Journal of the Royal Statistical Society Series D (The Statistician)* 1993;**42**:399-404. <http://dx.doi.org/10.2307/2348473>

179.	Johnson NP, Fisher RA, Braunholtz DA, Gillett WR, Lilford RJ. Survey of Australasian clinicians' prior beliefs concerning lipiodol flushing as a treatment for infertility: a Bayesian study. *Aust N Z J Obstet Gynaecol* 2006;**46**:298-304. <http://dx.doi.org/10.1111/j.1479-828X.2006.00596.x>

180.	Lilford R. Formal measurement of clinical uncertainty: prelude to a trial in perinatal medicine. The Fetal Compromise Group. *BMJ (Clinical research ed)* 1994;**308**:111-2.

181.	Wilson ECF, Usher-Smith JA, Emery J, Corrie PG, Walter FM. Expert Elicitation of Multinomial Probabilities for Decision-Analytic Modeling: An Application to Rates of Disease Progression in Undiagnosed and Untreated Melanoma. *Value in Health* 2018;**21**:669-76. <http://dx.doi.org/https://doi.org/10.1016/j.jval.2017.10.009>

182.	Vargas C, Bilbeny N, Balmaceda C, Rodríguez MF, Zitko P, Rojas R*, et al.* Costs and consequences of chronic pain due to musculoskeletal disorders from a health system perspective in Chile. *Pain reports* 2018;**3**:e656-e. <http://dx.doi.org/10.1097/PR9.0000000000000656>

183.	Ren S, Oakley JE. Assurance calculations for planning clinical trials with time-to-event outcomes. *Statistics in medicine* 2014;**33**:31-45. <http://dx.doi.org/10.1002/sim.5916>

184.	Chaloner K, Rhame FS. Quantifying and documenting prior beliefs in clinical trials. *Statistics In Medicine* 2001;**20**:581-600.

185.	Chaloner K, Church T, Louis TA, Matts JP. Graphical Elicitation of a Prior Distribution for a Clinical Trial. *Journal of the Royal Statistical Society Series D (The Statistician)* 1993;**42**:341-53. <http://dx.doi.org/10.2307/2348469>

186.	Freedman LS, Spiegelhalter DJ. The Assessment of the Subjective Opinion and its Use in Relation to Stopping Rules for Clinical Trials. *Journal of the Royal Statistical Society Series D (The Statistician)* 1983;**32**:153-60. <http://dx.doi.org/10.2307/2987606>

187.	Spiegelhalter DJ, Freedman LS, Parmar MK. Applying Bayesian ideas in drug development and clinical trials. *Stat Med* 1993;**12**:1501-11; discussion 13-7.

188.	Parmar MKB, Spiegelhalter DJ, Freedman LS, Committee CS. The chart trials: Bayesian design and monitoring in practice. 1994;**13**:1297-312. <http://dx.doi.org/10.1002/sim.4780131304>

189.	Parmar MK, Griffiths GO, Spiegelhalter DJ, Souhami RL, Altman DG, van der Scheuren E. Monitoring of large randomised clinical trials: a new approach with Bayesian methods. *Lancet* 2001;**358**:375-81. <http://dx.doi.org/10.1016/s0140-6736(01)05558-1>

190.	White IR, Pocock SJ, Wang D. Eliciting and using expert opinions about influence of patient characteristics on treatment effects: a Bayesian analysis of the CHARM trials. *Statistics in Medicine* 2005;**24**:3805-21. <http://dx.doi.org/10.1002/sim.2420>

191.	Singpurwalla ND. An Interactive PC-Based Procedure for Reliability Assessment Incorporating Expert Opinion and Survival Data. *Journal of the American Statistical Association* 1988;**83**:43-51. <http://dx.doi.org/10.2307/2288917>

192.	Claxton KP, Sculpher MJ. Using value of information analysis to prioritise health research: some lessons from recent UK experience. *Pharmacoeconomics* 2006;**24**:1055-68. <http://dx.doi.org/10.2165/00019053-200624110-00003>

193.	Cokely ET, Galesic M, Schulz E, Ghazal S, Garcia-Retamero R. Measuring risk literacy: The Berlin Numeracy Test. *Judgment and Decision Making* 2012;**7**:25-47.

194.	Scott SG, Bruce RA. Decision-Making Style: The Development and Assessment of a New Measure. 1995;**55**:818-31. <http://dx.doi.org/10.1177/0013164495055005017>

195.	Winston C, Cheng J, Allaire J, Xie Y. Shiny:Web Application Framework for R. R package version 1.2.0. In; 2018.

196.	Rowe G, Wright G. The Delphi technique as a forecasting tool: issues and analysis. *International Journal of Forecasting* 1999;**15**:353-75. <http://dx.doi.org/https://doi.org/10.1016/S0169-2070(99)00018-7>

197.	Tetlock P, Gardner D. *Superforecasting: The Art and Science of Prediction*. London: Random House; 2016.

198.	Cooke R. Elicitation in the Classical Model. In: Dias C, Morton A, Quigley J, editors. *Elicitation. The Science and Art of Structuring Judgement.*: Springer International Publishing; 2017.

199.	Spiegelhalter DJ, Myles JP, Jones DR, Abrams KR. Bayesian methods in health technology assessment: a review. *Health Technol Assess* 2000;**4**:1-130.

200.	Harnan SE, Tappenden P, Essat M, Gomersall T, Jon Minton, Wong R*, et al.* Measurement of exhaled nitric oxide concentration in asthma: a systematic review and economic evaluation of NIOX MINO, NIOX VERO and NObreath. *Health Technology Assessment* 2015;**19**.

201.	Soares M, Claxton K, Schulpher M. *Health opportunity costs in the NHS: assessing the implications of uncertainty using elicitation methods with experts*. Universities of Sheffield and York; 2017.

202.	Revie M, Bedford T, Walls L. Evaluation of elicitation methods to quantify Bayes linear models. *Proceedings of the Institution of Mechanical Engineers Part O-Journal of Risk and Reliability* 2010;**224**:322-32. <http://dx.doi.org/10.1243/1748006xjrr304>

203.	Garthwaite PH, Al-Awadhi SA, Elfadaly FG, Jenkinson DJ. Prior distribution elicitation for generalized linear and piecewise-linear models. *Journal of Applied Statistics* 2013;**40**:59-75. <http://dx.doi.org/10.1080/02664763.2012.734794>

204.	Dalal S, Khodyakov D, Srinivasan R, Straus S, Adams J. ExpertLens: A system for eliciting opinions from a large pool of non-collocated experts with diverse knowledge. *Technological Forecasting and Social Change* 2011;**78**:1426-44. <http://dx.doi.org/10.1016/j.techfore.2011.03.021>

205.	James A, Low C, Mengersen K. Elicitator : an expert elicitation tool for regression in ecology. *Environmental Modelling and Software* 2010;**25**:129-45.

206.	Bolger F, Wright G. Use of expert knowledge to anticipate the future: Issues, analysis and directions. *International Journal of Forecasting* 2017;**33**:230-43. <http://dx.doi.org/10.1016/j.ijforecast.2016.11.001>

207.	Bedford T, Quigley J, Walls L. Expert elicitation for reliable system design. *Statistical Science* 2006;**21**:428-50. <http://dx.doi.org/10.1214/088342306000000510>

208.	Krueger T, Page T, Hubacek K, Smith L, Hiscock K. The role of expert opinion in environmental modelling. *Environmental Modelling & Software* 2012;**36**:4-18. <http://dx.doi.org/10.1016/j.envsoft.2012.01.011>

209.	Wisniowski A, Bijak J, Christiansen S, Forster JJ, Keilman N, Raymer J*, et al.* Utilising Expert Opinion to Improve the Measurement of International Migration in Europe. *Journal of Official Statistics* 2013;**29**:583-607. <http://dx.doi.org/10.2478/jos-2013-0041>

210.	Mason AJ, Gomes M, Grieve R, Ulug P, Powell JT, Carpenter J. Development of a practical approach to expert elicitation for randomised controlled trials with missing health outcomes: Application to the IMPROVE trial. *Clinical Trials* 2017;**14**:357-67. <http://dx.doi.org/10.1177/1740774517711442>

211.	Hanea A, McBride M, Burgman M, Wintle B. Classical meets modern in the IDEA protocol for structured expert judgement. *Journal of Risk Research* 2018;**21**:417-33.

212.	Hanea AM, McBride MF, Burgman MA, Wintle BC. Classical meets modern in the IDEA protocol for structured expert judgement. *Journal of Risk Research* 2016; 10.1080/13669877.2016.1215346:1-17. <http://dx.doi.org/10.1080/13669877.2016.1215346>

213.	Hanea AM, McBride MF, Burgman MA, Wintle BC, Fidler F, Flander L*, et al.* InvestigateDiscussEstimateAggregate for structured expert judgement. *International Journal of Forecasting* 2017;**33**:267-79. <http://dx.doi.org/10.1016/j.ijforecast.2016.02.008>

214.	Drescher M, Perera AH, Johnson CJ, Buse LJ, Drew CA, Burgman MA. Toward rigorous use of expert knowledge in ecological research. *Ecosphere* 2013;**4**. <http://dx.doi.org/10.1890/es12-00415.1>

215.	Kuhnert PM, Martin TG, Griffiths SP. A guide to eliciting and using expert knowledge in Bayesian ecological models. *Ecology Letters* 2010;**13**:900-14. <http://dx.doi.org/10.1111/j.1461-0248.2010.01477.x>

216.	Ortiz NR, Wheeler TA, Breeding RJ, Hora S, Meyer MA, Keeney RL. Use of expert judgment in NUREG-1150. *Nuclear Engineering and Design* 1991;**126**:313-31. <http://dx.doi.org/10.1016/0029-5493(91)90023-b>

217.	Bonano EJ, Hora SC, Keeney RL, Winterfeldt Dv. *Elicitation and Use of Expert Judgment in Performance Assessment for High-Level Radioactive Waste Repositories* no. NUREG/CR-541 1, SAND89-1821. Washington D.C.: U.S. Nuclear Regulatory Commission; 1990.

218.	Garthwaite PH, O'Hagan A. Quantifying expert opinion in the UK water industry: an experimental study. *Journal of the Royal Statistical Society Series D-the Statistician* 2000;**49**:455-77. <http://dx.doi.org/10.1111/1467-9884.00246>

219.	Hanea AM, Burgman M, Hemming V. IDEA for uncertainty quantification. In: International Series in Operations Research and Management Science; 2018: 95-117. <http://dx.doi.org/10.1007/978-3-319-65052-4_5>

220.	Seaver DA, von Winterfeldt D, Edwards W. Eliciting subjective probability distributions on continuous variables. *Organizational Behavior and Human Performance* 1978;**21**:379-91. <http://dx.doi.org/https://doi.org/10.1016/0030-5073(78)90061-2>

221.	Murphy AH, Winkler RL. Credible Interval Temperature Forecasting: Some Experimental Results. 1974;**102**:784-94. <http://dx.doi.org/10.1175/1520-0493(1974)102><0784:Citfse>2.0.Co;2

1. Three additional participants had their responses invalidated due to failing to comply with directions for completion.
